# A hybrid experimental and machine learning framework for designing and predicting compressive strength of ultra-high-performance concrete

**DOI:** 10.1038/s41598-026-62257-0

**Published:** 2026-07-28

**Authors:** Mohamed Ayman, Amr ElNemr

**Affiliations:** Civil Engineering Department, German University in Cairo (GUC), Cairo, Egypt

**Keywords:** Ultra-high-performance concrete, Compressive strength, Machine learning, Metaheuristic optimization, Sustainability, Quartz-free concrete, Engineering, Materials science

## Abstract

Ultra-high-performance concrete (UHPC) offers exceptional mechanical and durability properties but often relies on quartz powder, raising sustainability and occupational health concerns. This study introduces an integrated experimental-computational framework for predicting the compressive strength of UHPC and developing quartz-free mixtures. Experimentally, the effects of mixing sequence, sand characteristics, superplasticizer chemistry, and curing regime were investigated, leading to a quartz-free UHPC achieving 136 MPa at 28 days under heat-curing. A dataset of 550 UHPC compressive strength records was compiled, incorporating quantitative mix proportions and categorical variables (cement type, superplasticizer base, fiber type, and specimen geometry). Sixty-three machine learning models from tree-based, boosting, and support vector machine families were optimized using seven meta-heuristic algorithms. The Particle Swarm Optimization-tuned XGBoost model achieved the highest prediction accuracy (R^2^ = 0.897, RMSE = 7.63 MPa), followed by the Differential Evolution-optimized Random Forest (R^2^ = 0.867, RMSE = 8.70 MPa). SHapley Additive exPlanations (SHAP) analysis identified curing age as the most influential predictor after optimization. The proposed framework enables accurate and interpretable UHPC strength prediction and supports the design of safer and more sustainable quartz-free UHPC with reduced experimental effort.

**Supplementary Information:**

The online version contains supplementary material available at 10.1038/s41598-026-62257-0.

## Introduction

Ultra-High-Performance Concrete (UHPC) is an advanced cementitious composite characterized by compressive strengths exceeding 120 MPa, superior durability, and the frequent incorporation of fiber reinforcement^1–3^. Its design philosophy hinges on microstructural enhancement through thermal treatment, pozzolanic reactions, and fiber incorporation to mitigate brittleness^4–6^. Conventional UHPC formulations typically include cement, quartz powder (QP), silica fume (SF), fine quartz sand (QS), superplasticizer (SP), and steel fibers. However, the reliance on fine quartz powder poses sustainability challenges and respirable crystalline silica (RCS) health risks^5,7–9^, prompting the search for safer alternatives. Table 1 provides an overview of the key aspects in designing UHPC.

**Table 1:** The philosophy parameters for the design of UHPC.

Aspect	Key points	References
Core design philosophy	1. Enhance microstructure via thermal treatment 2. Strengthen binder via pozzolanic reactions 3. Integrate fibers to reduce brittleness	^5^
Mixing	Intensive mixing is vital for homogeneity and even fiber dispersion, impacting target strength and flow	^18,19^
Superplasticizer (SP)	Preferred type: Polycarboxylate ether (PCE) for workability at low water-to-binder ratios Inferior types: Sulfonated variants (SNF/SMF) Dosing: Excessive SP can retard setting	^20–23^
Cement type	Common: Ordinary Portland Cement (for reactivity) Alternatives: CEM III/A slag or Calcium Aluminate Cement (CAC) for durability/fast strength, but with trade-offs	^20,24,25^
Supplementary materials	Fly ash, slag, and metakaolin improve sustainability and long-term durability, but often slow early strength	^26,27^
Fibers	Type and proportion significantly influence UHPC performance	^28–30^
Curing	Methods are key to developing microstructure and improving mechanical properties	^31^
Fillers (quartz powder)	Essential (~ 10 μm) in heat-cured UHPC, but poses respirable crystalline silica (RCS) risk, driving search for alternatives	^5,7–9^

The complex, non-linear interactions among UHPC constituents make its behavior difficult to model with traditional empirical methods. Machine learning (ML) offers a powerful alternative for capturing these intricate relationships^10,11^. Algorithms such as Support Vector Machines (SVM)^12^, Decision Trees (DT)^13^, Random Forests (RF)^14^, Gradient Boosting^15^, and Neural Networks^16^ can identify subtle patterns without predefined physical laws. The efficacy of these models is highly dependent on hyperparameter tuning, where metaheuristic optimization techniques inspired by natural, social, or biological processes have proven effective^17^.

Previous ML studies on UHPC strength prediction have often been limited by small datasets, narrow material variability, or the omission of key processing and categorical variables^32–34^. Early work by Abuodeh et al.^35^ utilized an Artificial Neural Network (ANN) with a limited dataset of 110 specimens, achieving a moderate performance (R^2^ = 0.801). In contrast, Zhou et al.^36^ reported a significantly higher R^2^ of 0.997 using an optimized Decision Tree model on a similarly small dataset. Despite these promising results, the limited sample size and narrow material variability in both studies restrict the generalizability of their findings.

Subsequent studies have adopted ensemble and boosting approaches with larger datasets. Qian et al.^37^ applied Gradient Boosting to 626 mixtures (R^2^ = 0.93), while Abdellatief et al.^38^ employed XGBoost on 357 mixtures (R^2^ = 0.901). Li et al.^39^ further enhanced model performance by integrating Random Forest with Snake Optimization (R^2^ = 0.9147), reflecting the increasing adoption of hybrid ML–metaheuristic frameworks. Similarly, Kumar et al.^40^ implemented a Bi-directional Long Short-Term Memory (LSTM) model on 810 mixtures (R^2^ = 0.9464), and Mohsennia et al.^41^ achieved a high R^2^ of 0.99 using CatBoost optimized with the Whale Optimization Algorithm.

Despite these advancements, several critical limitations remain. A key concern is the reliance on small or insufficiently diverse datasets, as observed in Abuodeh et al.^35^ and Zhou et al.^36^, which limits the ability of models to capture the full complexity of UHPC behavior. Additionally, dataset inconsistency poses a significant challenge. Qian et al.^37^ included approximately 56% of data below the 120 MPa threshold typically associated with UHPC, while Wang et al.^42^ incorporated around 35% ordinary concrete data, thereby reducing the relevance of these models for true UHPC applications.

Overall, although high predictive performance is frequently reported (R^2^ > 0.90), such outcomes are often dataset-dependent and may not reflect true model generalizability. This creates a gap between predictive modeling and the complex, interdependent reality of UHPC design. Most of the limitations are gathered into two points: small datasets and narrow materials availability, while the models used lack robustness and generalizability of findings. Table 2 summarizes the limitations and impact of the previous studies on UHPC strength prediction.

**Table 2:** The limitations and impact of the previous studies on UHPC strength prediction.

References	Dataset size (mixtures/samples)	Key limitation(s)	Impact on model relevance/generalizability
Abuodeh et al.^35^	110 samples	Small dataset; narrow material variability	Insufficient to represent the full behavior of UHPC
Zhou et al.^36^	110 samples	Small dataset; narrow material variability	Insufficient to represent the full behavior of UHPC
Qian et al.^37^	626 mixtures	56% of strength values were below the 120 MPa UHPC threshold^1–3^	Reduces the relevance of predictions for genuine UHPC performance
Wang et al.^42^	1328 mixtures	~ 35% of compressive strength data corresponded to ordinary concrete	Makes the dataset unsuitable for specifically modeling UHPC compressive strength

### Research significance

**Fig. 1:**
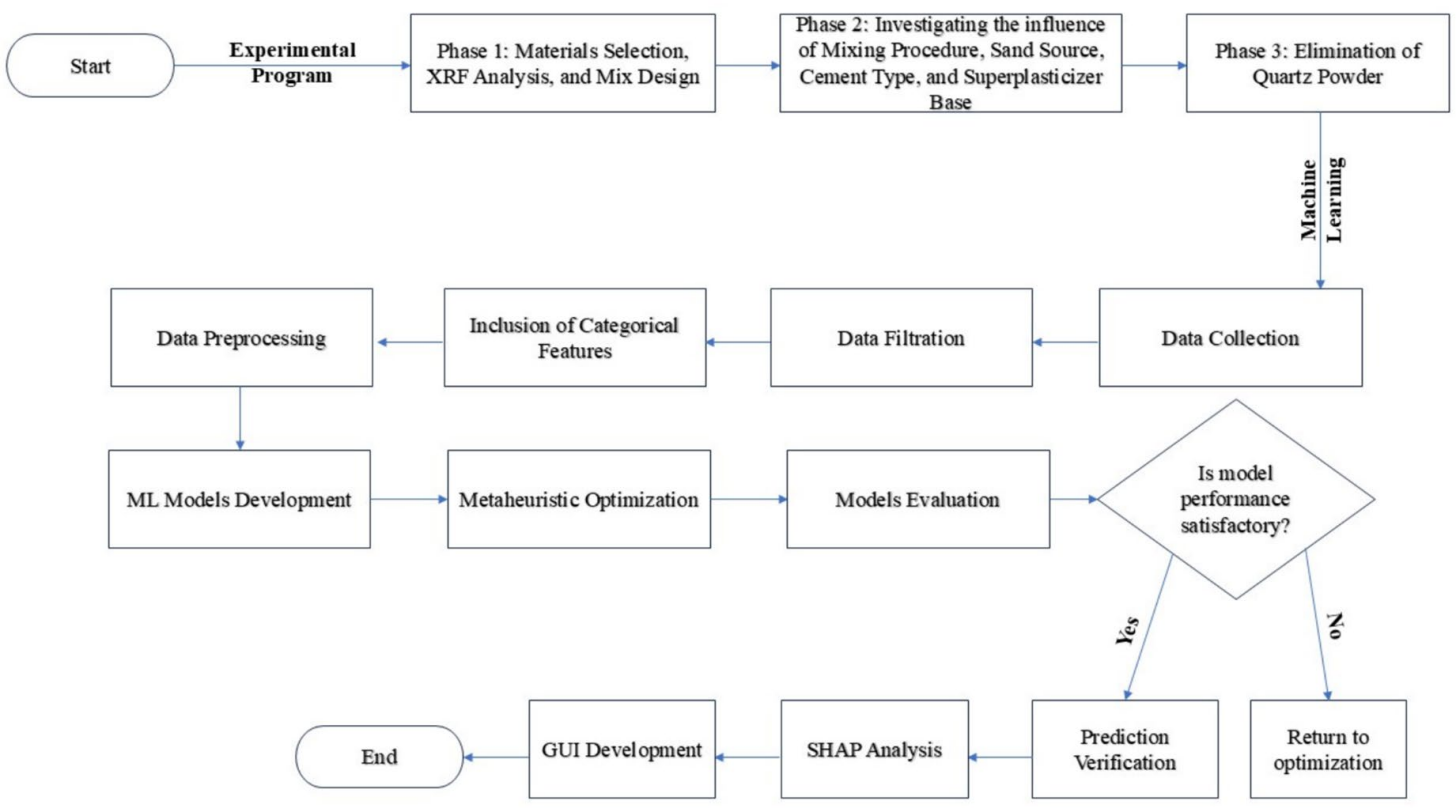
Hybrid experimental and machine learning framework for UHPC strength prediction.

This study addresses the limitations through an integrated approach. First, an experimental program systematically investigates critical factors often omitted in previous studies, such as the mixing procedure including high-energy methods for uniformity^18,19^, detailed material selection (distinguishing between superplasticizer types like Polycarboxylate Ether (PCE) vs. Sulfonated Naphthalene Formaldehyde (SNF)^20–23^ and comparing the influence of different cement types such as Oridinary Portland Cement (OPC) and SNF^20,24,25,27,31,43^, and eliminating the health risks of quartz powder by investigating the influence of total its total elimination, culminating in a high-strength quartz-free UHPC. Second, a comprehensive computational framework is established using a rigorously compiled dataset of 550 UHPC mixtures. The framework employs 63 optimized machine learning models which are divided into 9 base models each optimized with 7 metaheuristic algorithms allowing the investigation of these optimizers on compressive strength’s prediction and incorporates previously neglected but critical categorical features into the dataset such as fiber characteristics beyond simple content^28,29^, Superplasticizer (SP) chemistry, cement type, and curing methods^31^ alongside using SHAP analysis for model interpretability. The outcome is a robust, validated predictive tool that bridges material science and data-driven modeling by integrating key processing, characterization, and durability elements, thereby facilitating efficient and sustainable UHPC development. Figure 1 demonstrates the proposed hybrid experimental and machine learning framework for UHPC compressive strength prediction, including data generation, preprocessing, model development, optimization, evaluation, and interpretation.

## Experimental procedure

### Materials

#### Cements

Sulphate resistance cement 42.5N provided by Heidelberg Materials Egypt was initially used in this study; further in this study, Ordinary Portland Cement (OPC) replaced sulphate resistance cement. The chemical composition for cement, quartz powder, and four silica fume samples is provided in Table 3.

**Table 3:** Chemical composition of cement, quartz powder, and silica fume (SF).

Oxides	Quartz powder	Cement SR 42.5	SikaFume-HR	EFACO (SF)	Modern lab (SF)	Microsilica (SF)
SiO_2_	96.83	20.7	96.49	93.83	93.95	94.89
Al_2_O_3_	0.61	5.84	0.93	0.48	0.46	0.54
Fe_2_O_3_	1.13	6.26	0.5	1.21	1.44	1.03
CaO	0.78	56.09	0.99	1.16	1.1	0.55
MgO	0.12	2.94	0.62	0.37	0.36	0.09
SO_3_	0.02	2.57	0.08	0.09	0.08	0.04
NaO_2_	0.07	0.94	0.42	0.34	0.3	0.21
K_2_O	0.06	0.33	0.64	0.56	0.59	0.09
Cl	0	0.04	0.03	0.02	0	0

#### Silica fume

Four samples were analyzed via XRF; the deciding factor for the silica fume selection was the percentage of SiO_2_. The SikaFume-HR (96.49% SiO_2_) was used in this study. The chemical composition of all four samples is demonstrated in Table 3.

#### Quartz powder

The quartz powder used in this study was provided by Konouz Factory. The quartz powder had a mean particle size of 10 µm. XRF analysis was performed to determine the chemical composition of the quartz powder in Table 3.

#### Sand

Sand from three different sources was evaluated: local Konouz sand (~ 150 µm), sieved German University in Cairo’s Lab sand, and imported ASTM C778 graded sand (150–600 µm).

#### Superplasticizers

A polycarboxylate-based (PCE) admixture (Sika ViscoCrete^®^-3425), which meets the requirements for superplasticizers according to ASTM-C-494 Types G and F and BS EN 934 part 2: 2001, Sikament-NN (naphthalene-based) was also used in this study as it was recommended by the Silica Fume manufacturer. However, the study continued with Sika ViscoCrete^®^-3425. Table 4 provides the properties for both products.

**Table 4:** Technical data of SikaViscocrete-3425 and Sikament NN.

	Sika Viscocrete-3425	Sikament-NN
Base	Aqueous solution of modified polycarboxylates	Naphthalene formaldehyde sulphonate
Appearance/color	Clear liquid	Brown liquid
Density	1.08 kg/lt (ASTM C494)	1.2 kg/lt (ASTM C494)
pH value	4	–
Solid content	40% by weight	40% by weight

#### Mix proportions:

6 mix designs were developed in this study (Table 5); each mix was designated a mix ID that represents the materials used; the first mix (SR-K-P) was adopted from Soliman and Tagnit-Hamou^44^. This mix was selected as a reference due to the similarities between the materials used and the locally available materials. The purpose of this mix was to perform the required trials to determine the optimum mixing procedure and to verify the materials used before targeting the removal of quartz powder. The trials based on this mix developed 4 different mixes with different materials identified as SR-G-P, SR-A-P, SR-A-N, and OP-A-P. The final mix (OP-A-P-Q) was developed after deciding on the optimum mixing procedures and materials for the purpose of removing the quartz powder and to verify the predictions of the machine learning models. This mix’s compressive strength was predicted using the developed GUI before testing in the lab. The naming convention denotes cement type (SR, OP), sand source (K, G, A), SP type (P, N), and quartz exclusion (Q). A constant water-to-binder (w/b) ratio of 0.19 was maintained to isolate the influence of material type (cement, sand, and superplasticizer) on UHPC behavior, while maintaining a constant hydration environment.

**Table 5:** Mix design proportions.

Mix ID	Mix proportions (kg/m^3^)										W/B
	Cement		SF	QP	Sand			Superplasticizer		W	
	SR	OP			K	G	A	P	N		
SR-K-P	810	–	225	243	972	–	–	13	–	196	0.19
SR-G-P	810	–	225	243	–	972	–	13	–	196	0.19
SR-A-P	810	–	225	243	–	–	972	13	–	196	0.19
SR-A-N	810	–	225	243	–	–	972	–	13	196	0.19
OP-A-P	–	810	225	243	–	–	972	13	–	196	0.19
OP-A-P-Q	–	868	241	0	–	–	1047	16	–	210	0.19

### Mixing procedures

Four literature-based mixing procedures^18,19,28,29^ (detailed in Table 6) were trialed using a standard mortar mixer (ELE Digital, 5L). The optimal procedure^19^ involved dry mixing powders, adding 80% water with all SP at low speed (140 rpm), high-speed mixing (285 rpm), adding the remaining water, and final high-speed mixing. This procedure was adopted for all subsequent mixes on 7-day compressive strength and flowability.

**Table 6:** UHPC mixing procedures.

References	Mixer type	Mixing procedure	Mixing duration
^18^	High-energy shear mixer	Dry mix powdered materials for 10 min Gradually add half of the water, containing half the superplasticizer, over 5 min The remaining water and superplasticizer were gradually added during an additional 5 min of mixing	20 Min
^28^	Planetary mixer	Dry mix sand and silica fume for 10 min Add cement and quartz powder, and mix for 5 min Add pre-mixed water and superplasticizer, and mix for 10 min	25 Min
^29^	Pan mixer	Dry mix aggregates for 3 min at a low speed of 100 rpm Add half of the water and mix for 5 min on low speed Mix the other half of the water and all the superplasticizer for 5 min at a high speed of 470 rpm	13 Min
^19^	Planetary mixer	Dry mix all powder materials for 5 min at a low speed Mix 80% of water and all superplasticizer for 2 min at a low speed Mix for 3 min on high speed, then mix the remaining 20% of water added for 2 min at a low speed Mix for 8 min at a high speed	20 Min

### Testing procedure

#### Flowability

**Fig. 2:**
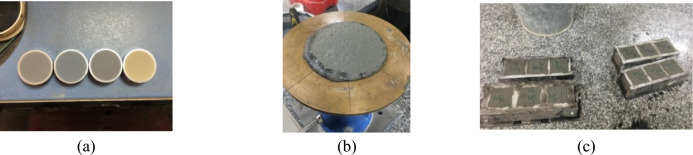
(**a**) Silica fume samples prepared for XRF analysis, (**b**) Flow table test, and (**c**) Compressive strength test cubes.

Assessed using the flow table test per ASTM C1437^45^. The mortar is placed into the cone on a flow table and subjected to 25 drops, spreading laterally. The percentage increase in the base diameter serves as a measure of the mixture’s plasticity and flow characteristics. Figure 2b depicts a representative sample from this study during the test.

#### Compressive strength

Determined on 50 mm cubes at 7 and 28 days using a 2000 kN capacity machine per ASTM C109^46^. Three replicates were tested for each condition. The final quartz-free mix (OP-A-P-Q) was tested under both standard (23 °C) and heat curing (90 °C) regimes (Fig. 2c).

## Machine learning framework

### Dataset development

A unified dataset of 550 UHPC compressive strength records was compiled by merging an existing database^34^ with manually extracted data from peer-reviewed literature^5,18–24,28,29,31–35,37,43,47–67^ spanning between 1996 and 2025. Records with strength < 120 MPa, unrealistic values, or mixes with extraneous components were filtered out.

#### Feature engineering

**Fig. 3:**
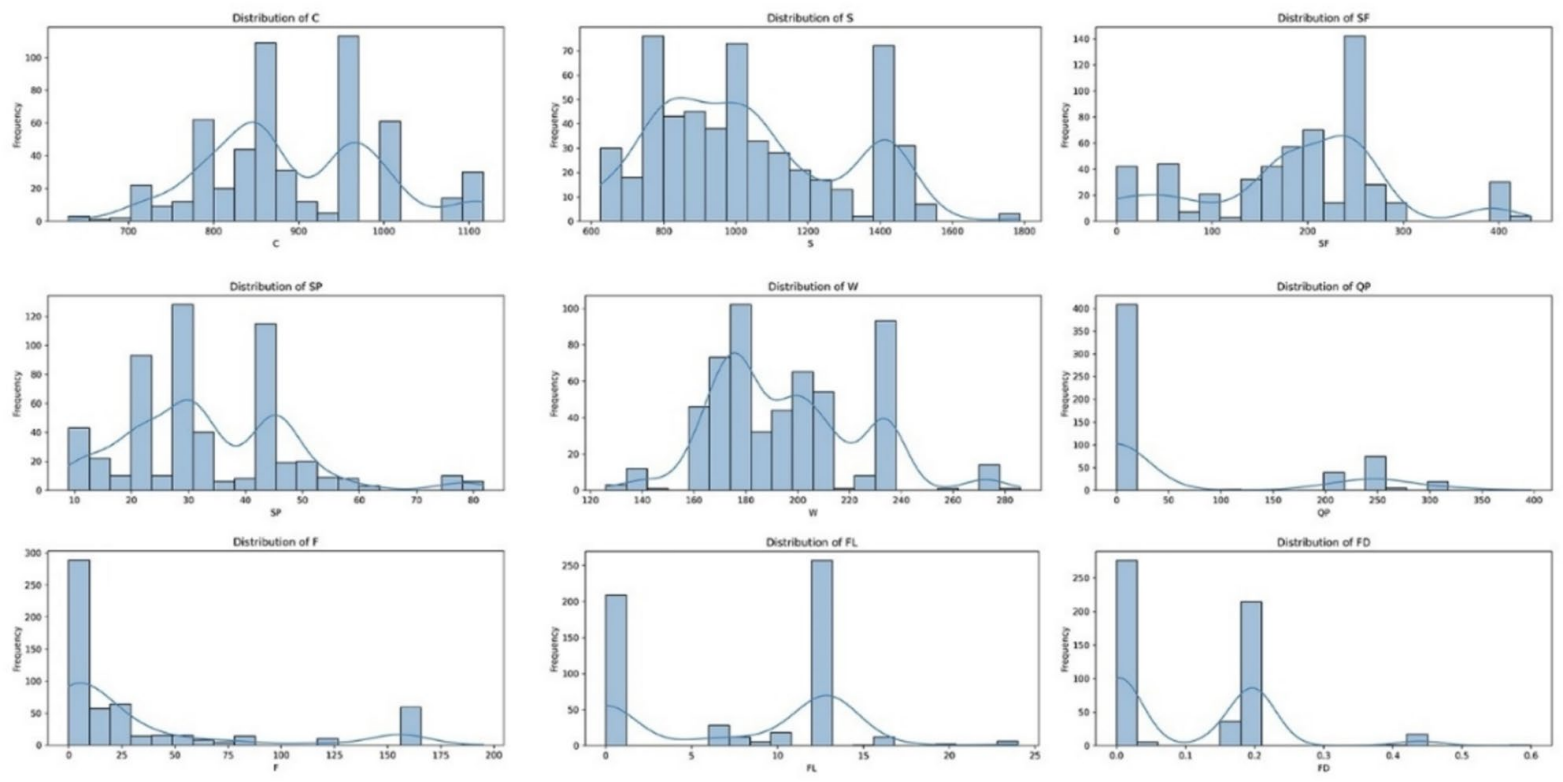
Dataset distribution.

The dataset included 16 input features: the numerical variables of Cement (C), Silica Fume (SF), Sand (S), Quartz Powder (QP), Water (W), Superplasticizer (SP), Fiber content (F), Water-binder ratio (WB), Curing Temperature (T), Fiber Length (FL), Fiber Diameter (FD), and Curing Age (A), as well as the categorical features of Cement Type (CT: 7 types), Superplasticizer Base (SPB: PCE, Polyacrylate, Naphthalene), Fiber Type (FT: None, Steel, Sisal, etc.), and Specimen Type (ST: Cube, Cylinder), which were processed using one-hot encoding. Table 7 and Fig. 3 present a statistical summary and distribution of the dataset features.

**Table 7:** Statistical summary of the dataset’s parameters.

Feature	Count	Mean	Std	Min	25%	50%	75%	Max
C	550.0	893.86	102.77	630.0	828.0	853.0	960.0	1116.7
SF	550.0	189.11	95.99	0.0	149.74	200.0	240.0	433.7
S	550.0	1035.53	255.17	625.35	850.0	1005.0	1231.0	1788.0
QP	550.0	64.77	111.84	0.0	0.0	0.0	200.0	397.0
W	550.0	195.64	28.21	126.0	175.0	196.19	212.5	286.0
SP	550.0	33.42	14.43	9	22.06	30.2	45	81.7
F	550.0	31.13	49.52	0	0	8.53	29.96	195
WB	550.0	0.18	0.02	0.12	0.16	0.2	0.2	0.24
T	550.0	22.83	0.82	20	23	23	23	90
CT	550.0	1	0.04	1	1	1	1	7
SPB	550.0	1	0	1	1	1	1	3
FT	550.0	2.48	2.58	0	0	1	1	5
FL	550.0	7.61	6.37	0	0	12	13	24
FD	550.0	0.11	0.11	0	0	0.02	0.2	0.6
ST	550.0	1	0	1	1	1	1	2
A	550.0	37.22	26.38	3	28	28	56	91
CS	550.0	153.19	21.86	120.1	135.0	149.5	170.58	200.0

C, Cement; SF, Silica fume; S, Sand; QP, Quartz powder; W, Water; SP, Superplasticizer; F, Fiber; WB, Water-binder ratio; T, Curing temperature; CT, Cement type; SPB, Superplasticizer base; FT, Fiber type; FL, Fiber length; FD, Fiber diameter; ST, Specimen type; A, Curing age; CS, Compressive strength; W/C, Water to cement ratio; std, standard deviation.

### Data preprocessing

Outlier removal was performed by applying the Interquartile Range (IQR) method to the numerical features, followed by an 80:20 stratified train-test split. To prepare the data for modeling, numerical features were standardized using Z-score normalization, while categorical features were one-hot encoded; all transformations were implemented through a Column Transformer that was exclusively fitted on the training set to prevent any potential data leakage.

### Model development and optimization

In this work, a total of nine base models were organized into three main groups based on their underlying methodology: tree-based models (Decision Tree (DT), Random Forest (RFR), and Extra Trees (ETR)), boosting models (XGBoost, AdaBoost, CatBoost, and LightGBM), and support vector machine-based models (SVR and NuSVR).

This grouping also aligns with common ensemble learning classifications. Specifically, DT, SVR, and NuSVR are considered single models^68–71^, RFR and ETR fall under bagging-based ensembles, and XGBoost, AdaBoost, CatBoost, and LightGBM are categorized as boosting-based ensembles^72^. A summary and classification of these models is presented in Table 8. The models’ hyperparameters are introduced in Table 9.

**Table 8:** Classification and brief description of the machine learning models used.

Model category	Model	Brief description
Single models	Decision Tree (DT)	A tree-based model that uses decision rules to predict outputs; simple and interpretable but prone to overfitting.^73,74^
	Support Vector Regression (SVR)	Uses kernel functions to model nonlinear relationships within a defined error margin (ε)^75^
	Nu-SupportVector Regression (NuSVR)	A variant of SVR that controls model flexibility using the ν parameter^76^
Bagging ensembles	Random Forest (RFR)	An ensemble of decision trees trained on random subsets of data and features to reduce overfitting^77,78^
	Extra Trees (ETR)	Similar to Random Forest, but uses more randomness in splitting, improving robustness to noise^79^
Boosting ensembles	XGBoost	A gradient boosting method that sequentially improves model performance by correcting residual errors.^80^
	AdaBoost	Combines weak learners sequentially, focusing more on misclassified samples.^81^
	CatBoost	A boosting algorithm with efficient handling of categorical features and reduced overfitting^82^
	LightGBM	A fast, histogram-based boosting model optimized for large datasets^83^

**Table 9:** Machine learning models hyperparameters.

Model	Hyperparameter	Search range
Decision tree	max_depth	5–20
	min_samples_split	2–15
	min_samples_leaf	1–5
Random forest	n_estimators	50–120
	max_depth	5–20
	min_samples_split	2–10
	min_samples_leaf	1–5
	max_features	0.3–0.8
Extra trees	n_estimators	50–120
	max_depth	5–20
	min_samples_split	2–10
	min_samples_leaf	1–5
	max_features	0.3–0.8
XGBoost	n_estimators	50–150
	max_depth	3–8
	learning_rate	0.05–0.2
	subsample	0.7–1.0
	colsample_bytree	0.6–1.0
AdaBoost	n_estimators	30–100
	learning_rate	0.05–0.5
CatBoost	iterations	50–150
	depth	3–6
	learning_rate	0.05–0.2
LightGBM	n_estimators	50–150
	max_depth	3–8
	learning_rate	0.05–0.2
	num_leaves	20–80
	subsample	0.7–1.0
SVR	C	1.0–100.0
	epsilon	0.01–0.1
	gamma	0.001–0.5
NuSVR	nu	0.1–0.9
	C	1.0–100.0
	gamma	0.001–0.5

Each base model was hyperparameter-optimized using seven metaheuristic algorithms^84^: Particle Swarm Optimization (PSO), Genetic Algorithm (GA), Simulated Annealing (SA), Differential Evolution (DE), Artificial Bee Colony (ABC), Harmony Search (HS), and Firefly Algorithm (FFA). This resulted in a total of 63 optimized models. Table 10 describes the seven optimizers used in this study, while Table 11 introduces the hyperparameters used for each optimizer.

**Table 10:** Description of the seven optimizers for tuning the nine models utilized on the data from machine learning.

Tuning models	Description of the model
Metaheuristic optimizers	To tune the nine machine learning models, the following seven metaheuristic optimizers were used separately on each model
Particle Swarm Optimizer (PSO	Inspired by flocking behavior in birds, particles move through the search space based on their own experience and the swarm’s best-known solution to find optimal results^85–87^
Genetic algorithm (GA)	This algorithm is based on natural selection; it explores complex search spaces through a population of evolving solutions^88^
Simulated annealing (SA)	Based on metallurgical annealing, the algorithm initially accepts worse solutions probabilistically to escape local optima, gradually reducing randomness as it “cools”^89^
Differential evolution (DE)	DE combines existing solutions to generate new ones, it maintains diversity and improves the results through crossovers and mutations^90^
Artificial bee colony (ABC)	Mimics the foraging behavior of bees, balancing exploration and exploitation through employed, onlooker, and scout bees^91,92^
Harmony search (HS)	Inspired by musical improvisation, it creates new solutions by combining existing ones, introducing small adjustments and randomness to approach an optimal “harmony” (the optimal solution)^93^
Firefly algorithm (FFA)	A swarm-based method where fireflies are attracted to better solutions, with movement influenced by light intensity and distance^94^

**Table 11:** Metaheuristic optimizers hyperparameters.

Model	Hyperparameter	Value
Particle Swarm Optimization (PSO)	n_particles	25
	max_iter	35
	w	0.6
	c1	1.2
	c2	2.2
Genetic algorithm (GA)	pop_size	24
	generations	35
	mutation_rate	0.25
	crossover_rate	0.75
Firefly algorithm (FFA)	n	22
	max_iter	33
	alpha	0.6
	beta	1.0
	gamma	1.5
Simulated annealing (SA)	max_iter	40
	T0	120
	alpha	0.85
Differential evolution (DE)	pop_size	20
	max_iter	30
	F	0.5
	CR	0.7
Artificial bee colony (ABC)	pop_size	20
	max_iter	30
	limit	5
Harmony search (HS)	hm_size	20
	max_iter	30
	HMCR	0.9
	PAR	0.3
	bw	0.01

### Performance evaluation and interpretability

Models were evaluated using the coefficient of determination (R^2^), Root Mean Square Error (RMSE), Mean Absolute Error (MAE), and Mean Absolute Percentage Error (MAPE). Model interpretability was achieved using Shapley Additive exPlanations (SHAP)^95^.

### Graphical user interface (GUI)

A user-friendly GUI was developed using Gradio for web deployment and Tkinter for desktop deployment. It allows users to input mix parameters, select a pre-trained optimal model (PSO-XGBoost or DE-RF), and obtain immediate compressive strength predictions.

## Results and discussion

### Experimental results

#### Mixing procedure

**Fig. 4:**
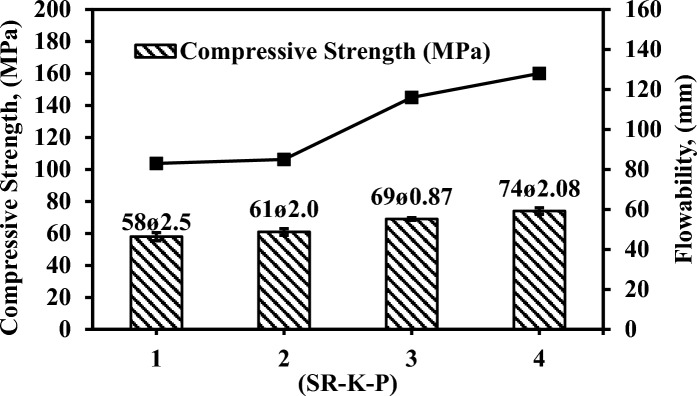
Influence of mixing procedure on compressive strength and flowability.

The mixing procedure significantly affected both flowability and compressive strength of UHPC (Fig. 4). The first procedure^18^ produced a 7-day compressive strength of 58 MPa and a flowability of 83 mm. The second procedure^28^ showed a modest improvement, with compressive strength increasing by 5% (61 MPa) and flowability by 2.5% (85 mm), attributed to the simultaneous addition of water and superplasticizer. A more substantial enhancement was observed in the third procedure^29^, where compressive strength increased by 16% and 12% compared to the first and second procedures, respectively, reaching 69 MPa. Flowability also increased by 29% and 27% (116 mm), likely due to the use of high-speed mixing. The fourth procedure^19^ further improved performance, achieving 74 MPa compressive strength and 128 mm flowability, corresponding to increases of 7% and 10% over the third procedure. Accordingly, the fourth procedure was adopted for subsequent trials. Table 6 summarizes the investigated mixing procedures.

#### Materials optimization and quartz removal

**Fig. 5:**
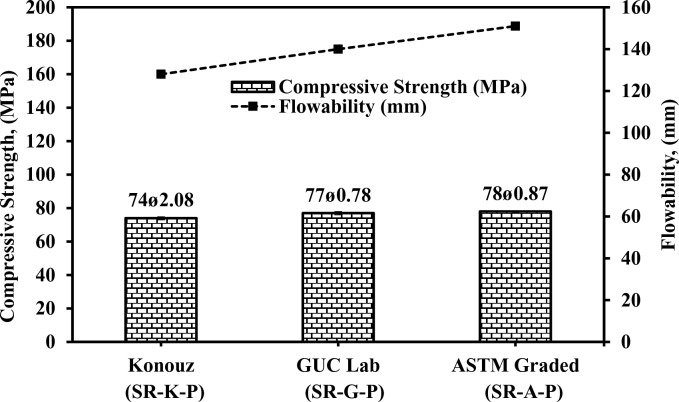
Influence of sand on compressive strength and flowability.

Further improvements in compressive strength required optimization of constituent materials. The initially used sand did not meet the required particle size distribution for UHPC. Replacing it with laboratory-graded sand (150–600 µm) resulted in a 4% increase in compressive strength and a 9% increase in flowability. ASTM C778^96^ graded sand provided a negligible increase in compressive strength (0.02%) but improved flowability by 8%, and was therefore selected for subsequent experiments (Fig. 5).

**Fig. 6:**
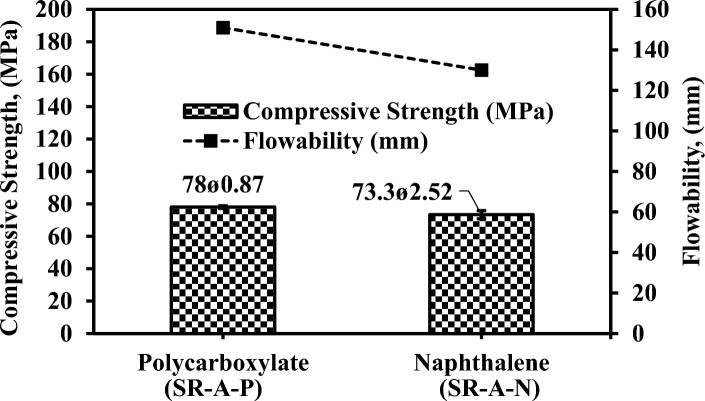
Influence of superplasticizer base on compressive strength and flowability.

The influence of superplasticizer type was also evaluated. Although the silica fume datasheet recommends a naphthalene-based superplasticizer, literature supports the use of polycarboxylate-based alternatives. Experimental results confirmed this, as the naphthalene-based superplasticizer reduced compressive strength by 7% and flowability by 14%. Consequently, a polycarboxylate-based superplasticizer was adopted for the remaining trials (Fig. 6).

**Fig. 7:**
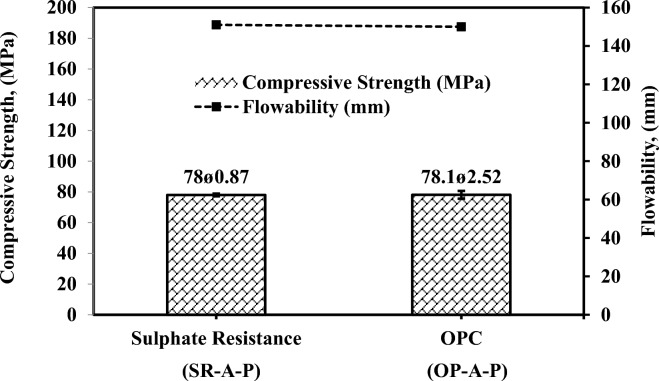
Influence of cement type on compressive strength and flowability.

The effect of cement type was assessed by replacing sulphate-resistant cement with ordinary Portland cement (OPC) from the same supplier. No significant differences in compressive strength or flowability were observed; therefore, OPC was selected due to its broader applicability (Fig. 7).

**Fig. 8:**
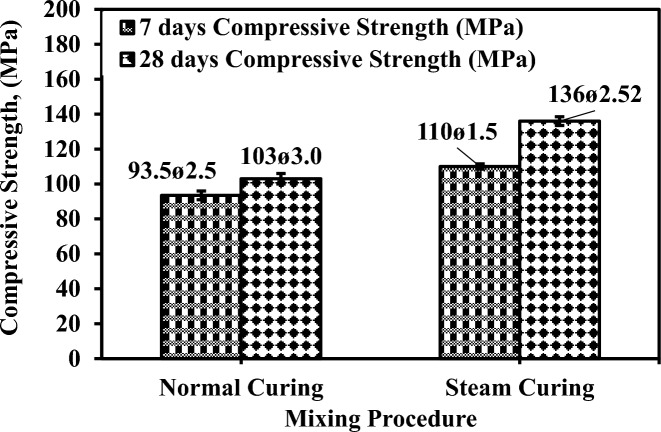
Compressive strength results (7 and 28 days) of quartz-free UHPC mix.

The elimination of quartz powder was subsequently investigated. After adjusting the mix while maintaining a water-to-binder ratio of 0.19, experimental results showed that removing quartz powder increased flowability by 26% and compressive strength by 17%, reaching 93.5 MPa and 103 MPa under normal curing at 7 and 28 days, respectively. Under steam curing, compressive strengths reached 110 MPa at 7 days and 136 MPa at 28 days (Fig. 8).

### Machine learning prediction performance

#### Tree-based models

**Fig. 9:**
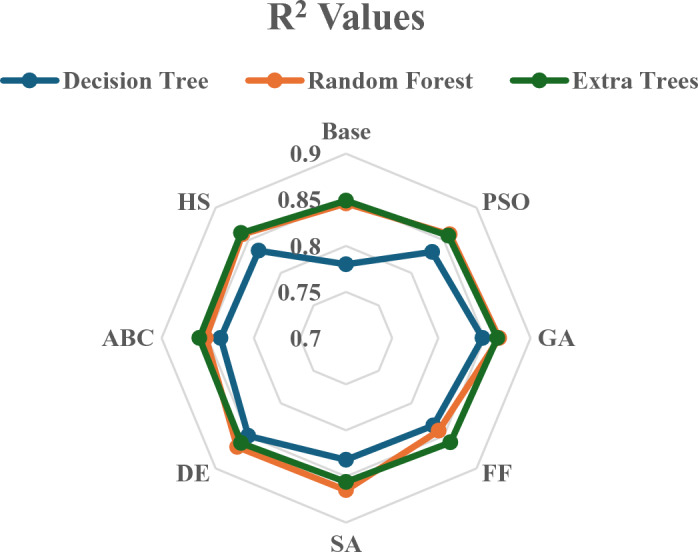
R^2^ values comparison of Tree Models Testing Results.

The DE-optimized Random Forest model delivered the best generalization, with testing R^2^ = 0.867 and RMSE = 8.70 MPa (Table 11). While base DT and ET models showed lower training error, they exhibited signs of overfitting. Figure 9 shows a comparison between the R^2^ results of the testing dataset.

#### Boosting models

**Fig. 10:**
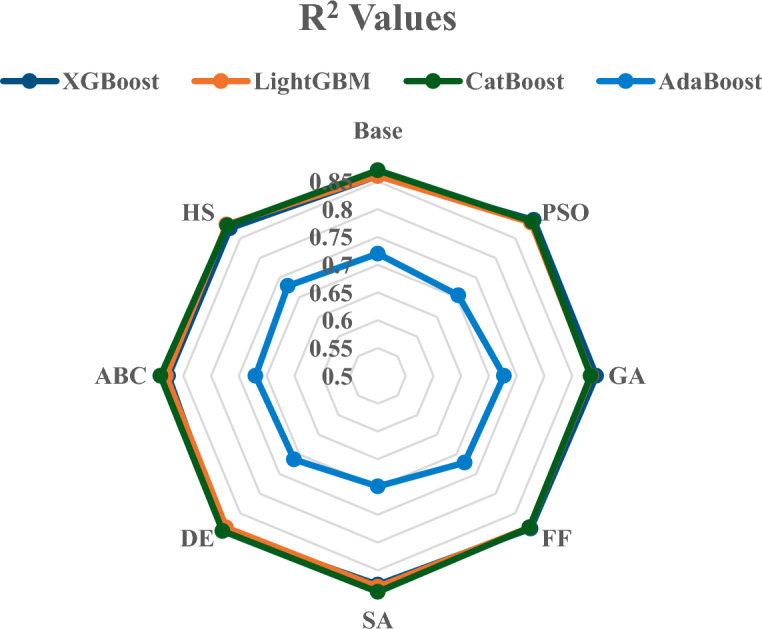
R^2^ values comparison of Boosting Models Testing Results.

The PSO-optimized XGBoost model was the top performer overall, achieving a testing R^2^ of 0.897 and RMSE of 7.63 MPa (Table 12). Other optimizers (GA, DE) also provided significant improvements over base models. AdaBoost was the least effective, even after optimization (Fig. 10).

**Table 12:** Tree models performance metrics (training and testing).

Model	Decision tree				Random forest				Extra trees			
Optimizer	RMSE	MAE	MAPE	R^2^	RMSE	MAE	MAPE	R^2^	RMSE	MAE	MAPE	R^2^
Training dataset												
Base	3.752	1.322	0.009	0.967	4.534	2.752	0.018	0.951	3.752	1.325	0.009	0.967
PSO	5.583	2.155	1.386	0.931	6.711	4.217	2.766	0.9	6.59	3.808	2.474	0.904
GA	8.029	5.292	3.433	0.857	6.211	3.621	2.358	0.915	5.746	2.708	1.756	0.927
FF	5.606	2.232	1.436	0.93	6.448	3.884	2.543	0.908	6.548	3.793	2.473	0.905
SA	5.585	2.17	1.398	0.931	6.356	3.842	2.508	0.911	6.899	4.298	2.808	0.895
DE	5.7	2.379	1.53	0.928	6.312	3.707	2.418	0.912	5.762	2.73	1.773	0.927
ABC	5.606	2.232	1.436	0.93	6.608	4.186	2.741	0.903	5.706	2.665	1.728	0.928
HS	5.583	2.155	1.386	0.931	6.402	3.785	2.483	0.909	6.357	3.656	2.389	0.911
Testing dataset												
Base	9.320	6.038	0.041	0.780	7.788	5.778	0.039	0.846	7.727	5.045	0.034	0.849
PSO	9.751	6.657	4.258	0.832	8.941	6.522	4.184	0.859	9.014	6.172	3.957	0.857
GA	9.3	6.808	4.44	0.848	8.719	6.137	3.943	0.866	8.783	5.807	3.749	0.864
FF	9.721	6.473	4.139	0.834	9.473	6.864	4.42	0.842	8.924	6.177	3.951	0.86
SA	9.753	6.592	4.199	0.832	8.743	6.163	3.96	0.865	9.032	6.307	4.038	0.856
DE	9.214	6.17	3.95	0.85	8.704	6	3.854	0.867	8.89	5.867	3.784	0.861
ABC	9.646	6.525	4.158	0.836	9.179	6.429	4.138	0.852	8.951	5.867	3.771	0.859
HS	9.702	6.543	4.184	0.834	8.945	5.961	3.856	0.859	8.883	6.068	3.918	0.861

#### Support vector machine models

**Fig. 11:**
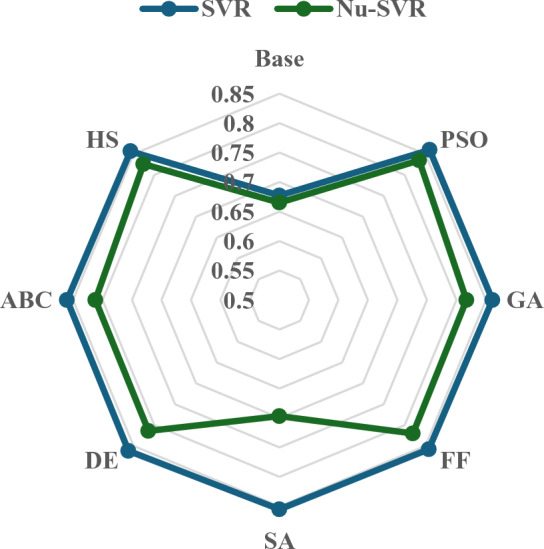
R^2^ values comparison of Support Vector Machine Models Testing Results.

Among SVM models, the DE-optimized SVR performed best (testing R^2^ = 0.862, RMSE = 8.08 MPa), demonstrating the value of metaheuristic tuning for these algorithms (Tables 13, 14, Fig. 11).

**Table 13:** Boosting models performance metrics (training and testing).

Model	XGBoost				LightGBM				CatBoost				AdaBoost			
Optimizer	RMSE	MAE	MAPE	R^2^	RMSE	MAE	MAPE	R^2^	RMSE	MAE	MAPE	R^2^	RMSE	MAE	MAPE	R^2^
Training results																
Non	3.75	1.41	0.93	0.97	5.52	3.84	2.6	0.93	3.96	2.13	1.41	0.96	9.46	8.16	5.51	0.79
PSO	7.5	5.192	3.413	0.876	6.716	4.096	2.687	0.9	6.663	4.247	2.775	0.902	12.27	9.899	6.531	0.667
GA	6.929	4.591	3.03	0.894	6.862	4.259	2.798	0.896	7.182	4.771	3.127	0.886	12.06	9.784	6.476	0.678
FF	6.799	4.42	2.895	0.898	6.897	4.324	2.841	0.895	7.728	5.376	3.538	0.868	12.23	9.915	6.546	0.669
SA	6.497	4.125	2.696	0.907	7.089	4.52	2.97	0.889	6.97	4.605	3.021	0.893	12.5	10.19	6.711	0.655
DE	7.127	4.817	3.176	0.888	6.91	4.346	2.854	0.894	7.594	5.203	3.427	0.872	12.24	9.946	6.592	0.669
ABC	6.774	4.459	2.927	0.899	6.985	4.406	2.89	0.892	7.034	4.617	3.023	0.891	12.05	9.7	6.428	0.679
HS	6.107	3.573	2.325	0.918	6.853	4.282	2.81	0.896	8.185	5.796	3.813	0.852	12.28	9.921	6.553	0.667
Testing results																
Non	7.49	5.1	3.43	0.86	7.41	5.98	4	0.86	7.22	4.93	3.34	0.87	10.6	9.12	6	0.72
PSO	7.632	5.917	3.819	0.897	7.89	5.298	3.42	0.89	7.786	5.651	3.62	0.893	12.94	10.62	6.867	0.705
GA	7.811	5.992	3.885	0.893	8.106	5.567	3.586	0.884	8.153	5.994	3.865	0.883	12.44	10.38	6.758	0.727
FF	7.928	5.9	3.796	0.889	8.034	5.439	3.51	0.886	8.055	6.02	3.883	0.886	12.59	10.44	6.746	0.721
SA	8.395	6.044	3.89	0.876	8.241	5.708	3.692	0.88	7.944	5.904	3.782	0.889	13.07	10.95	7.073	0.699
DE	7.767	5.917	3.781	0.894	8.045	5.565	3.599	0.886	7.71	5.791	3.727	0.895	12.77	10.55	6.824	0.713
ABC	8.356	6.241	4.033	0.877	8.206	5.651	3.636	0.881	7.863	5.82	3.73	0.891	12.61	10.29	6.678	0.72
HS	8.393	5.945	3.807	0.876	8.096	5.497	3.561	0.885	8.152	6.155	3.994	0.883	12.39	10.1	6.521	0.729

**Table 14:** SVM models performance metrics (training and testing).

Model	SVR						NuSVR							
Optimizer	RMSE	MAE	MAPE		R^2^		RMSE			MAE		MAPE		R^2^
Training results														
Non	11.5633	8.5103	0.0565		0.6826		12.4273			9.8803		0.0659		0.6334
PSO	7.439	4.01	2.595		0.865		7.271			3.887		2.519		0.842
GA	7.466	4.065	2.632		0.859		7.536			4.107		2.658		0.823
FF	7.479	4.011	2.593		0.863		7.243			3.988		2.585		0.838
SA	7.545	4.075	2.637		0.86		11.01			8.196		5.395		0.732
DE	7.439	4.01	2.594		0.866		7.265			3.909		2.534		0.829
ABC	7.437	4.015	2.598		0.864		7.275			3.969		2.573		0.819
HS	7.391	3.887	2.51		0.861		7.197			3.82		2.477		0.834
Testing results														
Non	11.3786	8.8087		0.0565		0.6772		11.499	9.5293		0.0626		0.6652	
PSO	8.082	5.55		3.49`		0.86		8.078	5.584		3.507		0.835	
GA	8.094	5.606		3.506		0.861		8.194	5.72		3.578		0.817	
FF	8.196	5.684		3.56		0.858		8.172	5.657		3.545		0.82	
SA	8.261	5.795		3.635		0.855		13.115	9.651		6.106		0.697	
DE	8.082	5.52		3.496		0.862		8.083	5.592		3.511		0.814	
ABC	8.083	5.57		3.494		0.86		8.093	5.612		3.522		0.812	
HS	8.224	5.681		3.566		0.857		8.161	5.623		3.529		0.826

### Model interpretability via SHAP analysis

**Fig. 12:**
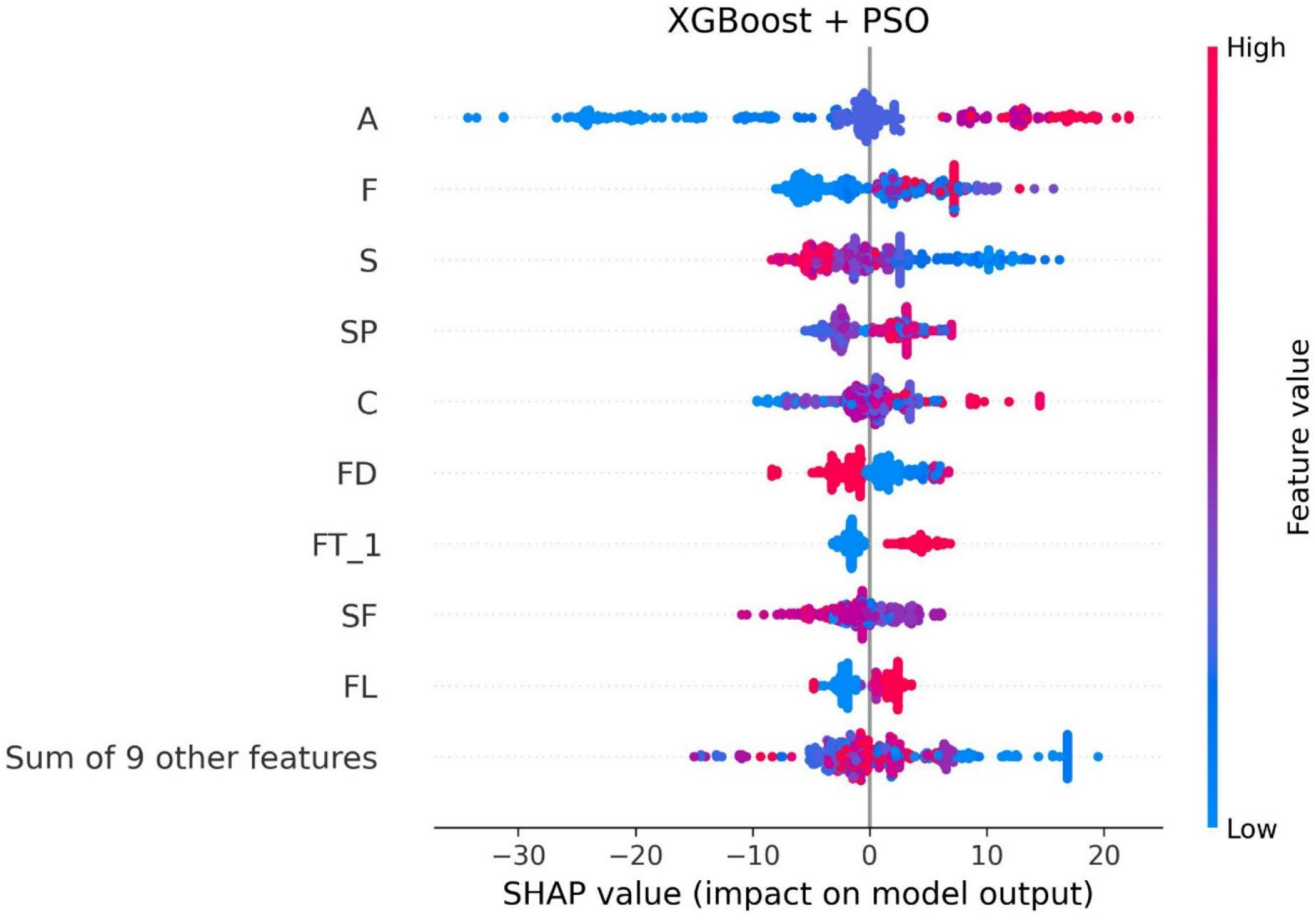
SHAP analysis of PSO-XGBoost model.

Based on SHAP analysis of the optimized models, a consistent hierarchy of feature importance was revealed: Curing Age (A) was the most influential positive predictor, while Fiber Content (F) was the most significant negative predictor. The features Silica Fume (SF), Sand (S), and Superplasticizer (SP) were also consistently identified as key positive drivers. Furthermore, the analysis validated the importance of the categorical variables: Cement Type (CT), Superplasticizer Base (SPB), and Fiber Type (FT), highlighting their necessity for accurate prediction. This ranking aligns with established material science principles, where age drives the hydration process, high fiber volumes can introduce voids and reduce workability, and silica fume and superplasticizers are crucial for effective particle packing and dispersion (Fig. 12).

Among all investigated models, the PSO-Optimized XGBoost model demonstrated the most physically consistent SHAP interpretation, with curing age as the dominant positive contributor, fiber content as the principal negative factor, and silica fume, sand, and superplasticizer showing consistent positive influence. The contribution of categorical variables such as the fiber type (FT) further validated the necessity of mix-design descriptors for accurate UHPC strength prediction. For detailed SHAP figures for each model and optimized model, refer to the supplementary materials, Appendix B.

### Validation and GUI implementation

**Fig. 13:**
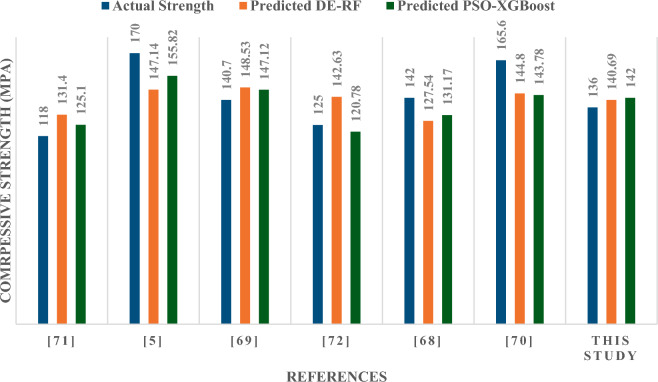
Model validation actual vs. predicted results.

**Fig. 14:**
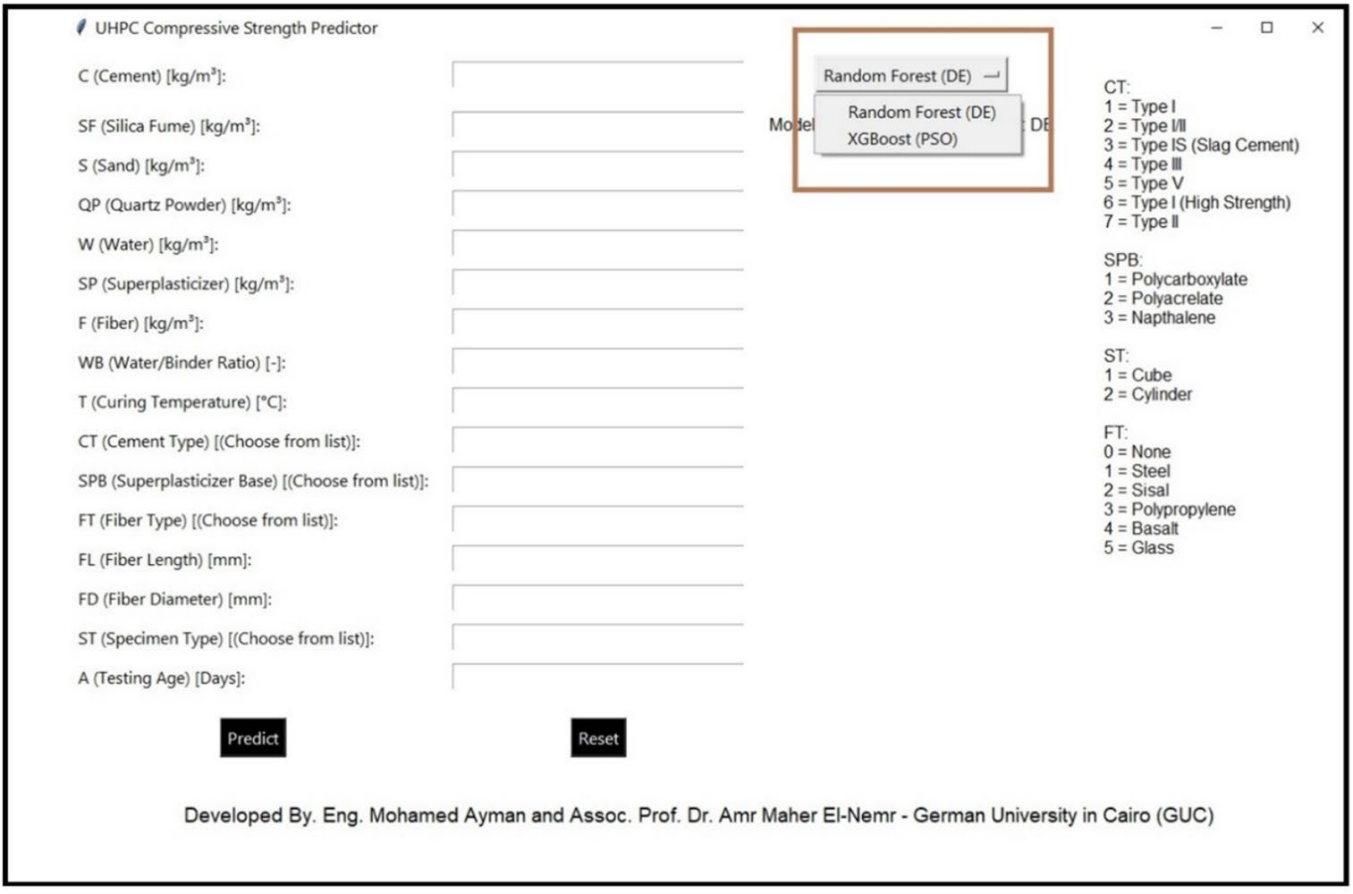
Desktop GUI Layout.

The predictive models were validated against unseen data from literature and the experimental quartz-free mix (Tables 15 and 16). The PSO-XG Boost model showed superior accuracy (94–97%). The developed GUI (Fig. 13) successfully predicted the experimental quartz-free mix strength at 142 MPa (PSO-XG Boost), closely matching the actual 136 MPa (Fig. 14). These results indicate a high prediction accuracy of 95–99% for the developed models, as validated experimentally.

**Table 15:** GUI prediction validation on unseen data.

Study	Actual strength	Predicted DE-RF strength	Predicted PSO-XGBoost strength	RF-accuracy	XGBoost accuracy
^54^	118	131.4	125.1	90	94
^5^	170	147.14	155.82	87	92
^31^	140.7	148.53	147.12	95	96
^55^	125	142.63	120.78	88	97
^22^	142	127.54	131.17	90	92
^53^	165.6	144.8	143.78	87	87
This study	136	140.69	142	97	96

**Table 16:** Validation mixes properties and mix design.

Study	Mix design (kg/m^3^)								Properties					
	C	SF	Sand	QP	W	SP	F	W.B	T	CT	SPB	FT	ST	Age
^54^	880	120	1000	0	200	14	0	0.2	90	1	1	0	1	7
^5^	955	239	1051	0	143	15	0	0.12	20	5	2	0	1	28
^31^	750	158	444	662	189	38	0	0.2	20	4	1	0	1	28
^55^	920	258	1030	0	235.6	31	92	0.2	90	1	1	2	1	7
^22^	870	131	1185	0	200	8	0	0.2	23	7	3	0	1	28
^53^	890	222	799	0	222	30	119	0.2	250	2	1	1	1	28

**Fig. 15:**
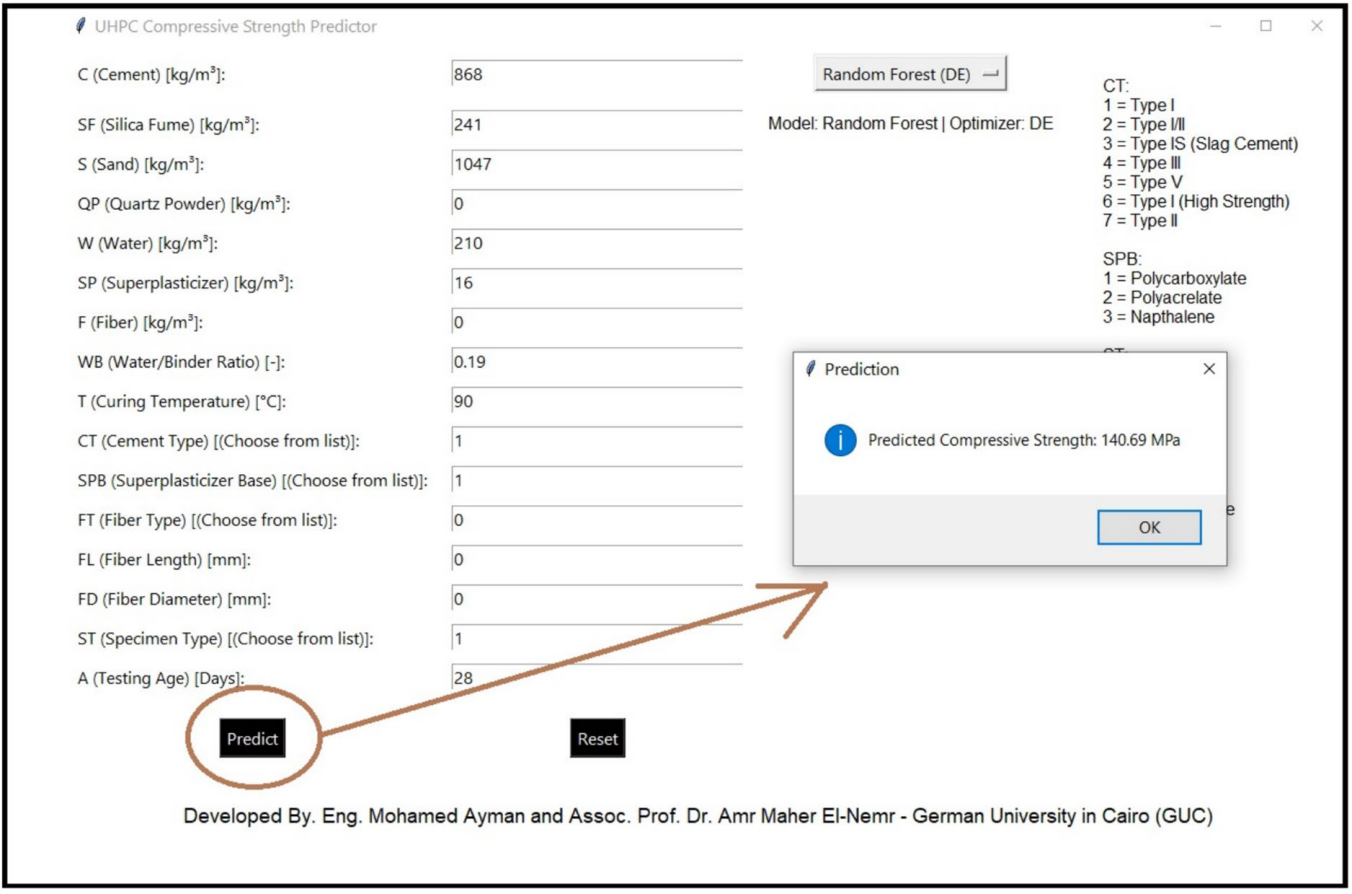
Prediction for mix OP-A-P-Q with DE-random forest.

The GUI was developed to predict the compressive strength of UHPC using the top-achieving prediction models: DE-optimized Random Forest and PSO-optimized XGBoost. The GUI is accessible through the following link: https://huggingface.co/spaces/MohamedAyman314/UHPC_CompressiveStrength_Metaheuristic (Fig. 15).

## Conclusions

This study bridges the gap between laboratory research and industrial application by integrating machine learning with experimental validation to predict the compressive strength of UHPC, nine ML models were developed (Decision Tree, Random Forest, Extra Trees, XGBoost, LightGBM, CatBoost, AdaBoost, SVR, and NuSVR) and optimized with seven metaheuristic optimizers (PSO, GA, SA, HS, ABC, DE, FFA) resulting in a total of 63 optimized ML model, finally experimental verification was performed to verify the adequacy of the prediction model and test the applicability of producing UHPC without quartz powder. Key findings include:

1. Model performance: In the tree-based model category, the Random Forest algorithm optimized using Differential Evolution (DE) demonstrated superior predictive performance compared to its counterparts, achieving an R^2^ value of 0.867, a mean absolute percentage error (MAPE) of 6.000, and a root mean square error (RMSE) of 8.704. Within the boosting model category, the XGBoost algorithm optimized via Particle Swarm Optimization (PSO) exhibited the highest accuracy, with an R^2^ of 0.897, a MAPE of 3.819, and an RMSE of 7.632. For the Support Vector Machine models, the SVR model outperformed the NuSVR model when optimized with DE, attaining an R^2^ value of 0.862, a MAPE of 3.496, and an RMSE of 8.082.
2. Feature importance: Shapley Additive exPlanations (SHAP) analysis revealed that the relative importance of features varied depending on the selected machine learning model and the optimization algorithm employed. Nonetheless, several variables consistently exhibited substantial influence on model predictions; the top three most influential features were curing age (A), fiber content (F), and silica fume content (S).
3. Influence of qualitative features: The inclusion of qualitative features in the prediction dataset, such as cement type (CT), superplasticizer base (SPB), specimen type (ST), and fiber type and properties (FT, FL, FD), proved to enhance predictive accuracy. These variables provide critical contextual information that improves the model’s capacity to generalize, thereby enabling researchers to more accurately anticipate the outcomes of experimental UHPC mix designs.
4. Mixing procedure: Findings from this study demonstrate a significant relationship between mixing procedure and the compressive strength of UHPC. The optimum sequence involves interchanging between low and high speeds and is defined by a critical material addition sequence: initially combining 80% of the water with the entire superplasticizer, followed by the delayed introduction of the remaining 20% of water. By following this procedure, a 22% increase in compressive strength was achieved.
5. Sustainability impact: The development of quartz-free UHPC concrete is applicable. However, the process of development needs high-quality control and several trials. The methodology followed in this study will allow researchers to reduce the number of trials significantly by selecting the optimum materials, mixing procedure, and predicting the compressive strength. The integration of machine learning in predicting the compressive strength of UHPC directly aligns with the objectives of SDG 9 (Industry, Innovation, and Infrastructure). By reducing the reliance on extensive experimental trials, this approach promotes industrial efficiency, fosters technological innovation in construction materials engineering, and supports the creation of resilient and durable infrastructure systems.

## Future work

Future research should expand available datasets by including chemical compositions of constituent materials, enabling more comprehensive analyses and improved predictive accuracy. Incorporating parameters related to mixing procedures, such as time and speed, would further align predictive models with real-world practices. Moreover, evaluating metaheuristic optimizers on neural network architectures could provide deeper insights into their suitability across different model families, and evaluating emerging methods such as Knowledge graph-guided data-driven design of ultra-high-performance concrete.

Advancing in these directions refines the predictive performance of machine learning models and supports their practical application in UHPC design. The methodology developed in the current study establishes a robust foundation for subsequent investigations into the application of metaheuristic algorithms for predicting the compressive strength of UHPC, contributing to the innovative advancement of data-driven approaches in construction materials engineering.

## Supplementary Information

Supplementary Information.

## Data Availability

The developed dataset for this study and any other data needed to replicate this work are publicly available at the following repository: https://zenodo.org/records/21169527.

## Code availability

The codes developed for data processing, model training, and analysis in this study are publicly available at the following repository^102^: 10.5281/zenodo.21169527.
