# Supplementary material for: A hybrid experimental and machine learning framework for designing and predicting compressive strength of ultra-high-performance concrete
Source: Sci Rep. 2026 Jul 28;16:23458. doi: 10.1038/s41598-026-62257-0 (PMC13408577; doi:10.1038/s41598-026-62257-0)
Supplement: Supplementary file 1 — Supplementary Information. [file 41598_2026_62257_MOESM1_ESM.docx]

**Supplementary Materials for Paper titled “****A Hybrid Experimental and Machine Learning Framework for Designing and Predicting Compressive Strength of Ultra-High-Performance Concrete”**

Table Of Contents

[Appendix A: Datasets and Dataset Proceeding Procedures 2](#_Toc233993597)

[Appendix A-1: Raw_Dataset.csv 3](#_Toc233993598)

[Appendix A-2: Dataset_Clean.csv 53](#_Toc233993599)

[Appendix A-3: Filtered_Dataset.csv 63](#_Toc233993600)

[Appendix B 71](#_Toc233993601)

[Appendix C 76](#_Toc233993602)

[Appendix D 77](#_Toc233993603)

[Appendix E 78](#_Toc233993604)

# Appendix A: Datasets and Dataset Proceeding Procedures

In order to reproduce the work performed in this paper; the following steps should be followed. In the provided link under section 7 “**Data Availability Statement**”, there are three “CSV” files available.

The first one is “**Raw_Dataset.csv**”; this is the initial compilation of data that included 1,765 compressive strength (CS) recordings across curing ages ranging from 3 days to 365 days. The following manual filtration process was followed on the “Raw_Dataset.csv” file to enhance data validity; focusing on the main UHPC materials and a compressive strength within the accepted margin, the filtration process was as follows:

1. Exclusion of studies with compressive strength below 120 MPa.
2. Exclusion of studies with extremely high compressive strength (ie. 810 MPa)
3. Exclusion of studies with high materials quantities out of known margins (ie. Cement content = 1500 kg/m2)
4. Exclusion of extremely high curing ages (ie. 365 days)
5. Inclusion of studies that only focus on the following materials; cement, silica fume, sand, quartz powder, superplasticizer, fibers, and water.

After manual filtration, the dataset contained 678 compressive strength records. The 678 records are in the file titled “Dataset_Clean.csv”. Subsequently, additional preprocessing steps, as described in Section 3.3, were performed, resulting in a final dataset titled “Filtered_Dataset.csv” comprising 550 compressive strength records. All datasets can be accessed through the link available in Section 7 “Data Availability Statement”.

The following pages of Appendix A include the datasets mentioned as follows:

1. **Appendix A-1**: Raw_Dataset.csv Initial Dataset with 1765 compressive strength results.
2. **Appendix A-2**: Dataset_Clean.csv Dataset with 678 results after manual filtration process.
3. **Appendix A-3**: Filtered_Dataset.csv Dataset with 550 results after final preprocessing; this dataset is the one used in the code

| Appendix A-1: Raw_Dataset.csv | | | | | | | | | | | | | | | | | | | | | | | |
| --- | --- | --- | --- | --- | --- | --- | --- | --- | --- | --- | --- | --- | --- | --- | --- | --- | --- | --- | --- | --- | --- | --- | --- |
| N | Cement | SF | Sand | NanoSilica | Quartz Powder | Water | Superplasticizer | Fibers | Fly Ash | Slag | MetaK | Lime Powder | Water/Binder Ratio | Curing Type | Curing Temp | Type of Cement | Superplasticizer Base | Fiber Type | Fiber Length | Fiber Diameter | Specimen Type | Testing Age | Compressive Strength |
| 1 | 450 | 50 | 1992 | 0 | 0 | 90 | 18 | 0 | 0 | 0 | 0 | 0 | 0.180 | 1 | 20 | 1 | 1 | 0 | 0 | 0 | 1 | 28 | 131 |
| 2 | 450 | 50 | 1992 | 0 | 0 | 90 | 18 | 0 | 0 | 0 | 0 | 0 | 0.180 | 1 | 20 | 1 | 1 | 0 | 0 | 0 | 1 | 56 | 139 |
| 3 | 426.3 | 0 | 1273.4 | 13.2 | 175.9 | 175.8 | 43.9 | 195 | 0 | 0 | 0 | 263.7 | 0.412 | 1 | 21 | 1 | 1 | 1 | 13 | 0.2 | 1 | 3 | 78.44 |
| 4 | 421.9 | 0 | 1273.4 | 17.6 | 175.9 | 175.8 | 43.9 | 195 | 0 | 0 | 0 | 263.7 | 0.417 | 1 | 21 | 1 | 1 | 1 | 13 | 0.2 | 1 | 3 | 79.69 |
| 5 | 417.5 | 0 | 1273.4 | 22 | 175.9 | 175.8 | 43.9 | 195 | 0 | 0 | 0 | 263.7 | 0.421 | 1 | 21 | 1 | 1 | 1 | 13 | 0.2 | 1 | 3 | 77.82 |
| 6 | 430.7 | 0 | 1273.4 | 8.8 | 175.9 | 175.8 | 43.9 | 0 | 0 | 0 | 0 | 263.7 | 0.408 | 1 | 21 | 1 | 1 | 0 | 0 | 0 | 1 | 7 | 65.98 |
| 7 | 426.3 | 0 | 1273.4 | 13.2 | 175.9 | 175.8 | 43.9 | 0 | 0 | 0 | 0 | 263.7 | 0.412 | 1 | 21 | 1 | 1 | 0 | 0 | 0 | 1 | 7 | 68.82 |
| 8 | 421.9 | 0 | 1273.4 | 17.6 | 175.9 | 175.8 | 43.9 | 0 | 0 | 0 | 0 | 263.7 | 0.417 | 1 | 21 | 1 | 1 | 0 | 0 | 0 | 1 | 7 | 69.13 |
| 9 | 417.5 | 0 | 1273.4 | 22 | 175.9 | 175.8 | 43.9 | 0 | 0 | 0 | 0 | 263.7 | 0.421 | 1 | 21 | 1 | 1 | 0 | 0 | 0 | 1 | 7 | 65.35 |
| 10 | 439.5 | 0 | 1273.4 | 0 | 175.9 | 175.8 | 43.9 | 195 | 0 | 0 | 0 | 263.7 | 0.400 | 1 | 21 | 1 | 1 | 1 | 13 | 0.2 | 1 | 7 | 90.89 |
| 11 | 435.1 | 0 | 1273.4 | 4.4 | 175.9 | 175.8 | 43.9 | 195 | 0 | 0 | 0 | 263.7 | 0.404 | 1 | 21 | 1 | 1 | 1 | 13 | 0.2 | 1 | 7 | 94.63 |
| 12 | 430.7 | 0 | 1273.4 | 8.8 | 175.9 | 175.8 | 43.9 | 195 | 0 | 0 | 0 | 263.7 | 0.408 | 1 | 21 | 1 | 1 | 1 | 13 | 0.2 | 1 | 7 | 96.5 |
| 13 | 630 | 70 | 1788 | 0 | 0 | 126 | 18 | 0 | 0 | 0 | 0 | 0 | 0.180 | 1 | 20 | 1 | 1 | 0 | 0 | 0 | 1 | 56 | 146 |
| 14 | 426.3 | 0 | 1273.4 | 13.2 | 175.9 | 175.8 | 43.9 | 195 | 0 | 0 | 0 | 263.7 | 0.412 | 1 | 21 | 1 | 1 | 1 | 13 | 0.2 | 1 | 7 | 100.86 |
| 15 | 421.9 | 0 | 1273.4 | 17.6 | 175.9 | 175.8 | 43.9 | 195 | 0 | 0 | 0 | 263.7 | 0.417 | 1 | 21 | 1 | 1 | 1 | 13 | 0.2 | 1 | 7 | 103.35 |
| 16 | 417.5 | 0 | 1273.4 | 22 | 175.9 | 175.8 | 43.9 | 195 | 0 | 0 | 0 | 263.7 | 0.421 | 1 | 21 | 1 | 1 | 1 | 13 | 0.2 | 1 | 7 | 98.99 |
| 17 | 435.1 | 0 | 1273.4 | 4.4 | 175.9 | 175.8 | 43.9 | 0 | 0 | 0 | 0 | 263.7 | 0.404 | 1 | 21 | 1 | 1 | 0 | 0 | 0 | 1 | 28 | 77.01 |
| 18 | 430.7 | 0 | 1273.4 | 8.8 | 175.9 | 175.8 | 43.9 | 0 | 0 | 0 | 0 | 263.7 | 0.408 | 1 | 21 | 1 | 1 | 0 | 0 | 0 | 1 | 28 | 81.1 |
| 19 | 426.3 | 0 | 1273.4 | 13.2 | 175.9 | 175.8 | 43.9 | 0 | 0 | 0 | 0 | 263.7 | 0.412 | 1 | 21 | 1 | 1 | 0 | 0 | 0 | 1 | 28 | 88.66 |
| 20 | 421.9 | 0 | 1273.4 | 17.6 | 175.9 | 175.8 | 43.9 | 0 | 0 | 0 | 0 | 263.7 | 0.417 | 1 | 21 | 1 | 1 | 0 | 0 | 0 | 1 | 28 | 91.18 |
| 21 | 417.5 | 0 | 1273.4 | 22 | 175.9 | 175.8 | 43.9 | 0 | 0 | 0 | 0 | 263.7 | 0.421 | 1 | 21 | 1 | 1 | 0 | 0 | 0 | 1 | 28 | 86.46 |
| 22 | 439.5 | 0 | 1273.4 | 0 | 175.9 | 175.8 | 43.9 | 195 | 0 | 0 | 0 | 263.7 | 0.400 | 1 | 21 | 1 | 1 | 1 | 13 | 0.2 | 1 | 28 | 113.93 |
| 23 | 435.1 | 0 | 1273.4 | 4.4 | 175.9 | 175.8 | 43.9 | 195 | 0 | 0 | 0 | 263.7 | 0.404 | 1 | 21 | 1 | 1 | 1 | 13 | 0.2 | 1 | 28 | 120.16 |
| 24 | 810 | 90 | 1539 | 0 | 0 | 162 | 18 | 0 | 0 | 0 | 0 | 0 | 0.180 | 1 | 20 | 1 | 1 | 0 | 0 | 0 | 1 | 56 | 150 |
| 25 | 430.7 | 0 | 1273.4 | 8.8 | 175.9 | 175.8 | 43.9 | 195 | 0 | 0 | 0 | 263.7 | 0.408 | 1 | 21 | 1 | 1 | 1 | 13 | 0.2 | 1 | 28 | 126.38 |
| 26 | 594.2 | 0 | 1282.3 | 24.8 | 0 | 176.9 | 44.2 | 0 | 0 | 0 | 0 | 265.3 | 0.298 | 1 | 21 | 1 | 1 | 0 | 0 | 0 | 1 | 28 | 98.89 |
| 27 | 594.2 | 0 | 1282.3 | 24.8 | 0 | 176.9 | 44.2 | 156 | 0 | 0 | 0 | 265.3 | 0.298 | 1 | 21 | 1 | 1 | 1 | 13 | 0.2 | 1 | 28 | 139.44 |
| 28 | 594.2 | 0 | 1282.3 | 24.8 | 0 | 176.9 | 44.2 | 156 | 0 | 0 | 0 | 265.3 | 0.298 | 1 | 21 | 1 | 1 | 1 | 13 | 0.2 | 1 | 28 | 141.67 |
| 29 | 594.2 | 0 | 1282.3 | 24.8 | 0 | 176.9 | 44.2 | 156 | 0 | 0 | 0 | 265.3 | 0.298 | 1 | 21 | 1 | 1 | 1 | 13 | 0.2 | 1 | 28 | 129.44 |
| 30 | 594.2 | 0 | 1282.3 | 24.8 | 0 | 176.9 | 44.2 | 156 | 0 | 0 | 0 | 265.3 | 0.298 | 1 | 21 | 1 | 1 | 1 | 13 | 0.2 | 1 | 28 | 124.44 |
| 31 | 594.2 | 0 | 1282.3 | 24.8 | 0 | 176.9 | 44.2 | 156 | 0 | 0 | 0 | 265.3 | 0.298 | 1 | 21 | 1 | 1 | 1 | 13 | 0.2 | 1 | 28 | 120.56 |
| 32 | 594.2 | 0 | 1282.3 | 24.8 | 0 | 176.9 | 44.2 | 156 | 0 | 0 | 0 | 265.3 | 0.298 | 1 | 21 | 1 | 1 | 1 | 13 | 0.2 | 1 | 28 | 129.01 |
| 33 | 594.2 | 0 | 1282.3 | 24.8 | 0 | 176.9 | 44.2 | 156 | 0 | 0 | 0 | 265.3 | 0.298 | 1 | 21 | 1 | 1 | 1 | 13 | 0.2 | 1 | 28 | 132.96 |
| 34 | 594.2 | 0 | 1282.3 | 24.8 | 0 | 176.9 | 44.2 | 156 | 0 | 0 | 0 | 265.3 | 0.298 | 1 | 21 | 1 | 1 | 1 | 13 | 0.2 | 1 | 28 | 129.58 |
| 35 | 810 | 90 | 1539 | 0 | 0 | 162 | 18 | 0 | 0 | 0 | 0 | 0 | 0.180 | 1 | 20 | 1 | 1 | 0 | 0 | 0 | 1 | 56 | 150 |
| 36 | 594.2 | 0 | 1282.3 | 24.8 | 0 | 176.9 | 44.2 | 156 | 0 | 0 | 0 | 265.3 | 0.298 | 1 | 21 | 1 | 1 | 1 | 13 | 0.2 | 1 | 28 | 136.34 |
| 37 | 594.2 | 0 | 1282.3 | 24.8 | 0 | 176.9 | 44.2 | 0 | 0 | 0 | 0 | 265.3 | 0.298 | 1 | 21 | 1 | 1 | 0 | 0 | 0 | 1 | 7 | 86.11 |
| 38 | 594.2 | 0 | 1282.3 | 24.8 | 0 | 176.9 | 44.2 | 156 | 0 | 0 | 0 | 265.3 | 0.298 | 1 | 21 | 1 | 1 | 1 | 13 | 0.2 | 1 | 7 | 113.89 |
| 39 | 594.2 | 0 | 1282.3 | 24.8 | 0 | 176.9 | 44.2 | 156 | 0 | 0 | 0 | 265.3 | 0.298 | 1 | 21 | 1 | 1 | 1 | 13 | 0.2 | 1 | 7 | 117.22 |
| 40 | 594.2 | 0 | 1282.3 | 24.8 | 0 | 176.9 | 44.2 | 156 | 0 | 0 | 0 | 265.3 | 0.298 | 1 | 21 | 1 | 1 | 1 | 13 | 0.2 | 1 | 7 | 114.44 |
| 41 | 594.2 | 0 | 1282.3 | 24.8 | 0 | 176.9 | 44.2 | 156 | 0 | 0 | 0 | 265.3 | 0.298 | 1 | 21 | 1 | 1 | 1 | 13 | 0.2 | 1 | 7 | 112.22 |
| 42 | 594.2 | 0 | 1282.3 | 24.8 | 0 | 176.9 | 44.2 | 156 | 0 | 0 | 0 | 265.3 | 0.298 | 1 | 21 | 1 | 1 | 1 | 13 | 0.2 | 1 | 7 | 111.11 |
| 43 | 753 | 188 | 1129 | 0 | 0 | 180.72 | 27 | 0 | 183 | 0 | 0 | 0 | 0.192 | 1 | 20 | 1 | 1 | 0 | 0 | 0 | 1 | 28 | 166 |
| 44 | 745 | 186 | 1118 | 0 | 0 | 171.35 | 27 | 0 | 181 | 0 | 0 | 0 | 0.184 | 1 | 20 | 1 | 1 | 0 | 0 | 0 | 1 | 28 | 162 |
| 45 | 778 | 194 | 1167 | 0 | 0 | 178.94 | 28 | 0 | 189 | 0 | 0 | 0 | 0.184 | 1 | 20 | 1 | 1 | 0 | 0 | 0 | 1 | 28 | 181 |
| 46 | 630 | 90 | 1539 | 0 | 0 | 162 | 18 | 0 | 0 | 180 | 0 | 0 | 0.225 | 1 | 20 | 1 | 1 | 0 | 0 | 0 | 1 | 56 | 141 |
| 47 | 758 | 190 | 1138 | 0 | 0 | 166.76 | 27 | 0 | 184 | 0 | 0 | 0 | 0.176 | 1 | 20 | 1 | 1 | 0 | 0 | 0 | 1 | 28 | 155 |
| 48 | 745 | 186 | 1118 | 0 | 0 | 171.35 | 27 | 0 | 181 | 0 | 0 | 0 | 0.184 | 1 | 20 | 1 | 1 | 0 | 0 | 0 | 1 | 28 | 159 |
| 49 | 890 | 0 | 1231 | 0 | 0 | 178 | 30.2 | 0 | 0 | 0 | 0 | 0 | 0.200 | 1 | 21 | 1 | 1 | 0 | 0 | 0 | 1 | 1 | 59 |
| 50 | 890 | 0 | 1231 | 0 | 0 | 178 | 30.2 | 0 | 0 | 0 | 0 | 0 | 0.200 | 1 | 21 | 1 | 1 | 0 | 0 | 0 | 1 | 1 | 70.7 |
| 51 | 845.5 | 44.5 | 1231 | 0 | 0 | 178 | 30.2 | 0 | 0 | 0 | 0 | 0 | 0.200 | 1 | 21 | 1 | 1 | 0 | 0 | 0 | 1 | 1 | 73.2 |
| 52 | 801 | 89 | 1231 | 0 | 0 | 178 | 30.2 | 0 | 0 | 0 | 0 | 0 | 0.200 | 1 | 21 | 1 | 1 | 0 | 0 | 0 | 1 | 1 | 75.7 |
| 53 | 756.5 | 133.5 | 1231 | 0 | 0 | 178 | 30.2 | 0 | 0 | 0 | 0 | 0 | 0.200 | 1 | 21 | 1 | 1 | 0 | 0 | 0 | 1 | 1 | 70.5 |
| 54 | 712 | 178 | 1231 | 0 | 0 | 178 | 30.2 | 0 | 0 | 0 | 0 | 0 | 0.200 | 1 | 21 | 1 | 1 | 0 | 0 | 0 | 1 | 1 | 62.1 |
| 55 | 845.5 | 44.5 | 1231 | 0 | 0 | 178 | 30.2 | 0 | 0 | 0 | 0 | 0 | 0.200 | 1 | 21 | 1 | 1 | 0 | 0 | 0 | 1 | 1 | 77.8 |
| 56 | 712 | 0 | 1231 | 0 | 0 | 178 | 30.2 | 0 | 178 | 0 | 0 | 0 | 0.250 | 1 | 21 | 1 | 1 | 0 | 0 | 0 | 1 | 1 | 53.7 |
| 57 | 450 | 90 | 1539 | 0 | 0 | 162 | 18 | 0 | 0 | 360 | 0 | 0 | 0.300 | 1 | 20 | 1 | 1 | 0 | 0 | 0 | 1 | 56 | 133 |
| 58 | 667.5 | 44.5 | 1231 | 0 | 0 | 178 | 30.2 | 0 | 178 | 0 | 0 | 0 | 0.250 | 1 | 21 | 1 | 1 | 0 | 0 | 0 | 1 | 1 | 52.8 |
| 59 | 1009 | 0 | 1231 | 0 | 0 | 201.8 | 34.2 | 0 | 0 | 0 | 0 | 0 | 0.200 | 1 | 21 | 1 | 1 | 0 | 0 | 0 | 1 | 1 | 72.8 |
| 60 | 1009 | 0 | 1231 | 0 | 0 | 201.8 | 34.2 | 0 | 0 | 0 | 0 | 0 | 0.200 | 1 | 21 | 1 | 1 | 0 | 0 | 0 | 1 | 1 | 73.2 |
| 61 | 958.55 | 50.45 | 1231 | 0 | 0 | 201.8 | 34.2 | 0 | 0 | 0 | 0 | 0 | 0.200 | 1 | 21 | 1 | 1 | 0 | 0 | 0 | 1 | 1 | 80.1 |
| 62 | 756.75 | 50.45 | 1231 | 0 | 0 | 201.8 | 34.2 | 0 | 201.8 | 0 | 0 | 0 | 0.250 | 1 | 21 | 1 | 1 | 0 | 0 | 0 | 1 | 1 | 53.1 |
| 63 | 890 | 0 | 1231 | 0 | 0 | 178 | 30.2 | 0 | 0 | 0 | 0 | 0 | 0.200 | 1 | 21 | 1 | 1 | 0 | 0 | 0 | 1 | 7 | 95.7 |
| 64 | 890 | 0 | 1231 | 0 | 0 | 178 | 30.2 | 0 | 0 | 0 | 0 | 0 | 0.200 | 1 | 21 | 1 | 1 | 0 | 0 | 0 | 1 | 7 | 97.4 |
| 65 | 845.5 | 44.5 | 1231 | 0 | 0 | 178 | 30.2 | 0 | 0 | 0 | 0 | 0 | 0.200 | 1 | 21 | 1 | 1 | 0 | 0 | 0 | 1 | 7 | 105.7 |
| 66 | 801 | 89 | 1231 | 0 | 0 | 178 | 30.2 | 0 | 0 | 0 | 0 | 0 | 0.200 | 1 | 21 | 1 | 1 | 0 | 0 | 0 | 1 | 7 | 102.6 |
| 67 | 756.5 | 133.5 | 1231 | 0 | 0 | 178 | 30.2 | 0 | 0 | 0 | 0 | 0 | 0.200 | 1 | 21 | 1 | 1 | 0 | 0 | 0 | 1 | 7 | 96.8 |
| 68 | 630 | 90 | 1539 | 0 | 0 | 126 | 18 | 0 | 0 | 180 | 0 | 0 | 0.175 | 1 | 20 | 1 | 1 | 0 | 0 | 0 | 1 | 56 | 159 |
| 69 | 712 | 178 | 1231 | 0 | 0 | 178 | 30.2 | 0 | 0 | 0 | 0 | 0 | 0.200 | 1 | 21 | 1 | 1 | 0 | 0 | 0 | 1 | 7 | 95.9 |
| 70 | 845.5 | 44.5 | 1231 | 0 | 0 | 178 | 30.2 | 0 | 0 | 0 | 0 | 0 | 0.200 | 1 | 21 | 1 | 1 | 0 | 0 | 0 | 1 | 7 | 106.1 |
| 71 | 712 | 0 | 1231 | 0 | 0 | 178 | 30.2 | 0 | 178 | 0 | 0 | 0 | 0.250 | 1 | 21 | 1 | 1 | 0 | 0 | 0 | 1 | 7 | 99.2 |
| 72 | 623 | 0 | 1231 | 0 | 0 | 178 | 30.2 | 0 | 267 | 0 | 0 | 0 | 0.286 | 1 | 21 | 1 | 1 | 0 | 0 | 0 | 1 | 7 | 101.2 |
| 73 | 534 | 0 | 1231 | 0 | 0 | 178 | 30.2 | 0 | 356 | 0 | 0 | 0 | 0.333 | 1 | 21 | 1 | 1 | 0 | 0 | 0 | 1 | 7 | 75.8 |
| 74 | 667.5 | 44.5 | 1231 | 0 | 0 | 178 | 30.2 | 0 | 178 | 0 | 0 | 0 | 0.250 | 1 | 21 | 1 | 1 | 0 | 0 | 0 | 1 | 7 | 92.8 |
| 75 | 1009 | 0 | 1231 | 0 | 0 | 201.8 | 34.2 | 0 | 0 | 0 | 0 | 0 | 0.200 | 1 | 21 | 1 | 1 | 0 | 0 | 0 | 1 | 7 | 102.8 |
| 76 | 1009 | 0 | 1231 | 0 | 0 | 201.8 | 34.2 | 0 | 0 | 0 | 0 | 0 | 0.200 | 1 | 21 | 1 | 1 | 0 | 0 | 0 | 1 | 7 | 102.3 |
| 77 | 958.55 | 50.45 | 1231 | 0 | 0 | 201.8 | 34.2 | 0 | 0 | 0 | 0 | 0 | 0.200 | 1 | 21 | 1 | 1 | 0 | 0 | 0 | 1 | 7 | 102.8 |
| 78 | 756.75 | 50.45 | 1231 | 0 | 0 | 201.8 | 34.2 | 0 | 201.8 | 0 | 0 | 0 | 0.250 | 1 | 21 | 1 | 1 | 0 | 0 | 0 | 1 | 7 | 101.5 |
| 79 | 630 | 90 | 1539 | 0 | 0 | 144 | 18 | 0 | 0 | 180 | 0 | 0 | 0.200 | 1 | 20 | 1 | 1 | 0 | 0 | 0 | 1 | 56 | 160 |
| 80 | 890 | 0 | 1231 | 0 | 0 | 178 | 30.2 | 0 | 0 | 0 | 0 | 0 | 0.200 | 1 | 21 | 1 | 1 | 0 | 0 | 0 | 1 | 28 | 106.3 |
| 81 | 890 | 0 | 1231 | 0 | 0 | 178 | 30.2 | 0 | 0 | 0 | 0 | 0 | 0.200 | 1 | 21 | 1 | 1 | 0 | 0 | 0 | 1 | 28 | 113.2 |
| 82 | 845.5 | 44.5 | 1231 | 0 | 0 | 178 | 30.2 | 0 | 0 | 0 | 0 | 0 | 0.200 | 1 | 21 | 1 | 1 | 0 | 0 | 0 | 1 | 28 | 117.2 |
| 83 | 801 | 89 | 1231 | 0 | 0 | 178 | 30.2 | 0 | 0 | 0 | 0 | 0 | 0.200 | 1 | 21 | 1 | 1 | 0 | 0 | 0 | 1 | 28 | 118.6 |
| 84 | 756.5 | 133.5 | 1231 | 0 | 0 | 178 | 30.2 | 0 | 0 | 0 | 0 | 0 | 0.200 | 1 | 21 | 1 | 1 | 0 | 0 | 0 | 1 | 28 | 118 |
| 85 | 712 | 178 | 1231 | 0 | 0 | 178 | 30.2 | 0 | 0 | 0 | 0 | 0 | 0.200 | 1 | 21 | 1 | 1 | 0 | 0 | 0 | 1 | 28 | 111 |
| 86 | 845.5 | 44.5 | 1231 | 0 | 0 | 178 | 30.2 | 0 | 0 | 0 | 0 | 0 | 0.200 | 1 | 21 | 1 | 1 | 0 | 0 | 0 | 1 | 28 | 116.6 |
| 87 | 712 | 0 | 1231 | 0 | 0 | 178 | 30.2 | 0 | 178 | 0 | 0 | 0 | 0.250 | 1 | 21 | 1 | 1 | 0 | 0 | 0 | 1 | 28 | 109.9 |
| 88 | 623 | 0 | 1231 | 0 | 0 | 178 | 30.2 | 0 | 267 | 0 | 0 | 0 | 0.286 | 1 | 21 | 1 | 1 | 0 | 0 | 0 | 1 | 28 | 114.8 |
| 89 | 534 | 0 | 1231 | 0 | 0 | 178 | 30.2 | 0 | 356 | 0 | 0 | 0 | 0.333 | 1 | 21 | 1 | 1 | 0 | 0 | 0 | 1 | 28 | 102.6 |
| 90 | 630 | 90 | 1539 | 0 | 0 | 162 | 18 | 0 | 0 | 180 | 0 | 0 | 0.225 | 1 | 20 | 1 | 1 | 0 | 0 | 0 | 1 | 56 | 141 |
| 91 | 667.5 | 44.5 | 1231 | 0 | 0 | 178 | 30.2 | 0 | 178 | 0 | 0 | 0 | 0.250 | 1 | 21 | 1 | 1 | 0 | 0 | 0 | 1 | 28 | 112.8 |
| 92 | 1009 | 0 | 1231 | 0 | 0 | 201.8 | 34.2 | 0 | 0 | 0 | 0 | 0 | 0.200 | 1 | 21 | 1 | 1 | 0 | 0 | 0 | 1 | 28 | 113.8 |
| 93 | 1009 | 0 | 1231 | 0 | 0 | 201.8 | 34.2 | 0 | 0 | 0 | 0 | 0 | 0.200 | 1 | 21 | 1 | 1 | 0 | 0 | 0 | 1 | 28 | 115.2 |
| 94 | 958.55 | 50.45 | 1231 | 0 | 0 | 201.8 | 34.2 | 0 | 0 | 0 | 0 | 0 | 0.200 | 1 | 21 | 1 | 1 | 0 | 0 | 0 | 1 | 28 | 115.4 |
| 95 | 756.75 | 50.45 | 1231 | 0 | 0 | 201.8 | 34.2 | 0 | 201.8 | 0 | 0 | 0 | 0.250 | 1 | 21 | 1 | 1 | 0 | 0 | 0 | 1 | 28 | 114.5 |
| 96 | 1151.11 | 211.89 | 1231 | 0 | 0 | 272.6 | 46.2 | 0 | 0 | 0 | 0 | 0 | 0.200 | 1 | 90 | 1 | 1 | 0 | 0 | 0 | 1 | 28 | 159.7 |
| 97 | 1151.11 | 211.89 | 1231 | 0 | 0 | 272.6 | 46.2 | 0 | 0 | 0 | 0 | 0 | 0.200 | 1 | 90 | 1 | 1 | 0 | 0 | 0 | 1 | 28 | 165.8 |
| 98 | 1151.11 | 211.89 | 1231 | 0 | 0 | 272.6 | 46.2 | 0 | 0 | 0 | 0 | 0 | 0.200 | 1 | 90 | 1 | 1 | 0 | 0 | 0 | 1 | 28 | 163.9 |
| 99 | 1151.11 | 211.89 | 1231 | 0 | 0 | 272.6 | 46.2 | 234 | 0 | 0 | 0 | 0 | 0.200 | 1 | 90 | 1 | 1 | 1 | 13 | 0.2 | 1 | 28 | 170.6 |
| 100 | 1151.11 | 211.89 | 1231 | 0 | 0 | 272.6 | 46.2 | 234 | 0 | 0 | 0 | 0 | 0.200 | 1 | 90 | 1 | 1 | 1 | 13 | 0.2 | 1 | 28 | 179 |
| 101 | 630 | 90 | 1539 | 0 | 0 | 144 | 18 | 0 | 0 | 180 | 0 | 0 | 0.200 | 1 | 20 | 1 | 1 | 0 | 0 | 0 | 1 | 56 | 160 |
| 102 | 1151.11 | 211.89 | 1231 | 0 | 0 | 272.6 | 46.2 | 234 | 0 | 0 | 0 | 0 | 0.200 | 1 | 90 | 1 | 1 | 1 | 13 | 0.2 | 1 | 28 | 177 |
| 103 | 890 | 0 | 1231 | 0 | 0 | 178 | 30.2 | 0 | 0 | 0 | 0 | 0 | 0.200 | 1 | 21 | 1 | 1 | 0 | 0 | 0 | 1 | 56 | 108.8 |
| 104 | 890 | 0 | 1231 | 0 | 0 | 178 | 30.2 | 0 | 0 | 0 | 0 | 0 | 0.200 | 1 | 21 | 1 | 1 | 0 | 0 | 0 | 1 | 56 | 113.8 |
| 105 | 845.5 | 44.5 | 1231 | 0 | 0 | 178 | 30.2 | 0 | 0 | 0 | 0 | 0 | 0.200 | 1 | 21 | 1 | 1 | 0 | 0 | 0 | 1 | 56 | 120.1 |
| 106 | 801 | 89 | 1231 | 0 | 0 | 178 | 30.2 | 0 | 0 | 0 | 0 | 0 | 0.200 | 1 | 21 | 1 | 1 | 0 | 0 | 0 | 1 | 56 | 127.4 |
| 107 | 756.5 | 133.5 | 1231 | 0 | 0 | 178 | 30.2 | 0 | 0 | 0 | 0 | 0 | 0.200 | 1 | 21 | 1 | 1 | 0 | 0 | 0 | 1 | 56 | 120.1 |
| 108 | 712 | 178 | 1231 | 0 | 0 | 178 | 30.2 | 0 | 0 | 0 | 0 | 0 | 0.200 | 1 | 21 | 1 | 1 | 0 | 0 | 0 | 1 | 56 | 117.2 |
| 109 | 845.5 | 44.5 | 1231 | 0 | 0 | 178 | 30.2 | 0 | 0 | 0 | 0 | 0 | 0.200 | 1 | 21 | 1 | 1 | 0 | 0 | 0 | 1 | 56 | 124.1 |
| 110 | 712 | 0 | 1231 | 0 | 0 | 178 | 30.2 | 0 | 178 | 0 | 0 | 0 | 0.250 | 1 | 21 | 1 | 1 | 0 | 0 | 0 | 1 | 56 | 110.3 |
| 111 | 623 | 0 | 1231 | 0 | 0 | 178 | 30.2 | 0 | 267 | 0 | 0 | 0 | 0.286 | 1 | 21 | 1 | 1 | 0 | 0 | 0 | 1 | 56 | 117.2 |
| 112 | 630 | 70 | 1788 | 0 | 0 | 126 | 18 | 0 | 0 | 0 | 0 | 0 | 0.180 | 1 | 20 | 1 | 1 | 0 | 0 | 0 | 1 | 28 | 135 |
| 113 | 450 | 90 | 1539 | 0 | 0 | 144 | 18 | 0 | 0 | 180 | 0 | 180 | 0.267 | 1 | 20 | 1 | 1 | 0 | 0 | 0 | 1 | 56 | 161 |
| 114 | 534 | 0 | 1231 | 0 | 0 | 178 | 30.2 | 0 | 356 | 0 | 0 | 0 | 0.333 | 1 | 21 | 1 | 1 | 0 | 0 | 0 | 1 | 56 | 110.9 |
| 115 | 667.5 | 44.5 | 1231 | 0 | 0 | 178 | 30.2 | 0 | 178 | 0 | 0 | 0 | 0.250 | 1 | 21 | 1 | 1 | 0 | 0 | 0 | 1 | 56 | 113.8 |
| 116 | 1009 | 0 | 1231 | 0 | 0 | 201.8 | 34.2 | 0 | 0 | 0 | 0 | 0 | 0.200 | 1 | 21 | 1 | 1 | 0 | 0 | 0 | 1 | 56 | 126.2 |
| 117 | 1009 | 0 | 1231 | 0 | 0 | 201.8 | 34.2 | 0 | 0 | 0 | 0 | 0 | 0.200 | 1 | 21 | 1 | 1 | 0 | 0 | 0 | 1 | 56 | 129.3 |
| 118 | 958.55 | 50.45 | 1231 | 0 | 0 | 201.8 | 34.2 | 0 | 0 | 0 | 0 | 0 | 0.200 | 1 | 21 | 1 | 1 | 0 | 0 | 0 | 1 | 56 | 129 |
| 119 | 756.75 | 50.45 | 1231 | 0 | 0 | 201.8 | 34.2 | 0 | 201.8 | 0 | 0 | 0 | 0.250 | 1 | 21 | 1 | 1 | 0 | 0 | 0 | 1 | 56 | 131.7 |
| 120 | 890 | 0 | 1231 | 0 | 0 | 178 | 30.2 | 0 | 0 | 0 | 0 | 0 | 0.200 | 1 | 21 | 1 | 1 | 0 | 0 | 0 | 1 | 90 | 114.1 |
| 121 | 890 | 0 | 1231 | 0 | 0 | 178 | 30.2 | 0 | 0 | 0 | 0 | 0 | 0.200 | 1 | 21 | 1 | 1 | 0 | 0 | 0 | 1 | 90 | 118.1 |
| 122 | 845.5 | 44.5 | 1231 | 0 | 0 | 178 | 30.2 | 0 | 0 | 0 | 0 | 0 | 0.200 | 1 | 21 | 1 | 1 | 0 | 0 | 0 | 1 | 90 | 125.4 |
| 123 | 801 | 89 | 1231 | 0 | 0 | 178 | 30.2 | 0 | 0 | 0 | 0 | 0 | 0.200 | 1 | 21 | 1 | 1 | 0 | 0 | 0 | 1 | 90 | 127.6 |
| 124 | 270 | 90 | 1539 | 0 | 0 | 144 | 18 | 0 | 0 | 180 | 0 | 360 | 0.400 | 1 | 20 | 1 | 1 | 0 | 0 | 0 | 1 | 56 | 139 |
| 125 | 756.5 | 133.5 | 1231 | 0 | 0 | 178 | 30.2 | 0 | 0 | 0 | 0 | 0 | 0.200 | 1 | 21 | 1 | 1 | 0 | 0 | 0 | 1 | 90 | 120.9 |
| 126 | 712 | 178 | 1231 | 0 | 0 | 178 | 30.2 | 0 | 0 | 0 | 0 | 0 | 0.200 | 1 | 21 | 1 | 1 | 0 | 0 | 0 | 1 | 90 | 118.6 |
| 127 | 845.5 | 44.5 | 1231 | 0 | 0 | 178 | 30.2 | 0 | 0 | 0 | 0 | 0 | 0.200 | 1 | 21 | 1 | 1 | 0 | 0 | 0 | 1 | 90 | 126.2 |
| 128 | 712 | 0 | 1231 | 0 | 0 | 178 | 30.2 | 0 | 178 | 0 | 0 | 0 | 0.250 | 1 | 21 | 1 | 1 | 0 | 0 | 0 | 1 | 90 | 117.5 |
| 129 | 623 | 0 | 1231 | 0 | 0 | 178 | 30.2 | 0 | 267 | 0 | 0 | 0 | 0.286 | 1 | 21 | 1 | 1 | 0 | 0 | 0 | 1 | 90 | 119.3 |
| 130 | 534 | 0 | 1231 | 0 | 0 | 178 | 30.2 | 0 | 356 | 0 | 0 | 0 | 0.333 | 1 | 21 | 1 | 1 | 0 | 0 | 0 | 1 | 90 | 119.1 |
| 131 | 667.5 | 44.5 | 1231 | 0 | 0 | 178 | 30.2 | 0 | 178 | 0 | 0 | 0 | 0.250 | 1 | 21 | 1 | 1 | 0 | 0 | 0 | 1 | 90 | 119.7 |
| 132 | 1009 | 0 | 1231 | 0 | 0 | 201.8 | 34.2 | 0 | 0 | 0 | 0 | 0 | 0.200 | 1 | 21 | 1 | 1 | 0 | 0 | 0 | 1 | 90 | 139.3 |
| 133 | 1009 | 0 | 1231 | 0 | 0 | 201.8 | 34.2 | 0 | 0 | 0 | 0 | 0 | 0.200 | 1 | 21 | 1 | 1 | 0 | 0 | 0 | 1 | 90 | 149.7 |
| 134 | 958.55 | 50.45 | 1231 | 0 | 0 | 201.8 | 34.2 | 0 | 0 | 0 | 0 | 0 | 0.200 | 1 | 21 | 1 | 1 | 0 | 0 | 0 | 1 | 90 | 155.2 |
| 135 | 450 | 50 | 1992 | 0 | 0 | 90 | 18 | 0 | 0 | 0 | 0 | 0 | 0.180 | 1 | 20 | 1 | 1 | 0 | 0 | 0 | 1 | 90 | 151 |
| 136 | 756.75 | 50.45 | 1231 | 0 | 0 | 201.8 | 34.2 | 0 | 201.8 | 0 | 0 | 0 | 0.250 | 1 | 21 | 1 | 1 | 0 | 0 | 0 | 1 | 90 | 152.1 |
| 137 | 472 | 262 | 1049 | 0 | 0 | 178 | 21 | 0 | 0 | 315 | 0 | 0 | 0.243 | 1 | 20 | 1 | 1 | 0 | 0 | 0 | 1 | 7 | 94.3 |
| 138 | 472 | 262 | 1049 | 0 | 0 | 178 | 21 | 156 | 0 | 315 | 0 | 0 | 0.243 | 1 | 20 | 1 | 1 | 1 | 13 | 0.2 | 1 | 7 | 114.9 |
| 139 | 472 | 262 | 1049 | 0 | 0 | 178 | 21 | 156 | 0 | 315 | 0 | 0 | 0.243 | 1 | 20 | 1 | 1 | 1 | 13 | 0.2 | 1 | 7 | 120.9 |
| 140 | 472 | 262 | 1049 | 0 | 0 | 178 | 21 | 156 | 0 | 315 | 0 | 0 | 0.243 | 1 | 20 | 1 | 1 | 1 | 13 | 0.2 | 1 | 7 | 108.3 |
| 141 | 472 | 262 | 1049 | 0 | 0 | 178 | 21 | 0 | 0 | 315 | 0 | 0 | 0.243 | 1 | 20 | 1 | 1 | 0 | 0 | 0 | 1 | 28 | 97.8 |
| 142 | 472 | 262 | 1049 | 0 | 0 | 178 | 21 | 156 | 0 | 315 | 0 | 0 | 0.243 | 1 | 20 | 1 | 1 | 1 | 13 | 0.2 | 1 | 28 | 134.4 |
| 143 | 472 | 262 | 1049 | 0 | 0 | 178 | 21 | 156 | 0 | 315 | 0 | 0 | 0.243 | 1 | 20 | 1 | 1 | 1 | 13 | 0.2 | 1 | 28 | 143.5 |
| 144 | 472 | 262 | 1049 | 0 | 0 | 178 | 21 | 156 | 0 | 315 | 0 | 0 | 0.243 | 1 | 20 | 1 | 1 | 1 | 13 | 0.2 | 1 | 28 | 127.4 |
| 145 | 472 | 262 | 1049 | 0 | 0 | 178 | 21 | 156 | 0 | 315 | 0 | 0 | 0.243 | 1 | 20 | 1 | 1 | 1 | 13 | 0.2 | 1 | 28 | 122.9 |
| 146 | 630 | 70 | 1788 | 0 | 0 | 126 | 18 | 0 | 0 | 0 | 0 | 0 | 0.180 | 1 | 20 | 1 | 1 | 0 | 0 | 0 | 1 | 90 | 160 |
| 147 | 472 | 262 | 1049 | 0 | 0 | 178 | 21 | 156 | 0 | 315 | 0 | 0 | 0.243 | 1 | 20 | 1 | 1 | 1 | 13 | 0.2 | 1 | 28 | 120.4 |
| 148 | 850 | 137.5 | 1100 | 0 | 0 | 176 | 8 | 234 | 112.5 | 0 | 0 | 0 | 0.178 | 1 | 23 | 1 | 1 | 1 | 13 | 0.2 | 1 | 28 | 162.1 |
| 149 | 850 | 137.5 | 1100 | 0 | 0 | 176 | 8 | 234 | 112.5 | 0 | 0 | 0 | 0.178 | 1 | 23 | 1 | 1 | 1 | 13 | 0.2 | 1 | 28 | 162.8 |
| 150 | 700 | 50 | 1104 | 0 | 0 | 180 | 30 | 0 | 150 | 0 | 0 | 0 | 0.240 | 1 | 20 | 1 | 1 | 0 | 0 | 0 | 1 | 1 | 57.9 |
| 151 | 700 | 50 | 1104 | 0 | 0 | 180 | 30 | 39 | 150 | 0 | 0 | 0 | 0.240 | 1 | 20 | 1 | 1 | 1 | 13 | 0.2 | 1 | 1 | 64.5 |
| 152 | 700 | 50 | 1104 | 0 | 0 | 180 | 30 | 78 | 150 | 0 | 0 | 0 | 0.240 | 1 | 20 | 1 | 1 | 1 | 13 | 0.2 | 1 | 1 | 73.8 |
| 153 | 700 | 50 | 1104 | 0 | 0 | 180 | 30 | 117 | 150 | 0 | 0 | 0 | 0.240 | 1 | 20 | 1 | 1 | 1 | 13 | 0.2 | 1 | 1 | 83.2 |
| 154 | 700 | 50 | 1104 | 0 | 0 | 180 | 30 | 156 | 150 | 0 | 0 | 0 | 0.240 | 1 | 20 | 1 | 1 | 1 | 13 | 0.2 | 1 | 1 | 88.8 |
| 155 | 700 | 50 | 1104 | 0 | 0 | 180 | 30 | 195 | 150 | 0 | 0 | 0 | 0.240 | 1 | 20 | 1 | 1 | 1 | 13 | 0.2 | 1 | 1 | 89.7 |
| 156 | 700 | 50 | 1104 | 0 | 0 | 180 | 30 | 0 | 150 | 0 | 0 | 0 | 0.240 | 1 | 20 | 1 | 1 | 0 | 0 | 0 | 1 | 7 | 94.4 |
| 157 | 810 | 90 | 1539 | 0 | 0 | 162 | 18 | 0 | 0 | 0 | 0 | 0 | 0.180 | 1 | 20 | 1 | 1 | 0 | 0 | 0 | 1 | 90 | 170 |
| 158 | 700 | 50 | 1104 | 0 | 0 | 180 | 30 | 39 | 150 | 0 | 0 | 0 | 0.240 | 1 | 20 | 1 | 1 | 1 | 13 | 0.2 | 1 | 7 | 96.3 |
| 159 | 700 | 50 | 1104 | 0 | 0 | 180 | 30 | 78 | 150 | 0 | 0 | 0 | 0.240 | 1 | 20 | 1 | 1 | 1 | 13 | 0.2 | 1 | 7 | 102.8 |
| 160 | 700 | 50 | 1104 | 0 | 0 | 180 | 30 | 117 | 150 | 0 | 0 | 0 | 0.240 | 1 | 20 | 1 | 1 | 1 | 13 | 0.2 | 1 | 7 | 115.9 |
| 161 | 700 | 50 | 1104 | 0 | 0 | 180 | 30 | 156 | 150 | 0 | 0 | 0 | 0.240 | 1 | 20 | 1 | 1 | 1 | 13 | 0.2 | 1 | 7 | 120.6 |
| 162 | 700 | 50 | 1104 | 0 | 0 | 180 | 30 | 195 | 150 | 0 | 0 | 0 | 0.240 | 1 | 20 | 1 | 1 | 1 | 13 | 0.2 | 1 | 7 | 125.2 |
| 163 | 700 | 50 | 1104 | 0 | 0 | 180 | 30 | 0 | 150 | 0 | 0 | 0 | 0.240 | 1 | 20 | 1 | 1 | 0 | 0 | 0 | 1 | 28 | 105.6 |
| 164 | 700 | 50 | 1104 | 0 | 0 | 180 | 30 | 78 | 150 | 0 | 0 | 0 | 0.240 | 1 | 20 | 1 | 1 | 1 | 13 | 0.2 | 1 | 28 | 138.3 |
| 165 | 700 | 50 | 1104 | 0 | 0 | 180 | 30 | 117 | 150 | 0 | 0 | 0 | 0.240 | 1 | 20 | 1 | 1 | 1 | 13 | 0.2 | 1 | 28 | 149.5 |
| 166 | 700 | 50 | 1104 | 0 | 0 | 180 | 30 | 156 | 150 | 0 | 0 | 0 | 0.240 | 1 | 20 | 1 | 1 | 1 | 13 | 0.2 | 1 | 28 | 153.3 |
| 167 | 700 | 50 | 1104 | 0 | 0 | 180 | 30 | 195 | 150 | 0 | 0 | 0 | 0.240 | 1 | 20 | 1 | 1 | 1 | 13 | 0.2 | 1 | 28 | 156.1 |
| 168 | 810 | 90 | 1539 | 0 | 0 | 162 | 18 | 0 | 0 | 0 | 0 | 0 | 0.180 | 1 | 20 | 1 | 1 | 0 | 0 | 0 | 1 | 90 | 170 |
| 169 | 741 | 185 | 815 | 0 | 194.25 | 185 | 9 | 156 | 0 | 0 | 0 | 64.75 | 0.200 | 1 | 30 | 1 | 1 | 1 | 13 | 0.2 | 1 | 1 | 76 |
| 170 | 741 | 185 | 815 | 0 | 129.5 | 185 | 9 | 156 | 0 | 0 | 0 | 129.5 | 0.200 | 1 | 30 | 1 | 1 | 1 | 13 | 0.2 | 1 | 1 | 78 |
| 171 | 741 | 185 | 815 | 0 | 64.75 | 185 | 9 | 156 | 0 | 0 | 0 | 194.25 | 0.200 | 1 | 30 | 1 | 1 | 1 | 13 | 0.2 | 1 | 1 | 82 |
| 172 | 741 | 185 | 815 | 0 | 0 | 185 | 9 | 0 | 0 | 0 | 0 | 259 | 0.200 | 1 | 30 | 1 | 1 | 0 | 0 | 0 | 1 | 1 | 82 |
| 173 | 741 | 185 | 815 | 0 | 259 | 185 | 9 | 156 | 0 | 0 | 0 | 0 | 0.200 | 1 | 30 | 1 | 1 | 1 | 13 | 0.2 | 1 | 3 | 117 |
| 174 | 741 | 185 | 815 | 0 | 194.25 | 185 | 9 | 156 | 0 | 0 | 0 | 64.75 | 0.200 | 1 | 30 | 1 | 1 | 1 | 13 | 0.2 | 1 | 3 | 118 |
| 175 | 741 | 185 | 815 | 0 | 129.5 | 185 | 9 | 156 | 0 | 0 | 0 | 129.5 | 0.200 | 1 | 30 | 1 | 1 | 1 | 13 | 0.2 | 1 | 3 | 111 |
| 176 | 741 | 185 | 815 | 0 | 64.75 | 185 | 9 | 156 | 0 | 0 | 0 | 194.25 | 0.200 | 1 | 30 | 1 | 1 | 1 | 13 | 0.2 | 1 | 3 | 116 |
| 177 | 741 | 185 | 815 | 0 | 0 | 185 | 9 | 0 | 0 | 0 | 0 | 259 | 0.200 | 1 | 30 | 1 | 1 | 0 | 0 | 0 | 1 | 3 | 115 |
| 178 | 741 | 185 | 815 | 0 | 259 | 185 | 9 | 156 | 0 | 0 | 0 | 0 | 0.200 | 1 | 30 | 1 | 1 | 1 | 13 | 0.2 | 1 | 7 | 144 |
| 179 | 630 | 90 | 1539 | 0 | 0 | 162 | 18 | 0 | 0 | 180 | 0 | 0 | 0.225 | 1 | 20 | 1 | 1 | 0 | 0 | 0 | 1 | 90 | 169 |
| 180 | 741 | 185 | 815 | 0 | 194.25 | 185 | 9 | 156 | 0 | 0 | 0 | 64.75 | 0.200 | 1 | 30 | 1 | 1 | 1 | 13 | 0.2 | 1 | 7 | 142 |
| 181 | 741 | 185 | 815 | 0 | 64.75 | 185 | 9 | 156 | 0 | 0 | 0 | 194.25 | 0.200 | 1 | 30 | 1 | 1 | 1 | 13 | 0.2 | 1 | 7 | 138 |
| 182 | 741 | 185 | 815 | 0 | 194.25 | 185 | 9 | 156 | 0 | 0 | 0 | 64.75 | 0.200 | 1 | 30 | 1 | 1 | 1 | 13 | 0.2 | 1 | 28 | 159 |
| 183 | 741 | 185 | 815 | 0 | 129.5 | 185 | 9 | 156 | 0 | 0 | 0 | 129.5 | 0.200 | 1 | 30 | 1 | 1 | 1 | 13 | 0.2 | 1 | 28 | 160 |
| 184 | 741 | 185 | 815 | 0 | 64.75 | 185 | 9 | 156 | 0 | 0 | 0 | 194.25 | 0.200 | 1 | 30 | 1 | 1 | 1 | 13 | 0.2 | 1 | 28 | 158 |
| 185 | 741 | 185 | 815 | 0 | 0 | 185 | 9 | 0 | 0 | 0 | 0 | 259 | 0.200 | 1 | 30 | 1 | 1 | 0 | 0 | 0 | 1 | 28 | 155 |
| 186 | 741 | 185 | 815 | 0 | 259 | 185 | 9 | 156 | 0 | 0 | 0 | 0 | 0.200 | 1 | 30 | 1 | 1 | 1 | 13 | 0.2 | 1 | 90 | 165 |
| 187 | 741 | 185 | 815 | 0 | 194.25 | 185 | 9 | 156 | 0 | 0 | 0 | 64.75 | 0.200 | 1 | 30 | 1 | 1 | 1 | 13 | 0.2 | 1 | 90 | 171 |
| 188 | 741 | 185 | 815 | 0 | 129.5 | 185 | 9 | 156 | 0 | 0 | 0 | 129.5 | 0.200 | 1 | 30 | 1 | 1 | 1 | 13 | 0.2 | 1 | 90 | 167 |
| 189 | 741 | 185 | 815 | 0 | 64.75 | 185 | 9 | 156 | 0 | 0 | 0 | 194.25 | 0.200 | 1 | 30 | 1 | 1 | 1 | 13 | 0.2 | 1 | 90 | 168 |
| 190 | 630 | 90 | 1539 | 0 | 0 | 126 | 18 | 0 | 0 | 180 | 0 | 0 | 0.175 | 1 | 20 | 1 | 1 | 0 | 0 | 0 | 1 | 90 | 168 |
| 191 | 890 | 222 | 799 | 0 | 0 | 222 | 29.7 | 117 | 0 | 0 | 0 | 0 | 0.200 | 1 | 23 | 1 | 1 | 1 | 13 | 0.2 | 1 | 28 | 130.3 |
| 192 | 933 | 233 | 1026 | 0 | 0 | 168 | 37.6 | 0 | 0 | 0 | 0 | 0 | 0.144 | 1 | 23 | 1 | 1 | 0 | 0 | 0 | 1 | 28 | 101.4 |
| 193 | 933 | 233 | 1026 | 0 | 0 | 168 | 37.6 | 0 | 0 | 0 | 0 | 0 | 0.144 | 1 | 23 | 1 | 1 | 0 | 0 | 0 | 1 | 28 | 116.2 |
| 194 | 850 | 260 | 850 | 0 | 212 | 170 | 45 | 156 | 0 | 0 | 0 | 0 | 0.153 | 1 | 23 | 1 | 1 | 1 | 13 | 0.2 | 1 | 28 | 170.4 |
| 195 | 850 | 260 | 850 | 0 | 212 | 170 | 45 | 156 | 0 | 0 | 0 | 0 | 0.153 | 1 | 23 | 1 | 1 | 1 | 13 | 0.2 | 1 | 28 | 184 |
| 196 | 950 | 234 | 1030 | 0 | 95 | 215 | 12.7 | 234 | 0 | 0 | 0 | 0 | 0.182 | 1 | 23 | 1 | 1 | 1 | 13 | 0.2 | 1 | 28 | 158.1 |
| 197 | 950 | 234 | 1030 | 0 | 95 | 215 | 12.7 | 234 | 0 | 0 | 0 | 0 | 0.182 | 1 | 23 | 1 | 1 | 1 | 13 | 0.2 | 1 | 28 | 168.6 |
| 198 | 850 | 260 | 850 | 0 | 212 | 170 | 45 | 156 | 0 | 0 | 0 | 0 | 0.153 | 1 | 23 | 1 | 1 | 1 | 13 | 0.2 | 1 | 28 | 180.8 |
| 199 | 850 | 260 | 850 | 0 | 212 | 170 | 45 | 156 | 0 | 0 | 0 | 0 | 0.153 | 1 | 23 | 1 | 1 | 1 | 13 | 0.2 | 1 | 28 | 192.2 |
| 200 | 850 | 260 | 850 | 0 | 212 | 170 | 45 | 156 | 0 | 0 | 0 | 0 | 0.153 | 1 | 23 | 1 | 1 | 1 | 13 | 0.2 | 1 | 28 | 206.6 |
| 201 | 630 | 90 | 1539 | 0 | 0 | 144 | 18 | 0 | 0 | 180 | 0 | 0 | 0.200 | 1 | 20 | 1 | 1 | 0 | 0 | 0 | 1 | 90 | 172 |
| 202 | 850 | 260 | 850 | 0 | 212 | 170 | 45 | 156 | 0 | 0 | 0 | 0 | 0.153 | 1 | 23 | 1 | 1 | 1 | 13 | 0.2 | 1 | 28 | 185.2 |
| 203 | 850 | 260 | 850 | 0 | 212 | 170 | 45 | 156 | 0 | 0 | 0 | 0 | 0.153 | 1 | 23 | 1 | 1 | 1 | 13 | 0.2 | 1 | 28 | 198.5 |
| 204 | 850 | 260 | 850 | 0 | 212 | 170 | 45 | 156 | 0 | 0 | 0 | 0 | 0.153 | 1 | 23 | 1 | 1 | 1 | 13 | 0.2 | 1 | 28 | 188.2 |
| 205 | 850 | 260 | 850 | 0 | 212 | 170 | 45 | 156 | 0 | 0 | 0 | 0 | 0.153 | 1 | 23 | 1 | 1 | 1 | 13 | 0.2 | 1 | 28 | 202.2 |
| 206 | 850 | 260 | 850 | 0 | 212 | 170 | 45 | 156 | 0 | 0 | 0 | 0 | 0.153 | 1 | 23 | 1 | 1 | 1 | 13 | 0.2 | 1 | 28 | 193.5 |
| 207 | 850 | 260 | 850 | 0 | 212 | 170 | 45 | 156 | 0 | 0 | 0 | 0 | 0.153 | 1 | 23 | 1 | 1 | 1 | 13 | 0.2 | 1 | 28 | 203.9 |
| 208 | 850 | 260 | 850 | 0 | 212 | 170 | 45 | 156 | 0 | 0 | 0 | 0 | 0.153 | 1 | 23 | 1 | 1 | 1 | 13 | 0.2 | 1 | 28 | 197.9 |
| 209 | 850 | 260 | 850 | 0 | 212 | 170 | 45 | 156 | 0 | 0 | 0 | 0 | 0.153 | 1 | 23 | 1 | 1 | 1 | 13 | 0.2 | 1 | 28 | 201.2 |
| 210 | 850 | 260 | 850 | 0 | 212 | 170 | 45 | 156 | 0 | 0 | 0 | 0 | 0.153 | 1 | 23 | 1 | 1 | 1 | 13 | 0.2 | 1 | 28 | 206.2 |
| 211 | 850 | 260 | 850 | 0 | 212 | 170 | 45 | 156 | 0 | 0 | 0 | 0 | 0.153 | 1 | 23 | 1 | 1 | 1 | 13 | 0.2 | 1 | 28 | 205.6 |
| 212 | 630 | 90 | 1539 | 0 | 0 | 162 | 18 | 0 | 0 | 180 | 0 | 0 | 0.225 | 1 | 20 | 1 | 1 | 0 | 0 | 0 | 1 | 90 | 169 |
| 213 | 850 | 260 | 850 | 0 | 212 | 170 | 45 | 156 | 0 | 0 | 0 | 0 | 0.153 | 1 | 23 | 1 | 1 | 1 | 13 | 0.2 | 1 | 28 | 199.9 |
| 214 | 850 | 260 | 850 | 0 | 212 | 170 | 45 | 156 | 0 | 0 | 0 | 0 | 0.153 | 1 | 23 | 1 | 1 | 1 | 13 | 0.2 | 1 | 28 | 195.2 |
| 215 | 850 | 260 | 850 | 0 | 212 | 170 | 45 | 156 | 0 | 0 | 0 | 0 | 0.153 | 1 | 23 | 1 | 1 | 1 | 13 | 0.2 | 1 | 28 | 190.2 |
| 216 | 850 | 260 | 850 | 0 | 212 | 170 | 45 | 156 | 0 | 0 | 0 | 0 | 0.153 | 1 | 23 | 1 | 1 | 1 | 13 | 0.2 | 1 | 28 | 208.2 |
| 217 | 850 | 260 | 850 | 0 | 212 | 170 | 45 | 156 | 0 | 0 | 0 | 0 | 0.153 | 1 | 23 | 1 | 1 | 1 | 13 | 0.2 | 1 | 28 | 181 |
| 218 | 850 | 260 | 850 | 0 | 212 | 170 | 45 | 156 | 0 | 0 | 0 | 0 | 0.153 | 1 | 23 | 1 | 1 | 1 | 13 | 0.2 | 1 | 28 | 192.2 |
| 219 | 850 | 260 | 850 | 0 | 212 | 170 | 45 | 156 | 0 | 0 | 0 | 0 | 0.153 | 1 | 23 | 1 | 1 | 1 | 13 | 0.2 | 1 | 28 | 206.78 |
| 220 | 850 | 260 | 850 | 0 | 212 | 170 | 45 | 156 | 0 | 0 | 0 | 0 | 0.153 | 1 | 23 | 1 | 1 | 1 | 13 | 0.2 | 1 | 28 | 182.4 |
| 221 | 850 | 260 | 850 | 0 | 212 | 170 | 45 | 156 | 0 | 0 | 0 | 0 | 0.153 | 1 | 23 | 1 | 1 | 1 | 13 | 0.2 | 1 | 28 | 194.9 |
| 222 | 850 | 260 | 850 | 0 | 212 | 170 | 45 | 156 | 0 | 0 | 0 | 0 | 0.153 | 1 | 23 | 1 | 1 | 1 | 13 | 0.2 | 1 | 28 | 207.8 |
| 223 | 810 | 90 | 1539 | 0 | 0 | 162 | 18 | 0 | 0 | 0 | 0 | 0 | 0.180 | 1 | 20 | 1 | 1 | 0 | 0 | 0 | 1 | 28 | 137 |
| 224 | 630 | 90 | 1539 | 0 | 0 | 144 | 18 | 0 | 0 | 180 | 0 | 0 | 0.200 | 1 | 20 | 1 | 1 | 0 | 0 | 0 | 1 | 90 | 172 |
| 225 | 850 | 260 | 850 | 0 | 212 | 170 | 45 | 156 | 0 | 0 | 0 | 0 | 0.153 | 1 | 23 | 1 | 1 | 1 | 13 | 0.2 | 1 | 28 | 186.1 |
| 226 | 850 | 260 | 850 | 0 | 212 | 170 | 45 | 156 | 0 | 0 | 0 | 0 | 0.153 | 1 | 23 | 1 | 1 | 1 | 13 | 0.2 | 1 | 28 | 196.9 |
| 227 | 850 | 260 | 850 | 0 | 212 | 170 | 45 | 156 | 0 | 0 | 0 | 0 | 0.153 | 1 | 23 | 1 | 1 | 1 | 13 | 0.2 | 1 | 28 | 186.8 |
| 228 | 850 | 260 | 850 | 0 | 212 | 170 | 45 | 156 | 0 | 0 | 0 | 0 | 0.153 | 1 | 23 | 1 | 1 | 1 | 13 | 0.2 | 1 | 28 | 197.6 |
| 229 | 850 | 260 | 850 | 0 | 212 | 170 | 45 | 156 | 0 | 0 | 0 | 0 | 0.153 | 1 | 23 | 1 | 1 | 1 | 13 | 0.2 | 1 | 28 | 187.5 |
| 230 | 850 | 260 | 850 | 0 | 212 | 170 | 45 | 156 | 0 | 0 | 0 | 0 | 0.153 | 1 | 23 | 1 | 1 | 1 | 13 | 0.2 | 1 | 28 | 200 |
| 231 | 850 | 260 | 850 | 0 | 212 | 170 | 45 | 156 | 0 | 0 | 0 | 0 | 0.153 | 1 | 23 | 1 | 1 | 1 | 13 | 0.2 | 1 | 28 | 173.2 |
| 232 | 850 | 260 | 850 | 0 | 212 | 170 | 45 | 156 | 0 | 0 | 0 | 0 | 0.153 | 1 | 23 | 1 | 1 | 1 | 13 | 0.2 | 1 | 28 | 184.4 |
| 233 | 850 | 260 | 850 | 0 | 212 | 170 | 45 | 156 | 0 | 0 | 0 | 0 | 0.153 | 1 | 23 | 1 | 1 | 1 | 13 | 0.2 | 1 | 28 | 196.9 |
| 234 | 850 | 260 | 850 | 0 | 212 | 170 | 45 | 156 | 0 | 0 | 0 | 0 | 0.153 | 1 | 23 | 1 | 1 | 1 | 13 | 0.2 | 1 | 28 | 181 |
| 235 | 450 | 90 | 1539 | 0 | 0 | 144 | 18 | 0 | 0 | 180 | 0 | 180 | 0.267 | 1 | 20 | 1 | 1 | 0 | 0 | 0 | 1 | 90 | 176 |
| 236 | 850 | 260 | 850 | 0 | 212 | 170 | 45 | 156 | 0 | 0 | 0 | 0 | 0.153 | 1 | 23 | 1 | 1 | 1 | 13 | 0.2 | 1 | 28 | 191.2 |
| 237 | 850 | 260 | 850 | 0 | 212 | 170 | 45 | 156 | 0 | 0 | 0 | 0 | 0.153 | 1 | 23 | 1 | 1 | 1 | 13 | 0.2 | 1 | 28 | 176.6 |
| 238 | 850 | 260 | 850 | 0 | 212 | 170 | 45 | 156 | 0 | 0 | 0 | 0 | 0.153 | 1 | 23 | 1 | 1 | 1 | 13 | 0.2 | 1 | 28 | 182.4 |
| 239 | 850 | 260 | 850 | 0 | 212 | 170 | 45 | 156 | 0 | 0 | 0 | 0 | 0.153 | 1 | 23 | 1 | 1 | 1 | 13 | 0.2 | 1 | 28 | 172.9 |
| 240 | 850 | 260 | 850 | 0 | 212 | 170 | 45 | 156 | 0 | 0 | 0 | 0 | 0.153 | 1 | 23 | 1 | 1 | 1 | 13 | 0.2 | 1 | 28 | 181.7 |
| 241 | 850 | 260 | 850 | 0 | 212 | 170 | 45 | 156 | 0 | 0 | 0 | 0 | 0.153 | 1 | 23 | 1 | 1 | 1 | 13 | 0.2 | 1 | 28 | 171.2 |
| 242 | 850 | 260 | 850 | 0 | 212 | 170 | 45 | 156 | 0 | 0 | 0 | 0 | 0.153 | 1 | 23 | 1 | 1 | 1 | 13 | 0.2 | 1 | 28 | 180.3 |
| 243 | 850 | 260 | 850 | 0 | 212 | 170 | 45 | 156 | 0 | 0 | 0 | 0 | 0.153 | 1 | 23 | 1 | 1 | 1 | 13 | 0.2 | 1 | 28 | 174.6 |
| 244 | 788.5 | 197.1 | 1104 | 0 | 0 | 160.3 | 50 | 156 | 0 | 0 | 0 | 0 | 0.163 | 1 | 23 | 1 | 1 | 1 | 13 | 0.2 | 1 | 28 | 187.1 |
| 245 | 788.5 | 197.1 | 1104 | 0 | 0 | 160.3 | 50 | 156 | 0 | 0 | 0 | 0 | 0.163 | 1 | 23 | 1 | 1 | 1 | 13 | 0.2 | 1 | 28 | 192.3 |
| 246 | 270 | 90 | 1539 | 0 | 0 | 144 | 18 | 0 | 0 | 180 | 0 | 360 | 0.400 | 1 | 20 | 1 | 1 | 0 | 0 | 0 | 1 | 90 | 150 |
| 247 | 788.5 | 197.1 | 1104 | 0 | 0 | 160.3 | 50 | 156 | 0 | 0 | 0 | 0 | 0.163 | 1 | 23 | 1 | 1 | 1 | 13 | 0.2 | 1 | 28 | 204.4 |
| 248 | 833 | 208 | 1125 | 0 | 0 | 208 | 25 | 0 | 0 | 0 | 0 | 0 | 0.200 | 1 | 23 | 1 | 1 | 0 | 0 | 0 | 1 | 28 | 147 |
| 249 | 825 | 206 | 1114 | 0 | 0 | 206 | 25 | 78 | 0 | 0 | 0 | 0 | 0.200 | 1 | 23 | 1 | 1 | 1 | 13 | 0.2 | 1 | 28 | 145.5 |
| 250 | 817 | 204 | 1102 | 0 | 0 | 204 | 25 | 156 | 0 | 0 | 0 | 0 | 0.200 | 1 | 23 | 1 | 1 | 1 | 13 | 0.2 | 1 | 28 | 147.5 |
| 251 | 808 | 202 | 1091 | 0 | 0 | 202 | 24 | 234 | 0 | 0 | 0 | 0 | 0.200 | 1 | 23 | 1 | 1 | 1 | 13 | 0.2 | 1 | 28 | 165.7 |
| 252 | 800 | 200 | 880 | 0 | 200 | 200 | 9.6 | 0 | 0 | 0 | 0 | 0 | 0.200 | 1 | 20 | 1 | 1 | 0 | 0 | 0 | 1 | 7 | 101.5 |
| 253 | 800 | 100 | 980 | 0 | 200 | 200 | 9.6 | 0 | 0 | 0 | 0 | 0 | 0.222 | 1 | 20 | 1 | 1 | 0 | 0 | 0 | 1 | 7 | 111.4 |
| 254 | 800 | 200 | 980 | 0 | 100 | 200 | 9.6 | 0 | 0 | 0 | 0 | 0 | 0.200 | 1 | 20 | 1 | 1 | 0 | 0 | 0 | 1 | 7 | 108.6 |
| 255 | 800 | 200 | 1080 | 0 | 0 | 200 | 14.4 | 0 | 0 | 0 | 0 | 0 | 0.200 | 1 | 20 | 1 | 1 | 0 | 0 | 0 | 1 | 7 | 111 |
| 256 | 800 | 200 | 880 | 0 | 200 | 200 | 9.6 | 0 | 0 | 0 | 0 | 0 | 0.200 | 1 | 20 | 1 | 1 | 0 | 0 | 0 | 1 | 28 | 141.3 |
| 257 | 450 | 50 | 1992 | 0 | 0 | 90 | 18 | 0 | 0 | 0 | 0 | 0 | 0.180 | 1 | 20 | 1 | 1 | 0 | 0 | 0 | 1 | 180 | 159 |
| 258 | 800 | 100 | 980 | 0 | 200 | 200 | 9.6 | 0 | 0 | 0 | 0 | 0 | 0.222 | 1 | 20 | 1 | 1 | 0 | 0 | 0 | 1 | 28 | 147.3 |
| 259 | 800 | 200 | 980 | 0 | 100 | 200 | 9.6 | 0 | 0 | 0 | 0 | 0 | 0.200 | 1 | 20 | 1 | 1 | 0 | 0 | 0 | 1 | 28 | 146.5 |
| 260 | 800 | 200 | 1080 | 0 | 0 | 200 | 14.4 | 0 | 0 | 0 | 0 | 0 | 0.200 | 1 | 20 | 1 | 1 | 0 | 0 | 0 | 1 | 28 | 159.2 |
| 261 | 800 | 200 | 880 | 0 | 200 | 200 | 9.6 | 0 | 0 | 0 | 0 | 0 | 0.200 | 1 | 20 | 1 | 1 | 0 | 0 | 0 | 1 | 90 | 163.3 |
| 262 | 800 | 100 | 980 | 0 | 200 | 200 | 9.6 | 0 | 0 | 0 | 0 | 0 | 0.222 | 1 | 20 | 1 | 1 | 0 | 0 | 0 | 1 | 90 | 166.4 |
| 263 | 800 | 200 | 980 | 0 | 100 | 200 | 9.6 | 0 | 0 | 0 | 0 | 0 | 0.200 | 1 | 20 | 1 | 1 | 0 | 0 | 0 | 1 | 90 | 175.9 |
| 264 | 800 | 100 | 980 | 0 | 200 | 200 | 9.6 | 0 | 0 | 0 | 0 | 0 | 0.222 | 1 | 20 | 1 | 1 | 0 | 0 | 0 | 1 | 90 | 141.1 |
| 265 | 950 | 255 | 873 | 0 | 0 | 189 | 31 | 0 | 0 | 0 | 0 | 0 | 0.157 | 1 | 23 | 1 | 1 | 0 | 0 | 0 | 1 | 7 | 104.2 |
| 266 | 941.5 | 255 | 873 | 9.5 | 0 | 189 | 31 | 0 | 0 | 0 | 0 | 0 | 0.158 | 1 | 23 | 1 | 1 | 0 | 0 | 0 | 1 | 7 | 109.8 |
| 267 | 932 | 255 | 873 | 19 | 0 | 189 | 31 | 0 | 0 | 0 | 0 | 0 | 0.159 | 1 | 23 | 1 | 1 | 0 | 0 | 0 | 1 | 7 | 112.6 |
| 268 | 630 | 70 | 1788 | 0 | 0 | 126 | 18 | 0 | 0 | 0 | 0 | 0 | 0.180 | 1 | 20 | 1 | 1 | 0 | 0 | 0 | 1 | 180 | 167 |
| 269 | 921.5 | 255 | 873 | 28.5 | 0 | 189 | 31 | 0 | 0 | 0 | 0 | 0 | 0.161 | 1 | 23 | 1 | 1 | 0 | 0 | 0 | 1 | 7 | 119.4 |
| 270 | 912 | 255 | 873 | 38 | 0 | 189 | 31 | 0 | 0 | 0 | 0 | 0 | 0.162 | 1 | 23 | 1 | 1 | 0 | 0 | 0 | 1 | 7 | 114.3 |
| 271 | 950 | 255 | 873 | 0 | 0 | 189 | 31 | 0 | 0 | 0 | 0 | 0 | 0.157 | 1 | 23 | 1 | 1 | 0 | 0 | 0 | 1 | 28 | 132.5 |
| 272 | 941.5 | 255 | 873 | 9.5 | 0 | 189 | 31 | 0 | 0 | 0 | 0 | 0 | 0.158 | 1 | 23 | 1 | 1 | 0 | 0 | 0 | 1 | 28 | 135.8 |
| 273 | 932 | 255 | 873 | 19 | 0 | 189 | 31 | 0 | 0 | 0 | 0 | 0 | 0.159 | 1 | 23 | 1 | 1 | 0 | 0 | 0 | 1 | 28 | 137 |
| 274 | 921.5 | 255 | 873 | 28.5 | 0 | 189 | 31 | 0 | 0 | 0 | 0 | 0 | 0.161 | 1 | 23 | 1 | 1 | 0 | 0 | 0 | 1 | 28 | 143.2 |
| 275 | 912 | 255 | 873 | 38 | 0 | 189 | 31 | 0 | 0 | 0 | 0 | 0 | 0.162 | 1 | 23 | 1 | 1 | 0 | 0 | 0 | 1 | 28 | 139.2 |
| 276 | 950 | 255 | 873 | 0 | 0 | 189 | 31 | 0 | 0 | 0 | 0 | 0 | 0.157 | 1 | 23 | 1 | 1 | 0 | 0 | 0 | 1 | 90 | 138.7 |
| 277 | 941.5 | 255 | 873 | 9.5 | 0 | 189 | 31 | 0 | 0 | 0 | 0 | 0 | 0.158 | 1 | 23 | 1 | 1 | 0 | 0 | 0 | 1 | 90 | 139.8 |
| 278 | 932 | 255 | 873 | 19 | 0 | 189 | 31 | 0 | 0 | 0 | 0 | 0 | 0.159 | 1 | 23 | 1 | 1 | 0 | 0 | 0 | 1 | 90 | 141.5 |
| 279 | 810 | 90 | 1539 | 0 | 0 | 162 | 18 | 0 | 0 | 0 | 0 | 0 | 0.180 | 1 | 20 | 1 | 1 | 0 | 0 | 0 | 1 | 180 | 174 |
| 280 | 921.5 | 255 | 873 | 28.5 | 0 | 189 | 31 | 0 | 0 | 0 | 0 | 0 | 0.161 | 1 | 23 | 1 | 1 | 0 | 0 | 0 | 1 | 90 | 147.7 |
| 281 | 912 | 255 | 873 | 38 | 0 | 189 | 31 | 0 | 0 | 0 | 0 | 0 | 0.162 | 1 | 23 | 1 | 1 | 0 | 0 | 0 | 1 | 90 | 140.4 |
| 282 | 800 | 0 | 1471.3 | 0 | 0 | 160 | 21.6 | 0 | 0 | 0 | 0 | 0 | 0.200 | 1 | 23 | 1 | 1 | 0 | 0 | 0 | 1 | 1 | 92.2 |
| 283 | 796 | 0 | 1461.1 | 4 | 0 | 160 | 25.2 | 0 | 0 | 0 | 0 | 0 | 0.201 | 1 | 23 | 1 | 1 | 0 | 0 | 0 | 1 | 1 | 82.8 |
| 284 | 792 | 0 | 1450.8 | 8 | 0 | 160 | 28.8 | 0 | 0 | 0 | 0 | 0 | 0.202 | 1 | 23 | 1 | 1 | 0 | 0 | 0 | 1 | 1 | 78.9 |
| 285 | 784 | 0 | 1430.2 | 16 | 0 | 160 | 36 | 0 | 0 | 0 | 0 | 0 | 0.204 | 1 | 23 | 1 | 1 | 0 | 0 | 0 | 1 | 1 | 77.9 |
| 286 | 776 | 0 | 1409.7 | 24 | 0 | 160 | 43.2 | 0 | 0 | 0 | 0 | 0 | 0.206 | 1 | 23 | 1 | 1 | 0 | 0 | 0 | 1 | 1 | 75.6 |
| 287 | 720 | 80 | 1422.9 | 0 | 0 | 160 | 29.6 | 0 | 0 | 0 | 0 | 0 | 0.200 | 1 | 23 | 1 | 1 | 0 | 0 | 0 | 1 | 1 | 81.8 |
| 288 | 716 | 80 | 1411.6 | 4 | 0 | 160 | 33.6 | 0 | 0 | 0 | 0 | 0 | 0.201 | 1 | 23 | 1 | 1 | 0 | 0 | 0 | 1 | 1 | 77.8 |
| 289 | 712 | 80 | 1400.3 | 8 | 0 | 160 | 37.6 | 0 | 0 | 0 | 0 | 0 | 0.202 | 1 | 23 | 1 | 1 | 0 | 0 | 0 | 1 | 1 | 69.4 |
| 290 | 810 | 90 | 1539 | 0 | 0 | 162 | 18 | 0 | 0 | 0 | 0 | 0 | 0.180 | 1 | 20 | 1 | 1 | 0 | 0 | 0 | 1 | 180 | 174 |
| 291 | 704 | 80 | 1379.8 | 16 | 0 | 160 | 44.8 | 0 | 0 | 0 | 0 | 0 | 0.204 | 1 | 23 | 1 | 1 | 0 | 0 | 0 | 1 | 1 | 61.5 |
| 292 | 696 | 80 | 1359.2 | 24 | 0 | 160 | 52 | 0 | 0 | 0 | 0 | 0 | 0.206 | 1 | 23 | 1 | 1 | 0 | 0 | 0 | 1 | 1 | 56.7 |
| 293 | 800 | 0 | 1471.3 | 0 | 0 | 160 | 21.6 | 0 | 0 | 0 | 0 | 0 | 0.200 | 1 | 23 | 1 | 1 | 0 | 0 | 0 | 1 | 3 | 103.9 |
| 294 | 796 | 0 | 1461.1 | 4 | 0 | 160 | 25.2 | 0 | 0 | 0 | 0 | 0 | 0.201 | 1 | 23 | 1 | 1 | 0 | 0 | 0 | 1 | 3 | 100.2 |
| 295 | 792 | 0 | 1450.8 | 8 | 0 | 160 | 28.8 | 0 | 0 | 0 | 0 | 0 | 0.202 | 1 | 23 | 1 | 1 | 0 | 0 | 0 | 1 | 3 | 99.3 |
| 296 | 784 | 0 | 1430.2 | 16 | 0 | 160 | 36 | 0 | 0 | 0 | 0 | 0 | 0.204 | 1 | 23 | 1 | 1 | 0 | 0 | 0 | 1 | 3 | 95.4 |
| 297 | 776 | 0 | 1409.7 | 24 | 0 | 160 | 43.2 | 0 | 0 | 0 | 0 | 0 | 0.206 | 1 | 23 | 1 | 1 | 0 | 0 | 0 | 1 | 3 | 94.5 |
| 298 | 720 | 80 | 1422.9 | 0 | 0 | 160 | 29.6 | 0 | 0 | 0 | 0 | 0 | 0.200 | 1 | 23 | 1 | 1 | 0 | 0 | 0 | 1 | 3 | 91.2 |
| 299 | 716 | 80 | 1411.6 | 4 | 0 | 160 | 33.6 | 0 | 0 | 0 | 0 | 0 | 0.201 | 1 | 23 | 1 | 1 | 0 | 0 | 0 | 1 | 3 | 87.9 |
| 300 | 712 | 80 | 1400.3 | 8 | 0 | 160 | 37.6 | 0 | 0 | 0 | 0 | 0 | 0.202 | 1 | 23 | 1 | 1 | 0 | 0 | 0 | 1 | 3 | 86.3 |
| 301 | 630 | 90 | 1539 | 0 | 0 | 162 | 18 | 0 | 0 | 180 | 0 | 0 | 0.225 | 1 | 20 | 1 | 1 | 0 | 0 | 0 | 1 | 180 | 170 |
| 302 | 704 | 80 | 1379.8 | 16 | 0 | 160 | 44.8 | 0 | 0 | 0 | 0 | 0 | 0.204 | 1 | 23 | 1 | 1 | 0 | 0 | 0 | 1 | 3 | 85.3 |
| 303 | 696 | 80 | 1359.2 | 24 | 0 | 160 | 52 | 0 | 0 | 0 | 0 | 0 | 0.206 | 1 | 23 | 1 | 1 | 0 | 0 | 0 | 1 | 3 | 84.3 |
| 304 | 800 | 0 | 1471.3 | 0 | 0 | 160 | 21.6 | 0 | 0 | 0 | 0 | 0 | 0.200 | 1 | 23 | 1 | 1 | 0 | 0 | 0 | 1 | 7 | 108 |
| 305 | 796 | 0 | 1461.1 | 4 | 0 | 160 | 25.2 | 0 | 0 | 0 | 0 | 0 | 0.201 | 1 | 23 | 1 | 1 | 0 | 0 | 0 | 1 | 7 | 108.7 |
| 306 | 792 | 0 | 1450.8 | 8 | 0 | 160 | 28.8 | 0 | 0 | 0 | 0 | 0 | 0.202 | 1 | 23 | 1 | 1 | 0 | 0 | 0 | 1 | 7 | 109 |
| 307 | 784 | 0 | 1430.2 | 16 | 0 | 160 | 36 | 0 | 0 | 0 | 0 | 0 | 0.204 | 1 | 23 | 1 | 1 | 0 | 0 | 0 | 1 | 7 | 109.2 |
| 308 | 776 | 0 | 1409.7 | 24 | 0 | 160 | 43.2 | 0 | 0 | 0 | 0 | 0 | 0.206 | 1 | 23 | 1 | 1 | 0 | 0 | 0 | 1 | 7 | 106.9 |
| 309 | 720 | 80 | 1422.9 | 0 | 0 | 160 | 29.6 | 0 | 0 | 0 | 0 | 0 | 0.200 | 1 | 23 | 1 | 1 | 0 | 0 | 0 | 1 | 7 | 104.3 |
| 310 | 716 | 80 | 1411.6 | 4 | 0 | 160 | 33.6 | 0 | 0 | 0 | 0 | 0 | 0.201 | 1 | 23 | 1 | 1 | 0 | 0 | 0 | 1 | 7 | 105.1 |
| 311 | 712 | 80 | 1400.3 | 8 | 0 | 160 | 37.6 | 0 | 0 | 0 | 0 | 0 | 0.202 | 1 | 23 | 1 | 1 | 0 | 0 | 0 | 1 | 7 | 106.2 |
| 312 | 630 | 90 | 1539 | 0 | 0 | 126 | 18 | 0 | 0 | 180 | 0 | 0 | 0.175 | 1 | 20 | 1 | 1 | 0 | 0 | 0 | 1 | 180 | 168 |
| 313 | 704 | 80 | 1379.8 | 16 | 0 | 160 | 44.8 | 0 | 0 | 0 | 0 | 0 | 0.204 | 1 | 23 | 1 | 1 | 0 | 0 | 0 | 1 | 7 | 107.1 |
| 314 | 696 | 80 | 1359.2 | 24 | 0 | 160 | 52 | 0 | 0 | 0 | 0 | 0 | 0.206 | 1 | 23 | 1 | 1 | 0 | 0 | 0 | 1 | 7 | 102.2 |
| 315 | 800 | 0 | 1471.3 | 0 | 0 | 160 | 21.6 | 0 | 0 | 0 | 0 | 0 | 0.200 | 1 | 23 | 1 | 1 | 0 | 0 | 0 | 1 | 14 | 112.4 |
| 316 | 796 | 0 | 1461.1 | 4 | 0 | 160 | 25.2 | 0 | 0 | 0 | 0 | 0 | 0.201 | 1 | 23 | 1 | 1 | 0 | 0 | 0 | 1 | 14 | 113.1 |
| 317 | 792 | 0 | 1450.8 | 8 | 0 | 160 | 28.8 | 0 | 0 | 0 | 0 | 0 | 0.202 | 1 | 23 | 1 | 1 | 0 | 0 | 0 | 1 | 14 | 114.3 |
| 318 | 784 | 0 | 1430.2 | 16 | 0 | 160 | 36 | 0 | 0 | 0 | 0 | 0 | 0.204 | 1 | 23 | 1 | 1 | 0 | 0 | 0 | 1 | 14 | 115.6 |
| 319 | 776 | 0 | 1409.7 | 24 | 0 | 160 | 43.2 | 0 | 0 | 0 | 0 | 0 | 0.206 | 1 | 23 | 1 | 1 | 0 | 0 | 0 | 1 | 14 | 111.5 |
| 320 | 720 | 80 | 1422.9 | 0 | 0 | 160 | 29.6 | 0 | 0 | 0 | 0 | 0 | 0.200 | 1 | 23 | 1 | 1 | 0 | 0 | 0 | 1 | 14 | 116.2 |
| 321 | 716 | 80 | 1411.6 | 4 | 0 | 160 | 33.6 | 0 | 0 | 0 | 0 | 0 | 0.201 | 1 | 23 | 1 | 1 | 0 | 0 | 0 | 1 | 14 | 118.5 |
| 322 | 712 | 80 | 1400.3 | 8 | 0 | 160 | 37.6 | 0 | 0 | 0 | 0 | 0 | 0.202 | 1 | 23 | 1 | 1 | 0 | 0 | 0 | 1 | 14 | 123 |
| 323 | 630 | 90 | 1539 | 0 | 0 | 144 | 18 | 0 | 0 | 180 | 0 | 0 | 0.200 | 1 | 20 | 1 | 1 | 0 | 0 | 0 | 1 | 180 | 175 |
| 324 | 704 | 80 | 1379.8 | 16 | 0 | 160 | 44.8 | 0 | 0 | 0 | 0 | 0 | 0.204 | 1 | 23 | 1 | 1 | 0 | 0 | 0 | 1 | 14 | 126 |
| 325 | 696 | 80 | 1359.2 | 24 | 0 | 160 | 52 | 0 | 0 | 0 | 0 | 0 | 0.206 | 1 | 23 | 1 | 1 | 0 | 0 | 0 | 1 | 14 | 119.2 |
| 326 | 800 | 0 | 1471.3 | 0 | 0 | 160 | 21.6 | 0 | 0 | 0 | 0 | 0 | 0.200 | 1 | 23 | 1 | 1 | 0 | 0 | 0 | 1 | 28 | 115.4 |
| 327 | 796 | 0 | 1461.1 | 4 | 0 | 160 | 25.2 | 0 | 0 | 0 | 0 | 0 | 0.201 | 1 | 23 | 1 | 1 | 0 | 0 | 0 | 1 | 28 | 116.8 |
| 328 | 792 | 0 | 1450.8 | 8 | 0 | 160 | 28.8 | 0 | 0 | 0 | 0 | 0 | 0.202 | 1 | 23 | 1 | 1 | 0 | 0 | 0 | 1 | 28 | 120.9 |
| 329 | 784 | 0 | 1430.2 | 16 | 0 | 160 | 36 | 0 | 0 | 0 | 0 | 0 | 0.204 | 1 | 23 | 1 | 1 | 0 | 0 | 0 | 1 | 28 | 123.4 |
| 330 | 776 | 0 | 1409.7 | 24 | 0 | 160 | 43.2 | 0 | 0 | 0 | 0 | 0 | 0.206 | 1 | 23 | 1 | 1 | 0 | 0 | 0 | 1 | 28 | 119.1 |
| 331 | 720 | 80 | 1422.9 | 0 | 0 | 160 | 29.6 | 0 | 0 | 0 | 0 | 0 | 0.200 | 1 | 23 | 1 | 1 | 0 | 0 | 0 | 1 | 28 | 120.7 |
| 332 | 716 | 80 | 1411.6 | 4 | 0 | 160 | 33.6 | 0 | 0 | 0 | 0 | 0 | 0.201 | 1 | 23 | 1 | 1 | 0 | 0 | 0 | 1 | 28 | 125 |
| 333 | 712 | 80 | 1400.3 | 8 | 0 | 160 | 37.6 | 0 | 0 | 0 | 0 | 0 | 0.202 | 1 | 23 | 1 | 1 | 0 | 0 | 0 | 1 | 28 | 130.1 |
| 334 | 810 | 90 | 1539 | 0 | 0 | 162 | 18 | 0 | 0 | 0 | 0 | 0 | 0.180 | 1 | 20 | 1 | 1 | 0 | 0 | 0 | 1 | 28 | 137 |
| 335 | 630 | 90 | 1539 | 0 | 0 | 162 | 18 | 0 | 0 | 180 | 0 | 0 | 0.225 | 1 | 20 | 1 | 1 | 0 | 0 | 0 | 1 | 180 | 170 |
| 336 | 704 | 80 | 1379.8 | 16 | 0 | 160 | 44.8 | 0 | 0 | 0 | 0 | 0 | 0.204 | 1 | 23 | 1 | 1 | 0 | 0 | 0 | 1 | 28 | 131.8 |
| 337 | 696 | 80 | 1359.2 | 24 | 0 | 160 | 52 | 0 | 0 | 0 | 0 | 0 | 0.206 | 1 | 23 | 1 | 1 | 0 | 0 | 0 | 1 | 28 | 127.3 |
| 338 | 800 | 0 | 1471.3 | 0 | 0 | 160 | 21.6 | 0 | 0 | 0 | 0 | 0 | 0.200 | 1 | 23 | 1 | 1 | 0 | 0 | 0 | 1 | 56 | 119.3 |
| 339 | 796 | 0 | 1461.1 | 4 | 0 | 160 | 25.2 | 0 | 0 | 0 | 0 | 0 | 0.201 | 1 | 23 | 1 | 1 | 0 | 0 | 0 | 1 | 56 | 120.9 |
| 340 | 792 | 0 | 1450.8 | 8 | 0 | 160 | 28.8 | 0 | 0 | 0 | 0 | 0 | 0.202 | 1 | 23 | 1 | 1 | 0 | 0 | 0 | 1 | 56 | 123.4 |
| 341 | 784 | 0 | 1430.2 | 16 | 0 | 160 | 36 | 0 | 0 | 0 | 0 | 0 | 0.204 | 1 | 23 | 1 | 1 | 0 | 0 | 0 | 1 | 56 | 126 |
| 342 | 776 | 0 | 1409.7 | 24 | 0 | 160 | 43.2 | 0 | 0 | 0 | 0 | 0 | 0.206 | 1 | 23 | 1 | 1 | 0 | 0 | 0 | 1 | 56 | 122.3 |
| 343 | 720 | 80 | 1422.9 | 0 | 0 | 160 | 29.6 | 0 | 0 | 0 | 0 | 0 | 0.200 | 1 | 23 | 1 | 1 | 0 | 0 | 0 | 1 | 56 | 124.2 |
| 344 | 716 | 80 | 1411.6 | 4 | 0 | 160 | 33.6 | 0 | 0 | 0 | 0 | 0 | 0.201 | 1 | 23 | 1 | 1 | 0 | 0 | 0 | 1 | 56 | 128.8 |
| 345 | 712 | 80 | 1400.3 | 8 | 0 | 160 | 37.6 | 0 | 0 | 0 | 0 | 0 | 0.202 | 1 | 23 | 1 | 1 | 0 | 0 | 0 | 1 | 56 | 134.8 |
| 346 | 630 | 90 | 1539 | 0 | 0 | 144 | 18 | 0 | 0 | 180 | 0 | 0 | 0.200 | 1 | 20 | 1 | 1 | 0 | 0 | 0 | 1 | 180 | 175 |
| 347 | 704 | 80 | 1379.8 | 16 | 0 | 160 | 44.8 | 0 | 0 | 0 | 0 | 0 | 0.204 | 1 | 23 | 1 | 1 | 0 | 0 | 0 | 1 | 56 | 137.4 |
| 348 | 696 | 80 | 1359.2 | 24 | 0 | 160 | 52 | 0 | 0 | 0 | 0 | 0 | 0.206 | 1 | 23 | 1 | 1 | 0 | 0 | 0 | 1 | 56 | 131.8 |
| 349 | 800 | 0 | 1471.3 | 0 | 0 | 160 | 21.6 | 0 | 0 | 0 | 0 | 0 | 0.200 | 1 | 23 | 1 | 1 | 0 | 0 | 0 | 1 | 90 | 124.1 |
| 350 | 796 | 0 | 1461.1 | 4 | 0 | 160 | 25.2 | 0 | 0 | 0 | 0 | 0 | 0.201 | 1 | 23 | 1 | 1 | 0 | 0 | 0 | 1 | 90 | 125.7 |
| 351 | 792 | 0 | 1450.8 | 8 | 0 | 160 | 28.8 | 0 | 0 | 0 | 0 | 0 | 0.202 | 1 | 23 | 1 | 1 | 0 | 0 | 0 | 1 | 90 | 130.6 |
| 352 | 784 | 0 | 1430.2 | 16 | 0 | 160 | 36 | 0 | 0 | 0 | 0 | 0 | 0.204 | 1 | 23 | 1 | 1 | 0 | 0 | 0 | 1 | 90 | 133.3 |
| 353 | 776 | 0 | 1409.7 | 24 | 0 | 160 | 43.2 | 0 | 0 | 0 | 0 | 0 | 0.206 | 1 | 23 | 1 | 1 | 0 | 0 | 0 | 1 | 90 | 129.9 |
| 354 | 720 | 80 | 1422.9 | 0 | 0 | 160 | 29.6 | 0 | 0 | 0 | 0 | 0 | 0.200 | 1 | 23 | 1 | 1 | 0 | 0 | 0 | 1 | 90 | 130.3 |
| 355 | 716 | 80 | 1411.6 | 4 | 0 | 160 | 33.6 | 0 | 0 | 0 | 0 | 0 | 0.201 | 1 | 23 | 1 | 1 | 0 | 0 | 0 | 1 | 90 | 132.8 |
| 356 | 712 | 80 | 1400.3 | 8 | 0 | 160 | 37.6 | 0 | 0 | 0 | 0 | 0 | 0.202 | 1 | 23 | 1 | 1 | 0 | 0 | 0 | 1 | 90 | 137.9 |
| 357 | 450 | 90 | 1539 | 0 | 0 | 144 | 18 | 0 | 0 | 180 | 0 | 180 | 0.267 | 1 | 20 | 1 | 1 | 0 | 0 | 0 | 1 | 180 | 179 |
| 358 | 704 | 80 | 1379.8 | 16 | 0 | 160 | 44.8 | 0 | 0 | 0 | 0 | 0 | 0.204 | 1 | 23 | 1 | 1 | 0 | 0 | 0 | 1 | 90 | 141.6 |
| 359 | 696 | 80 | 1359.2 | 24 | 0 | 160 | 52 | 0 | 0 | 0 | 0 | 0 | 0.206 | 1 | 23 | 1 | 1 | 0 | 0 | 0 | 1 | 90 | 138.3 |
| 360 | 550 | 0 | 1695 | 0 | 0 | 192.5 | 1.1 | 0 | 0 | 0 | 0 | 0 | 0.350 | 1 | 21 | 1 | 1 | 0 | 0 | 0 | 1 | 3 | 32.5 |
| 361 | 545.875 | 0 | 1685 | 4.125 | 0 | 192.5 | 4.4 | 0 | 0 | 0 | 0 | 0 | 0.353 | 1 | 21 | 1 | 1 | 0 | 0 | 0 | 1 | 3 | 35.3 |
| 362 | 541.75 | 0 | 1678 | 8.25 | 0 | 192.5 | 6.6 | 0 | 0 | 0 | 0 | 0 | 0.355 | 1 | 21 | 1 | 1 | 0 | 0 | 0 | 1 | 3 | 39.5 |
| 363 | 545.875 | 0 | 1685 | 4.125 | 0 | 192.5 | 4.4 | 0 | 0 | 0 | 0 | 0 | 0.353 | 1 | 21 | 1 | 1 | 0 | 0 | 0 | 1 | 3 | 37.1 |
| 364 | 541.75 | 0 | 1678 | 8.25 | 0 | 192.5 | 6.6 | 0 | 0 | 0 | 0 | 0 | 0.355 | 1 | 21 | 1 | 1 | 0 | 0 | 0 | 1 | 3 | 39.1 |
| 365 | 550 | 0 | 1762 | 0 | 0 | 165 | 3.85 | 0 | 0 | 0 | 0 | 0 | 0.300 | 1 | 21 | 1 | 1 | 0 | 0 | 0 | 1 | 3 | 45 |
| 366 | 545.875 | 0 | 1754 | 4.125 | 0 | 165 | 6.6 | 0 | 0 | 0 | 0 | 0 | 0.302 | 1 | 21 | 1 | 1 | 0 | 0 | 0 | 1 | 3 | 46.4 |
| 367 | 541.75 | 0 | 1748 | 8.25 | 0 | 165 | 8.25 | 0 | 0 | 0 | 0 | 0 | 0.305 | 1 | 21 | 1 | 1 | 0 | 0 | 0 | 1 | 3 | 51.6 |
| 368 | 270 | 90 | 1539 | 0 | 0 | 144 | 18 | 0 | 0 | 180 | 0 | 360 | 0.400 | 1 | 20 | 1 | 1 | 0 | 0 | 0 | 1 | 180 | 160 |
| 369 | 545.875 | 0 | 1754 | 4.125 | 0 | 165 | 6.6 | 0 | 0 | 0 | 0 | 0 | 0.302 | 1 | 21 | 1 | 1 | 0 | 0 | 0 | 1 | 3 | 45.4 |
| 370 | 541.75 | 0 | 1748 | 8.25 | 0 | 165 | 8.25 | 0 | 0 | 0 | 0 | 0 | 0.305 | 1 | 21 | 1 | 1 | 0 | 0 | 0 | 1 | 3 | 42.2 |
| 371 | 550 | 0 | 1830 | 0 | 0 | 137.5 | 6.6 | 0 | 0 | 0 | 0 | 0 | 0.250 | 1 | 21 | 1 | 1 | 0 | 0 | 0 | 1 | 3 | 58.9 |
| 372 | 545.875 | 0 | 1820 | 4.125 | 0 | 137.5 | 9.9 | 0 | 0 | 0 | 0 | 0 | 0.252 | 1 | 21 | 1 | 1 | 0 | 0 | 0 | 1 | 3 | 59.8 |
| 373 | 541.75 | 0 | 1812 | 8.25 | 0 | 137.5 | 12.65 | 0 | 0 | 0 | 0 | 0 | 0.254 | 1 | 21 | 1 | 1 | 0 | 0 | 0 | 1 | 3 | 63.3 |
| 374 | 545.875 | 0 | 1820 | 4.125 | 0 | 137.5 | 9.9 | 0 | 0 | 0 | 0 | 0 | 0.252 | 1 | 21 | 1 | 1 | 0 | 0 | 0 | 1 | 3 | 56.1 |
| 375 | 541.75 | 0 | 1812 | 8.25 | 0 | 137.5 | 12.65 | 0 | 0 | 0 | 0 | 0 | 0.254 | 1 | 21 | 1 | 1 | 0 | 0 | 0 | 1 | 3 | 53.1 |
| 376 | 550 | 0 | 1695 | 0 | 0 | 192.5 | 1.1 | 0 | 0 | 0 | 0 | 0 | 0.350 | 1 | 21 | 1 | 1 | 0 | 0 | 0 | 1 | 7 | 40.9 |
| 377 | 545.875 | 0 | 1685 | 4.125 | 0 | 192.5 | 4.4 | 0 | 0 | 0 | 0 | 0 | 0.353 | 1 | 21 | 1 | 1 | 0 | 0 | 0 | 1 | 7 | 44.9 |
| 378 | 541.75 | 0 | 1678 | 8.25 | 0 | 192.5 | 6.6 | 0 | 0 | 0 | 0 | 0 | 0.355 | 1 | 21 | 1 | 1 | 0 | 0 | 0 | 1 | 7 | 51.3 |
| 379 | 450 | 50 | 1992 | 0 | 0 | 90 | 18 | 0 | 0 | 0 | 0 | 0 | 0.180 | 1 | 20 | 1 | 1 | 0 | 0 | 0 | 1 | 365 | 161 |
| 380 | 545.875 | 0 | 1685 | 4.125 | 0 | 192.5 | 4.4 | 0 | 0 | 0 | 0 | 0 | 0.353 | 1 | 21 | 1 | 1 | 0 | 0 | 0 | 1 | 7 | 45.7 |
| 381 | 541.75 | 0 | 1678 | 8.25 | 0 | 192.5 | 6.6 | 0 | 0 | 0 | 0 | 0 | 0.355 | 1 | 21 | 1 | 1 | 0 | 0 | 0 | 1 | 7 | 49.3 |
| 382 | 550 | 0 | 1762 | 0 | 0 | 165 | 3.85 | 0 | 0 | 0 | 0 | 0 | 0.300 | 1 | 21 | 1 | 1 | 0 | 0 | 0 | 1 | 7 | 57.2 |
| 383 | 545.875 | 0 | 1754 | 4.125 | 0 | 165 | 6.6 | 0 | 0 | 0 | 0 | 0 | 0.302 | 1 | 21 | 1 | 1 | 0 | 0 | 0 | 1 | 7 | 59.7 |
| 384 | 541.75 | 0 | 1748 | 8.25 | 0 | 165 | 8.25 | 0 | 0 | 0 | 0 | 0 | 0.305 | 1 | 21 | 1 | 1 | 0 | 0 | 0 | 1 | 7 | 60.1 |
| 385 | 545.875 | 0 | 1754 | 4.125 | 0 | 165 | 6.6 | 0 | 0 | 0 | 0 | 0 | 0.302 | 1 | 21 | 1 | 1 | 0 | 0 | 0 | 1 | 7 | 52.5 |
| 386 | 541.75 | 0 | 1748 | 8.25 | 0 | 165 | 8.25 | 0 | 0 | 0 | 0 | 0 | 0.305 | 1 | 21 | 1 | 1 | 0 | 0 | 0 | 1 | 7 | 50.1 |
| 387 | 550 | 0 | 1830 | 0 | 0 | 137.5 | 6.6 | 0 | 0 | 0 | 0 | 0 | 0.250 | 1 | 21 | 1 | 1 | 0 | 0 | 0 | 1 | 7 | 71.6 |
| 388 | 545.875 | 0 | 1820 | 4.125 | 0 | 137.5 | 9.9 | 0 | 0 | 0 | 0 | 0 | 0.252 | 1 | 21 | 1 | 1 | 0 | 0 | 0 | 1 | 7 | 76 |
| 389 | 541.75 | 0 | 1812 | 8.25 | 0 | 137.5 | 12.65 | 0 | 0 | 0 | 0 | 0 | 0.254 | 1 | 21 | 1 | 1 | 0 | 0 | 0 | 1 | 7 | 75 |
| 390 | 630 | 70 | 1788 | 0 | 0 | 126 | 18 | 0 | 0 | 0 | 0 | 0 | 0.180 | 1 | 20 | 1 | 1 | 0 | 0 | 0 | 1 | 365 | 169 |
| 391 | 545.875 | 0 | 1820 | 4.125 | 0 | 137.5 | 9.9 | 0 | 0 | 0 | 0 | 0 | 0.252 | 1 | 21 | 1 | 1 | 0 | 0 | 0 | 1 | 7 | 69 |
| 392 | 541.75 | 0 | 1812 | 8.25 | 0 | 137.5 | 12.65 | 0 | 0 | 0 | 0 | 0 | 0.254 | 1 | 21 | 1 | 1 | 0 | 0 | 0 | 1 | 7 | 67.8 |
| 393 | 545.875 | 0 | 1685 | 4.125 | 0 | 192.5 | 4.4 | 0 | 0 | 0 | 0 | 0 | 0.353 | 1 | 21 | 1 | 1 | 0 | 0 | 0 | 1 | 28 | 53.4 |
| 394 | 541.75 | 0 | 1678 | 8.25 | 0 | 192.5 | 6.6 | 0 | 0 | 0 | 0 | 0 | 0.355 | 1 | 21 | 1 | 1 | 0 | 0 | 0 | 1 | 28 | 61.8 |
| 395 | 545.875 | 0 | 1685 | 4.125 | 0 | 192.5 | 4.4 | 0 | 0 | 0 | 0 | 0 | 0.353 | 1 | 21 | 1 | 1 | 0 | 0 | 0 | 1 | 28 | 54.5 |
| 396 | 541.75 | 0 | 1678 | 8.25 | 0 | 192.5 | 6.6 | 0 | 0 | 0 | 0 | 0 | 0.355 | 1 | 21 | 1 | 1 | 0 | 0 | 0 | 1 | 28 | 61 |
| 397 | 550 | 0 | 1762 | 0 | 0 | 165 | 3.85 | 0 | 0 | 0 | 0 | 0 | 0.300 | 1 | 21 | 1 | 1 | 0 | 0 | 0 | 1 | 28 | 67.2 |
| 398 | 545.875 | 0 | 1754 | 4.125 | 0 | 165 | 6.6 | 0 | 0 | 0 | 0 | 0 | 0.302 | 1 | 21 | 1 | 1 | 0 | 0 | 0 | 1 | 28 | 64.4 |
| 399 | 541.75 | 0 | 1748 | 8.25 | 0 | 165 | 8.25 | 0 | 0 | 0 | 0 | 0 | 0.305 | 1 | 21 | 1 | 1 | 0 | 0 | 0 | 1 | 28 | 72.6 |
| 400 | 545.875 | 0 | 1754 | 4.125 | 0 | 165 | 6.6 | 0 | 0 | 0 | 0 | 0 | 0.302 | 1 | 21 | 1 | 1 | 0 | 0 | 0 | 1 | 28 | 64.2 |
| 401 | 810 | 90 | 1539 | 0 | 0 | 162 | 18 | 0 | 0 | 0 | 0 | 0 | 0.180 | 1 | 20 | 1 | 1 | 0 | 0 | 0 | 1 | 365 | 177 |
| 402 | 541.75 | 0 | 1748 | 8.25 | 0 | 165 | 8.25 | 0 | 0 | 0 | 0 | 0 | 0.305 | 1 | 21 | 1 | 1 | 0 | 0 | 0 | 1 | 28 | 62.9 |
| 403 | 550 | 0 | 1830 | 0 | 0 | 137.5 | 6.6 | 0 | 0 | 0 | 0 | 0 | 0.250 | 1 | 21 | 1 | 1 | 0 | 0 | 0 | 1 | 28 | 85.5 |
| 404 | 545.875 | 0 | 1820 | 4.125 | 0 | 137.5 | 9.9 | 0 | 0 | 0 | 0 | 0 | 0.252 | 1 | 21 | 1 | 1 | 0 | 0 | 0 | 1 | 28 | 86.7 |
| 405 | 541.75 | 0 | 1812 | 8.25 | 0 | 137.5 | 12.65 | 0 | 0 | 0 | 0 | 0 | 0.254 | 1 | 21 | 1 | 1 | 0 | 0 | 0 | 1 | 28 | 85 |
| 406 | 545.875 | 0 | 1820 | 4.125 | 0 | 137.5 | 9.9 | 0 | 0 | 0 | 0 | 0 | 0.252 | 1 | 21 | 1 | 1 | 0 | 0 | 0 | 1 | 28 | 83.6 |
| 407 | 541.75 | 0 | 1812 | 8.25 | 0 | 137.5 | 12.65 | 0 | 0 | 0 | 0 | 0 | 0.254 | 1 | 21 | 1 | 1 | 0 | 0 | 0 | 1 | 28 | 84.7 |
| 408 | 545.875 | 0 | 1685 | 4.125 | 0 | 192.5 | 4.4 | 0 | 0 | 0 | 0 | 0 | 0.353 | 1 | 21 | 1 | 1 | 0 | 0 | 0 | 1 | 90 | 62 |
| 409 | 541.75 | 0 | 1678 | 8.25 | 0 | 192.5 | 6.6 | 0 | 0 | 0 | 0 | 0 | 0.355 | 1 | 21 | 1 | 1 | 0 | 0 | 0 | 1 | 90 | 65.9 |
| 410 | 545.875 | 0 | 1685 | 4.125 | 0 | 192.5 | 4.4 | 0 | 0 | 0 | 0 | 0 | 0.353 | 1 | 21 | 1 | 1 | 0 | 0 | 0 | 1 | 90 | 58.4 |
| 411 | 541.75 | 0 | 1678 | 8.25 | 0 | 192.5 | 6.6 | 0 | 0 | 0 | 0 | 0 | 0.355 | 1 | 21 | 1 | 1 | 0 | 0 | 0 | 1 | 90 | 65.3 |
| 412 | 810 | 90 | 1539 | 0 | 0 | 162 | 18 | 0 | 0 | 0 | 0 | 0 | 0.180 | 1 | 20 | 1 | 1 | 0 | 0 | 0 | 1 | 365 | 177 |
| 413 | 550 | 0 | 1762 | 0 | 0 | 165 | 3.85 | 0 | 0 | 0 | 0 | 0 | 0.300 | 1 | 21 | 1 | 1 | 0 | 0 | 0 | 1 | 90 | 74.3 |
| 414 | 545.875 | 0 | 1754 | 4.125 | 0 | 165 | 6.6 | 0 | 0 | 0 | 0 | 0 | 0.302 | 1 | 21 | 1 | 1 | 0 | 0 | 0 | 1 | 90 | 75.1 |
| 415 | 541.75 | 0 | 1748 | 8.25 | 0 | 165 | 8.25 | 0 | 0 | 0 | 0 | 0 | 0.305 | 1 | 21 | 1 | 1 | 0 | 0 | 0 | 1 | 90 | 76.9 |
| 416 | 545.875 | 0 | 1754 | 4.125 | 0 | 165 | 6.6 | 0 | 0 | 0 | 0 | 0 | 0.302 | 1 | 21 | 1 | 1 | 0 | 0 | 0 | 1 | 90 | 71.9 |
| 417 | 541.75 | 0 | 1748 | 8.25 | 0 | 165 | 8.25 | 0 | 0 | 0 | 0 | 0 | 0.305 | 1 | 21 | 1 | 1 | 0 | 0 | 0 | 1 | 90 | 70.8 |
| 418 | 550 | 0 | 1830 | 0 | 0 | 137.5 | 6.6 | 0 | 0 | 0 | 0 | 0 | 0.250 | 1 | 21 | 1 | 1 | 0 | 0 | 0 | 1 | 90 | 93.8 |
| 419 | 545.875 | 0 | 1820 | 4.125 | 0 | 137.5 | 9.9 | 0 | 0 | 0 | 0 | 0 | 0.252 | 1 | 21 | 1 | 1 | 0 | 0 | 0 | 1 | 90 | 95 |
| 420 | 541.75 | 0 | 1812 | 8.25 | 0 | 137.5 | 12.65 | 0 | 0 | 0 | 0 | 0 | 0.254 | 1 | 21 | 1 | 1 | 0 | 0 | 0 | 1 | 90 | 91.2 |
| 421 | 545.875 | 0 | 1820 | 4.125 | 0 | 137.5 | 9.9 | 0 | 0 | 0 | 0 | 0 | 0.252 | 1 | 21 | 1 | 1 | 0 | 0 | 0 | 1 | 90 | 90 |
| 422 | 541.75 | 0 | 1812 | 8.25 | 0 | 137.5 | 12.65 | 0 | 0 | 0 | 0 | 0 | 0.254 | 1 | 21 | 1 | 1 | 0 | 0 | 0 | 1 | 90 | 92.6 |
| 423 | 630 | 90 | 1539 | 0 | 0 | 162 | 18 | 0 | 0 | 180 | 0 | 0 | 0.225 | 1 | 20 | 1 | 1 | 0 | 0 | 0 | 1 | 365 | 174 |
| 424 | 950 | 200 | 570 | 0 | 350 | 230 | 53 | 234 | 0 | 0 | 0 | 0 | 0.200 | 1 | 20 | 1 | 1 | 1 | 13 | 0.2 | 1 | 28 | 140.7 |
| 425 | 931 | 200 | 570 | 19 | 350 | 230 | 53 | 234 | 0 | 0 | 0 | 0 | 0.203 | 1 | 20 | 1 | 1 | 1 | 13 | 0.2 | 1 | 28 | 150.6 |
| 426 | 902.5 | 200 | 570 | 47.5 | 350 | 230 | 53 | 234 | 0 | 0 | 0 | 0 | 0.209 | 1 | 20 | 1 | 1 | 1 | 13 | 0.2 | 1 | 28 | 148.4 |
| 427 | 950 | 200 | 690 | 0 | 350 | 230 | 53 | 234 | 0 | 0 | 0 | 0 | 0.200 | 1 | 20 | 1 | 1 | 1 | 13 | 0.2 | 1 | 28 | 144.7 |
| 428 | 931 | 200 | 690 | 19 | 350 | 230 | 53 | 234 | 0 | 0 | 0 | 0 | 0.203 | 1 | 20 | 1 | 1 | 1 | 13 | 0.2 | 1 | 28 | 155.4 |
| 429 | 902.5 | 200 | 690 | 47.5 | 350 | 230 | 53 | 234 | 0 | 0 | 0 | 0 | 0.209 | 1 | 20 | 1 | 1 | 1 | 13 | 0.2 | 1 | 28 | 142.4 |
| 430 | 950 | 200 | 810 | 0 | 350 | 230 | 53 | 234 | 0 | 0 | 0 | 0 | 0.200 | 1 | 20 | 1 | 1 | 1 | 13 | 0.2 | 1 | 28 | 144.5 |
| 431 | 902.5 | 200 | 810 | 47.5 | 350 | 230 | 53 | 234 | 0 | 0 | 0 | 0 | 0.209 | 1 | 20 | 1 | 1 | 1 | 13 | 0.2 | 1 | 28 | 137.2 |
| 432 | 863 | 216 | 923 | 0 | 0 | 177 | 32.37 | 0 | 0 | 0 | 0 | 0 | 0.164 | 1 | 20 | 1 | 1 | 0 | 0 | 0 | 1 | 7 | 88.9 |
| 433 | 647 | 216 | 923 | 0 | 0 | 177 | 32.37 | 0 | 0 | 216 | 0 | 0 | 0.205 | 1 | 20 | 1 | 1 | 0 | 0 | 0 | 1 | 7 | 86.7 |
| 434 | 630 | 90 | 1539 | 0 | 0 | 126 | 18 | 0 | 0 | 180 | 0 | 0 | 0.175 | 1 | 20 | 1 | 1 | 0 | 0 | 0 | 1 | 365 | 168 |
| 435 | 432 | 216 | 923 | 0 | 0 | 177 | 32.4 | 0 | 216 | 216 | 0 | 0 | 0.273 | 1 | 20 | 1 | 1 | 0 | 0 | 0 | 1 | 7 | 79.2 |
| 436 | 829 | 216 | 923 | 34.5 | 0 | 177 | 32.39 | 0 | 0 | 0 | 0 | 0 | 0.169 | 1 | 20 | 1 | 1 | 0 | 0 | 0 | 1 | 7 | 91.1 |
| 437 | 852 | 216 | 923 | 10.8 | 0 | 177 | 32.36 | 0 | 0 | 0 | 0 | 0 | 0.166 | 1 | 20 | 1 | 1 | 0 | 0 | 0 | 1 | 7 | 101.9 |
| 438 | 863 | 216 | 923 | 0 | 0 | 177 | 32.37 | 156 | 0 | 0 | 0 | 0 | 0.164 | 1 | 20 | 1 | 1 | 1 | 13 | 0.2 | 1 | 7 | 103.7 |
| 439 | 647 | 216 | 923 | 0 | 0 | 177 | 32.37 | 156 | 0 | 216 | 0 | 0 | 0.205 | 1 | 20 | 1 | 1 | 1 | 13 | 0.2 | 1 | 7 | 118.1 |
| 440 | 647 | 216 | 923 | 0 | 0 | 177 | 32.37 | 156 | 216 | 0 | 0 | 0 | 0.205 | 1 | 20 | 1 | 1 | 1 | 13 | 0.2 | 1 | 7 | 118.7 |
| 441 | 432 | 216 | 923 | 0 | 0 | 177 | 32.4 | 156 | 216 | 216 | 0 | 0 | 0.273 | 1 | 20 | 1 | 1 | 1 | 13 | 0.2 | 1 | 7 | 117.6 |
| 442 | 829 | 216 | 923 | 34.5 | 0 | 177 | 32.39 | 156 | 0 | 0 | 0 | 0 | 0.169 | 1 | 20 | 1 | 1 | 1 | 13 | 0.2 | 1 | 7 | 118.7 |
| 443 | 852 | 216 | 923 | 10.8 | 0 | 177 | 32.36 | 156 | 0 | 0 | 0 | 0 | 0.166 | 1 | 20 | 1 | 1 | 1 | 13 | 0.2 | 1 | 7 | 122.6 |
| 444 | 863 | 216 | 923 | 0 | 0 | 177 | 32.37 | 0 | 0 | 0 | 0 | 0 | 0.164 | 1 | 20 | 1 | 1 | 0 | 0 | 0 | 1 | 28 | 104.1 |
| 445 | 630 | 90 | 1539 | 0 | 0 | 126 | 18 | 0 | 0 | 180 | 0 | 0 | 0.175 | 1 | 20 | 1 | 1 | 0 | 0 | 0 | 1 | 28 | 150 |
| 446 | 630 | 90 | 1539 | 0 | 0 | 144 | 18 | 0 | 0 | 180 | 0 | 0 | 0.200 | 1 | 20 | 1 | 1 | 0 | 0 | 0 | 1 | 365 | 178 |
| 447 | 647 | 216 | 923 | 0 | 0 | 177 | 32.37 | 0 | 0 | 216 | 0 | 0 | 0.205 | 1 | 20 | 1 | 1 | 0 | 0 | 0 | 1 | 28 | 109 |
| 448 | 647 | 216 | 923 | 0 | 0 | 177 | 32.37 | 0 | 216 | 0 | 0 | 0 | 0.205 | 1 | 20 | 1 | 1 | 0 | 0 | 0 | 1 | 28 | 109 |
| 449 | 432 | 216 | 923 | 0 | 0 | 177 | 32.4 | 0 | 216 | 216 | 0 | 0 | 0.273 | 1 | 20 | 1 | 1 | 0 | 0 | 0 | 1 | 28 | 103 |
| 450 | 829 | 216 | 923 | 34.5 | 0 | 177 | 32.39 | 0 | 0 | 0 | 0 | 0 | 0.169 | 1 | 20 | 1 | 1 | 0 | 0 | 0 | 1 | 28 | 113.9 |
| 451 | 852 | 216 | 923 | 10.8 | 0 | 177 | 32.36 | 0 | 0 | 0 | 0 | 0 | 0.166 | 1 | 20 | 1 | 1 | 0 | 0 | 0 | 1 | 28 | 113.3 |
| 452 | 863 | 216 | 923 | 0 | 0 | 177 | 32.37 | 156 | 0 | 0 | 0 | 0 | 0.164 | 1 | 20 | 1 | 1 | 1 | 13 | 0.2 | 1 | 28 | 142.1 |
| 453 | 647 | 216 | 923 | 0 | 0 | 177 | 32.37 | 156 | 0 | 216 | 0 | 0 | 0.205 | 1 | 20 | 1 | 1 | 1 | 13 | 0.2 | 1 | 28 | 157.2 |
| 454 | 647 | 216 | 923 | 0 | 0 | 177 | 32.37 | 156 | 216 | 0 | 0 | 0 | 0.205 | 1 | 20 | 1 | 1 | 1 | 13 | 0.2 | 1 | 28 | 156 |
| 455 | 432 | 216 | 923 | 0 | 0 | 177 | 32.4 | 156 | 216 | 216 | 0 | 0 | 0.273 | 1 | 20 | 1 | 1 | 1 | 13 | 0.2 | 1 | 28 | 154.4 |
| 456 | 829 | 216 | 923 | 34.5 | 0 | 177 | 32.39 | 156 | 0 | 0 | 0 | 0 | 0.169 | 1 | 20 | 1 | 1 | 1 | 13 | 0.2 | 1 | 28 | 156 |
| 457 | 630 | 90 | 1539 | 0 | 0 | 162 | 18 | 0 | 0 | 180 | 0 | 0 | 0.225 | 1 | 20 | 1 | 1 | 0 | 0 | 0 | 1 | 365 | 174 |
| 458 | 852 | 216 | 923 | 10.8 | 0 | 177 | 32.36 | 156 | 0 | 0 | 0 | 0 | 0.166 | 1 | 20 | 1 | 1 | 1 | 13 | 0.2 | 1 | 28 | 154.4 |
| 459 | 900 | 220 | 1005 | 0 | 0 | 163 | 40 | 156 | 0 | 0 | 0 | 0 | 0.146 | 1 | 23 | 1 | 1 | 1 | 13 | 0.2 | 1 | 7 | 124.1 |
| 460 | 900 | 165 | 1021 | 0 | 0 | 163 | 40 | 156 | 55 | 0 | 0 | 0 | 0.153 | 1 | 23 | 1 | 1 | 1 | 13 | 0.2 | 1 | 7 | 115.5 |
| 461 | 900 | 110 | 1036 | 0 | 0 | 163 | 40 | 156 | 110 | 0 | 0 | 0 | 0.161 | 1 | 23 | 1 | 1 | 1 | 13 | 0.2 | 1 | 7 | 113.5 |
| 462 | 810 | 220 | 1002 | 0 | 0 | 163 | 40 | 156 | 90 | 0 | 0 | 0 | 0.158 | 1 | 23 | 1 | 1 | 1 | 13 | 0.2 | 1 | 7 | 116 |
| 463 | 720 | 220 | 998 | 0 | 0 | 163 | 40 | 156 | 180 | 0 | 0 | 0 | 0.173 | 1 | 23 | 1 | 1 | 1 | 13 | 0.2 | 1 | 7 | 116.5 |
| 464 | 630 | 220 | 994 | 0 | 0 | 163 | 40 | 156 | 270 | 0 | 0 | 0 | 0.192 | 1 | 23 | 1 | 1 | 1 | 13 | 0.2 | 1 | 7 | 110 |
| 465 | 900 | 220 | 1005 | 0 | 0 | 163 | 40 | 156 | 0 | 0 | 0 | 0 | 0.146 | 1 | 23 | 1 | 1 | 1 | 13 | 0.2 | 1 | 14 | 132.9 |
| 466 | 900 | 165 | 1021 | 0 | 0 | 163 | 40 | 156 | 55 | 0 | 0 | 0 | 0.153 | 1 | 23 | 1 | 1 | 1 | 13 | 0.2 | 1 | 14 | 123 |
| 467 | 900 | 110 | 1036 | 0 | 0 | 163 | 40 | 156 | 110 | 0 | 0 | 0 | 0.161 | 1 | 23 | 1 | 1 | 1 | 13 | 0.2 | 1 | 14 | 122 |
| 468 | 630 | 90 | 1539 | 0 | 0 | 144 | 18 | 0 | 0 | 180 | 0 | 0 | 0.200 | 1 | 20 | 1 | 1 | 0 | 0 | 0 | 1 | 365 | 178 |
| 469 | 810 | 220 | 1002 | 0 | 0 | 163 | 40 | 156 | 90 | 0 | 0 | 0 | 0.158 | 1 | 23 | 1 | 1 | 1 | 13 | 0.2 | 1 | 14 | 125.3 |
| 470 | 720 | 220 | 998 | 0 | 0 | 163 | 40 | 156 | 180 | 0 | 0 | 0 | 0.173 | 1 | 23 | 1 | 1 | 1 | 13 | 0.2 | 1 | 14 | 125.1 |
| 471 | 630 | 220 | 994 | 0 | 0 | 163 | 40 | 156 | 270 | 0 | 0 | 0 | 0.192 | 1 | 23 | 1 | 1 | 1 | 13 | 0.2 | 1 | 14 | 124.5 |
| 472 | 900 | 220 | 1005 | 0 | 0 | 163 | 40 | 156 | 0 | 0 | 0 | 0 | 0.146 | 1 | 23 | 1 | 1 | 1 | 13 | 0.2 | 1 | 28 | 143 |
| 473 | 900 | 165 | 1021 | 0 | 0 | 163 | 40 | 156 | 55 | 0 | 0 | 0 | 0.153 | 1 | 23 | 1 | 1 | 1 | 13 | 0.2 | 1 | 28 | 138.8 |
| 474 | 900 | 110 | 1036 | 0 | 0 | 163 | 40 | 156 | 110 | 0 | 0 | 0 | 0.161 | 1 | 23 | 1 | 1 | 1 | 13 | 0.2 | 1 | 28 | 138.2 |
| 475 | 810 | 220 | 1002 | 0 | 0 | 163 | 40 | 156 | 90 | 0 | 0 | 0 | 0.158 | 1 | 23 | 1 | 1 | 1 | 13 | 0.2 | 1 | 28 | 140.4 |
| 476 | 720 | 220 | 998 | 0 | 0 | 163 | 40 | 156 | 180 | 0 | 0 | 0 | 0.173 | 1 | 23 | 1 | 1 | 1 | 13 | 0.2 | 1 | 28 | 132.2 |
| 477 | 630 | 220 | 994 | 0 | 0 | 163 | 40 | 156 | 270 | 0 | 0 | 0 | 0.192 | 1 | 23 | 1 | 1 | 1 | 13 | 0.2 | 1 | 28 | 130.6 |
| 478 | 900 | 220 | 1005 | 0 | 0 | 163 | 40 | 156 | 0 | 0 | 0 | 0 | 0.146 | 1 | 23 | 1 | 1 | 1 | 13 | 0.2 | 1 | 56 | 161.6 |
| 479 | 450 | 90 | 1539 | 0 | 0 | 144 | 18 | 0 | 0 | 180 | 0 | 180 | 0.267 | 1 | 20 | 1 | 1 | 0 | 0 | 0 | 1 | 365 | 183 |
| 480 | 900 | 165 | 1021 | 0 | 0 | 163 | 40 | 156 | 55 | 0 | 0 | 0 | 0.153 | 1 | 23 | 1 | 1 | 1 | 13 | 0.2 | 1 | 56 | 154.8 |
| 481 | 900 | 110 | 1036 | 0 | 0 | 163 | 40 | 156 | 110 | 0 | 0 | 0 | 0.161 | 1 | 23 | 1 | 1 | 1 | 13 | 0.2 | 1 | 56 | 152.4 |
| 482 | 810 | 220 | 1002 | 0 | 0 | 163 | 40 | 156 | 90 | 0 | 0 | 0 | 0.158 | 1 | 23 | 1 | 1 | 1 | 13 | 0.2 | 1 | 56 | 157.2 |
| 483 | 720 | 220 | 998 | 0 | 0 | 163 | 40 | 156 | 180 | 0 | 0 | 0 | 0.173 | 1 | 23 | 1 | 1 | 1 | 13 | 0.2 | 1 | 56 | 153.7 |
| 484 | 630 | 220 | 994 | 0 | 0 | 163 | 40 | 156 | 270 | 0 | 0 | 0 | 0.192 | 1 | 23 | 1 | 1 | 1 | 13 | 0.2 | 1 | 56 | 151 |
| 485 | 666.4 | 210.5 | 820.8 | 0 | 259.6 | 164.2 | 17.5 | 156 | 0 | 35.1 | 0 | 0 | 0.187 | 1 | 90 | 1 | 1 | 1 | 13 | 0.2 | 1 | 28 | 144.7 |
| 486 | 631.4 | 210.5 | 820.8 | 0 | 259.6 | 164.2 | 17.5 | 156 | 0 | 70.1 | 0 | 0 | 0.195 | 1 | 90 | 1 | 1 | 1 | 13 | 0.2 | 1 | 28 | 159.1 |
| 487 | 596.3 | 210.5 | 820.8 | 0 | 259.6 | 164.2 | 17.5 | 156 | 0 | 105.2 | 0 | 0 | 0.204 | 1 | 90 | 1 | 1 | 1 | 13 | 0.2 | 1 | 28 | 147.4 |
| 488 | 561.2 | 210.5 | 820.8 | 0 | 259.6 | 164.2 | 17.5 | 156 | 0 | 140.3 | 0 | 0 | 0.213 | 1 | 90 | 1 | 1 | 1 | 13 | 0.2 | 1 | 28 | 139.6 |
| 489 | 526.1 | 210.5 | 820.8 | 0 | 259.6 | 164.2 | 17.5 | 156 | 0 | 175.4 | 0 | 0 | 0.223 | 1 | 90 | 1 | 1 | 1 | 13 | 0.2 | 1 | 28 | 130.6 |
| 490 | 270 | 90 | 1539 | 0 | 0 | 144 | 18 | 0 | 0 | 180 | 0 | 360 | 0.400 | 1 | 20 | 1 | 1 | 0 | 0 | 0 | 1 | 365 | 164 |
| 491 | 701.5 | 210.5 | 820.8 | 0 | 259.6 | 164.2 | 17.5 | 156 | 0 | 0 | 0 | 0 | 0.180 | 1 | 200 | 1 | 1 | 1 | 13 | 0.2 | 1 | 28 | 161.9 |
| 492 | 666.4 | 210.5 | 820.8 | 0 | 259.6 | 164.2 | 17.5 | 156 | 0 | 35.1 | 0 | 0 | 0.187 | 1 | 200 | 1 | 1 | 1 | 13 | 0.2 | 1 | 28 | 170.1 |
| 493 | 631.4 | 210.5 | 820.8 | 0 | 259.6 | 164.2 | 17.5 | 156 | 0 | 70.1 | 0 | 0 | 0.195 | 1 | 200 | 1 | 1 | 1 | 13 | 0.2 | 1 | 28 | 175.9 |
| 494 | 561.2 | 210.5 | 820.8 | 0 | 259.6 | 164.2 | 17.5 | 156 | 0 | 140.3 | 0 | 0 | 0.213 | 1 | 200 | 1 | 1 | 1 | 13 | 0.2 | 1 | 28 | 179.1 |
| 495 | 620.2 | 114.2 | 1277.5 | 0 | 0 | 161.9 | 36 | 156 | 164.9 | 0 | 0 | 0 | 0.220 | 1 | 20 | 1 | 1 | 1 | 13 | 0.2 | 1 | 1 | 91.1 |
| 496 | 558.1 | 114.2 | 1276.6 | 0 | 0 | 161.9 | 36 | 156 | 164.9 | 62 | 0 | 0 | 0.241 | 1 | 20 | 1 | 1 | 1 | 13 | 0.2 | 1 | 1 | 77.9 |
| 497 | 620.2 | 114.2 | 1277.5 | 0 | 0 | 161.9 | 36 | 156 | 164.9 | 0 | 0 | 0 | 0.220 | 1 | 20 | 1 | 1 | 1 | 13 | 0.2 | 1 | 3 | 113.8 |
| 498 | 558.1 | 114.2 | 1276.6 | 0 | 0 | 161.9 | 36 | 156 | 164.9 | 62 | 0 | 0 | 0.241 | 1 | 20 | 1 | 1 | 1 | 13 | 0.2 | 1 | 3 | 102.7 |
| 499 | 620.2 | 114.2 | 1277.5 | 0 | 0 | 161.9 | 36 | 156 | 164.9 | 0 | 0 | 0 | 0.220 | 1 | 20 | 1 | 1 | 1 | 13 | 0.2 | 1 | 7 | 134.2 |
| 500 | 558.1 | 114.2 | 1276.6 | 0 | 0 | 161.9 | 36 | 156 | 164.9 | 62 | 0 | 0 | 0.241 | 1 | 20 | 1 | 1 | 1 | 13 | 0.2 | 1 | 7 | 130.5 |
| 501 | 874.9 | 43.7 | 1273.4 | 0 | 0 | 202.1 | 45.9 | 0 | 0 | 0 | 0 | 0 | 0.220 | 1 | 21 | 1 | 1 | 0 | 0 | 0 | 1 | 7 | 85.91 |
| 502 | 496.1 | 114.2 | 1275.9 | 0 | 0 | 161.9 | 36 | 156 | 164.9 | 124 | 0 | 0 | 0.265 | 1 | 20 | 1 | 1 | 1 | 13 | 0.2 | 1 | 7 | 129.8 |
| 503 | 434.1 | 114.2 | 1275 | 0 | 0 | 161.9 | 36 | 156 | 164.9 | 186 | 0 | 0 | 0.295 | 1 | 20 | 1 | 1 | 1 | 13 | 0.2 | 1 | 7 | 121.7 |
| 504 | 558.1 | 114.2 | 1276.6 | 0 | 0 | 161.9 | 36 | 156 | 164.9 | 62 | 0 | 0 | 0.241 | 1 | 20 | 1 | 1 | 1 | 13 | 0.2 | 1 | 14 | 160.5 |
| 505 | 496.1 | 114.2 | 1275.9 | 0 | 0 | 161.9 | 36 | 156 | 164.9 | 124 | 0 | 0 | 0.265 | 1 | 20 | 1 | 1 | 1 | 13 | 0.2 | 1 | 14 | 162 |
| 506 | 434.1 | 114.2 | 1275 | 0 | 0 | 161.9 | 36 | 156 | 164.9 | 186 | 0 | 0 | 0.295 | 1 | 20 | 1 | 1 | 1 | 13 | 0.2 | 1 | 14 | 153.9 |
| 507 | 558.1 | 114.2 | 1276.6 | 0 | 0 | 161.9 | 36 | 156 | 164.9 | 62 | 0 | 0 | 0.241 | 1 | 20 | 1 | 1 | 1 | 13 | 0.2 | 1 | 28 | 170.7 |
| 508 | 496.1 | 114.2 | 1275.9 | 0 | 0 | 161.9 | 36 | 156 | 164.9 | 124 | 0 | 0 | 0.265 | 1 | 20 | 1 | 1 | 1 | 13 | 0.2 | 1 | 28 | 175.8 |
| 509 | 434.1 | 114.2 | 1275 | 0 | 0 | 161.9 | 36 | 156 | 164.9 | 186 | 0 | 0 | 0.295 | 1 | 20 | 1 | 1 | 1 | 13 | 0.2 | 1 | 28 | 168.4 |
| 510 | 863.2 | 215.8 | 1079 | 0 | 0 | 177 | 21.6 | 0 | 0 | 0 | 0 | 0 | 0.164 | 1 | 20 | 1 | 1 | 0 | 0 | 0 | 1 | 1 | 36.1 |
| 511 | 857.8 | 215.8 | 1079 | 5.4 | 0 | 177 | 21.6 | 0 | 0 | 0 | 0 | 0 | 0.165 | 1 | 20 | 1 | 1 | 0 | 0 | 0 | 1 | 1 | 42.2 |
| 512 | 612.4 | 43.7 | 1273.4 | 0 | 0 | 202.1 | 45.9 | 0 | 0 | 0 | 0 | 262.5 | 0.308 | 1 | 21 | 1 | 1 | 0 | 0 | 0 | 1 | 7 | 78.52 |
| 513 | 852.4 | 215.8 | 1079 | 10.8 | 0 | 177 | 21.6 | 0 | 0 | 0 | 0 | 0 | 0.166 | 1 | 20 | 1 | 1 | 0 | 0 | 0 | 1 | 1 | 47.7 |
| 514 | 847 | 215.8 | 1079 | 16.2 | 0 | 177 | 21.6 | 0 | 0 | 0 | 0 | 0 | 0.167 | 1 | 20 | 1 | 1 | 0 | 0 | 0 | 1 | 1 | 53.1 |
| 515 | 841.6 | 215.8 | 1079 | 21.6 | 0 | 177 | 21.6 | 0 | 0 | 0 | 0 | 0 | 0.167 | 1 | 20 | 1 | 1 | 0 | 0 | 0 | 1 | 1 | 53.1 |
| 516 | 863.2 | 215.8 | 1079 | 0 | 0 | 177 | 21.6 | 0 | 0 | 0 | 0 | 0 | 0.164 | 1 | 20 | 1 | 1 | 0 | 0 | 0 | 1 | 3 | 75.6 |
| 517 | 857.8 | 215.8 | 1079 | 5.4 | 0 | 177 | 21.6 | 0 | 0 | 0 | 0 | 0 | 0.165 | 1 | 20 | 1 | 1 | 0 | 0 | 0 | 1 | 3 | 81.7 |
| 518 | 852.4 | 215.8 | 1079 | 10.8 | 0 | 177 | 21.6 | 0 | 0 | 0 | 0 | 0 | 0.166 | 1 | 20 | 1 | 1 | 0 | 0 | 0 | 1 | 3 | 85.1 |
| 519 | 847 | 215.8 | 1079 | 16.2 | 0 | 177 | 21.6 | 0 | 0 | 0 | 0 | 0 | 0.167 | 1 | 20 | 1 | 1 | 0 | 0 | 0 | 1 | 3 | 87.8 |
| 520 | 841.6 | 215.8 | 1079 | 21.6 | 0 | 177 | 21.6 | 0 | 0 | 0 | 0 | 0 | 0.167 | 1 | 20 | 1 | 1 | 0 | 0 | 0 | 1 | 3 | 81.7 |
| 521 | 863.2 | 215.8 | 1079 | 0 | 0 | 177 | 21.6 | 0 | 0 | 0 | 0 | 0 | 0.164 | 1 | 20 | 1 | 1 | 0 | 0 | 0 | 1 | 7 | 83.7 |
| 522 | 857.8 | 215.8 | 1079 | 5.4 | 0 | 177 | 21.6 | 0 | 0 | 0 | 0 | 0 | 0.165 | 1 | 20 | 1 | 1 | 0 | 0 | 0 | 1 | 7 | 92.6 |
| 523 | 699.9 | 43.7 | 1273.4 | 0 | 175 | 202.1 | 45.9 | 0 | 0 | 0 | 0 | 0 | 0.272 | 1 | 21 | 1 | 1 | 0 | 0 | 0 | 1 | 7 | 85.91 |
| 524 | 852.4 | 215.8 | 1079 | 10.8 | 0 | 177 | 21.6 | 0 | 0 | 0 | 0 | 0 | 0.166 | 1 | 20 | 1 | 1 | 0 | 0 | 0 | 1 | 7 | 102.1 |
| 525 | 847 | 215.8 | 1079 | 16.2 | 0 | 177 | 21.6 | 0 | 0 | 0 | 0 | 0 | 0.167 | 1 | 20 | 1 | 1 | 0 | 0 | 0 | 1 | 7 | 105.5 |
| 526 | 841.6 | 215.8 | 1079 | 21.6 | 0 | 177 | 21.6 | 0 | 0 | 0 | 0 | 0 | 0.167 | 1 | 20 | 1 | 1 | 0 | 0 | 0 | 1 | 7 | 102.8 |
| 527 | 863.2 | 215.8 | 1079 | 0 | 0 | 177 | 21.6 | 0 | 0 | 0 | 0 | 0 | 0.164 | 1 | 20 | 1 | 1 | 0 | 0 | 0 | 1 | 28 | 103.5 |
| 528 | 857.8 | 215.8 | 1079 | 5.4 | 0 | 177 | 21.6 | 0 | 0 | 0 | 0 | 0 | 0.165 | 1 | 20 | 1 | 1 | 0 | 0 | 0 | 1 | 28 | 108.3 |
| 529 | 852.4 | 215.8 | 1079 | 10.8 | 0 | 177 | 21.6 | 0 | 0 | 0 | 0 | 0 | 0.166 | 1 | 20 | 1 | 1 | 0 | 0 | 0 | 1 | 28 | 113.7 |
| 530 | 847 | 215.8 | 1079 | 16.2 | 0 | 177 | 21.6 | 0 | 0 | 0 | 0 | 0 | 0.167 | 1 | 20 | 1 | 1 | 0 | 0 | 0 | 1 | 28 | 112.3 |
| 531 | 841.6 | 215.8 | 1079 | 21.6 | 0 | 177 | 21.6 | 0 | 0 | 0 | 0 | 0 | 0.167 | 1 | 20 | 1 | 1 | 0 | 0 | 0 | 1 | 28 | 110.3 |
| 532 | 863.2 | 215.8 | 1079 | 0 | 0 | 177 | 21.6 | 0 | 0 | 0 | 0 | 0 | 0.164 | 1 | 20 | 1 | 1 | 0 | 0 | 0 | 1 | 90 | 119.8 |
| 533 | 857.8 | 215.8 | 1079 | 5.4 | 0 | 177 | 21.6 | 0 | 0 | 0 | 0 | 0 | 0.165 | 1 | 20 | 1 | 1 | 0 | 0 | 0 | 1 | 90 | 128 |
| 534 | 874.9 | 43.7 | 1273.4 | 0 | 0 | 202.1 | 45.9 | 39 | 0 | 0 | 0 | 0 | 0.220 | 1 | 21 | 1 | 1 | 1 | 13 | 0.2 | 1 | 7 | 106.71 |
| 535 | 852.4 | 215.8 | 1079 | 10.8 | 0 | 177 | 21.6 | 0 | 0 | 0 | 0 | 0 | 0.166 | 1 | 20 | 1 | 1 | 0 | 0 | 0 | 1 | 90 | 130.7 |
| 536 | 847 | 215.8 | 1079 | 16.2 | 0 | 177 | 21.6 | 0 | 0 | 0 | 0 | 0 | 0.167 | 1 | 20 | 1 | 1 | 0 | 0 | 0 | 1 | 90 | 123.2 |
| 537 | 841.6 | 215.8 | 1079 | 21.6 | 0 | 177 | 21.6 | 0 | 0 | 0 | 0 | 0 | 0.167 | 1 | 20 | 1 | 1 | 0 | 0 | 0 | 1 | 90 | 117.8 |
| 538 | 750 | 144 | 990 | 0 | 0 | 182 | 38 | 0 | 200 | 0 | 0 | 0 | 0.204 | 1 | 20 | 1 | 1 | 0 | 0 | 0 | 1 | 3 | 82.3 |
| 539 | 675 | 144 | 990 | 0 | 0 | 182 | 38 | 0 | 200 | 75 | 0 | 0 | 0.222 | 1 | 20 | 1 | 1 | 0 | 0 | 0 | 1 | 3 | 68.9 |
| 540 | 600 | 144 | 990 | 0 | 0 | 182 | 36 | 0 | 200 | 150 | 0 | 0 | 0.245 | 1 | 20 | 1 | 1 | 0 | 0 | 0 | 1 | 3 | 64.5 |
| 541 | 525 | 144 | 990 | 0 | 0 | 182 | 36 | 0 | 200 | 225 | 0 | 0 | 0.272 | 1 | 20 | 1 | 1 | 0 | 0 | 0 | 1 | 3 | 60.7 |
| 542 | 450 | 144 | 990 | 0 | 0 | 182 | 34 | 0 | 200 | 300 | 0 | 0 | 0.306 | 1 | 20 | 1 | 1 | 0 | 0 | 0 | 1 | 3 | 53 |
| 543 | 375 | 144 | 990 | 0 | 0 | 182 | 30 | 0 | 200 | 375 | 0 | 0 | 0.351 | 1 | 20 | 1 | 1 | 0 | 0 | 0 | 1 | 3 | 48.1 |
| 544 | 750 | 144 | 990 | 0 | 0 | 182 | 38 | 0 | 200 | 0 | 0 | 0 | 0.204 | 1 | 20 | 1 | 1 | 0 | 0 | 0 | 1 | 7 | 100.1 |
| 545 | 612.4 | 43.7 | 1273.4 | 0 | 0 | 202.1 | 45.9 | 39 | 0 | 0 | 0 | 262.5 | 0.308 | 1 | 21 | 1 | 1 | 1 | 13 | 0.2 | 1 | 7 | 89.93 |
| 546 | 675 | 144 | 990 | 0 | 0 | 182 | 38 | 0 | 200 | 75 | 0 | 0 | 0.222 | 1 | 20 | 1 | 1 | 0 | 0 | 0 | 1 | 7 | 90 |
| 547 | 600 | 144 | 990 | 0 | 0 | 182 | 36 | 0 | 200 | 150 | 0 | 0 | 0.245 | 1 | 20 | 1 | 1 | 0 | 0 | 0 | 1 | 7 | 84.3 |
| 548 | 525 | 144 | 990 | 0 | 0 | 182 | 36 | 0 | 200 | 225 | 0 | 0 | 0.272 | 1 | 20 | 1 | 1 | 0 | 0 | 0 | 1 | 7 | 82.3 |
| 549 | 450 | 144 | 990 | 0 | 0 | 182 | 34 | 0 | 200 | 300 | 0 | 0 | 0.306 | 1 | 20 | 1 | 1 | 0 | 0 | 0 | 1 | 7 | 78 |
| 550 | 375 | 144 | 990 | 0 | 0 | 182 | 30 | 0 | 200 | 375 | 0 | 0 | 0.351 | 1 | 20 | 1 | 1 | 0 | 0 | 0 | 1 | 7 | 70.3 |
| 551 | 750 | 144 | 990 | 0 | 0 | 182 | 38 | 0 | 200 | 0 | 0 | 0 | 0.204 | 1 | 20 | 1 | 1 | 0 | 0 | 0 | 1 | 28 | 121.8 |
| 552 | 675 | 144 | 990 | 0 | 0 | 182 | 38 | 0 | 200 | 75 | 0 | 0 | 0.222 | 1 | 20 | 1 | 1 | 0 | 0 | 0 | 1 | 28 | 125.2 |
| 553 | 600 | 144 | 990 | 0 | 0 | 182 | 36 | 0 | 200 | 150 | 0 | 0 | 0.245 | 1 | 20 | 1 | 1 | 0 | 0 | 0 | 1 | 28 | 127.6 |
| 554 | 525 | 144 | 990 | 0 | 0 | 182 | 36 | 0 | 200 | 225 | 0 | 0 | 0.272 | 1 | 20 | 1 | 1 | 0 | 0 | 0 | 1 | 28 | 127.1 |
| 555 | 450 | 144 | 990 | 0 | 0 | 182 | 34 | 0 | 200 | 300 | 0 | 0 | 0.306 | 1 | 20 | 1 | 1 | 0 | 0 | 0 | 1 | 28 | 122.3 |
| 556 | 630 | 90 | 1539 | 0 | 0 | 144 | 18 | 0 | 0 | 180 | 0 | 0 | 0.200 | 1 | 20 | 1 | 1 | 0 | 0 | 0 | 1 | 28 | 142 |
| 557 | 699.9 | 43.7 | 1273.4 | 0 | 175 | 202.1 | 45.9 | 39 | 0 | 0 | 0 | 0 | 0.272 | 1 | 21 | 1 | 1 | 1 | 13 | 0.2 | 1 | 7 | 99.33 |
| 558 | 375 | 144 | 990 | 0 | 0 | 182 | 30 | 0 | 200 | 375 | 0 | 0 | 0.351 | 1 | 20 | 1 | 1 | 0 | 0 | 0 | 1 | 28 | 116 |
| 559 | 960 | 240 | 793.7 | 0 | 0 | 234 | 45 | 0 | 0 | 0 | 0 | 0 | 0.195 | 1 | 23 | 1 | 1 | 0 | 0 | 0 | 1 | 7 | 107.6 |
| 560 | 960 | 240 | 787.1 | 0 | 0 | 234 | 45 | 19.5 | 0 | 0 | 0 | 0 | 0.195 | 1 | 23 | 1 | 1 | 1 | 13 | 0.2 | 1 | 7 | 119.3 |
| 561 | 960 | 240 | 780.5 | 0 | 0 | 234 | 45 | 39 | 0 | 0 | 0 | 0 | 0.195 | 1 | 23 | 1 | 1 | 1 | 13 | 0.2 | 1 | 7 | 121.8 |
| 562 | 960 | 240 | 773.8 | 0 | 0 | 234 | 45 | 58.5 | 0 | 0 | 0 | 0 | 0.195 | 1 | 23 | 1 | 1 | 1 | 13 | 0.2 | 1 | 7 | 124.2 |
| 563 | 960 | 240 | 767.2 | 0 | 0 | 234 | 45 | 78 | 0 | 0 | 0 | 0 | 0.195 | 1 | 23 | 1 | 1 | 1 | 13 | 0.2 | 1 | 7 | 126.9 |
| 564 | 960 | 240 | 754 | 0 | 0 | 234 | 45 | 117 | 0 | 0 | 0 | 0 | 0.195 | 1 | 23 | 1 | 1 | 1 | 13 | 0.2 | 1 | 7 | 129.2 |
| 565 | 960 | 240 | 740.7 | 0 | 0 | 234 | 45 | 156 | 0 | 0 | 0 | 0 | 0.195 | 1 | 23 | 1 | 1 | 1 | 13 | 0.2 | 1 | 7 | 136.6 |
| 566 | 960 | 240 | 786.4 | 0 | 0 | 234 | 45 | 19.5 | 0 | 0 | 0 | 0 | 0.195 | 1 | 23 | 1 | 1 | 1 | 13 | 0.2 | 1 | 7 | 118.1 |
| 567 | 960 | 240 | 779.2 | 0 | 0 | 234 | 45 | 39 | 0 | 0 | 0 | 0 | 0.195 | 1 | 23 | 1 | 1 | 1 | 13 | 0.2 | 1 | 7 | 120.2 |
| 568 | 874.9 | 43.7 | 1273.4 | 0 | 0 | 202.1 | 45.9 | 78 | 0 | 0 | 0 | 0 | 0.220 | 1 | 21 | 1 | 1 | 1 | 13 | 0.2 | 1 | 7 | 108.73 |
| 569 | 960 | 240 | 771.9 | 0 | 0 | 234 | 45 | 58.5 | 0 | 0 | 0 | 0 | 0.195 | 1 | 23 | 1 | 1 | 1 | 13 | 0.2 | 1 | 7 | 122.6 |
| 570 | 960 | 240 | 764.7 | 0 | 0 | 234 | 45 | 78 | 0 | 0 | 0 | 0 | 0.195 | 1 | 23 | 1 | 1 | 1 | 13 | 0.2 | 1 | 7 | 127.4 |
| 571 | 960 | 240 | 720.7 | 0 | 0 | 234 | 57 | 117 | 0 | 0 | 0 | 0 | 0.195 | 1 | 23 | 1 | 1 | 1 | 13 | 0.2 | 1 | 7 | 130 |
| 572 | 960 | 240 | 706.2 | 0 | 0 | 234 | 57 | 156 | 0 | 0 | 0 | 0 | 0.195 | 1 | 23 | 1 | 1 | 1 | 13 | 0.2 | 1 | 7 | 132.4 |
| 573 | 960 | 240 | 793.7 | 0 | 0 | 234 | 45 | 0 | 0 | 0 | 0 | 0 | 0.195 | 1 | 23 | 1 | 1 | 0 | 0 | 0 | 1 | 28 | 136.6 |
| 574 | 960 | 240 | 787.1 | 0 | 0 | 234 | 45 | 19.5 | 0 | 0 | 0 | 0 | 0.195 | 1 | 23 | 1 | 1 | 1 | 13 | 0.2 | 1 | 28 | 148.7 |
| 575 | 960 | 240 | 780.5 | 0 | 0 | 234 | 45 | 39 | 0 | 0 | 0 | 0 | 0.195 | 1 | 23 | 1 | 1 | 1 | 13 | 0.2 | 1 | 28 | 150.1 |
| 576 | 960 | 240 | 773.8 | 0 | 0 | 234 | 45 | 58.5 | 0 | 0 | 0 | 0 | 0.195 | 1 | 23 | 1 | 1 | 1 | 13 | 0.2 | 1 | 28 | 152.5 |
| 577 | 960 | 240 | 767.2 | 0 | 0 | 234 | 45 | 78 | 0 | 0 | 0 | 0 | 0.195 | 1 | 23 | 1 | 1 | 1 | 13 | 0.2 | 1 | 28 | 154.8 |
| 578 | 960 | 240 | 754 | 0 | 0 | 234 | 45 | 117 | 0 | 0 | 0 | 0 | 0.195 | 1 | 23 | 1 | 1 | 1 | 13 | 0.2 | 1 | 28 | 160.6 |
| 579 | 612.4 | 43.7 | 1273.4 | 0 | 0 | 202.1 | 45.9 | 78 | 0 | 0 | 0 | 262.5 | 0.308 | 1 | 21 | 1 | 1 | 1 | 13 | 0.2 | 1 | 7 | 92.62 |
| 580 | 960 | 240 | 740.7 | 0 | 0 | 234 | 45 | 156 | 0 | 0 | 0 | 0 | 0.195 | 1 | 23 | 1 | 1 | 1 | 13 | 0.2 | 1 | 28 | 162.8 |
| 581 | 960 | 240 | 786.4 | 0 | 0 | 234 | 45 | 19.5 | 0 | 0 | 0 | 0 | 0.195 | 1 | 23 | 1 | 1 | 1 | 13 | 0.2 | 1 | 28 | 144.5 |
| 582 | 960 | 240 | 779.2 | 0 | 0 | 234 | 45 | 39 | 0 | 0 | 0 | 0 | 0.195 | 1 | 23 | 1 | 1 | 1 | 13 | 0.2 | 1 | 28 | 148.6 |
| 583 | 960 | 240 | 771.9 | 0 | 0 | 234 | 45 | 58.5 | 0 | 0 | 0 | 0 | 0.195 | 1 | 23 | 1 | 1 | 1 | 13 | 0.2 | 1 | 28 | 151.8 |
| 584 | 960 | 240 | 764.7 | 0 | 0 | 234 | 45 | 78 | 0 | 0 | 0 | 0 | 0.195 | 1 | 23 | 1 | 1 | 1 | 13 | 0.2 | 1 | 28 | 154.8 |
| 585 | 960 | 240 | 720.7 | 0 | 0 | 234 | 57 | 117 | 0 | 0 | 0 | 0 | 0.195 | 1 | 23 | 1 | 1 | 1 | 13 | 0.2 | 1 | 28 | 156.2 |
| 586 | 960 | 240 | 706.2 | 0 | 0 | 234 | 57 | 156 | 0 | 0 | 0 | 0 | 0.195 | 1 | 23 | 1 | 1 | 1 | 13 | 0.2 | 1 | 28 | 160.1 |
| 587 | 960 | 240 | 793.7 | 0 | 0 | 234 | 45 | 0 | 0 | 0 | 0 | 0 | 0.195 | 1 | 23 | 1 | 1 | 0 | 0 | 0 | 1 | 56 | 150.1 |
| 588 | 960 | 240 | 787.1 | 0 | 0 | 234 | 45 | 19.5 | 0 | 0 | 0 | 0 | 0.195 | 1 | 23 | 1 | 1 | 1 | 13 | 0.2 | 1 | 56 | 163.1 |
| 589 | 960 | 240 | 780.5 | 0 | 0 | 234 | 45 | 39 | 0 | 0 | 0 | 0 | 0.195 | 1 | 23 | 1 | 1 | 1 | 13 | 0.2 | 1 | 56 | 166.2 |
| 590 | 699.9 | 43.7 | 1273.4 | 0 | 175 | 202.1 | 45.9 | 78 | 0 | 0 | 0 | 0 | 0.272 | 1 | 21 | 1 | 1 | 1 | 13 | 0.2 | 1 | 7 | 102.01 |
| 591 | 960 | 240 | 773.8 | 0 | 0 | 234 | 45 | 58.5 | 0 | 0 | 0 | 0 | 0.195 | 1 | 23 | 1 | 1 | 1 | 13 | 0.2 | 1 | 56 | 167.6 |
| 592 | 960 | 240 | 767.2 | 0 | 0 | 234 | 45 | 78 | 0 | 0 | 0 | 0 | 0.195 | 1 | 23 | 1 | 1 | 1 | 13 | 0.2 | 1 | 56 | 170.7 |
| 593 | 960 | 240 | 754 | 0 | 0 | 234 | 45 | 117 | 0 | 0 | 0 | 0 | 0.195 | 1 | 23 | 1 | 1 | 1 | 13 | 0.2 | 1 | 56 | 172.7 |
| 594 | 960 | 240 | 740.7 | 0 | 0 | 234 | 45 | 156 | 0 | 0 | 0 | 0 | 0.195 | 1 | 23 | 1 | 1 | 1 | 13 | 0.2 | 1 | 56 | 173.2 |
| 595 | 960 | 240 | 786.4 | 0 | 0 | 234 | 45 | 19.5 | 0 | 0 | 0 | 0 | 0.195 | 1 | 23 | 1 | 1 | 1 | 13 | 0.2 | 1 | 56 | 160.9 |
| 596 | 960 | 240 | 779.2 | 0 | 0 | 234 | 45 | 39 | 0 | 0 | 0 | 0 | 0.195 | 1 | 23 | 1 | 1 | 1 | 13 | 0.2 | 1 | 56 | 163.3 |
| 597 | 960 | 240 | 771.9 | 0 | 0 | 234 | 45 | 58.5 | 0 | 0 | 0 | 0 | 0.195 | 1 | 23 | 1 | 1 | 1 | 13 | 0.2 | 1 | 56 | 164.7 |
| 598 | 960 | 240 | 764.7 | 0 | 0 | 234 | 45 | 78 | 0 | 0 | 0 | 0 | 0.195 | 1 | 23 | 1 | 1 | 1 | 13 | 0.2 | 1 | 56 | 164.9 |
| 599 | 960 | 240 | 720.7 | 0 | 0 | 234 | 57 | 117 | 0 | 0 | 0 | 0 | 0.195 | 1 | 23 | 1 | 1 | 1 | 13 | 0.2 | 1 | 56 | 165.5 |
| 600 | 960 | 240 | 706.2 | 0 | 0 | 234 | 57 | 156 | 0 | 0 | 0 | 0 | 0.195 | 1 | 23 | 1 | 1 | 1 | 13 | 0.2 | 1 | 56 | 166.7 |
| 601 | 874.9 | 43.7 | 1273.4 | 0 | 0 | 202.1 | 45.9 | 117 | 0 | 0 | 0 | 0 | 0.220 | 1 | 21 | 1 | 1 | 1 | 13 | 0.2 | 1 | 7 | 110.74 |
| 602 | 960 | 240 | 793.7 | 0 | 0 | 234 | 45 | 0 | 0 | 0 | 0 | 0 | 0.195 | 1 | 23 | 1 | 1 | 0 | 0 | 0 | 1 | 90 | 156.1 |
| 603 | 960 | 240 | 787.1 | 0 | 0 | 234 | 45 | 19.5 | 0 | 0 | 0 | 0 | 0.195 | 1 | 23 | 1 | 1 | 1 | 13 | 0.2 | 1 | 90 | 167.8 |
| 604 | 960 | 240 | 780.5 | 0 | 0 | 234 | 45 | 39 | 0 | 0 | 0 | 0 | 0.195 | 1 | 23 | 1 | 1 | 1 | 13 | 0.2 | 1 | 90 | 169.4 |
| 605 | 960 | 240 | 773.8 | 0 | 0 | 234 | 45 | 58.5 | 0 | 0 | 0 | 0 | 0.195 | 1 | 23 | 1 | 1 | 1 | 13 | 0.2 | 1 | 90 | 170.5 |
| 606 | 960 | 240 | 767.2 | 0 | 0 | 234 | 45 | 78 | 0 | 0 | 0 | 0 | 0.195 | 1 | 23 | 1 | 1 | 1 | 13 | 0.2 | 1 | 90 | 173.6 |
| 607 | 960 | 240 | 754 | 0 | 0 | 234 | 45 | 117 | 0 | 0 | 0 | 0 | 0.195 | 1 | 23 | 1 | 1 | 1 | 13 | 0.2 | 1 | 90 | 176.8 |
| 608 | 960 | 240 | 740.7 | 0 | 0 | 234 | 45 | 156 | 0 | 0 | 0 | 0 | 0.195 | 1 | 23 | 1 | 1 | 1 | 13 | 0.2 | 1 | 90 | 180.3 |
| 609 | 960 | 240 | 786.4 | 0 | 0 | 234 | 45 | 19.5 | 0 | 0 | 0 | 0 | 0.195 | 1 | 23 | 1 | 1 | 1 | 13 | 0.2 | 1 | 90 | 165.1 |
| 610 | 960 | 240 | 779.2 | 0 | 0 | 234 | 45 | 39 | 0 | 0 | 0 | 0 | 0.195 | 1 | 23 | 1 | 1 | 1 | 13 | 0.2 | 1 | 90 | 167.7 |
| 611 | 960 | 240 | 771.9 | 0 | 0 | 234 | 45 | 58.5 | 0 | 0 | 0 | 0 | 0.195 | 1 | 23 | 1 | 1 | 1 | 13 | 0.2 | 1 | 90 | 167.9 |
| 612 | 612.4 | 43.7 | 1273.4 | 0 | 0 | 202.1 | 45.9 | 117 | 0 | 0 | 0 | 262.5 | 0.308 | 1 | 21 | 1 | 1 | 1 | 13 | 0.2 | 1 | 7 | 100 |
| 613 | 960 | 240 | 764.7 | 0 | 0 | 234 | 45 | 78 | 0 | 0 | 0 | 0 | 0.195 | 1 | 23 | 1 | 1 | 1 | 13 | 0.2 | 1 | 90 | 169.1 |
| 614 | 960 | 240 | 720.7 | 0 | 0 | 234 | 57 | 117 | 0 | 0 | 0 | 0 | 0.195 | 1 | 23 | 1 | 1 | 1 | 13 | 0.2 | 1 | 90 | 170.9 |
| 615 | 960 | 240 | 706.2 | 0 | 0 | 234 | 57 | 156 | 0 | 0 | 0 | 0 | 0.195 | 1 | 23 | 1 | 1 | 1 | 13 | 0.2 | 1 | 90 | 172.5 |
| 616 | 960 | 240 | 793.7 | 0 | 0 | 234 | 45 | 0 | 0 | 0 | 0 | 0 | 0.195 | 1 | 23 | 1 | 1 | 0 | 0 | 0 | 1 | 180 | 161.3 |
| 617 | 960 | 240 | 787.1 | 0 | 0 | 234 | 45 | 19.5 | 0 | 0 | 0 | 0 | 0.195 | 1 | 23 | 1 | 1 | 1 | 13 | 0.2 | 1 | 180 | 169.8 |
| 618 | 960 | 240 | 780.5 | 0 | 0 | 234 | 45 | 39 | 0 | 0 | 0 | 0 | 0.195 | 1 | 23 | 1 | 1 | 1 | 13 | 0.2 | 1 | 180 | 170.9 |
| 619 | 960 | 240 | 773.8 | 0 | 0 | 234 | 45 | 58.5 | 0 | 0 | 0 | 0 | 0.195 | 1 | 23 | 1 | 1 | 1 | 13 | 0.2 | 1 | 180 | 172.3 |
| 620 | 960 | 240 | 767.2 | 0 | 0 | 234 | 45 | 78 | 0 | 0 | 0 | 0 | 0.195 | 1 | 23 | 1 | 1 | 1 | 13 | 0.2 | 1 | 180 | 175.2 |
| 621 | 960 | 240 | 754 | 0 | 0 | 234 | 45 | 117 | 0 | 0 | 0 | 0 | 0.195 | 1 | 23 | 1 | 1 | 1 | 13 | 0.2 | 1 | 180 | 178.1 |
| 622 | 960 | 240 | 740.7 | 0 | 0 | 234 | 45 | 156 | 0 | 0 | 0 | 0 | 0.195 | 1 | 23 | 1 | 1 | 1 | 13 | 0.2 | 1 | 180 | 182.4 |
| 623 | 699.9 | 43.7 | 1273.4 | 0 | 175 | 202.1 | 45.9 | 117 | 0 | 0 | 0 | 0 | 0.272 | 1 | 21 | 1 | 1 | 1 | 13 | 0.2 | 1 | 7 | 108.73 |
| 624 | 960 | 240 | 786.4 | 0 | 0 | 234 | 45 | 19.5 | 0 | 0 | 0 | 0 | 0.195 | 1 | 23 | 1 | 1 | 1 | 13 | 0.2 | 1 | 180 | 167.9 |
| 625 | 960 | 240 | 779.2 | 0 | 0 | 234 | 45 | 39 | 0 | 0 | 0 | 0 | 0.195 | 1 | 23 | 1 | 1 | 1 | 13 | 0.2 | 1 | 180 | 169.1 |
| 626 | 960 | 240 | 771.9 | 0 | 0 | 234 | 45 | 58.5 | 0 | 0 | 0 | 0 | 0.195 | 1 | 23 | 1 | 1 | 1 | 13 | 0.2 | 1 | 180 | 170.3 |
| 627 | 960 | 240 | 764.7 | 0 | 0 | 234 | 45 | 78 | 0 | 0 | 0 | 0 | 0.195 | 1 | 23 | 1 | 1 | 1 | 13 | 0.2 | 1 | 180 | 172.5 |
| 628 | 960 | 240 | 720.7 | 0 | 0 | 234 | 57 | 117 | 0 | 0 | 0 | 0 | 0.195 | 1 | 23 | 1 | 1 | 1 | 13 | 0.2 | 1 | 180 | 174.2 |
| 629 | 960 | 240 | 706.2 | 0 | 0 | 234 | 57 | 156 | 0 | 0 | 0 | 0 | 0.195 | 1 | 23 | 1 | 1 | 1 | 13 | 0.2 | 1 | 180 | 177.2 |
| 630 | 472 | 262 | 1049 | 0 | 0 | 178 | 21 | 0 | 0 | 315 | 0 | 0 | 0.243 | 1 | 23 | 1 | 1 | 0 | 0 | 0 | 1 | 7 | 94.5 |
| 631 | 472 | 262 | 1049 | 0 | 0 | 178 | 21 | 156 | 0 | 315 | 0 | 0 | 0.243 | 1 | 23 | 1 | 1 | 1 | 13 | 0.2 | 1 | 7 | 115 |
| 632 | 472 | 262 | 1049 | 0 | 0 | 178 | 21 | 156 | 0 | 315 | 0 | 0 | 0.243 | 1 | 23 | 1 | 1 | 1 | 13 | 0.2 | 1 | 7 | 120.9 |
| 633 | 472 | 262 | 1049 | 0 | 0 | 178 | 21 | 156 | 0 | 315 | 0 | 0 | 0.243 | 1 | 23 | 1 | 1 | 1 | 13 | 0.2 | 1 | 7 | 108.8 |
| 634 | 874.9 | 43.7 | 1273.4 | 0 | 0 | 202.1 | 45.9 | 156 | 0 | 0 | 0 | 0 | 0.220 | 1 | 21 | 1 | 1 | 1 | 13 | 0.2 | 1 | 7 | 120.81 |
| 635 | 472 | 262 | 1049 | 0 | 0 | 178 | 21 | 0 | 0 | 315 | 0 | 0 | 0.243 | 1 | 23 | 1 | 1 | 0 | 0 | 0 | 1 | 28 | 98.3 |
| 636 | 472 | 262 | 1049 | 0 | 0 | 178 | 21 | 156 | 0 | 315 | 0 | 0 | 0.243 | 1 | 23 | 1 | 1 | 1 | 13 | 0.2 | 1 | 28 | 134.3 |
| 637 | 472 | 262 | 1049 | 0 | 0 | 178 | 21 | 156 | 0 | 315 | 0 | 0 | 0.243 | 1 | 23 | 1 | 1 | 1 | 13 | 0.2 | 1 | 28 | 143.5 |
| 638 | 472 | 262 | 1049 | 0 | 0 | 178 | 21 | 156 | 0 | 315 | 0 | 0 | 0.243 | 1 | 23 | 1 | 1 | 1 | 13 | 0.2 | 1 | 28 | 127.6 |
| 639 | 472 | 262 | 1049 | 0 | 0 | 178 | 21 | 156 | 0 | 315 | 0 | 0 | 0.243 | 1 | 23 | 1 | 1 | 1 | 13 | 0.2 | 1 | 28 | 123.4 |
| 640 | 472 | 262 | 1049 | 0 | 0 | 178 | 21 | 156 | 0 | 315 | 0 | 0 | 0.243 | 1 | 23 | 1 | 1 | 1 | 13 | 0.2 | 1 | 28 | 120.5 |
| 641 | 680 | 120 | 1462 | 0 | 0 | 160 | 9.59 | 0 | 0 | 0 | 0 | 0 | 0.200 | 1 | 23 | 1 | 1 | 0 | 0 | 0 | 1 | 7 | 63.7 |
| 642 | 640 | 160 | 1446.4 | 0 | 0 | 160 | 9.6 | 0 | 0 | 0 | 0 | 0 | 0.200 | 1 | 23 | 1 | 1 | 0 | 0 | 0 | 1 | 7 | 57.4 |
| 643 | 600 | 200 | 1434 | 0 | 0 | 160 | 9.6 | 0 | 0 | 0 | 0 | 0 | 0.200 | 1 | 23 | 1 | 1 | 0 | 0 | 0 | 1 | 7 | 60.5 |
| 644 | 680 | 120 | 1502.8 | 0 | 0 | 144 | 9.59 | 0 | 0 | 0 | 0 | 0 | 0.180 | 1 | 23 | 1 | 1 | 0 | 0 | 0 | 1 | 7 | 68.49 |
| 645 | 612.4 | 43.7 | 1273.4 | 0 | 0 | 202.1 | 45.9 | 156 | 0 | 0 | 0 | 262.5 | 0.308 | 1 | 21 | 1 | 1 | 1 | 13 | 0.2 | 1 | 7 | 105.37 |
| 646 | 680 | 120 | 1421.2 | 0 | 0 | 176 | 9.59 | 0 | 0 | 0 | 0 | 0 | 0.220 | 1 | 23 | 1 | 1 | 0 | 0 | 0 | 1 | 7 | 55.32 |
| 647 | 680 | 120 | 1462 | 0 | 0 | 160 | 11.22 | 0 | 0 | 0 | 0 | 0 | 0.200 | 1 | 23 | 1 | 1 | 0 | 0 | 0 | 1 | 7 | 63.22 |
| 648 | 680 | 120 | 1468.8 | 0 | 0 | 160 | 8.02 | 0 | 0 | 0 | 0 | 0 | 0.200 | 1 | 23 | 1 | 1 | 0 | 0 | 0 | 1 | 7 | 58.83 |
| 649 | 765 | 135 | 1315.8 | 0 | 0 | 180 | 10.79 | 0 | 0 | 0 | 0 | 0 | 0.200 | 1 | 23 | 1 | 1 | 0 | 0 | 0 | 1 | 7 | 66.8 |
| 650 | 720 | 180 | 1303.2 | 0 | 0 | 180 | 10.8 | 0 | 0 | 0 | 0 | 0 | 0.200 | 1 | 23 | 1 | 1 | 0 | 0 | 0 | 1 | 7 | 69.2 |
| 651 | 675 | 225 | 1289.25 | 0 | 0 | 180 | 10.8 | 0 | 0 | 0 | 0 | 0 | 0.200 | 1 | 23 | 1 | 1 | 0 | 0 | 0 | 1 | 7 | 64.5 |
| 652 | 720 | 180 | 1353.6 | 0 | 0 | 162 | 10.8 | 0 | 0 | 0 | 0 | 0 | 0.180 | 1 | 23 | 1 | 1 | 0 | 0 | 0 | 1 | 7 | 72 |
| 653 | 720 | 180 | 1260 | 0 | 0 | 198 | 10.8 | 0 | 0 | 0 | 0 | 0 | 0.220 | 1 | 23 | 1 | 1 | 0 | 0 | 0 | 1 | 7 | 64.1 |
| 654 | 720 | 180 | 1296 | 0 | 0 | 180 | 12.6 | 0 | 0 | 0 | 0 | 0 | 0.200 | 1 | 23 | 1 | 1 | 0 | 0 | 0 | 1 | 7 | 70.24 |
| 655 | 720 | 180 | 1310.4 | 0 | 0 | 180 | 9 | 0 | 0 | 0 | 0 | 0 | 0.200 | 1 | 23 | 1 | 1 | 0 | 0 | 0 | 1 | 7 | 67.61 |
| 656 | 699.9 | 43.7 | 1273.4 | 0 | 175 | 202.1 | 45.9 | 156 | 0 | 0 | 0 | 0 | 0.272 | 1 | 21 | 1 | 1 | 1 | 13 | 0.2 | 1 | 7 | 114.77 |
| 657 | 850 | 150 | 1173 | 0 | 0 | 200 | 11.99 | 0 | 0 | 0 | 0 | 0 | 0.200 | 1 | 23 | 1 | 1 | 0 | 0 | 0 | 1 | 7 | 72.3 |
| 658 | 800 | 200 | 1160 | 0 | 0 | 200 | 12 | 0 | 0 | 0 | 0 | 0 | 0.200 | 1 | 23 | 1 | 1 | 0 | 0 | 0 | 1 | 7 | 73.1 |
| 659 | 750 | 250 | 1140 | 0 | 0 | 200 | 12 | 0 | 0 | 0 | 0 | 0 | 0.200 | 1 | 23 | 1 | 1 | 0 | 0 | 0 | 1 | 7 | 67.6 |
| 660 | 850 | 150 | 1224 | 0 | 0 | 180 | 11.99 | 0 | 0 | 0 | 0 | 0 | 0.180 | 1 | 23 | 1 | 1 | 0 | 0 | 0 | 1 | 7 | 74.63 |
| 661 | 850 | 150 | 1122 | 0 | 0 | 220 | 11.99 | 0 | 0 | 0 | 0 | 0 | 0.220 | 1 | 23 | 1 | 1 | 0 | 0 | 0 | 1 | 7 | 67.61 |
| 662 | 850 | 150 | 1173 | 0 | 0 | 200 | 14.03 | 0 | 0 | 0 | 0 | 0 | 0.200 | 1 | 23 | 1 | 1 | 0 | 0 | 0 | 1 | 7 | 72.88 |
| 663 | 850 | 150 | 1181.5 | 0 | 0 | 200 | 10.03 | 0 | 0 | 0 | 0 | 0 | 0.200 | 1 | 23 | 1 | 1 | 0 | 0 | 0 | 1 | 7 | 69.37 |
| 664 | 680 | 120 | 1462 | 0 | 0 | 160 | 9.59 | 0 | 0 | 0 | 0 | 0 | 0.200 | 1 | 23 | 1 | 1 | 0 | 0 | 0 | 1 | 28 | 118.7 |
| 665 | 640 | 160 | 1446.4 | 0 | 0 | 160 | 9.6 | 0 | 0 | 0 | 0 | 0 | 0.200 | 1 | 23 | 1 | 1 | 0 | 0 | 0 | 1 | 28 | 113.2 |
| 666 | 680 | 120 | 1502.8 | 0 | 0 | 144 | 9.59 | 0 | 0 | 0 | 0 | 0 | 0.180 | 1 | 23 | 1 | 1 | 0 | 0 | 0 | 1 | 28 | 122.93 |
| 667 | 630 | 90 | 1539 | 0 | 0 | 144 | 18 | 0 | 0 | 180 | 0 | 0 | 0.200 | 1 | 20 | 1 | 1 | 0 | 0 | 0 | 1 | 28 | 142 |
| 668 | 612.4 | 43.7 | 1273.4 | 0 | 0 | 202.1 | 45.9 | 195 | 0 | 0 | 0 | 262.5 | 0.308 | 1 | 21 | 1 | 1 | 1 | 13 | 0.2 | 1 | 7 | 114.09 |
| 669 | 680 | 120 | 1421.2 | 0 | 0 | 176 | 9.59 | 0 | 0 | 0 | 0 | 0 | 0.220 | 1 | 23 | 1 | 1 | 0 | 0 | 0 | 1 | 28 | 108.88 |
| 670 | 680 | 120 | 1462 | 0 | 0 | 160 | 11.22 | 0 | 0 | 0 | 0 | 0 | 0.200 | 1 | 23 | 1 | 1 | 0 | 0 | 0 | 1 | 28 | 122.05 |
| 671 | 680 | 120 | 1468.8 | 0 | 0 | 160 | 8.02 | 0 | 0 | 0 | 0 | 0 | 0.200 | 1 | 23 | 1 | 1 | 0 | 0 | 0 | 1 | 28 | 111.51 |
| 672 | 765 | 135 | 1315.8 | 0 | 0 | 180 | 10.79 | 0 | 0 | 0 | 0 | 0 | 0.200 | 1 | 23 | 1 | 1 | 0 | 0 | 0 | 1 | 28 | 122.6 |
| 673 | 720 | 180 | 1303.2 | 0 | 0 | 180 | 10.8 | 0 | 0 | 0 | 0 | 0 | 0.200 | 1 | 23 | 1 | 1 | 0 | 0 | 0 | 1 | 28 | 124.2 |
| 674 | 675 | 225 | 1289.25 | 0 | 0 | 180 | 10.8 | 0 | 0 | 0 | 0 | 0 | 0.200 | 1 | 23 | 1 | 1 | 0 | 0 | 0 | 1 | 28 | 123.4 |
| 675 | 720 | 180 | 1353.6 | 0 | 0 | 162 | 10.8 | 0 | 0 | 0 | 0 | 0 | 0.180 | 1 | 23 | 1 | 1 | 0 | 0 | 0 | 1 | 28 | 128.2 |
| 676 | 720 | 180 | 1260 | 0 | 0 | 198 | 10.8 | 0 | 0 | 0 | 0 | 0 | 0.220 | 1 | 23 | 1 | 1 | 0 | 0 | 0 | 1 | 28 | 122.05 |
| 677 | 720 | 180 | 1296 | 0 | 0 | 180 | 12.6 | 0 | 0 | 0 | 0 | 0 | 0.200 | 1 | 23 | 1 | 1 | 0 | 0 | 0 | 1 | 28 | 127.32 |
| 678 | 720 | 180 | 1310.4 | 0 | 0 | 180 | 9 | 0 | 0 | 0 | 0 | 0 | 0.200 | 1 | 23 | 1 | 1 | 0 | 0 | 0 | 1 | 28 | 125.56 |
| 679 | 699.9 | 43.7 | 1273.4 | 0 | 175 | 202.1 | 45.9 | 195 | 0 | 0 | 0 | 0 | 0.272 | 1 | 21 | 1 | 1 | 1 | 13 | 0.2 | 1 | 7 | 122.82 |
| 680 | 850 | 150 | 1173 | 0 | 0 | 200 | 11.99 | 0 | 0 | 0 | 0 | 0 | 0.200 | 1 | 23 | 1 | 1 | 0 | 0 | 0 | 1 | 28 | 128.1 |
| 681 | 800 | 200 | 1160 | 0 | 0 | 200 | 12 | 0 | 0 | 0 | 0 | 0 | 0.200 | 1 | 23 | 1 | 1 | 0 | 0 | 0 | 1 | 28 | 126.6 |
| 682 | 750 | 250 | 1140 | 0 | 0 | 200 | 12 | 0 | 0 | 0 | 0 | 0 | 0.200 | 1 | 23 | 1 | 1 | 0 | 0 | 0 | 1 | 28 | 125 |
| 683 | 850 | 150 | 1224 | 0 | 0 | 180 | 11.99 | 0 | 0 | 0 | 0 | 0 | 0.180 | 1 | 23 | 1 | 1 | 0 | 0 | 0 | 1 | 28 | 132.59 |
| 684 | 850 | 150 | 1122 | 0 | 0 | 220 | 11.99 | 0 | 0 | 0 | 0 | 0 | 0.220 | 1 | 23 | 1 | 1 | 0 | 0 | 0 | 1 | 28 | 122.93 |
| 685 | 850 | 150 | 1173 | 0 | 0 | 200 | 14.03 | 0 | 0 | 0 | 0 | 0 | 0.200 | 1 | 23 | 1 | 1 | 0 | 0 | 0 | 1 | 28 | 129.95 |
| 686 | 850 | 150 | 1181.5 | 0 | 0 | 200 | 10.03 | 0 | 0 | 0 | 0 | 0 | 0.200 | 1 | 23 | 1 | 1 | 0 | 0 | 0 | 1 | 28 | 127.32 |
| 687 | 729 | 182 | 1150 | 0 | 0 | 182 | 27 | 156 | 0 | 0 | 0 | 0 | 0.200 | 1 | 20 | 1 | 1 | 1 | 13 | 0.2 | 1 | 28 | 148 |
| 688 | 365 | 183 | 1150 | 0 | 0 | 183 | 17 | 156 | 0 | 365 | 0 | 0 | 0.334 | 1 | 20 | 1 | 1 | 1 | 13 | 0.2 | 1 | 28 | 151 |
| 689 | 480 | 171 | 1150 | 0 | 0 | 171 | 33 | 156 | 206 | 0 | 0 | 0 | 0.263 | 1 | 20 | 1 | 1 | 1 | 13 | 0.2 | 1 | 28 | 157 |
| 690 | 874.9 | 43.7 | 1273.4 | 0 | 0 | 202.1 | 45.9 | 0 | 0 | 0 | 0 | 0 | 0.220 | 1 | 21 | 1 | 1 | 0 | 0 | 0 | 1 | 28 | 100.67 |
| 691 | 480 | 171 | 1150 | 0 | 0 | 171 | 33 | 156 | 206 | 0 | 0 | 0 | 0.263 | 1 | 210 | 1 | 1 | 1 | 13 | 0.2 | 1 | 28 | 172 |
| 692 | 863 | 216 | 1079 | 0 | 0 | 177 | 21.6 | 0 | 0 | 0 | 0 | 0 | 0.164 | 1 | 23 | 1 | 1 | 0 | 0 | 0 | 1 | 1 | 35.68 |
| 693 | 858 | 216 | 1079 | 5.4 | 0 | 177 | 21.6 | 0 | 0 | 0 | 0 | 0 | 0.165 | 1 | 23 | 1 | 1 | 0 | 0 | 0 | 1 | 1 | 42.39 |
| 694 | 852 | 216 | 1079 | 10.8 | 0 | 177 | 21.6 | 0 | 0 | 0 | 0 | 0 | 0.166 | 1 | 23 | 1 | 1 | 0 | 0 | 0 | 1 | 1 | 48.2 |
| 695 | 847 | 216 | 1079 | 16.2 | 0 | 177 | 21.6 | 0 | 0 | 0 | 0 | 0 | 0.167 | 1 | 23 | 1 | 1 | 0 | 0 | 0 | 1 | 1 | 53.38 |
| 696 | 842 | 216 | 1079 | 21.6 | 0 | 177 | 21.6 | 0 | 0 | 0 | 0 | 0 | 0.167 | 1 | 23 | 1 | 1 | 0 | 0 | 0 | 1 | 1 | 53.08 |
| 697 | 863 | 216 | 1079 | 0 | 0 | 177 | 21.6 | 0 | 0 | 0 | 0 | 0 | 0.164 | 1 | 23 | 1 | 1 | 0 | 0 | 0 | 1 | 3 | 75.05 |
| 698 | 858 | 216 | 1079 | 5.4 | 0 | 177 | 21.6 | 0 | 0 | 0 | 0 | 0 | 0.165 | 1 | 23 | 1 | 1 | 0 | 0 | 0 | 1 | 3 | 82.07 |
| 699 | 852 | 216 | 1079 | 10.8 | 0 | 177 | 21.6 | 0 | 0 | 0 | 0 | 0 | 0.166 | 1 | 23 | 1 | 1 | 0 | 0 | 0 | 1 | 3 | 85.12 |
| 700 | 847 | 216 | 1079 | 16.2 | 0 | 177 | 21.6 | 0 | 0 | 0 | 0 | 0 | 0.167 | 1 | 23 | 1 | 1 | 0 | 0 | 0 | 1 | 3 | 87.88 |
| 701 | 612.4 | 43.7 | 1273.4 | 0 | 0 | 202.1 | 45.9 | 0 | 0 | 0 | 0 | 262.5 | 0.308 | 1 | 21 | 1 | 1 | 0 | 0 | 0 | 1 | 28 | 88.59 |
| 702 | 842 | 216 | 1079 | 21.6 | 0 | 177 | 21.6 | 0 | 0 | 0 | 0 | 0 | 0.167 | 1 | 23 | 1 | 1 | 0 | 0 | 0 | 1 | 3 | 82.09 |
| 703 | 863 | 216 | 1079 | 0 | 0 | 177 | 21.6 | 0 | 0 | 0 | 0 | 0 | 0.164 | 1 | 23 | 1 | 1 | 0 | 0 | 0 | 1 | 7 | 92.48 |
| 704 | 858 | 216 | 1079 | 5.4 | 0 | 177 | 21.6 | 0 | 0 | 0 | 0 | 0 | 0.165 | 1 | 23 | 1 | 1 | 0 | 0 | 0 | 1 | 7 | 92.79 |
| 705 | 852 | 216 | 1079 | 10.8 | 0 | 177 | 21.6 | 0 | 0 | 0 | 0 | 0 | 0.166 | 1 | 23 | 1 | 1 | 0 | 0 | 0 | 1 | 7 | 101.64 |
| 706 | 847 | 216 | 1079 | 16.2 | 0 | 177 | 21.6 | 0 | 0 | 0 | 0 | 0 | 0.167 | 1 | 23 | 1 | 1 | 0 | 0 | 0 | 1 | 7 | 106.22 |
| 707 | 842 | 216 | 1079 | 21.6 | 0 | 177 | 21.6 | 0 | 0 | 0 | 0 | 0 | 0.167 | 1 | 23 | 1 | 1 | 0 | 0 | 0 | 1 | 7 | 103.18 |
| 708 | 863 | 216 | 1079 | 0 | 0 | 177 | 21.6 | 0 | 0 | 0 | 0 | 0 | 0.164 | 1 | 23 | 1 | 1 | 0 | 0 | 0 | 1 | 28 | 103.81 |
| 709 | 858 | 216 | 1079 | 5.4 | 0 | 177 | 21.6 | 0 | 0 | 0 | 0 | 0 | 0.165 | 1 | 23 | 1 | 1 | 0 | 0 | 0 | 1 | 28 | 108.09 |
| 710 | 852 | 216 | 1079 | 10.8 | 0 | 177 | 21.6 | 0 | 0 | 0 | 0 | 0 | 0.166 | 1 | 23 | 1 | 1 | 0 | 0 | 0 | 1 | 28 | 113.58 |
| 711 | 847 | 216 | 1079 | 16.2 | 0 | 177 | 21.6 | 0 | 0 | 0 | 0 | 0 | 0.167 | 1 | 23 | 1 | 1 | 0 | 0 | 0 | 1 | 28 | 112.68 |
| 712 | 874.9 | 43.7 | 1273.4 | 0 | 0 | 202.1 | 45.9 | 39 | 0 | 0 | 0 | 0 | 0.220 | 1 | 21 | 1 | 1 | 1 | 13 | 0.2 | 1 | 28 | 115.44 |
| 713 | 842 | 216 | 1079 | 21.6 | 0 | 177 | 21.6 | 0 | 0 | 0 | 0 | 0 | 0.167 | 1 | 23 | 1 | 1 | 0 | 0 | 0 | 1 | 28 | 110.25 |
| 714 | 863 | 216 | 1079 | 0 | 0 | 177 | 21.6 | 0 | 0 | 0 | 0 | 0 | 0.164 | 1 | 23 | 1 | 1 | 0 | 0 | 0 | 1 | 28 | 119.41 |
| 715 | 858 | 216 | 1079 | 5.4 | 0 | 177 | 21.6 | 0 | 0 | 0 | 0 | 0 | 0.165 | 1 | 23 | 1 | 1 | 0 | 0 | 0 | 1 | 28 | 128.26 |
| 716 | 852 | 216 | 1079 | 10.8 | 0 | 177 | 21.6 | 0 | 0 | 0 | 0 | 0 | 0.166 | 1 | 23 | 1 | 1 | 0 | 0 | 0 | 1 | 28 | 129.79 |
| 717 | 847 | 216 | 1079 | 16.2 | 0 | 177 | 21.6 | 0 | 0 | 0 | 0 | 0 | 0.167 | 1 | 23 | 1 | 1 | 0 | 0 | 0 | 1 | 28 | 124 |
| 718 | 842 | 216 | 1079 | 21.6 | 0 | 177 | 21.6 | 0 | 0 | 0 | 0 | 0 | 0.167 | 1 | 23 | 1 | 1 | 0 | 0 | 0 | 1 | 28 | 117.62 |
| 719 | 1251.2 | 291.3 | 407.8 | 0 | 0 | 201.4 | 28.2 | 0 | 0 | 0 | 0 | 0 | 0.131 | 1 | 23 | 1 | 1 | 0 | 0 | 0 | 1 | 1 | 82.81 |
| 720 | 825.8 | 192.3 | 444.4 | 0 | 0 | 201.4 | 10.5 | 0 | 0 | 0 | 0 | 486.2 | 0.198 | 1 | 23 | 1 | 1 | 0 | 0 | 0 | 1 | 1 | 70.14 |
| 721 | 575.6 | 134 | 455.7 | 0 | 0 | 201.4 | 5.1 | 0 | 0 | 0 | 0 | 772.2 | 0.284 | 1 | 23 | 1 | 1 | 0 | 0 | 0 | 1 | 1 | 33.49 |
| 722 | 325.3 | 75.7 | 455.4 | 0 | 0 | 201.4 | 5.2 | 0 | 0 | 0 | 0 | 1058.2 | 0.502 | 1 | 23 | 1 | 1 | 0 | 0 | 0 | 1 | 1 | 28.51 |
| 723 | 612.4 | 43.7 | 1273.4 | 0 | 0 | 202.1 | 45.9 | 39 | 0 | 0 | 0 | 262.5 | 0.308 | 1 | 21 | 1 | 1 | 1 | 13 | 0.2 | 1 | 28 | 100.67 |
| 724 | 1251.2 | 291.3 | 407.8 | 0 | 0 | 201.4 | 28.2 | 0 | 0 | 0 | 0 | 0 | 0.131 | 1 | 23 | 1 | 1 | 0 | 0 | 0 | 1 | 3 | 112.692 |
| 725 | 825.8 | 192.3 | 444.4 | 0 | 0 | 201.4 | 10.5 | 0 | 0 | 0 | 0 | 486.2 | 0.198 | 1 | 23 | 1 | 1 | 0 | 0 | 0 | 1 | 3 | 116.31 |
| 726 | 325.3 | 75.7 | 455.4 | 0 | 0 | 201.4 | 5.2 | 0 | 0 | 0 | 0 | 1058.2 | 0.502 | 1 | 23 | 1 | 1 | 0 | 0 | 0 | 1 | 3 | 54.32 |
| 727 | 1251.2 | 291.3 | 407.8 | 0 | 0 | 201.4 | 28.2 | 0 | 0 | 0 | 0 | 0 | 0.131 | 1 | 23 | 1 | 1 | 0 | 0 | 0 | 1 | 7 | 124.94 |
| 728 | 325.3 | 75.7 | 455.4 | 0 | 0 | 201.4 | 5.2 | 0 | 0 | 0 | 0 | 1058.2 | 0.502 | 1 | 23 | 1 | 1 | 0 | 0 | 0 | 1 | 7 | 75.62 |
| 729 | 1251.2 | 291.3 | 407.8 | 0 | 0 | 201.4 | 28.2 | 0 | 0 | 0 | 0 | 0 | 0.131 | 1 | 23 | 1 | 1 | 0 | 0 | 0 | 1 | 14 | 140.83 |
| 730 | 825.8 | 192.3 | 444.4 | 0 | 0 | 201.4 | 10.5 | 0 | 0 | 0 | 0 | 486.2 | 0.198 | 1 | 23 | 1 | 1 | 0 | 0 | 0 | 1 | 14 | 148.52 |
| 731 | 575.6 | 134 | 455.7 | 0 | 0 | 201.4 | 5.1 | 0 | 0 | 0 | 0 | 772.2 | 0.284 | 1 | 23 | 1 | 1 | 0 | 0 | 0 | 1 | 14 | 142.64 |
| 732 | 325.3 | 75.7 | 455.4 | 0 | 0 | 201.4 | 5.2 | 0 | 0 | 0 | 0 | 1058.2 | 0.502 | 1 | 23 | 1 | 1 | 0 | 0 | 0 | 1 | 14 | 97.84 |
| 733 | 1251.2 | 291.3 | 407.8 | 0 | 0 | 201.4 | 28.2 | 0 | 0 | 0 | 0 | 0 | 0.131 | 1 | 23 | 1 | 1 | 0 | 0 | 0 | 1 | 28 | 147.27 |
| 734 | 699.9 | 43.7 | 1273.4 | 0 | 175 | 202.1 | 45.9 | 39 | 0 | 0 | 0 | 0 | 0.272 | 1 | 21 | 1 | 1 | 1 | 13 | 0.2 | 1 | 28 | 106.71 |
| 735 | 825.8 | 192.3 | 444.4 | 0 | 0 | 201.4 | 10.5 | 0 | 0 | 0 | 0 | 486.2 | 0.198 | 1 | 23 | 1 | 1 | 0 | 0 | 0 | 1 | 28 | 167.18 |
| 736 | 575.6 | 134 | 455.7 | 0 | 0 | 201.4 | 5.1 | 0 | 0 | 0 | 0 | 772.2 | 0.284 | 1 | 23 | 1 | 1 | 0 | 0 | 0 | 1 | 28 | 155.87 |
| 737 | 325.3 | 75.7 | 455.4 | 0 | 0 | 201.4 | 5.2 | 0 | 0 | 0 | 0 | 1058.2 | 0.502 | 1 | 23 | 1 | 1 | 0 | 0 | 0 | 1 | 28 | 113.34 |
| 738 | 1251.2 | 291.3 | 407.8 | 0 | 0 | 201.4 | 28.2 | 0 | 0 | 0 | 0 | 0 | 0.131 | 1 | 23 | 1 | 1 | 0 | 0 | 0 | 1 | 56 | 156.53 |
| 739 | 575.6 | 134 | 455.7 | 0 | 0 | 201.4 | 5.1 | 0 | 0 | 0 | 0 | 772.2 | 0.284 | 1 | 23 | 1 | 1 | 0 | 0 | 0 | 1 | 56 | 171 |
| 740 | 325.3 | 75.7 | 455.4 | 0 | 0 | 201.4 | 5.2 | 0 | 0 | 0 | 0 | 1058.2 | 0.502 | 1 | 23 | 1 | 1 | 0 | 0 | 0 | 1 | 56 | 123.05 |
| 741 | 788.5 | 433.7 | 867.4 | 0 | 0 | 160.3 | 52.6 | 0 | 0 | 0 | 0 | 0 | 0.131 | 1 | 23 | 1 | 1 | 0 | 0 | 0 | 1 | 28 | 195.2 |
| 742 | 788.5 | 433.7 | 867.4 | 0 | 0 | 160.3 | 52.6 | 156 | 0 | 0 | 0 | 0 | 0.131 | 1 | 23 | 1 | 1 | 1 | 13 | 0.2 | 1 | 28 | 220.2 |
| 743 | 788.5 | 433.7 | 867.4 | 0 | 0 | 160.3 | 52.6 | 156 | 0 | 0 | 0 | 0 | 0.131 | 1 | 23 | 1 | 1 | 1 | 13 | 0.2 | 1 | 28 | 209.6 |
| 744 | 788.5 | 433.7 | 867.4 | 0 | 0 | 160.3 | 52.6 | 156 | 0 | 0 | 0 | 0 | 0.131 | 1 | 23 | 1 | 1 | 1 | 13 | 0.2 | 1 | 28 | 201.4 |
| 745 | 874.9 | 43.7 | 1273.4 | 0 | 0 | 202.1 | 45.9 | 78 | 0 | 0 | 0 | 0 | 0.220 | 1 | 21 | 1 | 1 | 1 | 13 | 0.2 | 1 | 28 | 124.83 |
| 746 | 788.5 | 433.7 | 867.4 | 0 | 0 | 160.3 | 52.6 | 156 | 0 | 0 | 0 | 0 | 0.131 | 1 | 23 | 1 | 1 | 1 | 13 | 0.2 | 1 | 28 | 218.4 |
| 747 | 788.5 | 433.7 | 867.4 | 0 | 0 | 160.3 | 52.6 | 156 | 0 | 0 | 0 | 0 | 0.131 | 1 | 23 | 1 | 1 | 1 | 13 | 0.2 | 1 | 28 | 218.5 |
| 748 | 788.5 | 433.7 | 867.4 | 0 | 0 | 160.3 | 52.6 | 156 | 0 | 0 | 0 | 0 | 0.131 | 1 | 23 | 1 | 1 | 1 | 13 | 0.2 | 1 | 28 | 205.1 |
| 749 | 788.5 | 433.7 | 867.4 | 0 | 0 | 160.3 | 52.6 | 39 | 0 | 0 | 0 | 0 | 0.131 | 1 | 23 | 1 | 1 | 1 | 13 | 0.2 | 1 | 28 | 209.4 |
| 750 | 788.5 | 433.7 | 867.4 | 0 | 0 | 160.3 | 52.6 | 78 | 0 | 0 | 0 | 0 | 0.131 | 1 | 23 | 1 | 1 | 1 | 13 | 0.2 | 1 | 28 | 199.6 |
| 751 | 788.5 | 433.7 | 867.4 | 0 | 0 | 160.3 | 52.6 | 117 | 0 | 0 | 0 | 0 | 0.131 | 1 | 23 | 1 | 1 | 1 | 13 | 0.2 | 1 | 28 | 209.7 |
| 752 | 788.5 | 433.7 | 867.4 | 0 | 0 | 160.3 | 52.6 | 39 | 0 | 0 | 0 | 0 | 0.131 | 1 | 23 | 1 | 1 | 1 | 13 | 0.2 | 1 | 28 | 198.6 |
| 753 | 788.5 | 433.7 | 867.4 | 0 | 0 | 160.3 | 52.6 | 78 | 0 | 0 | 0 | 0 | 0.131 | 1 | 23 | 1 | 1 | 1 | 13 | 0.2 | 1 | 28 | 208.4 |
| 754 | 788.5 | 433.7 | 867.4 | 0 | 0 | 160.3 | 52.6 | 117 | 0 | 0 | 0 | 0 | 0.131 | 1 | 23 | 1 | 1 | 1 | 13 | 0.2 | 1 | 28 | 220.5 |
| 755 | 788.5 | 433.7 | 867.4 | 0 | 0 | 160.3 | 52.6 | 39 | 0 | 0 | 0 | 0 | 0.131 | 1 | 23 | 1 | 1 | 1 | 13 | 0.2 | 1 | 28 | 211.2 |
| 756 | 612.4 | 43.7 | 1273.4 | 0 | 0 | 202.1 | 45.9 | 78 | 0 | 0 | 0 | 262.5 | 0.308 | 1 | 21 | 1 | 1 | 1 | 13 | 0.2 | 1 | 28 | 114.09 |
| 757 | 788.5 | 433.7 | 867.4 | 0 | 0 | 160.3 | 52.6 | 78 | 0 | 0 | 0 | 0 | 0.131 | 1 | 23 | 1 | 1 | 1 | 13 | 0.2 | 1 | 28 | 186.7 |
| 758 | 788.5 | 433.7 | 867.4 | 0 | 0 | 160.3 | 52.6 | 117 | 0 | 0 | 0 | 0 | 0.131 | 1 | 23 | 1 | 1 | 1 | 13 | 0.2 | 1 | 28 | 218.1 |
| 759 | 946 | 71 | 860 | 0 | 0 | 166 | 35.76 | 0 | 166 | 0 | 0 | 47 | 0.163 | 1 | 23 | 1 | 1 | 0 | 0 | 0 | 1 | 3 | 120.77 |
| 760 | 784 | 157 | 823 | 0 | 0 | 171 | 39.66 | 0 | 0 | 0 | 0 | 235 | 0.182 | 1 | 23 | 1 | 1 | 0 | 0 | 0 | 1 | 3 | 102.73 |
| 761 | 937 | 70 | 851 | 0 | 0 | 164 | 36.82 | 78 | 164 | 0 | 0 | 47 | 0.163 | 1 | 23 | 1 | 1 | 1 | 13 | 0.2 | 1 | 3 | 108.74 |
| 762 | 777 | 155 | 816 | 0 | 0 | 169 | 40.64 | 78 | 0 | 0 | 0 | 233 | 0.181 | 1 | 23 | 1 | 1 | 1 | 13 | 0.2 | 1 | 3 | 101.09 |
| 763 | 946 | 71 | 860 | 0 | 0 | 166 | 35.76 | 0 | 166 | 0 | 0 | 47 | 0.163 | 1 | 23 | 1 | 1 | 0 | 0 | 0 | 1 | 14 | 131.69 |
| 764 | 784 | 157 | 823 | 0 | 0 | 171 | 39.66 | 0 | 0 | 0 | 0 | 235 | 0.182 | 1 | 23 | 1 | 1 | 0 | 0 | 0 | 1 | 14 | 118.03 |
| 765 | 937 | 70 | 851 | 0 | 0 | 164 | 36.82 | 78 | 164 | 0 | 0 | 47 | 0.163 | 1 | 23 | 1 | 1 | 1 | 13 | 0.2 | 1 | 14 | 123.5 |
| 766 | 777 | 155 | 816 | 0 | 0 | 169 | 40.64 | 78 | 0 | 0 | 0 | 233 | 0.181 | 1 | 23 | 1 | 1 | 1 | 13 | 0.2 | 1 | 14 | 110.93 |
| 767 | 699.9 | 43.7 | 1273.4 | 0 | 175 | 202.1 | 45.9 | 78 | 0 | 0 | 0 | 0 | 0.272 | 1 | 21 | 1 | 1 | 1 | 13 | 0.2 | 1 | 28 | 120.81 |
| 768 | 946 | 71 | 860 | 0 | 0 | 166 | 35.76 | 0 | 166 | 0 | 0 | 47 | 0.163 | 1 | 23 | 1 | 1 | 0 | 0 | 0 | 1 | 28 | 144.81 |
| 769 | 784 | 157 | 823 | 0 | 0 | 171 | 39.66 | 0 | 0 | 0 | 0 | 235 | 0.182 | 1 | 23 | 1 | 1 | 0 | 0 | 0 | 1 | 28 | 127.87 |
| 770 | 937 | 70 | 851 | 0 | 0 | 164 | 36.82 | 78 | 164 | 0 | 0 | 47 | 0.163 | 1 | 23 | 1 | 1 | 1 | 13 | 0.2 | 1 | 28 | 150.82 |
| 771 | 777 | 155 | 816 | 0 | 0 | 169 | 40.64 | 78 | 0 | 0 | 0 | 233 | 0.181 | 1 | 23 | 1 | 1 | 1 | 13 | 0.2 | 1 | 28 | 134.43 |
| 772 | 946 | 71 | 860 | 0 | 0 | 166 | 35.76 | 0 | 166 | 0 | 0 | 47 | 0.163 | 1 | 23 | 1 | 1 | 0 | 0 | 0 | 1 | 90 | 160.66 |
| 773 | 784 | 157 | 823 | 0 | 0 | 171 | 39.66 | 0 | 0 | 0 | 0 | 235 | 0.182 | 1 | 23 | 1 | 1 | 0 | 0 | 0 | 1 | 90 | 150.82 |
| 774 | 937 | 70 | 851 | 0 | 0 | 164 | 36.82 | 78 | 164 | 0 | 0 | 47 | 0.163 | 1 | 23 | 1 | 1 | 1 | 13 | 0.2 | 1 | 90 | 166.67 |
| 775 | 777 | 155 | 816 | 0 | 0 | 169 | 40.64 | 78 | 0 | 0 | 0 | 233 | 0.181 | 1 | 23 | 1 | 1 | 1 | 13 | 0.2 | 1 | 90 | 151.91 |
| 776 | 792 | 264 | 1056 | 0 | 0 | 173 | 21.1 | 156 | 0 | 0 | 0 | 0 | 0.164 | 1 | 20 | 1 | 1 | 1 | 13 | 0.2 | 1 | 3 | 102.86 |
| 777 | 784 | 261 | 1045 | 0 | 0 | 171 | 20.9 | 234 | 0 | 0 | 0 | 0 | 0.164 | 1 | 20 | 1 | 1 | 1 | 13 | 0.2 | 1 | 3 | 106 |
| 778 | 450 | 90 | 1539 | 0 | 0 | 144 | 18 | 0 | 0 | 180 | 0 | 180 | 0.267 | 1 | 20 | 1 | 1 | 0 | 0 | 0 | 1 | 28 | 150 |
| 779 | 874.9 | 43.7 | 1273.4 | 0 | 0 | 202.1 | 45.9 | 117 | 0 | 0 | 0 | 0 | 0.220 | 1 | 21 | 1 | 1 | 1 | 13 | 0.2 | 1 | 28 | 132.22 |
| 780 | 809 | 270 | 1079 | 0 | 0 | 177 | 21.6 | 0 | 0 | 0 | 0 | 0 | 0.164 | 1 | 20 | 1 | 1 | 0 | 0 | 0 | 1 | 7 | 92.77 |
| 781 | 800 | 267 | 1067 | 0 | 0 | 175 | 21.3 | 78 | 0 | 0 | 0 | 0 | 0.164 | 1 | 20 | 1 | 1 | 1 | 13 | 0.2 | 1 | 7 | 110.42 |
| 782 | 792 | 264 | 1056 | 0 | 0 | 173 | 21.1 | 156 | 0 | 0 | 0 | 0 | 0.164 | 1 | 20 | 1 | 1 | 1 | 13 | 0.2 | 1 | 7 | 123.66 |
| 783 | 784 | 261 | 1045 | 0 | 0 | 171 | 20.9 | 234 | 0 | 0 | 0 | 0 | 0.164 | 1 | 20 | 1 | 1 | 1 | 13 | 0.2 | 1 | 7 | 128.07 |
| 784 | 809 | 270 | 1079 | 0 | 0 | 177 | 21.6 | 0 | 0 | 0 | 0 | 0 | 0.164 | 1 | 20 | 1 | 1 | 0 | 0 | 0 | 1 | 28 | 106 |
| 785 | 800 | 267 | 1067 | 0 | 0 | 175 | 21.3 | 78 | 0 | 0 | 0 | 0 | 0.164 | 1 | 20 | 1 | 1 | 1 | 13 | 0.2 | 1 | 28 | 129.96 |
| 786 | 792 | 264 | 1056 | 0 | 0 | 173 | 21.1 | 156 | 0 | 0 | 0 | 0 | 0.164 | 1 | 20 | 1 | 1 | 1 | 13 | 0.2 | 1 | 28 | 150.13 |
| 787 | 784 | 261 | 1045 | 0 | 0 | 171 | 20.9 | 234 | 0 | 0 | 0 | 0 | 0.164 | 1 | 20 | 1 | 1 | 1 | 13 | 0.2 | 1 | 28 | 158.32 |
| 788 | 800 | 267 | 1067 | 0 | 0 | 175 | 21.3 | 78 | 0 | 0 | 0 | 0 | 0.164 | 1 | 20 | 1 | 1 | 1 | 13 | 0.2 | 1 | 90 | 148.87 |
| 789 | 792 | 264 | 1056 | 0 | 0 | 173 | 21.1 | 156 | 0 | 0 | 0 | 0 | 0.164 | 1 | 20 | 1 | 1 | 1 | 13 | 0.2 | 1 | 90 | 161.47 |
| 790 | 612.4 | 43.7 | 1273.4 | 0 | 0 | 202.1 | 45.9 | 117 | 0 | 0 | 0 | 262.5 | 0.308 | 1 | 21 | 1 | 1 | 1 | 13 | 0.2 | 1 | 28 | 126.17 |
| 791 | 784 | 261 | 1045 | 0 | 0 | 171 | 20.9 | 234 | 0 | 0 | 0 | 0 | 0.164 | 1 | 20 | 1 | 1 | 1 | 13 | 0.2 | 1 | 90 | 166.51 |
| 792 | 699.9 | 43.7 | 1273.4 | 0 | 175 | 202.1 | 45.9 | 117 | 0 | 0 | 0 | 0 | 0.272 | 1 | 21 | 1 | 1 | 1 | 13 | 0.2 | 1 | 28 | 130.2 |
| 793 | 874.9 | 43.7 | 1273.4 | 0 | 0 | 202.1 | 45.9 | 156 | 0 | 0 | 0 | 0 | 0.220 | 1 | 21 | 1 | 1 | 1 | 13 | 0.2 | 1 | 28 | 146.31 |
| 794 | 612.4 | 43.7 | 1273.4 | 0 | 0 | 202.1 | 45.9 | 156 | 0 | 0 | 0 | 262.5 | 0.308 | 1 | 21 | 1 | 1 | 1 | 13 | 0.2 | 1 | 28 | 134.23 |
| 795 | 699.9 | 43.7 | 1273.4 | 0 | 175 | 202.1 | 45.9 | 156 | 0 | 0 | 0 | 0 | 0.272 | 1 | 21 | 1 | 1 | 1 | 13 | 0.2 | 1 | 28 | 139.6 |
| 796 | 874.9 | 43.7 | 1273.4 | 0 | 0 | 202.1 | 45.9 | 195 | 0 | 0 | 0 | 0 | 0.220 | 1 | 21 | 1 | 1 | 1 | 13 | 0.2 | 1 | 28 | 155.71 |
| 797 | 612.4 | 43.7 | 1273.4 | 0 | 0 | 202.1 | 45.9 | 195 | 0 | 0 | 0 | 262.5 | 0.308 | 1 | 21 | 1 | 1 | 1 | 13 | 0.2 | 1 | 28 | 141.61 |
| 798 | 699.9 | 43.7 | 1273.4 | 0 | 175 | 202.1 | 45.9 | 195 | 0 | 0 | 0 | 0 | 0.272 | 1 | 21 | 1 | 1 | 1 | 13 | 0.2 | 1 | 28 | 148.32 |
| 799 | 729 | 124 | 833 | 0 | 397 | 170 | 30 | 156 | 0 | 0 | 0 | 0 | 0.199 | 1 | 23 | 1 | 1 | 1 | 13 | 0.2 | 1 | 28 | 166.1 |
| 800 | 270 | 90 | 1539 | 0 | 0 | 144 | 18 | 0 | 0 | 180 | 0 | 360 | 0.400 | 1 | 20 | 1 | 1 | 0 | 0 | 0 | 1 | 28 | 130 |
| 801 | 401 | 124 | 833 | 0 | 397 | 170 | 30 | 156 | 328 | 0 | 0 | 0 | 0.324 | 1 | 23 | 1 | 1 | 1 | 13 | 0.2 | 1 | 28 | 124.7 |
| 802 | 401 | 124 | 833 | 0 | 397 | 170 | 30 | 156 | 0 | 328 | 0 | 0 | 0.324 | 1 | 23 | 1 | 1 | 1 | 13 | 0.2 | 1 | 28 | 139.4 |
| 803 | 439.5 | 0 | 1273.4 | 0 | 175.9 | 175.8 | 43.9 | 0 | 0 | 0 | 0 | 263.7 | 0.400 | 1 | 21 | 1 | 1 | 0 | 0 | 0 | 1 | 3 | 49.61 |
| 804 | 435.1 | 0 | 1273.4 | 4.4 | 175.9 | 175.8 | 43.9 | 0 | 0 | 0 | 0 | 263.7 | 0.404 | 1 | 21 | 1 | 1 | 0 | 0 | 0 | 1 | 3 | 50.87 |
| 805 | 430.7 | 0 | 1273.4 | 8.8 | 175.9 | 175.8 | 43.9 | 0 | 0 | 0 | 0 | 263.7 | 0.408 | 1 | 21 | 1 | 1 | 0 | 0 | 0 | 1 | 3 | 54.96 |
| 806 | 426.3 | 0 | 1273.4 | 13.2 | 175.9 | 175.8 | 43.9 | 0 | 0 | 0 | 0 | 263.7 | 0.412 | 1 | 21 | 1 | 1 | 0 | 0 | 0 | 1 | 3 | 56.85 |
| 807 | 421.9 | 0 | 1273.4 | 17.6 | 175.9 | 175.8 | 43.9 | 0 | 0 | 0 | 0 | 263.7 | 0.417 | 1 | 21 | 1 | 1 | 0 | 0 | 0 | 1 | 3 | 57.17 |
| 808 | 417.5 | 0 | 1273.4 | 22 | 175.9 | 175.8 | 43.9 | 0 | 0 | 0 | 0 | 263.7 | 0.421 | 1 | 21 | 1 | 1 | 0 | 0 | 0 | 1 | 3 | 55.91 |
| 809 | 435.1 | 0 | 1273.4 | 4.4 | 175.9 | 175.8 | 43.9 | 195 | 0 | 0 | 0 | 263.7 | 0.404 | 1 | 21 | 1 | 1 | 1 | 13 | 0.2 | 1 | 3 | 70.35 |
| 810 | 430.7 | 0 | 1273.4 | 8.8 | 175.9 | 175.8 | 43.9 | 195 | 0 | 0 | 0 | 263.7 | 0.408 | 1 | 21 | 1 | 1 | 1 | 13 | 0.2 | 1 | 3 | 72.84 |
| 811 | 797.78 | 112.36 | 1123.63 | 0 | 0 | 224.73 | 28.09 | 0 | 44.95 | 112.36 | 56.18 | 0 | 0.247 | 1 | 23 | 1 | 1 | 0 | 0 | 0 | 1 | 1 | 68 |
| 812 | 797.78 | 112.36 | 1123.63 | 0 | 0 | 224.73 | 28.09 | 11.9667 | 44.95 | 112.36 | 56.18 | 0 | 0.247 | 1 | 23 | 1 | 1 | 4 | 18 | 0.013 | 1 | 1 | 66.5 |
| 813 | 922 | 230 | 1152 | 0 | 0 | 184 | 11.52 | 0 | 0 | 0 | 0 | 0 | 0.160 | 1 | 23 | 1 | 1 | 0 | 0 | 0 | 1 | 7 | 109.7 |
| 814 | 922 | 230 | 1152 | 0 | 0 | 184 | 11.52 | 1.844 | 0 | 0 | 0 | 0 | 0.160 | 1 | 23 | 1 | 1 | 4 | 12 | 0.018 | 1 | 7 | 112.8 |
| 815 | 922 | 230 | 1152 | 0 | 0 | 184 | 11.52 | 4.61 | 0 | 0 | 0 | 0 | 0.160 | 1 | 23 | 1 | 1 | 4 | 12 | 0.018 | 1 | 7 | 115 |
| 816 | 922 | 230 | 1152 | 0 | 0 | 184 | 11.52 | 9.22 | 0 | 0 | 0 | 0 | 0.160 | 1 | 23 | 1 | 1 | 4 | 12 | 0.018 | 1 | 7 | 117.9 |
| 817 | 922 | 230 | 1152 | 0 | 0 | 184 | 11.52 | 13.83 | 0 | 0 | 0 | 0 | 0.160 | 1 | 23 | 1 | 1 | 4 | 12 | 0.018 | 1 | 7 | 125.1 |
| 818 | 922 | 230 | 1152 | 0 | 0 | 184 | 11.52 | 16.135 | 0 | 0 | 0 | 0 | 0.160 | 1 | 23 | 1 | 1 | 4 | 12 | 0.018 | 1 | 7 | 119.3 |
| 819 | 922 | 230 | 1152 | 0 | 0 | 184 | 11.52 | 0 | 0 | 0 | 0 | 0 | 0.160 | 1 | 23 | 1 | 1 | 0 | 0 | 0 | 1 | 28 | 156.8 |
| 820 | 922 | 230 | 1152 | 0 | 0 | 184 | 11.52 | 1.844 | 0 | 0 | 0 | 0 | 0.160 | 1 | 23 | 1 | 1 | 4 | 12 | 0.018 | 1 | 28 | 158.9 |
| 821 | 922 | 230 | 1152 | 0 | 0 | 184 | 11.52 | 4.61 | 0 | 0 | 0 | 0 | 0.160 | 1 | 23 | 1 | 1 | 4 | 12 | 0.018 | 1 | 28 | 163.7 |
| 822 | 922 | 230 | 1152 | 0 | 0 | 184 | 11.52 | 9.22 | 0 | 0 | 0 | 0 | 0.160 | 1 | 23 | 1 | 1 | 4 | 12 | 0.018 | 1 | 28 | 167.5 |
| 823 | 797.78 | 112.36 | 1123.63 | 0 | 0 | 224.73 | 28.09 | 11.9667 | 44.95 | 112.36 | 56.18 | 0 | 0.247 | 1 | 23 | 1 | 1 | 4 | 18 | 0.013 | 1 | 14 | 108.5 |
| 824 | 922 | 230 | 1152 | 0 | 0 | 184 | 11.52 | 13.83 | 0 | 0 | 0 | 0 | 0.160 | 1 | 23 | 1 | 1 | 4 | 12 | 0.018 | 1 | 28 | 168.2 |
| 825 | 922 | 230 | 1152 | 0 | 0 | 184 | 11.52 | 16.135 | 0 | 0 | 0 | 0 | 0.160 | 1 | 23 | 1 | 1 | 4 | 12 | 0.018 | 1 | 28 | 163.4 |
| 826 | 863 | 216 | 1079 | 0 | 0 | 194.22 | 43.16 | 0 | 0 | 0 | 0 | 0 | 0.180 | 1 | 23 | 1 | 1 | 0 | 0 | 0 | 1 | 28 | 133.6 |
| 827 | 863 | 216 | 1079 | 0 | 0 | 194.22 | 43.16 | 8.63 | 0 | 0 | 0 | 0 | 0.180 | 1 | 23 | 1 | 1 | 1 | 13 | 0.2 | 1 | 28 | 144.7 |
| 828 | 863 | 216 | 1079 | 0 | 0 | 194.22 | 43.16 | 17.26 | 0 | 0 | 0 | 0 | 0.180 | 1 | 23 | 1 | 1 | 1 | 13 | 0.2 | 1 | 28 | 164.6 |
| 829 | 863 | 216 | 1079 | 0 | 0 | 194.22 | 43.16 | 25.89 | 0 | 0 | 0 | 0 | 0.180 | 1 | 23 | 1 | 1 | 1 | 13 | 0.2 | 1 | 28 | 176.4 |
| 830 | 828 | 207 | 911 | 0 | 248 | 186.4 | 21 | 0 | 0 | 0 | 0 | 0 | 0.180 | 1 | 23 | 1 | 1 | 0 | 0 | 0 | 1 | 3 | 103 |
| 831 | 828 | 207 | 911 | 0 | 248 | 186.4 | 21 | 24.84 | 0 | 0 | 0 | 0 | 0.180 | 1 | 23 | 1 | 1 | 1 | 12 | 0.2 | 1 | 3 | 136.6 |
| 832 | 828 | 207 | 911 | 0 | 248 | 186.4 | 21 | 8.28 | 0 | 0 | 0 | 0 | 0.180 | 1 | 23 | 1 | 1 | 4 | 10 | 0.44 | 1 | 3 | 111.3 |
| 833 | 828 | 207 | 911 | 0 | 248 | 186.4 | 21 | 16.56 | 0 | 0 | 0 | 0 | 0.180 | 1 | 23 | 1 | 1 | 4 | 10 | 0.44 | 1 | 3 | 129 |
| 834 | 797.78 | 112.36 | 1123.63 | 0 | 0 | 224.73 | 28.09 | 11.9667 | 44.95 | 112.36 | 56.18 | 0 | 0.247 | 1 | 23 | 1 | 1 | 4 | 18 | 0.013 | 1 | 28 | 119.6 |
| 835 | 828 | 207 | 911 | 0 | 248 | 186.4 | 21 | 24.84 | 0 | 0 | 0 | 0 | 0.180 | 1 | 23 | 1 | 1 | 4 | 10 | 0.44 | 1 | 3 | 136 |
| 836 | 828 | 207 | 911 | 0 | 248 | 186.4 | 21 | 8.28 | 0 | 0 | 0 | 0 | 0.180 | 1 | 23 | 1 | 1 | 4 | 10 | 0.86 | 1 | 3 | 108.3 |
| 837 | 828 | 207 | 911 | 0 | 248 | 186.4 | 21 | 16.56 | 0 | 0 | 0 | 0 | 0.180 | 1 | 23 | 1 | 1 | 4 | 10 | 0.86 | 1 | 3 | 112.3 |
| 838 | 828 | 207 | 911 | 0 | 248 | 186.4 | 21 | 24.84 | 0 | 0 | 0 | 0 | 0.180 | 1 | 23 | 1 | 1 | 4 | 10 | 0.86 | 1 | 3 | 113.9 |
| 839 | 828 | 207 | 911 | 0 | 248 | 186.4 | 21 | 24.84 | 0 | 0 | 0 | 0 | 0.180 | 1 | 23 | 1 | 1 | 4 | 20 | 0.86 | 1 | 3 | 101.5 |
| 840 | 828 | 207 | 911 | 0 | 248 | 186.4 | 21 | 0 | 0 | 0 | 0 | 0 | 0.180 | 1 | 23 | 1 | 1 | 0 | 0 | 0 | 1 | 7 | 124.1 |
| 841 | 828 | 207 | 911 | 0 | 248 | 186.4 | 21 | 24.84 | 0 | 0 | 0 | 0 | 0.180 | 1 | 23 | 1 | 1 | 1 | 12 | 0.2 | 1 | 7 | 156.8 |
| 842 | 828 | 207 | 911 | 0 | 248 | 186.4 | 21 | 8.28 | 0 | 0 | 0 | 0 | 0.180 | 1 | 23 | 1 | 1 | 4 | 10 | 0.44 | 1 | 7 | 135.1 |
| 843 | 828 | 207 | 911 | 0 | 248 | 186.4 | 21 | 16.56 | 0 | 0 | 0 | 0 | 0.180 | 1 | 23 | 1 | 1 | 4 | 10 | 0.44 | 1 | 7 | 140.1 |
| 844 | 828 | 207 | 911 | 0 | 248 | 186.4 | 21 | 24.84 | 0 | 0 | 0 | 0 | 0.180 | 1 | 23 | 1 | 1 | 4 | 10 | 0.44 | 1 | 7 | 153 |
| 845 | 797.78 | 112.36 | 1123.63 | 0 | 0 | 224.73 | 28.09 | 15.9556 | 44.95 | 112.36 | 56.18 | 0 | 0.247 | 1 | 23 | 1 | 1 | 4 | 18 | 0.013 | 1 | 1 | 66.9 |
| 846 | 828 | 207 | 911 | 0 | 248 | 186.4 | 21 | 8.28 | 0 | 0 | 0 | 0 | 0.180 | 1 | 23 | 1 | 1 | 4 | 10 | 0.86 | 1 | 7 | 121.8 |
| 847 | 828 | 207 | 911 | 0 | 248 | 186.4 | 21 | 16.56 | 0 | 0 | 0 | 0 | 0.180 | 1 | 23 | 1 | 1 | 4 | 10 | 0.86 | 1 | 7 | 123.6 |
| 848 | 828 | 207 | 911 | 0 | 248 | 186.4 | 21 | 24.84 | 0 | 0 | 0 | 0 | 0.180 | 1 | 23 | 1 | 1 | 4 | 10 | 0.86 | 1 | 7 | 125.6 |
| 849 | 828 | 207 | 911 | 0 | 248 | 186.4 | 21 | 24.84 | 0 | 0 | 0 | 0 | 0.180 | 1 | 23 | 1 | 1 | 4 | 20 | 0.86 | 1 | 7 | 119.3 |
| 850 | 828 | 207 | 911 | 0 | 248 | 186.4 | 21 | 0 | 0 | 0 | 0 | 0 | 0.180 | 1 | 23 | 1 | 1 | 0 | 0 | 0 | 1 | 28 | 144.6 |
| 851 | 828 | 207 | 911 | 0 | 248 | 186.4 | 21 | 24.84 | 0 | 0 | 0 | 0 | 0.180 | 1 | 23 | 1 | 1 | 1 | 12 | 0.2 | 1 | 28 | 198.9 |
| 852 | 828 | 207 | 911 | 0 | 248 | 186.4 | 21 | 8.28 | 0 | 0 | 0 | 0 | 0.180 | 1 | 23 | 1 | 1 | 4 | 10 | 0.44 | 1 | 28 | 155.2 |
| 853 | 828 | 207 | 911 | 0 | 248 | 186.4 | 21 | 16.56 | 0 | 0 | 0 | 0 | 0.180 | 1 | 23 | 1 | 1 | 4 | 10 | 0.44 | 1 | 28 | 165.6 |
| 854 | 828 | 207 | 911 | 0 | 248 | 186.4 | 21 | 24.84 | 0 | 0 | 0 | 0 | 0.180 | 1 | 23 | 1 | 1 | 4 | 10 | 0.44 | 1 | 28 | 175.3 |
| 855 | 828 | 207 | 911 | 0 | 248 | 186.4 | 21 | 8.28 | 0 | 0 | 0 | 0 | 0.180 | 1 | 23 | 1 | 1 | 4 | 10 | 0.86 | 1 | 28 | 150.4 |
| 856 | 797.78 | 112.36 | 1123.63 | 0 | 0 | 224.73 | 28.09 | 15.9556 | 44.95 | 112.36 | 56.18 | 0 | 0.247 | 1 | 23 | 1 | 1 | 4 | 18 | 0.013 | 1 | 14 | 108.5 |
| 857 | 828 | 207 | 911 | 0 | 248 | 186.4 | 21 | 16.56 | 0 | 0 | 0 | 0 | 0.180 | 1 | 23 | 1 | 1 | 4 | 10 | 0.86 | 1 | 28 | 152.6 |
| 858 | 828 | 207 | 911 | 0 | 248 | 186.4 | 21 | 24.84 | 0 | 0 | 0 | 0 | 0.180 | 1 | 23 | 1 | 1 | 4 | 10 | 0.86 | 1 | 28 | 155.1 |
| 859 | 828 | 207 | 911 | 0 | 248 | 186.4 | 21 | 24.84 | 0 | 0 | 0 | 0 | 0.180 | 1 | 23 | 1 | 1 | 4 | 20 | 0.86 | 1 | 28 | 148.1 |
| 860 | 646 | 0 | 1076 | 0 | 0 | 172 | 10 | 0 | 0 | 430 | 0 | 0 | 0.266 | 1 | 23 | 1 | 1 | 0 | 0 | 0 | 1 | 28 | 115.3 |
| 861 | 646 | 0 | 1076 | 0 | 0 | 172 | 10 | 3.23 | 0 | 430 | 0 | 0 | 0.266 | 1 | 23 | 1 | 1 | 1 | 13 | 0.2 | 1 | 28 | 118.8 |
| 862 | 646 | 0 | 1076 | 0 | 0 | 172 | 10 | 6.46 | 0 | 430 | 0 | 0 | 0.266 | 1 | 23 | 1 | 1 | 1 | 13 | 0.2 | 1 | 28 | 135.4 |
| 863 | 646 | 0 | 1076 | 0 | 0 | 172 | 10 | 9.69 | 0 | 430 | 0 | 0 | 0.266 | 1 | 23 | 1 | 1 | 1 | 13 | 0.2 | 1 | 28 | 138.1 |
| 864 | 646 | 0 | 1076 | 0 | 0 | 172 | 10 | 12.92 | 0 | 430 | 0 | 0 | 0.266 | 1 | 23 | 1 | 1 | 1 | 13 | 0.2 | 1 | 28 | 140.8 |
| 865 | 646 | 0 | 1076 | 0 | 0 | 172 | 10 | 16.15 | 0 | 430 | 0 | 0 | 0.266 | 1 | 23 | 1 | 1 | 1 | 13 | 0.2 | 1 | 28 | 147.2 |
| 866 | 1000 | 250 | 1000 | 0 | 0 | 200 | 50 | 0 | 0 | 0 | 0 | 0 | 0.160 | 1 | 23 | 1 | 1 | 0 | 0 | 0 | 1 | 28 | 105.5 |
| 867 | 797.78 | 112.36 | 1123.63 | 0 | 0 | 224.73 | 28.09 | 15.9556 | 44.95 | 112.36 | 56.18 | 0 | 0.247 | 1 | 23 | 1 | 1 | 4 | 18 | 0.013 | 1 | 28 | 114.8 |
| 868 | 1000 | 250 | 1000 | 0 | 0 | 200 | 50 | 5 | 0 | 0 | 0 | 0 | 0.160 | 1 | 23 | 1 | 1 | 1 | 12 | 0.2 | 1 | 28 | 114.8 |
| 869 | 1000 | 250 | 1000 | 0 | 0 | 200 | 50 | 10 | 0 | 0 | 0 | 0 | 0.160 | 1 | 23 | 1 | 1 | 1 | 12 | 0.2 | 1 | 28 | 129.5 |
| 870 | 1000 | 250 | 1000 | 0 | 0 | 200 | 50 | 15 | 0 | 0 | 0 | 0 | 0.160 | 1 | 23 | 1 | 1 | 1 | 12 | 0.2 | 1 | 28 | 140 |
| 871 | 1000 | 250 | 1000 | 0 | 0 | 200 | 50 | 5 | 0 | 0 | 0 | 0 | 0.160 | 1 | 23 | 1 | 1 | 4 | 12 | 0.018 | 1 | 28 | 151.8 |
| 872 | 1000 | 250 | 1000 | 0 | 0 | 200 | 50 | 10 | 0 | 0 | 0 | 0 | 0.160 | 1 | 23 | 1 | 1 | 4 | 12 | 0.018 | 1 | 28 | 159.6 |
| 873 | 1000 | 250 | 1000 | 0 | 0 | 200 | 50 | 15 | 0 | 0 | 0 | 0 | 0.160 | 1 | 23 | 1 | 1 | 4 | 12 | 0.018 | 1 | 28 | 148.4 |
| 874 | 900 | 108 | 1000 | 0 | 0 | 161.28 | 18 | 0 | 0 | 0 | 0 | 0 | 0.160 | 1 | 23 | 1 | 1 | 0 | 0 | 0 | 1 | 28 | 151 |
| 875 | 900 | 108 | 1000 | 0 | 0 | 161.28 | 18 | 11.25 | 0 | 0 | 0 | 0 | 0.160 | 1 | 23 | 1 | 1 | 1 | 35 | 0.603448276 | 1 | 28 | 154.1 |
| 876 | 900 | 108 | 1000 | 0 | 0 | 161.28 | 18 | 6.21 | 0 | 0 | 0 | 0 | 0.160 | 1 | 23 | 1 | 1 | 3 | 48 | 0.75 | 1 | 28 | 137.8 |
| 877 | 900 | 108 | 1000 | 0 | 0 | 161.28 | 18 | 4.68 | 0 | 0 | 0 | 0 | 0.160 | 1 | 23 | 1 | 1 | 3 | 48 | 0.75 | 1 | 28 | 138.8 |
| 878 | 797.78 | 112.36 | 1123.63 | 0 | 0 | 224.73 | 28.09 | 19.9445 | 44.95 | 112.36 | 56.18 | 0 | 0.247 | 1 | 23 | 1 | 1 | 4 | 18 | 0.013 | 1 | 1 | 66.3 |
| 879 | 900 | 108 | 1000 | 0 | 0 | 161.28 | 18 | 3.06 | 0 | 0 | 0 | 0 | 0.160 | 1 | 23 | 1 | 1 | 3 | 48 | 0.75 | 1 | 28 | 125.5 |
| 880 | 900 | 108 | 1000 | 0 | 0 | 161.28 | 18 | 6.21 | 0 | 0 | 0 | 0 | 0.160 | 1 | 23 | 1 | 1 | 3 | 54 | 0.054 | 1 | 28 | 132.7 |
| 881 | 900 | 108 | 1000 | 0 | 0 | 161.28 | 18 | 4.68 | 0 | 0 | 0 | 0 | 0.160 | 1 | 23 | 1 | 1 | 3 | 54 | 0.054 | 1 | 28 | 134.7 |
| 882 | 900 | 108 | 1000 | 0 | 0 | 161.28 | 18 | 3.06 | 0 | 0 | 0 | 0 | 0.160 | 1 | 23 | 1 | 1 | 3 | 54 | 0.054 | 1 | 28 | 139.8 |
| 883 | 936 | 140.4 | 1170 | 0 | 0 | 224.64 | 28.08 | 0 | 0 | 0 | 0 | 0 | 0.209 | 1 | 23 | 1 | 1 | 0 | 0 | 0 | 1 | 28 | 140.1 |
| 884 | 936 | 140.4 | 1170 | 0 | 0 | 224.64 | 28.08 | 3.744 | 0 | 0 | 0 | 0 | 0.209 | 1 | 23 | 1 | 1 | 3 | 12 | 0.038 | 1 | 28 | 150.3 |
| 885 | 936 | 140.4 | 1170 | 0 | 0 | 224.64 | 28.08 | 7.488 | 0 | 0 | 0 | 0 | 0.209 | 1 | 23 | 1 | 1 | 3 | 12 | 0.038 | 1 | 28 | 154.8 |
| 886 | 936 | 140.4 | 1170 | 0 | 0 | 224.64 | 28.08 | 11.232 | 0 | 0 | 0 | 0 | 0.209 | 1 | 23 | 1 | 1 | 3 | 12 | 0.038 | 1 | 28 | 160.5 |
| 887 | 936 | 140.4 | 1170 | 0 | 0 | 224.64 | 28.08 | 18.72 | 0 | 0 | 0 | 0 | 0.209 | 1 | 23 | 1 | 1 | 1 | 13 | 0.2 | 1 | 28 | 163.8 |
| 888 | 833 | 167 | 836 | 0 | 363 | 184 | 24 | 0 | 0 | 0 | 0 | 0 | 0.184 | 1 | 23 | 1 | 1 | 0 | 0 | 0 | 1 | 28 | 110.6 |
| 889 | 797.78 | 112.36 | 1123.63 | 0 | 0 | 224.73 | 28.09 | 19.9445 | 44.95 | 112.36 | 56.18 | 0 | 0.247 | 1 | 23 | 1 | 1 | 4 | 18 | 0.013 | 1 | 14 | 107.9 |
| 890 | 833 | 167 | 836 | 0 | 363 | 184 | 24 | 0 | 0 | 0 | 0 | 0 | 0.184 | 1 | 23 | 1 | 1 | 0 | 0 | 0 | 1 | 28 | 103.3 |
| 891 | 833 | 167 | 836 | 0 | 363 | 184 | 24 | 0 | 0 | 0 | 0 | 0 | 0.184 | 1 | 23 | 1 | 1 | 0 | 0 | 0 | 1 | 28 | 97.2 |
| 892 | 833 | 167 | 836 | 0 | 363 | 184 | 24 | 0 | 0 | 0 | 0 | 0 | 0.184 | 1 | 23 | 1 | 1 | 0 | 0 | 0 | 1 | 28 | 94.6 |
| 893 | 833 | 167 | 836 | 0 | 363 | 184 | 24 | 1.666 | 0 | 0 | 0 | 0 | 0.184 | 1 | 23 | 1 | 1 | 3 | 20 | 0.034 | 1 | 28 | 135.7 |
| 894 | 833 | 167 | 836 | 0 | 363 | 184 | 24 | 1.666 | 0 | 0 | 0 | 0 | 0.184 | 1 | 23 | 1 | 1 | 3 | 20 | 0.034 | 1 | 28 | 135.6 |
| 895 | 833 | 167 | 836 | 0 | 363 | 184 | 24 | 1.666 | 0 | 0 | 0 | 0 | 0.184 | 1 | 23 | 1 | 1 | 3 | 20 | 0.034 | 1 | 28 | 118.7 |
| 896 | 833 | 167 | 836 | 0 | 363 | 184 | 24 | 1.666 | 0 | 0 | 0 | 0 | 0.184 | 1 | 23 | 1 | 1 | 3 | 20 | 0.034 | 1 | 28 | 117.6 |
| 897 | 833 | 167 | 836 | 0 | 363 | 184 | 24 | 3.332 | 0 | 0 | 0 | 0 | 0.184 | 1 | 23 | 1 | 1 | 3 | 20 | 0.034 | 1 | 28 | 118.7 |
| 898 | 833 | 167 | 836 | 0 | 363 | 184 | 24 | 3.332 | 0 | 0 | 0 | 0 | 0.184 | 1 | 23 | 1 | 1 | 3 | 20 | 0.034 | 1 | 28 | 114.7 |
| 899 | 833 | 167 | 836 | 0 | 363 | 184 | 24 | 3.332 | 0 | 0 | 0 | 0 | 0.184 | 1 | 23 | 1 | 1 | 3 | 20 | 0.034 | 1 | 28 | 103.4 |
| 900 | 797.78 | 112.36 | 1123.63 | 0 | 0 | 224.73 | 28.09 | 19.9445 | 44.95 | 112.36 | 56.18 | 0 | 0.247 | 1 | 23 | 1 | 1 | 4 | 18 | 0.013 | 1 | 28 | 108.3 |
| 901 | 833 | 167 | 836 | 0 | 363 | 184 | 24 | 3.332 | 0 | 0 | 0 | 0 | 0.184 | 1 | 23 | 1 | 1 | 3 | 20 | 0.034 | 1 | 28 | 91.9 |
| 902 | 833 | 167 | 836 | 0 | 363 | 184 | 24 | 4.998 | 0 | 0 | 0 | 0 | 0.184 | 1 | 23 | 1 | 1 | 3 | 20 | 0.034 | 1 | 28 | 112.3 |
| 903 | 833 | 167 | 836 | 0 | 363 | 184 | 24 | 4.998 | 0 | 0 | 0 | 0 | 0.184 | 1 | 23 | 1 | 1 | 3 | 20 | 0.034 | 1 | 28 | 109.9 |
| 904 | 833 | 167 | 836 | 0 | 363 | 184 | 24 | 4.998 | 0 | 0 | 0 | 0 | 0.184 | 1 | 23 | 1 | 1 | 3 | 20 | 0.034 | 1 | 28 | 102.1 |
| 905 | 833 | 167 | 836 | 0 | 363 | 184 | 24 | 4.998 | 0 | 0 | 0 | 0 | 0.184 | 1 | 23 | 1 | 1 | 3 | 20 | 0.034 | 1 | 28 | 101.1 |
| 906 | 875 | 150 | 1100 | 0 | 0 | 180 | 35 | 6.5625 | 0 | 0 | 0 | 0 | 0.176 | 1 | 23 | 1 | 1 | 4 | 24 | 0.023 | 1 | 28 | 129.8 |
| 907 | 875 | 150 | 1100 | 0 | 0 | 180 | 35 | 6.5625 | 0 | 0 | 0 | 0 | 0.176 | 1 | 23 | 1 | 1 | 4 | 24 | 0.023 | 1 | 28 | 130.8 |
| 908 | 875 | 150 | 1100 | 0 | 0 | 180 | 35 | 6.5625 | 0 | 0 | 0 | 0 | 0.176 | 1 | 23 | 1 | 1 | 4 | 24 | 0.023 | 1 | 28 | 134.9 |
| 909 | 875 | 150 | 1100 | 0 | 0 | 180 | 35 | 8.75 | 0 | 0 | 0 | 0 | 0.176 | 1 | 23 | 1 | 1 | 4 | 24 | 0.023 | 1 | 28 | 130.1 |
| 910 | 875 | 150 | 1100 | 0 | 0 | 180 | 35 | 8.75 | 0 | 0 | 0 | 0 | 0.176 | 1 | 23 | 1 | 1 | 4 | 24 | 0.023 | 1 | 28 | 125.4 |
| 911 | 797.78 | 112.36 | 1123.63 | 0 | 0 | 224.73 | 28.09 | 3.9889 | 44.95 | 112.36 | 56.18 | 0 | 0.247 | 1 | 23 | 1 | 1 | 3 | 10 | 0.023 | 1 | 1 | 72.7 |
| 912 | 875 | 150 | 1100 | 0 | 0 | 180 | 35 | 8.75 | 0 | 0 | 0 | 0 | 0.176 | 1 | 23 | 1 | 1 | 4 | 24 | 0.023 | 1 | 28 | 135.1 |
| 913 | 683.9 | 205.2 | 1025.9 | 0 | 218.8 | 177 | 8 | 0 | 0 | 0 | 0 | 0 | 0.199 | 1 | 23 | 1 | 1 | 0 | 0 | 0 | 1 | 28 | 99.9 |
| 914 | 670.12 | 201 | 1005.18 | 0 | 214.4 | 174.23 | 13.07 | 10.0518 | 0 | 0 | 0 | 0 | 0.200 | 1 | 23 | 1 | 1 | 5 | 10 | 0.01 | 1 | 28 | 106.3 |
| 915 | 665.23 | 199.58 | 997.9 | 0 | 212.88 | 172.97 | 12.97 | 14.63506 | 0 | 0 | 0 | 0 | 0.200 | 1 | 23 | 1 | 1 | 5 | 10 | 0.01 | 1 | 28 | 111.5 |
| 916 | 701.8 | 0 | 1052.7 | 0 | 224.6 | 182.5 | 12.77 | 0 | 0 | 0 | 210.5 | 0 | 0.260 | 1 | 23 | 1 | 1 | 0 | 0 | 0 | 1 | 28 | 95.7 |
| 917 | 687.57 | 0 | 1031 | 0 | 220 | 178.8 | 17.9 | 10.31355 | 0 | 0 | 206.27 | 0 | 0.260 | 1 | 23 | 1 | 1 | 5 | 10 | 0.01 | 1 | 28 | 106.3 |
| 918 | 681.43 | 0 | 1022 | 0 | 218 | 177 | 19.5 | 14.99146 | 0 | 0 | 204.4 | 0 | 0.260 | 1 | 23 | 1 | 1 | 5 | 10 | 0.01 | 1 | 28 | 99.9 |
| 919 | 998.8 | 176.25 | 1010.1 | 0 | 0 | 141 | 75.2 | 0 | 0 | 0 | 0 | 0 | 0.120 | 1 | 23 | 1 | 1 | 0 | 0 | 0 | 1 | 7 | 137.9 |
| 920 | 998.8 | 176.25 | 991.1 | 0 | 0 | 141 | 77.6 | 4.994 | 0 | 0 | 0 | 0 | 0.120 | 1 | 23 | 1 | 1 | 5 | 13 | 0.018 | 1 | 7 | 142.4 |
| 921 | 998.8 | 176.25 | 975 | 0 | 0 | 141 | 78.7 | 9.988 | 0 | 0 | 0 | 0 | 0.120 | 1 | 23 | 1 | 1 | 5 | 13 | 0.018 | 1 | 7 | 146.1 |
| 922 | 797.78 | 112.36 | 1123.63 | 0 | 0 | 224.73 | 28.09 | 0 | 44.95 | 112.36 | 56.18 | 0 | 0.247 | 1 | 23 | 1 | 1 | 0 | 0 | 0 | 1 | 14 | 106.2 |
| 923 | 797.78 | 112.36 | 1123.63 | 0 | 0 | 224.73 | 28.09 | 3.9889 | 44.95 | 112.36 | 56.18 | 0 | 0.247 | 1 | 23 | 1 | 1 | 3 | 10 | 0.023 | 1 | 14 | 108.3 |
| 924 | 998.8 | 176.25 | 954.5 | 0 | 0 | 141 | 81.7 | 14.982 | 0 | 0 | 0 | 0 | 0.120 | 1 | 23 | 1 | 1 | 5 | 13 | 0.018 | 1 | 7 | 151.7 |
| 925 | 998.8 | 176.25 | 942.7 | 0 | 0 | 141 | 82.8 | 19.976 | 0 | 0 | 0 | 0 | 0.120 | 1 | 23 | 1 | 1 | 5 | 13 | 0.018 | 1 | 7 | 151.1 |
| 926 | 998.8 | 176.25 | 920.8 | 0 | 0 | 141 | 84.6 | 24.97 | 0 | 0 | 0 | 0 | 0.120 | 1 | 23 | 1 | 1 | 5 | 13 | 0.018 | 1 | 7 | 151.3 |
| 927 | 998.8 | 176.25 | 898.7 | 0 | 0 | 141 | 88.2 | 29.964 | 0 | 0 | 0 | 0 | 0.120 | 1 | 23 | 1 | 1 | 5 | 13 | 0.018 | 1 | 7 | 151.3 |
| 928 | 998.8 | 176.25 | 1013.9 | 0 | 0 | 164.5 | 49.4 | 0 | 0 | 0 | 0 | 0 | 0.140 | 1 | 23 | 1 | 1 | 0 | 0 | 0 | 1 | 7 | 129 |
| 929 | 998.8 | 176.25 | 998 | 0 | 0 | 164.5 | 49.4 | 4.994 | 0 | 0 | 0 | 0 | 0.140 | 1 | 23 | 1 | 1 | 5 | 13 | 0.018 | 1 | 7 | 131.6 |
| 930 | 998.8 | 176.25 | 981.9 | 0 | 0 | 164.5 | 50.5 | 9.988 | 0 | 0 | 0 | 0 | 0.140 | 1 | 23 | 1 | 1 | 5 | 13 | 0.018 | 1 | 7 | 137.6 |
| 931 | 998.8 | 176.25 | 962.9 | 0 | 0 | 164.5 | 52.9 | 14.982 | 0 | 0 | 0 | 0 | 0.140 | 1 | 23 | 1 | 1 | 5 | 13 | 0.018 | 1 | 7 | 143.5 |
| 932 | 998.8 | 176.25 | 946.8 | 0 | 0 | 164.5 | 54.1 | 19.976 | 0 | 0 | 0 | 0 | 0.140 | 1 | 23 | 1 | 1 | 5 | 13 | 0.018 | 1 | 7 | 143.2 |
| 933 | 998.8 | 176.25 | 930.6 | 0 | 0 | 164.5 | 55.2 | 24.97 | 0 | 0 | 0 | 0 | 0.140 | 1 | 23 | 1 | 1 | 5 | 13 | 0.018 | 1 | 7 | 143.2 |
| 934 | 797.78 | 112.36 | 1123.63 | 0 | 0 | 224.73 | 28.09 | 3.9889 | 44.95 | 112.36 | 56.18 | 0 | 0.247 | 1 | 23 | 1 | 1 | 3 | 10 | 0.023 | 1 | 28 | 126.8 |
| 935 | 998.8 | 176.25 | 900.1 | 0 | 0 | 164.5 | 62.3 | 29.964 | 0 | 0 | 0 | 0 | 0.140 | 1 | 23 | 1 | 1 | 5 | 13 | 0.018 | 1 | 7 | 143 |
| 936 | 998.8 | 176.25 | 1010.1 | 0 | 0 | 141 | 75.2 | 0 | 0 | 0 | 0 | 0 | 0.120 | 1 | 23 | 1 | 1 | 0 | 0 | 0 | 1 | 14 | 153.3 |
| 937 | 998.8 | 176.25 | 991.1 | 0 | 0 | 141 | 77.6 | 4.994 | 0 | 0 | 0 | 0 | 0.120 | 1 | 23 | 1 | 1 | 5 | 13 | 0.018 | 1 | 14 | 157.5 |
| 938 | 998.8 | 176.25 | 975 | 0 | 0 | 141 | 78.7 | 9.988 | 0 | 0 | 0 | 0 | 0.120 | 1 | 23 | 1 | 1 | 5 | 13 | 0.018 | 1 | 14 | 162 |
| 939 | 998.8 | 176.25 | 954.5 | 0 | 0 | 141 | 81.7 | 14.982 | 0 | 0 | 0 | 0 | 0.120 | 1 | 23 | 1 | 1 | 5 | 13 | 0.018 | 1 | 14 | 164.3 |
| 940 | 998.8 | 176.25 | 942.7 | 0 | 0 | 141 | 82.8 | 19.976 | 0 | 0 | 0 | 0 | 0.120 | 1 | 23 | 1 | 1 | 5 | 13 | 0.018 | 1 | 14 | 164.2 |
| 941 | 998.8 | 176.25 | 920.8 | 0 | 0 | 141 | 84.6 | 24.97 | 0 | 0 | 0 | 0 | 0.120 | 1 | 23 | 1 | 1 | 5 | 13 | 0.018 | 1 | 14 | 164.2 |
| 942 | 998.8 | 176.25 | 898.7 | 0 | 0 | 141 | 88.2 | 29.964 | 0 | 0 | 0 | 0 | 0.120 | 1 | 23 | 1 | 1 | 5 | 13 | 0.018 | 1 | 14 | 163.8 |
| 943 | 998.8 | 176.25 | 1013.9 | 0 | 0 | 164.5 | 49.4 | 0 | 0 | 0 | 0 | 0 | 0.140 | 1 | 23 | 1 | 1 | 0 | 0 | 0 | 1 | 14 | 146.1 |
| 944 | 998.8 | 176.25 | 998 | 0 | 0 | 164.5 | 49.4 | 4.994 | 0 | 0 | 0 | 0 | 0.140 | 1 | 23 | 1 | 1 | 5 | 13 | 0.018 | 1 | 14 | 147.5 |
| 945 | 797.78 | 112.36 | 1123.63 | 0 | 0 | 224.73 | 28.09 | 7.9778 | 44.95 | 112.36 | 56.18 | 0 | 0.247 | 1 | 23 | 1 | 1 | 3 | 10 | 0.023 | 1 | 1 | 72.1 |
| 946 | 998.8 | 176.25 | 981.9 | 0 | 0 | 164.5 | 50.5 | 9.988 | 0 | 0 | 0 | 0 | 0.140 | 1 | 23 | 1 | 1 | 5 | 13 | 0.018 | 1 | 14 | 152 |
| 947 | 998.8 | 176.25 | 962.9 | 0 | 0 | 164.5 | 52.9 | 14.982 | 0 | 0 | 0 | 0 | 0.140 | 1 | 23 | 1 | 1 | 5 | 13 | 0.018 | 1 | 14 | 158.7 |
| 948 | 998.8 | 176.25 | 946.8 | 0 | 0 | 164.5 | 54.1 | 19.976 | 0 | 0 | 0 | 0 | 0.140 | 1 | 23 | 1 | 1 | 5 | 13 | 0.018 | 1 | 14 | 158.4 |
| 949 | 998.8 | 176.25 | 930.6 | 0 | 0 | 164.5 | 55.2 | 24.97 | 0 | 0 | 0 | 0 | 0.140 | 1 | 23 | 1 | 1 | 5 | 13 | 0.018 | 1 | 14 | 158.2 |
| 950 | 998.8 | 176.25 | 900.1 | 0 | 0 | 164.5 | 62.3 | 29.964 | 0 | 0 | 0 | 0 | 0.140 | 1 | 23 | 1 | 1 | 5 | 13 | 0.018 | 1 | 14 | 158.2 |
| 951 | 998.8 | 176.25 | 1010.1 | 0 | 0 | 141 | 75.2 | 0 | 0 | 0 | 0 | 0 | 0.120 | 1 | 23 | 1 | 1 | 0 | 0 | 0 | 1 | 28 | 167.6 |
| 952 | 998.8 | 176.25 | 991.1 | 0 | 0 | 141 | 77.6 | 4.994 | 0 | 0 | 0 | 0 | 0.120 | 1 | 23 | 1 | 1 | 5 | 13 | 0.018 | 1 | 28 | 169.1 |
| 953 | 998.8 | 176.25 | 975 | 0 | 0 | 141 | 78.7 | 9.988 | 0 | 0 | 0 | 0 | 0.120 | 1 | 23 | 1 | 1 | 5 | 13 | 0.018 | 1 | 28 | 181 |
| 954 | 998.8 | 176.25 | 954.5 | 0 | 0 | 141 | 81.7 | 14.982 | 0 | 0 | 0 | 0 | 0.120 | 1 | 23 | 1 | 1 | 5 | 13 | 0.018 | 1 | 28 | 184.4 |
| 955 | 998.8 | 176.25 | 942.7 | 0 | 0 | 141 | 82.8 | 19.976 | 0 | 0 | 0 | 0 | 0.120 | 1 | 23 | 1 | 1 | 5 | 13 | 0.018 | 1 | 28 | 183.5 |
| 956 | 797.78 | 112.36 | 1123.63 | 0 | 0 | 224.73 | 28.09 | 7.9778 | 44.95 | 112.36 | 56.18 | 0 | 0.247 | 1 | 23 | 1 | 1 | 3 | 10 | 0.023 | 1 | 14 | 105.3 |
| 957 | 998.8 | 176.25 | 920.8 | 0 | 0 | 141 | 84.6 | 24.97 | 0 | 0 | 0 | 0 | 0.120 | 1 | 23 | 1 | 1 | 5 | 13 | 0.018 | 1 | 28 | 183.5 |
| 958 | 998.8 | 176.25 | 898.7 | 0 | 0 | 141 | 88.2 | 29.964 | 0 | 0 | 0 | 0 | 0.120 | 1 | 23 | 1 | 1 | 5 | 13 | 0.018 | 1 | 28 | 183.4 |
| 959 | 998.8 | 176.25 | 1013.9 | 0 | 0 | 164.5 | 49.4 | 0 | 0 | 0 | 0 | 0 | 0.140 | 1 | 23 | 1 | 1 | 0 | 0 | 0 | 1 | 28 | 162.2 |
| 960 | 998.8 | 176.25 | 998 | 0 | 0 | 164.5 | 49.4 | 4.994 | 0 | 0 | 0 | 0 | 0.140 | 1 | 23 | 1 | 1 | 5 | 13 | 0.018 | 1 | 28 | 164.5 |
| 961 | 998.8 | 176.25 | 981.9 | 0 | 0 | 164.5 | 50.5 | 9.988 | 0 | 0 | 0 | 0 | 0.140 | 1 | 23 | 1 | 1 | 5 | 13 | 0.018 | 1 | 28 | 178.6 |
| 962 | 998.8 | 176.25 | 962.9 | 0 | 0 | 164.5 | 52.9 | 14.982 | 0 | 0 | 0 | 0 | 0.140 | 1 | 23 | 1 | 1 | 5 | 13 | 0.018 | 1 | 28 | 180.1 |
| 963 | 998.8 | 176.25 | 946.8 | 0 | 0 | 164.5 | 54.1 | 19.976 | 0 | 0 | 0 | 0 | 0.140 | 1 | 23 | 1 | 1 | 5 | 13 | 0.018 | 1 | 28 | 180.1 |
| 964 | 998.8 | 176.25 | 930.6 | 0 | 0 | 164.5 | 55.2 | 24.97 | 0 | 0 | 0 | 0 | 0.140 | 1 | 23 | 1 | 1 | 5 | 13 | 0.018 | 1 | 28 | 180.1 |
| 965 | 998.8 | 176.25 | 900.1 | 0 | 0 | 164.5 | 62.3 | 29.964 | 0 | 0 | 0 | 0 | 0.140 | 1 | 23 | 1 | 1 | 5 | 13 | 0.018 | 1 | 28 | 179.9 |
| 966 | 850 | 212.5 | 935 | 0 | 255 | 212.5 | 21.2 | 0 | 0 | 0 | 0 | 0 | 0.200 | 1 | 23 | 1 | 1 | 0 | 0 | 0 | 1 | 28 | 144.6 |
| 967 | 797.78 | 112.36 | 1123.63 | 0 | 0 | 224.73 | 28.09 | 7.9778 | 44.95 | 112.36 | 56.18 | 0 | 0.247 | 1 | 23 | 1 | 1 | 3 | 10 | 0.023 | 1 | 28 | 121 |
| 968 | 850 | 212.5 | 935 | 0 | 255 | 212.5 | 21.2 | 0.85 | 0 | 0 | 0 | 0 | 0.200 | 1 | 23 | 1 | 1 | 4 | 9 | 0.013 | 1 | 28 | 149.8 |
| 969 | 850 | 212.5 | 935 | 0 | 255 | 212.5 | 21.2 | 2.55 | 0 | 0 | 0 | 0 | 0.200 | 1 | 23 | 1 | 1 | 4 | 9 | 0.013 | 1 | 28 | 153.2 |
| 970 | 850 | 212.5 | 935 | 0 | 255 | 212.5 | 21.2 | 4.25 | 0 | 0 | 0 | 0 | 0.200 | 1 | 23 | 1 | 1 | 4 | 9 | 0.013 | 1 | 28 | 141.7 |
| 971 | 850 | 212.5 | 935 | 0 | 255 | 212.5 | 21.2 | 5.95 | 0 | 0 | 0 | 0 | 0.200 | 1 | 23 | 1 | 1 | 4 | 9 | 0.013 | 1 | 28 | 137.1 |
| 972 | 850 | 212.5 | 935 | 0 | 255 | 212.5 | 21.2 | 2.55 | 0 | 0 | 0 | 0 | 0.200 | 1 | 23 | 1 | 1 | 3 | 9 | 0.017 | 1 | 28 | 138.9 |
| 973 | 850 | 212.5 | 935 | 0 | 255 | 212.5 | 21.2 | 25.5 | 0 | 0 | 0 | 0 | 0.200 | 1 | 23 | 1 | 1 | 4 | 10 | 0.4 | 1 | 28 | 148.6 |
| 974 | 850 | 212.5 | 935 | 0 | 255 | 212.5 | 21.2 | 25.5 | 0 | 0 | 0 | 0 | 0.200 | 1 | 23 | 1 | 1 | 3 | 15 | 0.6 | 1 | 28 | 165 |
| 975 | 853 | 170.6 | 1023.6 | 0 | 255.9 | 196.19 | 29.86 | 0 | 0 | 0 | 0 | 0 | 0.192 | 1 | 23 | 1 | 1 | 0 | 0 | 0 | 1 | 7 | 137.9 |
| 976 | 853 | 170.6 | 1023.6 | 0 | 255.9 | 196.19 | 29.86 | 0 | 0 | 0 | 0 | 0 | 0.192 | 1 | 23 | 1 | 1 | 0 | 0 | 0 | 1 | 14 | 149.5 |
| 977 | 853 | 170.6 | 1023.6 | 0 | 255.9 | 196.19 | 29.86 | 0 | 0 | 0 | 0 | 0 | 0.192 | 1 | 23 | 1 | 1 | 0 | 0 | 0 | 1 | 28 | 158.9 |
| 978 | 797.78 | 112.36 | 1123.63 | 0 | 0 | 224.73 | 28.09 | 11.9667 | 44.95 | 112.36 | 56.18 | 0 | 0.247 | 1 | 23 | 1 | 1 | 3 | 10 | 0.023 | 1 | 1 | 70.1 |
| 979 | 853 | 170.6 | 1023.6 | 0 | 255.9 | 196.19 | 29.86 | 0 | 0 | 0 | 0 | 0 | 0.192 | 1 | 23 | 1 | 1 | 0 | 0 | 0 | 1 | 56 | 168.4 |
| 980 | 853 | 170.6 | 1023.6 | 0 | 255.9 | 196.19 | 29.86 | 8.53 | 0 | 0 | 0 | 0 | 0.192 | 1 | 23 | 1 | 1 | 1 | 8 | 0.2 | 1 | 7 | 142.1 |
| 981 | 853 | 170.6 | 1023.6 | 0 | 255.9 | 196.19 | 29.86 | 8.53 | 0 | 0 | 0 | 0 | 0.192 | 1 | 23 | 1 | 1 | 1 | 8 | 0.2 | 1 | 14 | 154.7 |
| 982 | 853 | 170.6 | 1023.6 | 0 | 255.9 | 196.19 | 29.86 | 8.53 | 0 | 0 | 0 | 0 | 0.192 | 1 | 23 | 1 | 1 | 1 | 8 | 0.2 | 1 | 28 | 164.2 |
| 983 | 853 | 170.6 | 1023.6 | 0 | 255.9 | 196.19 | 29.86 | 8.53 | 0 | 0 | 0 | 0 | 0.192 | 1 | 23 | 1 | 1 | 1 | 8 | 0.2 | 1 | 56 | 173.7 |
| 984 | 853 | 170.6 | 1023.6 | 0 | 255.9 | 196.19 | 29.86 | 25.59 | 0 | 0 | 0 | 0 | 0.192 | 1 | 23 | 1 | 1 | 1 | 8 | 0.2 | 1 | 7 | 149.5 |
| 985 | 853 | 170.6 | 1023.6 | 0 | 255.9 | 196.19 | 29.86 | 25.59 | 0 | 0 | 0 | 0 | 0.192 | 1 | 23 | 1 | 1 | 1 | 8 | 0.2 | 1 | 14 | 162.1 |
| 986 | 853 | 170.6 | 1023.6 | 0 | 255.9 | 196.19 | 29.86 | 25.59 | 0 | 0 | 0 | 0 | 0.192 | 1 | 23 | 1 | 1 | 1 | 8 | 0.2 | 1 | 28 | 172.6 |
| 987 | 853 | 170.6 | 1023.6 | 0 | 255.9 | 196.19 | 29.86 | 25.59 | 0 | 0 | 0 | 0 | 0.192 | 1 | 23 | 1 | 1 | 1 | 8 | 0.2 | 1 | 56 | 183.2 |
| 988 | 853 | 170.6 | 1023.6 | 0 | 255.9 | 196.19 | 29.86 | 51.18 | 0 | 0 | 0 | 0 | 0.192 | 1 | 23 | 1 | 1 | 1 | 8 | 0.2 | 1 | 7 | 155.8 |
| 989 | 797.78 | 112.36 | 1123.63 | 0 | 0 | 224.73 | 28.09 | 11.9667 | 44.95 | 112.36 | 56.18 | 0 | 0.247 | 1 | 23 | 1 | 1 | 3 | 10 | 0.023 | 1 | 14 | 106.3 |
| 990 | 853 | 170.6 | 1023.6 | 0 | 255.9 | 196.19 | 29.86 | 51.18 | 0 | 0 | 0 | 0 | 0.192 | 1 | 23 | 1 | 1 | 1 | 8 | 0.2 | 1 | 14 | 168.4 |
| 991 | 853 | 170.6 | 1023.6 | 0 | 255.9 | 196.19 | 29.86 | 51.18 | 0 | 0 | 0 | 0 | 0.192 | 1 | 23 | 1 | 1 | 1 | 8 | 0.2 | 1 | 28 | 180 |
| 992 | 853 | 170.6 | 1023.6 | 0 | 255.9 | 196.19 | 29.86 | 51.18 | 0 | 0 | 0 | 0 | 0.192 | 1 | 23 | 1 | 1 | 1 | 8 | 0.2 | 1 | 56 | 190.5 |
| 993 | 853 | 170.6 | 1023.6 | 0 | 255.9 | 196.19 | 29.86 | 8.53 | 0 | 0 | 0 | 0 | 0.192 | 1 | 23 | 1 | 1 | 1 | 12 | 0.2 | 1 | 7 | 142.1 |
| 994 | 853 | 170.6 | 1023.6 | 0 | 255.9 | 196.19 | 29.86 | 8.53 | 0 | 0 | 0 | 0 | 0.192 | 1 | 23 | 1 | 1 | 1 | 12 | 0.2 | 1 | 14 | 156.8 |
| 995 | 853 | 170.6 | 1023.6 | 0 | 255.9 | 196.19 | 29.86 | 8.53 | 0 | 0 | 0 | 0 | 0.192 | 1 | 23 | 1 | 1 | 1 | 12 | 0.2 | 1 | 28 | 166.3 |
| 996 | 853 | 170.6 | 1023.6 | 0 | 255.9 | 196.19 | 29.86 | 8.53 | 0 | 0 | 0 | 0 | 0.192 | 1 | 23 | 1 | 1 | 1 | 12 | 0.2 | 1 | 56 | 173.7 |
| 997 | 853 | 170.6 | 1023.6 | 0 | 255.9 | 196.19 | 29.86 | 25.59 | 0 | 0 | 0 | 0 | 0.192 | 1 | 23 | 1 | 1 | 1 | 12 | 0.2 | 1 | 7 | 148.4 |
| 998 | 853 | 170.6 | 1023.6 | 0 | 255.9 | 196.19 | 29.86 | 25.59 | 0 | 0 | 0 | 0 | 0.192 | 1 | 23 | 1 | 1 | 1 | 12 | 0.2 | 1 | 14 | 163.2 |
| 999 | 853 | 170.6 | 1023.6 | 0 | 255.9 | 196.19 | 29.86 | 25.59 | 0 | 0 | 0 | 0 | 0.192 | 1 | 23 | 1 | 1 | 1 | 12 | 0.2 | 1 | 28 | 174.7 |
| 1000 | 797.78 | 112.36 | 1123.63 | 0 | 0 | 224.73 | 28.09 | 11.9667 | 44.95 | 112.36 | 56.18 | 0 | 0.247 | 1 | 23 | 1 | 1 | 3 | 10 | 0.023 | 1 | 28 | 119.7 |
| 1001 | 853 | 170.6 | 1023.6 | 0 | 255.9 | 196.19 | 29.86 | 25.59 | 0 | 0 | 0 | 0 | 0.192 | 1 | 23 | 1 | 1 | 1 | 12 | 0.2 | 1 | 56 | 185.3 |
| 1002 | 853 | 170.6 | 1023.6 | 0 | 255.9 | 196.19 | 29.86 | 51.18 | 0 | 0 | 0 | 0 | 0.192 | 1 | 23 | 1 | 1 | 1 | 12 | 0.2 | 1 | 7 | 154.7 |
| 1003 | 853 | 170.6 | 1023.6 | 0 | 255.9 | 196.19 | 29.86 | 51.18 | 0 | 0 | 0 | 0 | 0.192 | 1 | 23 | 1 | 1 | 1 | 12 | 0.2 | 1 | 14 | 167.4 |
| 1004 | 853 | 170.6 | 1023.6 | 0 | 255.9 | 196.19 | 29.86 | 51.18 | 0 | 0 | 0 | 0 | 0.192 | 1 | 23 | 1 | 1 | 1 | 12 | 0.2 | 1 | 28 | 182.1 |
| 1005 | 853 | 170.6 | 1023.6 | 0 | 255.9 | 196.19 | 29.86 | 51.18 | 0 | 0 | 0 | 0 | 0.192 | 1 | 23 | 1 | 1 | 1 | 12 | 0.2 | 1 | 56 | 193.7 |
| 1006 | 853 | 170.6 | 1023.6 | 0 | 255.9 | 196.19 | 29.86 | 8.53 | 0 | 0 | 0 | 0 | 0.192 | 1 | 23 | 1 | 1 | 1 | 16 | 0.2 | 1 | 7 | 144.2 |
| 1007 | 853 | 170.6 | 1023.6 | 0 | 255.9 | 196.19 | 29.86 | 8.53 | 0 | 0 | 0 | 0 | 0.192 | 1 | 23 | 1 | 1 | 1 | 16 | 0.2 | 1 | 14 | 155.8 |
| 1008 | 853 | 170.6 | 1023.6 | 0 | 255.9 | 196.19 | 29.86 | 8.53 | 0 | 0 | 0 | 0 | 0.192 | 1 | 23 | 1 | 1 | 1 | 16 | 0.2 | 1 | 28 | 167.4 |
| 1009 | 853 | 170.6 | 1023.6 | 0 | 255.9 | 196.19 | 29.86 | 8.53 | 0 | 0 | 0 | 0 | 0.192 | 1 | 23 | 1 | 1 | 1 | 16 | 0.2 | 1 | 56 | 175.8 |
| 1010 | 853 | 170.6 | 1023.6 | 0 | 255.9 | 196.19 | 29.86 | 25.59 | 0 | 0 | 0 | 0 | 0.192 | 1 | 23 | 1 | 1 | 1 | 16 | 0.2 | 1 | 7 | 150.5 |
| 1011 | 797.78 | 112.36 | 1123.63 | 0 | 0 | 224.73 | 28.09 | 15.9556 | 44.95 | 112.36 | 56.18 | 0 | 0.247 | 1 | 23 | 1 | 1 | 3 | 10 | 0.023 | 1 | 1 | 68.5 |
| 1012 | 853 | 170.6 | 1023.6 | 0 | 255.9 | 196.19 | 29.86 | 25.59 | 0 | 0 | 0 | 0 | 0.192 | 1 | 23 | 1 | 1 | 1 | 16 | 0.2 | 1 | 14 | 160 |
| 1013 | 853 | 170.6 | 1023.6 | 0 | 255.9 | 196.19 | 29.86 | 25.59 | 0 | 0 | 0 | 0 | 0.192 | 1 | 23 | 1 | 1 | 1 | 16 | 0.2 | 1 | 28 | 173.7 |
| 1014 | 853 | 170.6 | 1023.6 | 0 | 255.9 | 196.19 | 29.86 | 25.59 | 0 | 0 | 0 | 0 | 0.192 | 1 | 23 | 1 | 1 | 1 | 16 | 0.2 | 1 | 56 | 184.2 |
| 1015 | 853 | 170.6 | 1023.6 | 0 | 255.9 | 196.19 | 29.86 | 51.18 | 0 | 0 | 0 | 0 | 0.192 | 1 | 23 | 1 | 1 | 1 | 16 | 0.2 | 1 | 7 | 156.8 |
| 1016 | 853 | 170.6 | 1023.6 | 0 | 255.9 | 196.19 | 29.86 | 51.18 | 0 | 0 | 0 | 0 | 0.192 | 1 | 23 | 1 | 1 | 1 | 16 | 0.2 | 1 | 14 | 171.6 |
| 1017 | 853 | 170.6 | 1023.6 | 0 | 255.9 | 196.19 | 29.86 | 51.18 | 0 | 0 | 0 | 0 | 0.192 | 1 | 23 | 1 | 1 | 1 | 16 | 0.2 | 1 | 28 | 178.9 |
| 1018 | 853 | 170.6 | 1023.6 | 0 | 255.9 | 196.19 | 29.86 | 51.18 | 0 | 0 | 0 | 0 | 0.192 | 1 | 23 | 1 | 1 | 1 | 16 | 0.2 | 1 | 56 | 194.7 |
| 1019 | 890 | 0 | 1401.2 | 0 | 0 | 178 | 30.2 | 0 | 0 | 0 | 0 | 0 | 0.200 | 1 | 23 | 1 | 1 | 0 | 0 | 0 | 1 | 1 | 67.2 |
| 1020 | 890 | 0 | 1401.2 | 0 | 0 | 178 | 30.2 | 0 | 0 | 0 | 0 | 0 | 0.200 | 1 | 23 | 1 | 1 | 0 | 0 | 0 | 1 | 1 | 83.6 |
| 1021 | 845.5 | 44.5 | 1401.2 | 0 | 0 | 178 | 30.2 | 0 | 0 | 0 | 0 | 0 | 0.200 | 1 | 23 | 1 | 1 | 0 | 0 | 0 | 1 | 1 | 83.9 |
| 1022 | 797.78 | 112.36 | 1123.63 | 0 | 0 | 224.73 | 28.09 | 15.9556 | 44.95 | 112.36 | 56.18 | 0 | 0.247 | 1 | 23 | 1 | 1 | 3 | 10 | 0.023 | 1 | 14 | 105.7 |
| 1023 | 801 | 89 | 1401.2 | 0 | 0 | 178 | 30.2 | 0 | 0 | 0 | 0 | 0 | 0.200 | 1 | 23 | 1 | 1 | 0 | 0 | 0 | 1 | 1 | 85.8 |
| 1024 | 756.5 | 133.5 | 1401.2 | 0 | 0 | 178 | 30.2 | 0 | 0 | 0 | 0 | 0 | 0.200 | 1 | 23 | 1 | 1 | 0 | 0 | 0 | 1 | 1 | 83.2 |
| 1025 | 712 | 178 | 1401.2 | 0 | 0 | 178 | 30.2 | 0 | 0 | 0 | 0 | 0 | 0.200 | 1 | 23 | 1 | 1 | 0 | 0 | 0 | 1 | 1 | 68.1 |
| 1026 | 845.5 | 44.5 | 1401.2 | 0 | 0 | 178 | 30.2 | 0 | 0 | 0 | 0 | 0 | 0.200 | 1 | 23 | 1 | 1 | 0 | 0 | 0 | 1 | 1 | 88.4 |
| 1027 | 712 | 0 | 1401.2 | 0 | 0 | 178 | 30.2 | 0 | 178 | 0 | 0 | 0 | 0.250 | 1 | 23 | 1 | 1 | 0 | 0 | 0 | 1 | 1 | 60.2 |
| 1028 | 667.5 | 44.5 | 1401.2 | 0 | 0 | 178 | 30.2 | 0 | 178 | 0 | 0 | 0 | 0.250 | 1 | 23 | 1 | 1 | 0 | 0 | 0 | 1 | 1 | 62 |
| 1029 | 1009 | 0 | 1456.8 | 0 | 0 | 201.8 | 34.2 | 0 | 0 | 0 | 0 | 0 | 0.200 | 1 | 23 | 1 | 1 | 0 | 0 | 0 | 1 | 1 | 82.6 |
| 1030 | 1009 | 0 | 1456.8 | 0 | 0 | 201.8 | 34.2 | 0 | 0 | 0 | 0 | 0 | 0.200 | 1 | 23 | 1 | 1 | 0 | 0 | 0 | 1 | 1 | 81.3 |
| 1031 | 958.55 | 50.45 | 1456.8 | 0 | 0 | 201.8 | 34.2 | 0 | 0 | 0 | 0 | 0 | 0.200 | 1 | 23 | 1 | 1 | 0 | 0 | 0 | 1 | 1 | 96.4 |
| 1032 | 756.75 | 50.45 | 1456.8 | 0 | 0 | 201.8 | 34.2 | 0 | 201.8 | 0 | 0 | 0 | 0.250 | 1 | 23 | 1 | 1 | 0 | 0 | 0 | 1 | 1 | 59.8 |
| 1033 | 797.78 | 112.36 | 1123.63 | 0 | 0 | 224.73 | 28.09 | 0 | 44.95 | 112.36 | 56.18 | 0 | 0.247 | 1 | 23 | 1 | 1 | 0 | 0 | 0 | 1 | 28 | 120.4 |
| 1034 | 797.78 | 112.36 | 1123.63 | 0 | 0 | 224.73 | 28.09 | 15.9556 | 44.95 | 112.36 | 56.18 | 0 | 0.247 | 1 | 23 | 1 | 1 | 3 | 10 | 0.023 | 1 | 28 | 117.7 |
| 1035 | 890 | 0 | 1401.2 | 0 | 0 | 178 | 30.2 | 0 | 0 | 0 | 0 | 0 | 0.200 | 1 | 23 | 1 | 1 | 0 | 0 | 0 | 1 | 1 | 68.6 |
| 1036 | 890 | 0 | 1401.2 | 0 | 0 | 178 | 30.2 | 0 | 0 | 0 | 0 | 0 | 0.200 | 1 | 23 | 1 | 1 | 0 | 0 | 0 | 1 | 1 | 81.4 |
| 1037 | 845.5 | 44.5 | 1401.2 | 0 | 0 | 178 | 30.2 | 0 | 0 | 0 | 0 | 0 | 0.200 | 1 | 23 | 1 | 1 | 0 | 0 | 0 | 1 | 1 | 85 |
| 1038 | 801 | 89 | 1401.2 | 0 | 0 | 178 | 30.2 | 0 | 0 | 0 | 0 | 0 | 0.200 | 1 | 23 | 1 | 1 | 0 | 0 | 0 | 1 | 1 | 87.9 |
| 1039 | 756.5 | 133.5 | 1401.2 | 0 | 0 | 178 | 30.2 | 0 | 0 | 0 | 0 | 0 | 0.200 | 1 | 23 | 1 | 1 | 0 | 0 | 0 | 1 | 1 | 80.2 |
| 1040 | 712 | 178 | 1401.2 | 0 | 0 | 178 | 30.2 | 0 | 0 | 0 | 0 | 0 | 0.200 | 1 | 23 | 1 | 1 | 0 | 0 | 0 | 1 | 1 | 72.9 |
| 1041 | 845.5 | 44.5 | 1401.2 | 0 | 0 | 178 | 30.2 | 0 | 0 | 0 | 0 | 0 | 0.200 | 1 | 23 | 1 | 1 | 0 | 0 | 0 | 1 | 1 | 89.3 |
| 1042 | 712 | 0 | 1401.2 | 0 | 0 | 178 | 30.2 | 0 | 178 | 0 | 0 | 0 | 0.250 | 1 | 23 | 1 | 1 | 0 | 0 | 0 | 1 | 1 | 62.4 |
| 1043 | 667.5 | 44.5 | 1401.2 | 0 | 0 | 178 | 30.2 | 0 | 178 | 0 | 0 | 0 | 0.250 | 1 | 23 | 1 | 1 | 0 | 0 | 0 | 1 | 1 | 60.1 |
| 1044 | 1009 | 0 | 1456.8 | 0 | 0 | 201.8 | 34.2 | 0 | 0 | 0 | 0 | 0 | 0.200 | 1 | 23 | 1 | 1 | 0 | 0 | 0 | 1 | 1 | 85.2 |
| 1045 | 797.78 | 112.36 | 1123.63 | 0 | 0 | 224.73 | 28.09 | 19.9445 | 44.95 | 112.36 | 56.18 | 0 | 0.247 | 1 | 23 | 1 | 1 | 3 | 10 | 0.023 | 1 | 1 | 65.2 |
| 1046 | 1009 | 0 | 1456.8 | 0 | 0 | 201.8 | 34.2 | 0 | 0 | 0 | 0 | 0 | 0.200 | 1 | 23 | 1 | 1 | 0 | 0 | 0 | 1 | 1 | 87.5 |
| 1047 | 958.55 | 50.45 | 1456.8 | 0 | 0 | 201.8 | 34.2 | 0 | 0 | 0 | 0 | 0 | 0.200 | 1 | 23 | 1 | 1 | 0 | 0 | 0 | 1 | 1 | 93.7 |
| 1048 | 756.75 | 50.45 | 1456.8 | 0 | 0 | 201.8 | 34.2 | 0 | 201.8 | 0 | 0 | 0 | 0.250 | 1 | 23 | 1 | 1 | 0 | 0 | 0 | 1 | 1 | 62 |
| 1049 | 890 | 0 | 1401.2 | 0 | 0 | 178 | 30.2 | 0 | 0 | 0 | 0 | 0 | 0.200 | 1 | 23 | 1 | 1 | 0 | 0 | 0 | 1 | 1 | 68.6 |
| 1050 | 890 | 0 | 1401.2 | 0 | 0 | 178 | 30.2 | 0 | 0 | 0 | 0 | 0 | 0.200 | 1 | 23 | 1 | 1 | 0 | 0 | 0 | 1 | 1 | 79.9 |
| 1051 | 845.5 | 44.5 | 1401.2 | 0 | 0 | 178 | 30.2 | 0 | 0 | 0 | 0 | 0 | 0.200 | 1 | 23 | 1 | 1 | 0 | 0 | 0 | 1 | 1 | 84.8 |
| 1052 | 801 | 89 | 1401.2 | 0 | 0 | 178 | 30.2 | 0 | 0 | 0 | 0 | 0 | 0.200 | 1 | 23 | 1 | 1 | 0 | 0 | 0 | 1 | 1 | 88.6 |
| 1053 | 756.5 | 133.5 | 1401.2 | 0 | 0 | 178 | 30.2 | 0 | 0 | 0 | 0 | 0 | 0.200 | 1 | 23 | 1 | 1 | 0 | 0 | 0 | 1 | 1 | 81 |
| 1054 | 712 | 178 | 1401.2 | 0 | 0 | 178 | 30.2 | 0 | 0 | 0 | 0 | 0 | 0.200 | 1 | 23 | 1 | 1 | 0 | 0 | 0 | 1 | 1 | 74.2 |
| 1055 | 845.5 | 44.5 | 1401.2 | 0 | 0 | 178 | 30.2 | 0 | 0 | 0 | 0 | 0 | 0.200 | 1 | 23 | 1 | 1 | 0 | 0 | 0 | 1 | 1 | 91.9 |
| 1056 | 797.78 | 112.36 | 1123.63 | 0 | 0 | 224.73 | 28.09 | 19.9445 | 44.95 | 112.36 | 56.18 | 0 | 0.247 | 1 | 23 | 1 | 1 | 3 | 10 | 0.023 | 1 | 14 | 103.4 |
| 1057 | 712 | 0 | 1401.2 | 0 | 0 | 178 | 30.2 | 0 | 178 | 0 | 0 | 0 | 0.250 | 1 | 23 | 1 | 1 | 0 | 0 | 0 | 1 | 1 | 63.5 |
| 1058 | 667.5 | 44.5 | 1401.2 | 0 | 0 | 178 | 30.2 | 0 | 178 | 0 | 0 | 0 | 0.250 | 1 | 23 | 1 | 1 | 0 | 0 | 0 | 1 | 1 | 60.9 |
| 1059 | 1009 | 0 | 1456.8 | 0 | 0 | 201.8 | 34.2 | 0 | 0 | 0 | 0 | 0 | 0.200 | 1 | 23 | 1 | 1 | 0 | 0 | 0 | 1 | 1 | 84.4 |
| 1060 | 1009 | 0 | 1456.8 | 0 | 0 | 201.8 | 34.2 | 0 | 0 | 0 | 0 | 0 | 0.200 | 1 | 23 | 1 | 1 | 0 | 0 | 0 | 1 | 1 | 84.8 |
| 1061 | 958.55 | 50.45 | 1456.8 | 0 | 0 | 201.8 | 34.2 | 0 | 0 | 0 | 0 | 0 | 0.200 | 1 | 23 | 1 | 1 | 0 | 0 | 0 | 1 | 1 | 92.5 |
| 1062 | 756.75 | 50.45 | 1456.8 | 0 | 0 | 201.8 | 34.2 | 0 | 201.8 | 0 | 0 | 0 | 0.250 | 1 | 23 | 1 | 1 | 0 | 0 | 0 | 1 | 1 | 61.3 |
| 1063 | 890 | 0 | 1401.2 | 0 | 0 | 178 | 30.2 | 0 | 0 | 0 | 0 | 0 | 0.200 | 1 | 23 | 1 | 1 | 0 | 0 | 0 | 1 | 7 | 111.3 |
| 1064 | 890 | 0 | 1401.2 | 0 | 0 | 178 | 30.2 | 0 | 0 | 0 | 0 | 0 | 0.200 | 1 | 23 | 1 | 1 | 0 | 0 | 0 | 1 | 7 | 112.2 |
| 1065 | 845.5 | 44.5 | 1401.2 | 0 | 0 | 178 | 30.2 | 0 | 0 | 0 | 0 | 0 | 0.200 | 1 | 23 | 1 | 1 | 0 | 0 | 0 | 1 | 7 | 120.9 |
| 1066 | 801 | 89 | 1401.2 | 0 | 0 | 178 | 30.2 | 0 | 0 | 0 | 0 | 0 | 0.200 | 1 | 23 | 1 | 1 | 0 | 0 | 0 | 1 | 7 | 117.3 |
| 1067 | 797.78 | 112.36 | 1123.63 | 0 | 0 | 224.73 | 28.09 | 19.9445 | 44.95 | 112.36 | 56.18 | 0 | 0.247 | 1 | 23 | 1 | 1 | 3 | 10 | 0.023 | 1 | 28 | 109.9 |
| 1068 | 756.5 | 133.5 | 1401.2 | 0 | 0 | 178 | 30.2 | 0 | 0 | 0 | 0 | 0 | 0.200 | 1 | 23 | 1 | 1 | 0 | 0 | 0 | 1 | 7 | 112.6 |
| 1069 | 712 | 178 | 1401.2 | 0 | 0 | 178 | 30.2 | 0 | 0 | 0 | 0 | 0 | 0.200 | 1 | 23 | 1 | 1 | 0 | 0 | 0 | 1 | 7 | 110.6 |
| 1070 | 845.5 | 44.5 | 1401.2 | 0 | 0 | 178 | 30.2 | 0 | 0 | 0 | 0 | 0 | 0.200 | 1 | 23 | 1 | 1 | 0 | 0 | 0 | 1 | 7 | 118.3 |
| 1071 | 712 | 0 | 1401.2 | 0 | 0 | 178 | 30.2 | 0 | 178 | 0 | 0 | 0 | 0.250 | 1 | 23 | 1 | 1 | 0 | 0 | 0 | 1 | 7 | 115.6 |
| 1072 | 623 | 0 | 1401.2 | 0 | 0 | 178 | 30.2 | 0 | 267 | 0 | 0 | 0 | 0.286 | 1 | 23 | 1 | 1 | 0 | 0 | 0 | 1 | 7 | 117.3 |
| 1073 | 534 | 0 | 1401.2 | 0 | 0 | 178 | 30.2 | 0 | 356 | 0 | 0 | 0 | 0.333 | 1 | 23 | 1 | 1 | 0 | 0 | 0 | 1 | 7 | 84.1 |
| 1074 | 667.5 | 44.5 | 1401.2 | 0 | 0 | 178 | 30.2 | 0 | 178 | 0 | 0 | 0 | 0.250 | 1 | 23 | 1 | 1 | 0 | 0 | 0 | 1 | 7 | 107.2 |
| 1075 | 1009 | 0 | 1456.8 | 0 | 0 | 201.8 | 34.2 | 0 | 0 | 0 | 0 | 0 | 0.200 | 1 | 23 | 1 | 1 | 0 | 0 | 0 | 1 | 7 | 116.2 |
| 1076 | 1009 | 0 | 1456.8 | 0 | 0 | 201.8 | 34.2 | 0 | 0 | 0 | 0 | 0 | 0.200 | 1 | 23 | 1 | 1 | 0 | 0 | 0 | 1 | 7 | 117.5 |
| 1077 | 958.55 | 50.45 | 1456.8 | 0 | 0 | 201.8 | 34.2 | 0 | 0 | 0 | 0 | 0 | 0.200 | 1 | 23 | 1 | 1 | 0 | 0 | 0 | 1 | 7 | 119 |
| 1078 | 797.78 | 112.36 | 1123.63 | 0 | 0 | 224.73 | 28.09 | 3.9889 | 44.95 | 112.36 | 56.18 | 0 | 0.247 | 1 | 23 | 1 | 1 | 5 | 12 | 0.01 | 1 | 1 | 75.4 |
| 1079 | 756.75 | 50.45 | 1456.8 | 0 | 0 | 201.8 | 34.2 | 0 | 201.8 | 0 | 0 | 0 | 0.250 | 1 | 23 | 1 | 1 | 0 | 0 | 0 | 1 | 7 | 115.8 |
| 1080 | 890 | 0 | 1401.2 | 0 | 0 | 178 | 30.2 | 0 | 0 | 0 | 0 | 0 | 0.200 | 1 | 23 | 1 | 1 | 0 | 0 | 0 | 1 | 7 | 110.4 |
| 1081 | 890 | 0 | 1401.2 | 0 | 0 | 178 | 30.2 | 0 | 0 | 0 | 0 | 0 | 0.200 | 1 | 23 | 1 | 1 | 0 | 0 | 0 | 1 | 7 | 112.5 |
| 1082 | 845.5 | 44.5 | 1401.2 | 0 | 0 | 178 | 30.2 | 0 | 0 | 0 | 0 | 0 | 0.200 | 1 | 23 | 1 | 1 | 0 | 0 | 0 | 1 | 7 | 121.3 |
| 1083 | 801 | 89 | 1401.2 | 0 | 0 | 178 | 30.2 | 0 | 0 | 0 | 0 | 0 | 0.200 | 1 | 23 | 1 | 1 | 0 | 0 | 0 | 1 | 7 | 118.6 |
| 1084 | 756.5 | 133.5 | 1401.2 | 0 | 0 | 178 | 30.2 | 0 | 0 | 0 | 0 | 0 | 0.200 | 1 | 23 | 1 | 1 | 0 | 0 | 0 | 1 | 7 | 111.3 |
| 1085 | 712 | 178 | 1401.2 | 0 | 0 | 178 | 30.2 | 0 | 0 | 0 | 0 | 0 | 0.200 | 1 | 23 | 1 | 1 | 0 | 0 | 0 | 1 | 7 | 110.9 |
| 1086 | 845.5 | 44.5 | 1401.2 | 0 | 0 | 178 | 30.2 | 0 | 0 | 0 | 0 | 0 | 0.200 | 1 | 23 | 1 | 1 | 0 | 0 | 0 | 1 | 7 | 121.9 |
| 1087 | 712 | 0 | 1401.2 | 0 | 0 | 178 | 30.2 | 0 | 178 | 0 | 0 | 0 | 0.250 | 1 | 23 | 1 | 1 | 0 | 0 | 0 | 1 | 7 | 111.1 |
| 1088 | 623 | 0 | 1401.2 | 0 | 0 | 178 | 30.2 | 0 | 267 | 0 | 0 | 0 | 0.286 | 1 | 23 | 1 | 1 | 0 | 0 | 0 | 1 | 7 | 115.2 |
| 1089 | 797.78 | 112.36 | 1123.63 | 0 | 0 | 224.73 | 28.09 | 3.9889 | 44.95 | 112.36 | 56.18 | 0 | 0.247 | 1 | 23 | 1 | 1 | 5 | 12 | 0.01 | 1 | 14 | 110.3 |
| 1090 | 534 | 0 | 1401.2 | 0 | 0 | 178 | 30.2 | 0 | 356 | 0 | 0 | 0 | 0.333 | 1 | 23 | 1 | 1 | 0 | 0 | 0 | 1 | 7 | 88.4 |
| 1091 | 667.5 | 44.5 | 1401.2 | 0 | 0 | 178 | 30.2 | 0 | 178 | 0 | 0 | 0 | 0.250 | 1 | 23 | 1 | 1 | 0 | 0 | 0 | 1 | 7 | 109 |
| 1092 | 1009 | 0 | 1456.8 | 0 | 0 | 201.8 | 34.2 | 0 | 0 | 0 | 0 | 0 | 0.200 | 1 | 23 | 1 | 1 | 0 | 0 | 0 | 1 | 7 | 119.5 |
| 1093 | 1009 | 0 | 1456.8 | 0 | 0 | 201.8 | 34.2 | 0 | 0 | 0 | 0 | 0 | 0.200 | 1 | 23 | 1 | 1 | 0 | 0 | 0 | 1 | 7 | 117.8 |
| 1094 | 958.55 | 50.45 | 1456.8 | 0 | 0 | 201.8 | 34.2 | 0 | 0 | 0 | 0 | 0 | 0.200 | 1 | 23 | 1 | 1 | 0 | 0 | 0 | 1 | 7 | 116 |
| 1095 | 756.75 | 50.45 | 1456.8 | 0 | 0 | 201.8 | 34.2 | 0 | 201.8 | 0 | 0 | 0 | 0.250 | 1 | 23 | 1 | 1 | 0 | 0 | 0 | 1 | 7 | 118.3 |
| 1096 | 890 | 0 | 1401.2 | 0 | 0 | 178 | 30.2 | 0 | 0 | 0 | 0 | 0 | 0.200 | 1 | 23 | 1 | 1 | 0 | 0 | 0 | 1 | 7 | 109.8 |
| 1097 | 890 | 0 | 1401.2 | 0 | 0 | 178 | 30.2 | 0 | 0 | 0 | 0 | 0 | 0.200 | 1 | 23 | 1 | 1 | 0 | 0 | 0 | 1 | 7 | 112.8 |
| 1098 | 845.5 | 44.5 | 1401.2 | 0 | 0 | 178 | 30.2 | 0 | 0 | 0 | 0 | 0 | 0.200 | 1 | 23 | 1 | 1 | 0 | 0 | 0 | 1 | 7 | 124 |
| 1099 | 801 | 89 | 1401.2 | 0 | 0 | 178 | 30.2 | 0 | 0 | 0 | 0 | 0 | 0.200 | 1 | 23 | 1 | 1 | 0 | 0 | 0 | 1 | 7 | 119.5 |
| 1100 | 797.78 | 112.36 | 1123.63 | 0 | 0 | 224.73 | 28.09 | 3.9889 | 44.95 | 112.36 | 56.18 | 0 | 0.247 | 1 | 23 | 1 | 1 | 5 | 12 | 0.01 | 1 | 28 | 128.4 |
| 1101 | 756.5 | 133.5 | 1401.2 | 0 | 0 | 178 | 30.2 | 0 | 0 | 0 | 0 | 0 | 0.200 | 1 | 23 | 1 | 1 | 0 | 0 | 0 | 1 | 7 | 111.5 |
| 1102 | 712 | 178 | 1401.2 | 0 | 0 | 178 | 30.2 | 0 | 0 | 0 | 0 | 0 | 0.200 | 1 | 23 | 1 | 1 | 0 | 0 | 0 | 1 | 7 | 110.8 |
| 1103 | 845.5 | 44.5 | 1401.2 | 0 | 0 | 178 | 30.2 | 0 | 0 | 0 | 0 | 0 | 0.200 | 1 | 23 | 1 | 1 | 0 | 0 | 0 | 1 | 7 | 127.5 |
| 1104 | 712 | 0 | 1401.2 | 0 | 0 | 178 | 30.2 | 0 | 178 | 0 | 0 | 0 | 0.250 | 1 | 23 | 1 | 1 | 0 | 0 | 0 | 1 | 7 | 117 |
| 1105 | 623 | 0 | 1401.2 | 0 | 0 | 178 | 30.2 | 0 | 267 | 0 | 0 | 0 | 0.286 | 1 | 23 | 1 | 1 | 0 | 0 | 0 | 1 | 7 | 118.2 |
| 1106 | 534 | 0 | 1401.2 | 0 | 0 | 178 | 30.2 | 0 | 356 | 0 | 0 | 0 | 0.333 | 1 | 23 | 1 | 1 | 0 | 0 | 0 | 1 | 7 | 90.2 |
| 1107 | 667.5 | 44.5 | 1401.2 | 0 | 0 | 178 | 30.2 | 0 | 178 | 0 | 0 | 0 | 0.250 | 1 | 23 | 1 | 1 | 0 | 0 | 0 | 1 | 7 | 105.3 |
| 1108 | 1009 | 0 | 1456.8 | 0 | 0 | 201.8 | 34.2 | 0 | 0 | 0 | 0 | 0 | 0.200 | 1 | 23 | 1 | 1 | 0 | 0 | 0 | 1 | 7 | 120.5 |
| 1109 | 1009 | 0 | 1456.8 | 0 | 0 | 201.8 | 34.2 | 0 | 0 | 0 | 0 | 0 | 0.200 | 1 | 23 | 1 | 1 | 0 | 0 | 0 | 1 | 7 | 119.2 |
| 1110 | 958.55 | 50.45 | 1456.8 | 0 | 0 | 201.8 | 34.2 | 0 | 0 | 0 | 0 | 0 | 0.200 | 1 | 23 | 1 | 1 | 0 | 0 | 0 | 1 | 7 | 121.3 |
| 1111 | 797.78 | 112.36 | 1123.63 | 0 | 0 | 224.73 | 28.09 | 7.9778 | 44.95 | 112.36 | 56.18 | 0 | 0.247 | 1 | 23 | 1 | 1 | 5 | 12 | 0.01 | 1 | 1 | 74.8 |
| 1112 | 756.75 | 50.45 | 1456.8 | 0 | 0 | 201.8 | 34.2 | 0 | 201.8 | 0 | 0 | 0 | 0.250 | 1 | 23 | 1 | 1 | 0 | 0 | 0 | 1 | 7 | 117.6 |
| 1113 | 890 | 0 | 1401.2 | 0 | 0 | 178 | 30.2 | 0 | 0 | 0 | 0 | 0 | 0.200 | 1 | 23 | 1 | 1 | 0 | 0 | 0 | 1 | 28 | 122.1 |
| 1114 | 890 | 0 | 1401.2 | 0 | 0 | 178 | 30.2 | 0 | 0 | 0 | 0 | 0 | 0.200 | 1 | 23 | 1 | 1 | 0 | 0 | 0 | 1 | 28 | 131.8 |
| 1115 | 845.5 | 44.5 | 1401.2 | 0 | 0 | 178 | 30.2 | 0 | 0 | 0 | 0 | 0 | 0.200 | 1 | 23 | 1 | 1 | 0 | 0 | 0 | 1 | 28 | 134 |
| 1116 | 801 | 89 | 1401.2 | 0 | 0 | 178 | 30.2 | 0 | 0 | 0 | 0 | 0 | 0.200 | 1 | 23 | 1 | 1 | 0 | 0 | 0 | 1 | 28 | 134.6 |
| 1117 | 756.5 | 133.5 | 1401.2 | 0 | 0 | 178 | 30.2 | 0 | 0 | 0 | 0 | 0 | 0.200 | 1 | 23 | 1 | 1 | 0 | 0 | 0 | 1 | 28 | 136.3 |
| 1118 | 712 | 178 | 1401.2 | 0 | 0 | 178 | 30.2 | 0 | 0 | 0 | 0 | 0 | 0.200 | 1 | 23 | 1 | 1 | 0 | 0 | 0 | 1 | 28 | 127.6 |
| 1119 | 845.5 | 44.5 | 1401.2 | 0 | 0 | 178 | 30.2 | 0 | 0 | 0 | 0 | 0 | 0.200 | 1 | 23 | 1 | 1 | 0 | 0 | 0 | 1 | 28 | 134.8 |
| 1120 | 712 | 0 | 1401.2 | 0 | 0 | 178 | 30.2 | 0 | 178 | 0 | 0 | 0 | 0.250 | 1 | 23 | 1 | 1 | 0 | 0 | 0 | 1 | 28 | 127.1 |
| 1121 | 623 | 0 | 1401.2 | 0 | 0 | 178 | 30.2 | 0 | 267 | 0 | 0 | 0 | 0.286 | 1 | 23 | 1 | 1 | 0 | 0 | 0 | 1 | 28 | 131.8 |
| 1122 | 797.78 | 112.36 | 1123.63 | 0 | 0 | 224.73 | 28.09 | 7.9778 | 44.95 | 112.36 | 56.18 | 0 | 0.247 | 1 | 23 | 1 | 1 | 5 | 12 | 0.01 | 1 | 14 | 105.3 |
| 1123 | 534 | 0 | 1401.2 | 0 | 0 | 178 | 30.2 | 0 | 356 | 0 | 0 | 0 | 0.333 | 1 | 23 | 1 | 1 | 0 | 0 | 0 | 1 | 28 | 119 |
| 1124 | 667.5 | 44.5 | 1401.2 | 0 | 0 | 178 | 30.2 | 0 | 178 | 0 | 0 | 0 | 0.250 | 1 | 23 | 1 | 1 | 0 | 0 | 0 | 1 | 28 | 130.6 |
| 1125 | 1009 | 0 | 1456.8 | 0 | 0 | 201.8 | 34.2 | 0 | 0 | 0 | 0 | 0 | 0.200 | 1 | 23 | 1 | 1 | 0 | 0 | 0 | 1 | 28 | 131.7 |
| 1126 | 1009 | 0 | 1456.8 | 0 | 0 | 201.8 | 34.2 | 0 | 0 | 0 | 0 | 0 | 0.200 | 1 | 23 | 1 | 1 | 0 | 0 | 0 | 1 | 28 | 131.7 |
| 1127 | 958.55 | 50.45 | 1456.8 | 0 | 0 | 201.8 | 34.2 | 0 | 0 | 0 | 0 | 0 | 0.200 | 1 | 23 | 1 | 1 | 0 | 0 | 0 | 1 | 28 | 132.8 |
| 1128 | 756.75 | 50.45 | 1456.8 | 0 | 0 | 201.8 | 34.2 | 0 | 201.8 | 0 | 0 | 0 | 0.250 | 1 | 23 | 1 | 1 | 0 | 0 | 0 | 1 | 28 | 131.4 |
| 1129 | 890 | 0 | 1401.2 | 0 | 0 | 178 | 30.2 | 0 | 0 | 0 | 0 | 0 | 0.200 | 1 | 23 | 1 | 1 | 0 | 0 | 0 | 1 | 28 | 121.4 |
| 1130 | 890 | 0 | 1401.2 | 0 | 0 | 178 | 30.2 | 0 | 0 | 0 | 0 | 0 | 0.200 | 1 | 23 | 1 | 1 | 0 | 0 | 0 | 1 | 28 | 130.3 |
| 1131 | 845.5 | 44.5 | 1401.2 | 0 | 0 | 178 | 30.2 | 0 | 0 | 0 | 0 | 0 | 0.200 | 1 | 23 | 1 | 1 | 0 | 0 | 0 | 1 | 28 | 136.3 |
| 1132 | 801 | 89 | 1401.2 | 0 | 0 | 178 | 30.2 | 0 | 0 | 0 | 0 | 0 | 0.200 | 1 | 23 | 1 | 1 | 0 | 0 | 0 | 1 | 28 | 137.4 |
| 1133 | 797.78 | 112.36 | 1123.63 | 0 | 0 | 224.73 | 28.09 | 7.9778 | 44.95 | 112.36 | 56.18 | 0 | 0.247 | 1 | 23 | 1 | 1 | 5 | 12 | 0.01 | 1 | 28 | 123.4 |
| 1134 | 756.5 | 133.5 | 1401.2 | 0 | 0 | 178 | 30.2 | 0 | 0 | 0 | 0 | 0 | 0.200 | 1 | 23 | 1 | 1 | 0 | 0 | 0 | 1 | 28 | 136.9 |
| 1135 | 712 | 178 | 1401.2 | 0 | 0 | 178 | 30.2 | 0 | 0 | 0 | 0 | 0 | 0.200 | 1 | 23 | 1 | 1 | 0 | 0 | 0 | 1 | 28 | 129.5 |
| 1136 | 845.5 | 44.5 | 1401.2 | 0 | 0 | 178 | 30.2 | 0 | 0 | 0 | 0 | 0 | 0.200 | 1 | 23 | 1 | 1 | 0 | 0 | 0 | 1 | 28 | 136.4 |
| 1137 | 712 | 0 | 1401.2 | 0 | 0 | 178 | 30.2 | 0 | 178 | 0 | 0 | 0 | 0.250 | 1 | 23 | 1 | 1 | 0 | 0 | 0 | 1 | 28 | 126.4 |
| 1138 | 623 | 0 | 1401.2 | 0 | 0 | 178 | 30.2 | 0 | 267 | 0 | 0 | 0 | 0.286 | 1 | 23 | 1 | 1 | 0 | 0 | 0 | 1 | 28 | 131.4 |
| 1139 | 534 | 0 | 1401.2 | 0 | 0 | 178 | 30.2 | 0 | 356 | 0 | 0 | 0 | 0.333 | 1 | 23 | 1 | 1 | 0 | 0 | 0 | 1 | 28 | 117.6 |
| 1140 | 667.5 | 44.5 | 1401.2 | 0 | 0 | 178 | 30.2 | 0 | 178 | 0 | 0 | 0 | 0.250 | 1 | 23 | 1 | 1 | 0 | 0 | 0 | 1 | 28 | 130.3 |
| 1141 | 1009 | 0 | 1456.8 | 0 | 0 | 201.8 | 34.2 | 0 | 0 | 0 | 0 | 0 | 0.200 | 1 | 23 | 1 | 1 | 0 | 0 | 0 | 1 | 28 | 132.9 |
| 1142 | 1009 | 0 | 1456.8 | 0 | 0 | 201.8 | 34.2 | 0 | 0 | 0 | 0 | 0 | 0.200 | 1 | 23 | 1 | 1 | 0 | 0 | 0 | 1 | 28 | 132.5 |
| 1143 | 958.55 | 50.45 | 1456.8 | 0 | 0 | 201.8 | 34.2 | 0 | 0 | 0 | 0 | 0 | 0.200 | 1 | 23 | 1 | 1 | 0 | 0 | 0 | 1 | 28 | 132.8 |
| 1144 | 797.78 | 112.36 | 1123.63 | 0 | 0 | 224.73 | 28.09 | 3.9889 | 44.95 | 112.36 | 56.18 | 0 | 0.247 | 1 | 23 | 1 | 1 | 4 | 18 | 0.013 | 1 | 1 | 67.4 |
| 1145 | 797.78 | 112.36 | 1123.63 | 0 | 0 | 224.73 | 28.09 | 11.9667 | 44.95 | 112.36 | 56.18 | 0 | 0.247 | 1 | 23 | 1 | 1 | 5 | 12 | 0.01 | 1 | 1 | 71.1 |
| 1146 | 756.75 | 50.45 | 1456.8 | 0 | 0 | 201.8 | 34.2 | 0 | 201.8 | 0 | 0 | 0 | 0.250 | 1 | 23 | 1 | 1 | 0 | 0 | 0 | 1 | 28 | 132 |
| 1147 | 890 | 0 | 1401.2 | 0 | 0 | 178 | 30.2 | 0 | 0 | 0 | 0 | 0 | 0.200 | 1 | 23 | 1 | 1 | 0 | 0 | 0 | 1 | 28 | 124.9 |
| 1148 | 890 | 0 | 1401.2 | 0 | 0 | 178 | 30.2 | 0 | 0 | 0 | 0 | 0 | 0.200 | 1 | 23 | 1 | 1 | 0 | 0 | 0 | 1 | 28 | 130.2 |
| 1149 | 845.5 | 44.5 | 1401.2 | 0 | 0 | 178 | 30.2 | 0 | 0 | 0 | 0 | 0 | 0.200 | 1 | 23 | 1 | 1 | 0 | 0 | 0 | 1 | 28 | 135.8 |
| 1150 | 801 | 89 | 1401.2 | 0 | 0 | 178 | 30.2 | 0 | 0 | 0 | 0 | 0 | 0.200 | 1 | 23 | 1 | 1 | 0 | 0 | 0 | 1 | 28 | 138.9 |
| 1151 | 756.5 | 133.5 | 1401.2 | 0 | 0 | 178 | 30.2 | 0 | 0 | 0 | 0 | 0 | 0.200 | 1 | 23 | 1 | 1 | 0 | 0 | 0 | 1 | 28 | 135.7 |
| 1152 | 712 | 178 | 1401.2 | 0 | 0 | 178 | 30.2 | 0 | 0 | 0 | 0 | 0 | 0.200 | 1 | 23 | 1 | 1 | 0 | 0 | 0 | 1 | 28 | 127.5 |
| 1153 | 845.5 | 44.5 | 1401.2 | 0 | 0 | 178 | 30.2 | 0 | 0 | 0 | 0 | 0 | 0.200 | 1 | 23 | 1 | 1 | 0 | 0 | 0 | 1 | 28 | 132.8 |
| 1154 | 712 | 0 | 1401.2 | 0 | 0 | 178 | 30.2 | 0 | 178 | 0 | 0 | 0 | 0.250 | 1 | 23 | 1 | 1 | 0 | 0 | 0 | 1 | 28 | 127.4 |
| 1155 | 623 | 0 | 1401.2 | 0 | 0 | 178 | 30.2 | 0 | 267 | 0 | 0 | 0 | 0.286 | 1 | 23 | 1 | 1 | 0 | 0 | 0 | 1 | 28 | 134.6 |
| 1156 | 797.78 | 112.36 | 1123.63 | 0 | 0 | 224.73 | 28.09 | 11.9667 | 44.95 | 112.36 | 56.18 | 0 | 0.247 | 1 | 23 | 1 | 1 | 5 | 12 | 0.01 | 1 | 14 | 109.2 |
| 1157 | 534 | 0 | 1401.2 | 0 | 0 | 178 | 30.2 | 0 | 356 | 0 | 0 | 0 | 0.333 | 1 | 23 | 1 | 1 | 0 | 0 | 0 | 1 | 28 | 119 |
| 1158 | 667.5 | 44.5 | 1401.2 | 0 | 0 | 178 | 30.2 | 0 | 178 | 0 | 0 | 0 | 0.250 | 1 | 23 | 1 | 1 | 0 | 0 | 0 | 1 | 28 | 129.9 |
| 1159 | 1009 | 0 | 1456.8 | 0 | 0 | 201.8 | 34.2 | 0 | 0 | 0 | 0 | 0 | 0.200 | 1 | 23 | 1 | 1 | 0 | 0 | 0 | 1 | 28 | 129.7 |
| 1160 | 1009 | 0 | 1456.8 | 0 | 0 | 201.8 | 34.2 | 0 | 0 | 0 | 0 | 0 | 0.200 | 1 | 23 | 1 | 1 | 0 | 0 | 0 | 1 | 28 | 135 |
| 1161 | 958.55 | 50.45 | 1456.8 | 0 | 0 | 201.8 | 34.2 | 0 | 0 | 0 | 0 | 0 | 0.200 | 1 | 23 | 1 | 1 | 0 | 0 | 0 | 1 | 28 | 134.2 |
| 1162 | 756.75 | 50.45 | 1456.8 | 0 | 0 | 201.8 | 34.2 | 0 | 201.8 | 0 | 0 | 0 | 0.250 | 1 | 23 | 1 | 1 | 0 | 0 | 0 | 1 | 28 | 133.3 |
| 1163 | 890 | 0 | 1401.2 | 0 | 0 | 178 | 30.2 | 0 | 0 | 0 | 0 | 0 | 0.200 | 1 | 23 | 1 | 1 | 0 | 0 | 0 | 1 | 56 | 125.7 |
| 1164 | 890 | 0 | 1401.2 | 0 | 0 | 178 | 30.2 | 0 | 0 | 0 | 0 | 0 | 0.200 | 1 | 23 | 1 | 1 | 0 | 0 | 0 | 1 | 56 | 131.4 |
| 1165 | 845.5 | 44.5 | 1401.2 | 0 | 0 | 178 | 30.2 | 0 | 0 | 0 | 0 | 0 | 0.200 | 1 | 23 | 1 | 1 | 0 | 0 | 0 | 1 | 56 | 138.9 |
| 1166 | 801 | 89 | 1401.2 | 0 | 0 | 178 | 30.2 | 0 | 0 | 0 | 0 | 0 | 0.200 | 1 | 23 | 1 | 1 | 0 | 0 | 0 | 1 | 56 | 147.8 |
| 1167 | 797.78 | 112.36 | 1123.63 | 0 | 0 | 224.73 | 28.09 | 11.9667 | 44.95 | 112.36 | 56.18 | 0 | 0.247 | 1 | 23 | 1 | 1 | 5 | 12 | 0.01 | 1 | 28 | 123.4 |
| 1168 | 756.5 | 133.5 | 1401.2 | 0 | 0 | 178 | 30.2 | 0 | 0 | 0 | 0 | 0 | 0.200 | 1 | 23 | 1 | 1 | 0 | 0 | 0 | 1 | 56 | 138.7 |
| 1169 | 712 | 178 | 1401.2 | 0 | 0 | 178 | 30.2 | 0 | 0 | 0 | 0 | 0 | 0.200 | 1 | 23 | 1 | 1 | 0 | 0 | 0 | 1 | 56 | 135.5 |
| 1170 | 845.5 | 44.5 | 1401.2 | 0 | 0 | 178 | 30.2 | 0 | 0 | 0 | 0 | 0 | 0.200 | 1 | 23 | 1 | 1 | 0 | 0 | 0 | 1 | 56 | 143.6 |
| 1171 | 712 | 0 | 1401.2 | 0 | 0 | 178 | 30.2 | 0 | 178 | 0 | 0 | 0 | 0.250 | 1 | 23 | 1 | 1 | 0 | 0 | 0 | 1 | 56 | 127.2 |
| 1172 | 623 | 0 | 1401.2 | 0 | 0 | 178 | 30.2 | 0 | 267 | 0 | 0 | 0 | 0.286 | 1 | 23 | 1 | 1 | 0 | 0 | 0 | 1 | 56 | 135.3 |
| 1173 | 534 | 0 | 1401.2 | 0 | 0 | 178 | 30.2 | 0 | 356 | 0 | 0 | 0 | 0.333 | 1 | 23 | 1 | 1 | 0 | 0 | 0 | 1 | 56 | 126.6 |
| 1174 | 667.5 | 44.5 | 1401.2 | 0 | 0 | 178 | 30.2 | 0 | 178 | 0 | 0 | 0 | 0.250 | 1 | 23 | 1 | 1 | 0 | 0 | 0 | 1 | 56 | 130.4 |
| 1175 | 1009 | 0 | 1456.8 | 0 | 0 | 201.8 | 34.2 | 0 | 0 | 0 | 0 | 0 | 0.200 | 1 | 23 | 1 | 1 | 0 | 0 | 0 | 1 | 56 | 146.5 |
| 1176 | 1009 | 0 | 1456.8 | 0 | 0 | 201.8 | 34.2 | 0 | 0 | 0 | 0 | 0 | 0.200 | 1 | 23 | 1 | 1 | 0 | 0 | 0 | 1 | 56 | 148.9 |
| 1177 | 958.55 | 50.45 | 1456.8 | 0 | 0 | 201.8 | 34.2 | 0 | 0 | 0 | 0 | 0 | 0.200 | 1 | 23 | 1 | 1 | 0 | 0 | 0 | 1 | 56 | 149 |
| 1178 | 797.78 | 112.36 | 1123.63 | 0 | 0 | 224.73 | 28.09 | 15.9556 | 44.95 | 112.36 | 56.18 | 0 | 0.247 | 1 | 23 | 1 | 1 | 5 | 12 | 0.01 | 1 | 1 | 70.5 |
| 1179 | 756.75 | 50.45 | 1456.8 | 0 | 0 | 201.8 | 34.2 | 0 | 201.8 | 0 | 0 | 0 | 0.250 | 1 | 23 | 1 | 1 | 0 | 0 | 0 | 1 | 56 | 150.7 |
| 1180 | 890 | 0 | 1401.2 | 0 | 0 | 178 | 30.2 | 0 | 0 | 0 | 0 | 0 | 0.200 | 1 | 23 | 1 | 1 | 0 | 0 | 0 | 1 | 56 | 124.5 |
| 1181 | 890 | 0 | 1401.2 | 0 | 0 | 178 | 30.2 | 0 | 0 | 0 | 0 | 0 | 0.200 | 1 | 23 | 1 | 1 | 0 | 0 | 0 | 1 | 56 | 130.7 |
| 1182 | 845.5 | 44.5 | 1401.2 | 0 | 0 | 178 | 30.2 | 0 | 0 | 0 | 0 | 0 | 0.200 | 1 | 23 | 1 | 1 | 0 | 0 | 0 | 1 | 56 | 138.4 |
| 1183 | 801 | 89 | 1401.2 | 0 | 0 | 178 | 30.2 | 0 | 0 | 0 | 0 | 0 | 0.200 | 1 | 23 | 1 | 1 | 0 | 0 | 0 | 1 | 56 | 146.6 |
| 1184 | 756.5 | 133.5 | 1401.2 | 0 | 0 | 178 | 30.2 | 0 | 0 | 0 | 0 | 0 | 0.200 | 1 | 23 | 1 | 1 | 0 | 0 | 0 | 1 | 56 | 138 |
| 1185 | 712 | 178 | 1401.2 | 0 | 0 | 178 | 30.2 | 0 | 0 | 0 | 0 | 0 | 0.200 | 1 | 23 | 1 | 1 | 0 | 0 | 0 | 1 | 56 | 136.4 |
| 1186 | 845.5 | 44.5 | 1401.2 | 0 | 0 | 178 | 30.2 | 0 | 0 | 0 | 0 | 0 | 0.200 | 1 | 23 | 1 | 1 | 0 | 0 | 0 | 1 | 56 | 143.5 |
| 1187 | 712 | 0 | 1401.2 | 0 | 0 | 178 | 30.2 | 0 | 178 | 0 | 0 | 0 | 0.250 | 1 | 23 | 1 | 1 | 0 | 0 | 0 | 1 | 56 | 127.5 |
| 1188 | 623 | 0 | 1401.2 | 0 | 0 | 178 | 30.2 | 0 | 267 | 0 | 0 | 0 | 0.286 | 1 | 23 | 1 | 1 | 0 | 0 | 0 | 1 | 56 | 134.9 |
| 1189 | 797.78 | 112.36 | 1123.63 | 0 | 0 | 224.73 | 28.09 | 15.9556 | 44.95 | 112.36 | 56.18 | 0 | 0.247 | 1 | 23 | 1 | 1 | 5 | 12 | 0.01 | 1 | 14 | 108 |
| 1190 | 534 | 0 | 1401.2 | 0 | 0 | 178 | 30.2 | 0 | 356 | 0 | 0 | 0 | 0.333 | 1 | 23 | 1 | 1 | 0 | 0 | 0 | 1 | 56 | 128.2 |
| 1191 | 667.5 | 44.5 | 1401.2 | 0 | 0 | 178 | 30.2 | 0 | 178 | 0 | 0 | 0 | 0.250 | 1 | 23 | 1 | 1 | 0 | 0 | 0 | 1 | 56 | 131.4 |
| 1192 | 1009 | 0 | 1456.8 | 0 | 0 | 201.8 | 34.2 | 0 | 0 | 0 | 0 | 0 | 0.200 | 1 | 23 | 1 | 1 | 0 | 0 | 0 | 1 | 56 | 146.7 |
| 1193 | 1009 | 0 | 1456.8 | 0 | 0 | 201.8 | 34.2 | 0 | 0 | 0 | 0 | 0 | 0.200 | 1 | 23 | 1 | 1 | 0 | 0 | 0 | 1 | 56 | 149 |
| 1194 | 958.55 | 50.45 | 1456.8 | 0 | 0 | 201.8 | 34.2 | 0 | 0 | 0 | 0 | 0 | 0.200 | 1 | 23 | 1 | 1 | 0 | 0 | 0 | 1 | 56 | 148.4 |
| 1195 | 756.75 | 50.45 | 1456.8 | 0 | 0 | 201.8 | 34.2 | 0 | 201.8 | 0 | 0 | 0 | 0.250 | 1 | 23 | 1 | 1 | 0 | 0 | 0 | 1 | 56 | 152.5 |
| 1196 | 890 | 0 | 1401.2 | 0 | 0 | 178 | 30.2 | 0 | 0 | 0 | 0 | 0 | 0.200 | 1 | 23 | 1 | 1 | 0 | 0 | 0 | 1 | 56 | 126.8 |
| 1197 | 890 | 0 | 1401.2 | 0 | 0 | 178 | 30.2 | 0 | 0 | 0 | 0 | 0 | 0.200 | 1 | 23 | 1 | 1 | 0 | 0 | 0 | 1 | 56 | 132.1 |
| 1198 | 845.5 | 44.5 | 1401.2 | 0 | 0 | 178 | 30.2 | 0 | 0 | 0 | 0 | 0 | 0.200 | 1 | 23 | 1 | 1 | 0 | 0 | 0 | 1 | 56 | 138.8 |
| 1199 | 801 | 89 | 1401.2 | 0 | 0 | 178 | 30.2 | 0 | 0 | 0 | 0 | 0 | 0.200 | 1 | 23 | 1 | 1 | 0 | 0 | 0 | 1 | 56 | 147 |
| 1200 | 797.78 | 112.36 | 1123.63 | 0 | 0 | 224.73 | 28.09 | 15.9556 | 44.95 | 112.36 | 56.18 | 0 | 0.247 | 1 | 23 | 1 | 1 | 5 | 12 | 0.01 | 1 | 28 | 120 |
| 1201 | 756.5 | 133.5 | 1401.2 | 0 | 0 | 178 | 30.2 | 0 | 0 | 0 | 0 | 0 | 0.200 | 1 | 23 | 1 | 1 | 0 | 0 | 0 | 1 | 56 | 139.4 |
| 1202 | 712 | 178 | 1401.2 | 0 | 0 | 178 | 30.2 | 0 | 0 | 0 | 0 | 0 | 0.200 | 1 | 23 | 1 | 1 | 0 | 0 | 0 | 1 | 56 | 134.2 |
| 1203 | 845.5 | 44.5 | 1401.2 | 0 | 0 | 178 | 30.2 | 0 | 0 | 0 | 0 | 0 | 0.200 | 1 | 23 | 1 | 1 | 0 | 0 | 0 | 1 | 56 | 143 |
| 1204 | 712 | 0 | 1401.2 | 0 | 0 | 178 | 30.2 | 0 | 178 | 0 | 0 | 0 | 0.250 | 1 | 23 | 1 | 1 | 0 | 0 | 0 | 1 | 56 | 127.5 |
| 1205 | 623 | 0 | 1401.2 | 0 | 0 | 178 | 30.2 | 0 | 267 | 0 | 0 | 0 | 0.286 | 1 | 23 | 1 | 1 | 0 | 0 | 0 | 1 | 56 | 135.9 |
| 1206 | 534 | 0 | 1401.2 | 0 | 0 | 178 | 30.2 | 0 | 356 | 0 | 0 | 0 | 0.333 | 1 | 23 | 1 | 1 | 0 | 0 | 0 | 1 | 56 | 129.5 |
| 1207 | 667.5 | 44.5 | 1401.2 | 0 | 0 | 178 | 30.2 | 0 | 178 | 0 | 0 | 0 | 0.250 | 1 | 23 | 1 | 1 | 0 | 0 | 0 | 1 | 56 | 132.5 |
| 1208 | 1009 | 0 | 1456.8 | 0 | 0 | 201.8 | 34.2 | 0 | 0 | 0 | 0 | 0 | 0.200 | 1 | 23 | 1 | 1 | 0 | 0 | 0 | 1 | 56 | 144.1 |
| 1209 | 1009 | 0 | 1456.8 | 0 | 0 | 201.8 | 34.2 | 0 | 0 | 0 | 0 | 0 | 0.200 | 1 | 23 | 1 | 1 | 0 | 0 | 0 | 1 | 56 | 150.2 |
| 1210 | 958.55 | 50.45 | 1456.8 | 0 | 0 | 201.8 | 34.2 | 0 | 0 | 0 | 0 | 0 | 0.200 | 1 | 23 | 1 | 1 | 0 | 0 | 0 | 1 | 56 | 149.6 |
| 1211 | 797.78 | 112.36 | 1123.63 | 0 | 0 | 224.73 | 28.09 | 19.9445 | 44.95 | 112.36 | 56.18 | 0 | 0.247 | 1 | 23 | 1 | 1 | 5 | 12 | 0.01 | 1 | 1 | 70.2 |
| 1212 | 756.75 | 50.45 | 1456.8 | 0 | 0 | 201.8 | 34.2 | 0 | 201.8 | 0 | 0 | 0 | 0.250 | 1 | 23 | 1 | 1 | 0 | 0 | 0 | 1 | 56 | 153.2 |
| 1213 | 890 | 0 | 1401.2 | 0 | 0 | 178 | 30.2 | 0 | 0 | 0 | 0 | 0 | 0.200 | 1 | 23 | 1 | 1 | 0 | 0 | 0 | 1 | 90 | 133.1 |
| 1214 | 890 | 0 | 1401.2 | 0 | 0 | 178 | 30.2 | 0 | 0 | 0 | 0 | 0 | 0.200 | 1 | 23 | 1 | 1 | 0 | 0 | 0 | 1 | 90 | 136.1 |
| 1215 | 845.5 | 44.5 | 1401.2 | 0 | 0 | 178 | 30.2 | 0 | 0 | 0 | 0 | 0 | 0.200 | 1 | 23 | 1 | 1 | 0 | 0 | 0 | 1 | 90 | 145.8 |
| 1216 | 801 | 89 | 1401.2 | 0 | 0 | 178 | 30.2 | 0 | 0 | 0 | 0 | 0 | 0.200 | 1 | 23 | 1 | 1 | 0 | 0 | 0 | 1 | 90 | 148.2 |
| 1217 | 756.5 | 133.5 | 1401.2 | 0 | 0 | 178 | 30.2 | 0 | 0 | 0 | 0 | 0 | 0.200 | 1 | 23 | 1 | 1 | 0 | 0 | 0 | 1 | 90 | 138.9 |
| 1218 | 712 | 178 | 1401.2 | 0 | 0 | 178 | 30.2 | 0 | 0 | 0 | 0 | 0 | 0.200 | 1 | 23 | 1 | 1 | 0 | 0 | 0 | 1 | 90 | 136.8 |
| 1219 | 845.5 | 44.5 | 1401.2 | 0 | 0 | 178 | 30.2 | 0 | 0 | 0 | 0 | 0 | 0.200 | 1 | 23 | 1 | 1 | 0 | 0 | 0 | 1 | 90 | 146.8 |
| 1220 | 712 | 0 | 1401.2 | 0 | 0 | 178 | 30.2 | 0 | 178 | 0 | 0 | 0 | 0.250 | 1 | 23 | 1 | 1 | 0 | 0 | 0 | 1 | 90 | 133.6 |
| 1221 | 623 | 0 | 1401.2 | 0 | 0 | 178 | 30.2 | 0 | 267 | 0 | 0 | 0 | 0.286 | 1 | 23 | 1 | 1 | 0 | 0 | 0 | 1 | 90 | 136.1 |
| 1222 | 797.78 | 112.36 | 1123.63 | 0 | 0 | 224.73 | 28.09 | 19.9445 | 44.95 | 112.36 | 56.18 | 0 | 0.247 | 1 | 23 | 1 | 1 | 5 | 12 | 0.01 | 1 | 14 | 106.4 |
| 1223 | 534 | 0 | 1401.2 | 0 | 0 | 178 | 30.2 | 0 | 356 | 0 | 0 | 0 | 0.333 | 1 | 23 | 1 | 1 | 0 | 0 | 0 | 1 | 90 | 138.4 |
| 1224 | 667.5 | 44.5 | 1401.2 | 0 | 0 | 178 | 30.2 | 0 | 178 | 0 | 0 | 0 | 0.250 | 1 | 23 | 1 | 1 | 0 | 0 | 0 | 1 | 90 | 140.7 |
| 1225 | 1009 | 0 | 1456.8 | 0 | 0 | 201.8 | 34.2 | 0 | 0 | 0 | 0 | 0 | 0.200 | 1 | 23 | 1 | 1 | 0 | 0 | 0 | 1 | 90 | 159.6 |
| 1226 | 1009 | 0 | 1456.8 | 0 | 0 | 201.8 | 34.2 | 0 | 0 | 0 | 0 | 0 | 0.200 | 1 | 23 | 1 | 1 | 0 | 0 | 0 | 1 | 90 | 172 |
| 1227 | 958.55 | 50.45 | 1456.8 | 0 | 0 | 201.8 | 34.2 | 0 | 0 | 0 | 0 | 0 | 0.200 | 1 | 23 | 1 | 1 | 0 | 0 | 0 | 1 | 90 | 180.8 |
| 1228 | 756.75 | 50.45 | 1456.8 | 0 | 0 | 201.8 | 34.2 | 0 | 201.8 | 0 | 0 | 0 | 0.250 | 1 | 23 | 1 | 1 | 0 | 0 | 0 | 1 | 90 | 180.2 |
| 1229 | 890 | 0 | 1401.2 | 0 | 0 | 178 | 30.2 | 0 | 0 | 0 | 0 | 0 | 0.200 | 1 | 23 | 1 | 1 | 0 | 0 | 0 | 1 | 90 | 131.2 |
| 1230 | 890 | 0 | 1401.2 | 0 | 0 | 178 | 30.2 | 0 | 0 | 0 | 0 | 0 | 0.200 | 1 | 23 | 1 | 1 | 0 | 0 | 0 | 1 | 90 | 134.1 |
| 1231 | 845.5 | 44.5 | 1401.2 | 0 | 0 | 178 | 30.2 | 0 | 0 | 0 | 0 | 0 | 0.200 | 1 | 23 | 1 | 1 | 0 | 0 | 0 | 1 | 90 | 143.7 |
| 1232 | 801 | 89 | 1401.2 | 0 | 0 | 178 | 30.2 | 0 | 0 | 0 | 0 | 0 | 0.200 | 1 | 23 | 1 | 1 | 0 | 0 | 0 | 1 | 90 | 148 |
| 1233 | 797.78 | 112.36 | 1123.63 | 0 | 0 | 224.73 | 28.09 | 19.9445 | 44.95 | 112.36 | 56.18 | 0 | 0.247 | 1 | 23 | 1 | 1 | 5 | 12 | 0.01 | 1 | 28 | 110.9 |
| 1234 | 756.5 | 133.5 | 1401.2 | 0 | 0 | 178 | 30.2 | 0 | 0 | 0 | 0 | 0 | 0.200 | 1 | 23 | 1 | 1 | 0 | 0 | 0 | 1 | 90 | 138.4 |
| 1235 | 712 | 178 | 1401.2 | 0 | 0 | 178 | 30.2 | 0 | 0 | 0 | 0 | 0 | 0.200 | 1 | 23 | 1 | 1 | 0 | 0 | 0 | 1 | 90 | 136.3 |
| 1236 | 845.5 | 44.5 | 1401.2 | 0 | 0 | 178 | 30.2 | 0 | 0 | 0 | 0 | 0 | 0.200 | 1 | 23 | 1 | 1 | 0 | 0 | 0 | 1 | 90 | 144.8 |
| 1237 | 712 | 0 | 1401.2 | 0 | 0 | 178 | 30.2 | 0 | 178 | 0 | 0 | 0 | 0.250 | 1 | 23 | 1 | 1 | 0 | 0 | 0 | 1 | 90 | 136.8 |
| 1238 | 623 | 0 | 1401.2 | 0 | 0 | 178 | 30.2 | 0 | 267 | 0 | 0 | 0 | 0.286 | 1 | 23 | 1 | 1 | 0 | 0 | 0 | 1 | 90 | 138 |
| 1239 | 534 | 0 | 1401.2 | 0 | 0 | 178 | 30.2 | 0 | 356 | 0 | 0 | 0 | 0.333 | 1 | 23 | 1 | 1 | 0 | 0 | 0 | 1 | 90 | 139.6 |
| 1240 | 667.5 | 44.5 | 1401.2 | 0 | 0 | 178 | 30.2 | 0 | 178 | 0 | 0 | 0 | 0.250 | 1 | 23 | 1 | 1 | 0 | 0 | 0 | 1 | 90 | 138.6 |
| 1241 | 1009 | 0 | 1456.8 | 0 | 0 | 201.8 | 34.2 | 0 | 0 | 0 | 0 | 0 | 0.200 | 1 | 23 | 1 | 1 | 0 | 0 | 0 | 1 | 90 | 163.1 |
| 1242 | 1009 | 0 | 1456.8 | 0 | 0 | 201.8 | 34.2 | 0 | 0 | 0 | 0 | 0 | 0.200 | 1 | 23 | 1 | 1 | 0 | 0 | 0 | 1 | 90 | 175.3 |
| 1243 | 958.55 | 50.45 | 1456.8 | 0 | 0 | 201.8 | 34.2 | 0 | 0 | 0 | 0 | 0 | 0.200 | 1 | 23 | 1 | 1 | 0 | 0 | 0 | 1 | 90 | 180.1 |
| 1244 | 1120 | 145 | 1120 | 0 | 0 | 228 | 21 | 0 | 0 | 105 | 0 | 0 | 0.180 | 1 | 23 | 1 | 1 | 0 | 0 | 0 | 1 | 7 | 88.7 |
| 1245 | 756.75 | 50.45 | 1456.8 | 0 | 0 | 201.8 | 34.2 | 0 | 201.8 | 0 | 0 | 0 | 0.250 | 1 | 23 | 1 | 1 | 0 | 0 | 0 | 1 | 90 | 178.6 |
| 1246 | 890 | 0 | 1401.2 | 0 | 0 | 178 | 30.2 | 0 | 0 | 0 | 0 | 0 | 0.200 | 1 | 23 | 1 | 1 | 0 | 0 | 0 | 1 | 90 | 131.1 |
| 1247 | 890 | 0 | 1401.2 | 0 | 0 | 178 | 30.2 | 0 | 0 | 0 | 0 | 0 | 0.200 | 1 | 23 | 1 | 1 | 0 | 0 | 0 | 1 | 90 | 139.1 |
| 1248 | 845.5 | 44.5 | 1401.2 | 0 | 0 | 178 | 30.2 | 0 | 0 | 0 | 0 | 0 | 0.200 | 1 | 23 | 1 | 1 | 0 | 0 | 0 | 1 | 90 | 145.1 |
| 1249 | 801 | 89 | 1401.2 | 0 | 0 | 178 | 30.2 | 0 | 0 | 0 | 0 | 0 | 0.200 | 1 | 23 | 1 | 1 | 0 | 0 | 0 | 1 | 90 | 146 |
| 1250 | 756.5 | 133.5 | 1401.2 | 0 | 0 | 178 | 30.2 | 0 | 0 | 0 | 0 | 0 | 0.200 | 1 | 23 | 1 | 1 | 0 | 0 | 0 | 1 | 90 | 141.6 |
| 1251 | 712 | 178 | 1401.2 | 0 | 0 | 178 | 30.2 | 0 | 0 | 0 | 0 | 0 | 0.200 | 1 | 23 | 1 | 1 | 0 | 0 | 0 | 1 | 90 | 137.9 |
| 1252 | 845.5 | 44.5 | 1401.2 | 0 | 0 | 178 | 30.2 | 0 | 0 | 0 | 0 | 0 | 0.200 | 1 | 23 | 1 | 1 | 0 | 0 | 0 | 1 | 90 | 145.6 |
| 1253 | 712 | 0 | 1401.2 | 0 | 0 | 178 | 30.2 | 0 | 178 | 0 | 0 | 0 | 0.250 | 1 | 23 | 1 | 1 | 0 | 0 | 0 | 1 | 90 | 136.8 |
| 1254 | 623 | 0 | 1401.2 | 0 | 0 | 178 | 30.2 | 0 | 267 | 0 | 0 | 0 | 0.286 | 1 | 23 | 1 | 1 | 0 | 0 | 0 | 1 | 90 | 139.3 |
| 1255 | 797.78 | 112.36 | 1123.63 | 0 | 0 | 224.73 | 28.09 | 3.9889 | 44.95 | 112.36 | 56.18 | 0 | 0.247 | 1 | 23 | 1 | 1 | 4 | 18 | 0.013 | 1 | 14 | 111.5 |
| 1256 | 1120 | 145 | 1120 | 0 | 0 | 228 | 21 | 2.8 | 0 | 105 | 0 | 0 | 0.180 | 1 | 23 | 1 | 1 | 4 | 12 | 0.45 | 1 | 7 | 107.5 |
| 1257 | 534 | 0 | 1401.2 | 0 | 0 | 178 | 30.2 | 0 | 356 | 0 | 0 | 0 | 0.333 | 1 | 23 | 1 | 1 | 0 | 0 | 0 | 1 | 90 | 134.7 |
| 1258 | 667.5 | 44.5 | 1401.2 | 0 | 0 | 178 | 30.2 | 0 | 178 | 0 | 0 | 0 | 0.250 | 1 | 23 | 1 | 1 | 0 | 0 | 0 | 1 | 90 | 135.5 |
| 1259 | 1009 | 0 | 1456.8 | 0 | 0 | 201.8 | 34.2 | 0 | 0 | 0 | 0 | 0 | 0.200 | 1 | 23 | 1 | 1 | 0 | 0 | 0 | 1 | 90 | 160 |
| 1260 | 1009 | 0 | 1456.8 | 0 | 0 | 201.8 | 34.2 | 0 | 0 | 0 | 0 | 0 | 0.200 | 1 | 23 | 1 | 1 | 0 | 0 | 0 | 1 | 90 | 171.4 |
| 1261 | 958.55 | 50.45 | 1456.8 | 0 | 0 | 201.8 | 34.2 | 0 | 0 | 0 | 0 | 0 | 0.200 | 1 | 23 | 1 | 1 | 0 | 0 | 0 | 1 | 90 | 176.9 |
| 1262 | 756.75 | 50.45 | 1456.8 | 0 | 0 | 201.8 | 34.2 | 0 | 201.8 | 0 | 0 | 0 | 0.250 | 1 | 23 | 1 | 1 | 0 | 0 | 0 | 1 | 90 | 168.3 |
| 1263 | 1076.77 | 286.23 | 1090.8 | 0 | 0 | 272.6 | 46.2 | 0 | 0 | 0 | 0 | 0 | 0.200 | 1 | 23 | 1 | 1 | 0 | 0 | 0 | 1 | 28 | 145.4 |
| 1264 | 1076.77 | 286.23 | 1090.8 | 0 | 0 | 272.6 | 46.2 | 32.3031 | 0 | 0 | 0 | 0 | 0.200 | 1 | 23 | 1 | 1 | 1 | 12.7 | 0.2 | 1 | 28 | 158.2 |
| 1265 | 1076.77 | 286.23 | 1090.8 | 0 | 0 | 272.6 | 46.2 | 0 | 0 | 0 | 0 | 0 | 0.200 | 1 | 23 | 1 | 1 | 0 | 0 | 0 | 1 | 28 | 128.7 |
| 1266 | 1076.77 | 286.23 | 1090.8 | 0 | 0 | 272.6 | 46.2 | 32.3031 | 0 | 0 | 0 | 0 | 0.200 | 1 | 23 | 1 | 1 | 1 | 12.7 | 0.2 | 1 | 28 | 168.1 |
| 1267 | 1120 | 145 | 1120 | 0 | 0 | 228 | 21 | 5.6 | 0 | 105 | 0 | 0 | 0.180 | 1 | 23 | 1 | 1 | 4 | 12 | 0.45 | 1 | 7 | 116 |
| 1268 | 1076.77 | 286.23 | 1090.8 | 0 | 0 | 272.6 | 46.2 | 0 | 0 | 0 | 0 | 0 | 0.200 | 1 | 23 | 1 | 1 | 0 | 0 | 0 | 1 | 28 | 184.5 |
| 1269 | 1076.77 | 286.23 | 1090.8 | 0 | 0 | 272.6 | 46.2 | 32.3031 | 0 | 0 | 0 | 0 | 0.200 | 1 | 23 | 1 | 1 | 1 | 12.7 | 0.2 | 1 | 28 | 197 |
| 1270 | 1076.77 | 286.23 | 1090.8 | 0 | 0 | 272.6 | 46.2 | 0 | 0 | 0 | 0 | 0 | 0.200 | 1 | 23 | 1 | 1 | 0 | 0 | 0 | 1 | 28 | 160.6 |
| 1271 | 1076.77 | 286.23 | 1090.8 | 0 | 0 | 272.6 | 46.2 | 32.3031 | 0 | 0 | 0 | 0 | 0.200 | 1 | 23 | 1 | 1 | 1 | 12.7 | 0.2 | 1 | 28 | 174.5 |
| 1272 | 1076.77 | 286.23 | 1090.8 | 0 | 0 | 272.6 | 46.2 | 0 | 0 | 0 | 0 | 0 | 0.200 | 1 | 23 | 1 | 1 | 0 | 0 | 0 | 1 | 28 | 191.5 |
| 1273 | 1076.77 | 286.23 | 1090.8 | 0 | 0 | 272.6 | 46.2 | 32.3031 | 0 | 0 | 0 | 0 | 0.200 | 1 | 23 | 1 | 1 | 1 | 12.7 | 0.2 | 1 | 28 | 206.7 |
| 1274 | 1076.77 | 286.23 | 1090.8 | 0 | 0 | 272.6 | 46.2 | 0 | 0 | 0 | 0 | 0 | 0.200 | 1 | 23 | 1 | 1 | 0 | 0 | 0 | 1 | 28 | 165.6 |
| 1275 | 1076.77 | 286.23 | 1090.8 | 0 | 0 | 272.6 | 46.2 | 32.3031 | 0 | 0 | 0 | 0 | 0.200 | 1 | 23 | 1 | 1 | 1 | 12.7 | 0.2 | 1 | 28 | 172.5 |
| 1276 | 1076.77 | 286.23 | 1090.8 | 0 | 0 | 272.6 | 46.2 | 0 | 0 | 0 | 0 | 0 | 0.200 | 1 | 23 | 1 | 1 | 0 | 0 | 0 | 1 | 28 | 189.3 |
| 1277 | 1076.77 | 286.23 | 1090.8 | 0 | 0 | 272.6 | 46.2 | 32.3031 | 0 | 0 | 0 | 0 | 0.200 | 1 | 23 | 1 | 1 | 1 | 12.7 | 0.2 | 1 | 28 | 204.4 |
| 1278 | 1120 | 145 | 1120 | 0 | 0 | 228 | 21 | 8.4 | 0 | 105 | 0 | 0 | 0.180 | 1 | 23 | 1 | 1 | 4 | 12 | 0.45 | 1 | 7 | 112.5 |
| 1279 | 1076.77 | 286.23 | 1090.8 | 0 | 0 | 272.6 | 46.2 | 0 | 0 | 0 | 0 | 0 | 0.200 | 1 | 23 | 1 | 1 | 0 | 0 | 0 | 1 | 28 | 164.6 |
| 1280 | 1076.77 | 286.23 | 1090.8 | 0 | 0 | 272.6 | 46.2 | 32.3031 | 0 | 0 | 0 | 0 | 0.200 | 1 | 23 | 1 | 1 | 1 | 12.7 | 0.2 | 1 | 28 | 135.1 |
| 1281 | 960 | 240 | 793.7 | 0 | 0 | 234 | 45 | 0 | 0 | 0 | 0 | 0 | 0.195 | 1 | 23 | 1 | 1 | 0 | 6 | 0.16 | 1 | 7 | 124 |
| 1282 | 960 | 240 | 787.1 | 0 | 0 | 234 | 45 | 2.4 | 0 | 0 | 0 | 0 | 0.195 | 1 | 23 | 1 | 1 | 1 | 6 | 0.16 | 1 | 7 | 137.7 |
| 1283 | 960 | 240 | 780.5 | 0 | 0 | 234 | 45 | 4.8 | 0 | 0 | 0 | 0 | 0.195 | 1 | 23 | 1 | 1 | 1 | 6 | 0.16 | 1 | 7 | 140.6 |
| 1284 | 960 | 240 | 773.8 | 0 | 0 | 234 | 45 | 7.2 | 0 | 0 | 0 | 0 | 0.195 | 1 | 23 | 1 | 1 | 1 | 6 | 0.16 | 1 | 7 | 143.5 |
| 1285 | 960 | 240 | 767.2 | 0 | 0 | 234 | 45 | 9.6 | 0 | 0 | 0 | 0 | 0.195 | 1 | 23 | 1 | 1 | 1 | 6 | 0.16 | 1 | 7 | 146.7 |
| 1286 | 960 | 240 | 754 | 0 | 0 | 234 | 45 | 14.4 | 0 | 0 | 0 | 0 | 0.195 | 1 | 23 | 1 | 1 | 1 | 6 | 0.16 | 1 | 7 | 149.2 |
| 1287 | 960 | 240 | 740.7 | 0 | 0 | 234 | 45 | 19.2 | 0 | 0 | 0 | 0 | 0.195 | 1 | 23 | 1 | 1 | 1 | 6 | 0.16 | 1 | 7 | 157.8 |
| 1288 | 960 | 240 | 793.7 | 0 | 0 | 234 | 45 | 0 | 0 | 0 | 0 | 0 | 0.195 | 1 | 23 | 1 | 1 | 0 | 6 | 0.16 | 1 | 28 | 157.8 |
| 1289 | 1120 | 145 | 1120 | 0 | 0 | 228 | 21 | 11.2 | 0 | 105 | 0 | 0 | 0.180 | 1 | 23 | 1 | 1 | 4 | 12 | 0.45 | 1 | 7 | 119.9 |
| 1290 | 960 | 240 | 787.1 | 0 | 0 | 234 | 45 | 2.4 | 0 | 0 | 0 | 0 | 0.195 | 1 | 23 | 1 | 1 | 1 | 6 | 0.16 | 1 | 28 | 172.1 |
| 1291 | 960 | 240 | 780.5 | 0 | 0 | 234 | 45 | 4.8 | 0 | 0 | 0 | 0 | 0.195 | 1 | 23 | 1 | 1 | 1 | 6 | 0.16 | 1 | 28 | 173.6 |
| 1292 | 960 | 240 | 773.8 | 0 | 0 | 234 | 45 | 7.2 | 0 | 0 | 0 | 0 | 0.195 | 1 | 23 | 1 | 1 | 1 | 6 | 0.16 | 1 | 28 | 176.1 |
| 1293 | 960 | 240 | 767.2 | 0 | 0 | 234 | 45 | 9.6 | 0 | 0 | 0 | 0 | 0.195 | 1 | 23 | 1 | 1 | 1 | 6 | 0.16 | 1 | 28 | 178.9 |
| 1294 | 960 | 240 | 754 | 0 | 0 | 234 | 45 | 14.4 | 0 | 0 | 0 | 0 | 0.195 | 1 | 23 | 1 | 1 | 1 | 6 | 0.16 | 1 | 28 | 185.4 |
| 1295 | 960 | 240 | 740.7 | 0 | 0 | 234 | 45 | 19.2 | 0 | 0 | 0 | 0 | 0.195 | 1 | 23 | 1 | 1 | 1 | 6 | 0.16 | 1 | 28 | 188.3 |
| 1296 | 960 | 240 | 793.7 | 0 | 0 | 234 | 45 | 0 | 0 | 0 | 0 | 0 | 0.195 | 1 | 23 | 1 | 1 | 0 | 6 | 0.16 | 1 | 56 | 173.6 |
| 1297 | 960 | 240 | 787.1 | 0 | 0 | 234 | 45 | 2.4 | 0 | 0 | 0 | 0 | 0.195 | 1 | 23 | 1 | 1 | 1 | 6 | 0.16 | 1 | 56 | 188.6 |
| 1298 | 960 | 240 | 780.5 | 0 | 0 | 234 | 45 | 4.8 | 0 | 0 | 0 | 0 | 0.195 | 1 | 23 | 1 | 1 | 1 | 6 | 0.16 | 1 | 56 | 191.8 |
| 1299 | 960 | 240 | 773.8 | 0 | 0 | 234 | 45 | 7.2 | 0 | 0 | 0 | 0 | 0.195 | 1 | 23 | 1 | 1 | 1 | 6 | 0.16 | 1 | 56 | 193.6 |
| 1300 | 1120 | 145 | 1120 | 0 | 0 | 228 | 21 | 16.8 | 0 | 105 | 0 | 0 | 0.180 | 1 | 23 | 1 | 1 | 4 | 12 | 0.45 | 1 | 7 | 116.1 |
| 1301 | 960 | 240 | 767.2 | 0 | 0 | 234 | 45 | 9.6 | 0 | 0 | 0 | 0 | 0.195 | 1 | 23 | 1 | 1 | 1 | 6 | 0.16 | 1 | 56 | 197.2 |
| 1302 | 960 | 240 | 754 | 0 | 0 | 234 | 45 | 14.4 | 0 | 0 | 0 | 0 | 0.195 | 1 | 23 | 1 | 1 | 1 | 6 | 0.16 | 1 | 56 | 199.4 |
| 1303 | 960 | 240 | 740.7 | 0 | 0 | 234 | 45 | 19.2 | 0 | 0 | 0 | 0 | 0.195 | 1 | 23 | 1 | 1 | 1 | 6 | 0.16 | 1 | 56 | 200.1 |
| 1304 | 960 | 240 | 793.7 | 0 | 0 | 234 | 45 | 0 | 0 | 0 | 0 | 0 | 0.195 | 1 | 23 | 1 | 1 | 0 | 6 | 0.16 | 1 | 90 | 180.4 |
| 1305 | 960 | 240 | 787.1 | 0 | 0 | 234 | 45 | 2.4 | 0 | 0 | 0 | 0 | 0.195 | 1 | 23 | 1 | 1 | 1 | 6 | 0.16 | 1 | 90 | 193.6 |
| 1306 | 960 | 240 | 780.5 | 0 | 0 | 234 | 45 | 4.8 | 0 | 0 | 0 | 0 | 0.195 | 1 | 23 | 1 | 1 | 1 | 6 | 0.16 | 1 | 90 | 195.4 |
| 1307 | 960 | 240 | 773.8 | 0 | 0 | 234 | 45 | 7.2 | 0 | 0 | 0 | 0 | 0.195 | 1 | 23 | 1 | 1 | 1 | 6 | 0.16 | 1 | 90 | 197.2 |
| 1308 | 960 | 240 | 767.2 | 0 | 0 | 234 | 45 | 9.6 | 0 | 0 | 0 | 0 | 0.195 | 1 | 23 | 1 | 1 | 1 | 6 | 0.16 | 1 | 90 | 200.8 |
| 1309 | 960 | 240 | 754 | 0 | 0 | 234 | 45 | 14.4 | 0 | 0 | 0 | 0 | 0.195 | 1 | 23 | 1 | 1 | 1 | 6 | 0.16 | 1 | 90 | 204.4 |
| 1310 | 960 | 240 | 740.7 | 0 | 0 | 234 | 45 | 19.2 | 0 | 0 | 0 | 0 | 0.195 | 1 | 23 | 1 | 1 | 1 | 6 | 0.16 | 1 | 90 | 208.7 |
| 1311 | 1120 | 145 | 1120 | 0 | 0 | 228 | 21 | 2.8 | 0 | 105 | 0 | 0 | 0.180 | 1 | 23 | 1 | 1 | 1 | 12 | 0.3 | 1 | 7 | 111.4 |
| 1312 | 1116.7 | 390.85 | 625.35 | 0 | 0 | 212.17 | 23.45 | 0 | 0 | 0 | 0 | 0 | 0.141 | 1 | 23 | 1 | 1 | 0 | 0 | 0 | 1 | 28 | 160.5 |
| 1313 | 1116.7 | 390.85 | 625.35 | 0 | 0 | 212.17 | 23.45 | 0 | 0 | 0 | 0 | 0 | 0.141 | 1 | 23 | 1 | 1 | 0 | 0 | 0 | 1 | 42 | 189.4 |
| 1314 | 1116.7 | 390.85 | 625.35 | 0 | 0 | 212.17 | 23.45 | 22.334 | 0 | 0 | 0 | 0 | 0.141 | 1 | 23 | 1 | 1 | 1 | 13 | 0.2 | 1 | 28 | 169.8 |
| 1315 | 1116.7 | 390.85 | 625.35 | 0 | 0 | 212.17 | 23.45 | 22.334 | 0 | 0 | 0 | 0 | 0.141 | 1 | 23 | 1 | 1 | 1 | 13 | 0.2 | 1 | 42 | 196.4 |
| 1316 | 1116.7 | 390.85 | 625.35 | 0 | 0 | 212.17 | 23.45 | 33.501 | 0 | 0 | 0 | 0 | 0.141 | 1 | 23 | 1 | 1 | 1 | 13 | 0.2 | 1 | 28 | 174.4 |
| 1317 | 1116.7 | 390.85 | 625.35 | 0 | 0 | 212.17 | 23.45 | 33.501 | 0 | 0 | 0 | 0 | 0.141 | 1 | 23 | 1 | 1 | 1 | 13 | 0.2 | 1 | 42 | 198.7 |
| 1318 | 1116.7 | 390.85 | 625.35 | 0 | 0 | 212.17 | 23.45 | 44.668 | 0 | 0 | 0 | 0 | 0.141 | 1 | 23 | 1 | 1 | 1 | 13 | 0.2 | 1 | 28 | 189.4 |
| 1319 | 1116.7 | 390.85 | 625.35 | 0 | 0 | 212.17 | 23.45 | 44.668 | 0 | 0 | 0 | 0 | 0.141 | 1 | 23 | 1 | 1 | 1 | 13 | 0.2 | 1 | 42 | 202.1 |
| 1320 | 1116.7 | 390.85 | 625.35 | 0 | 0 | 212.17 | 23.45 | 55.835 | 0 | 0 | 0 | 0 | 0.141 | 1 | 23 | 1 | 1 | 1 | 13 | 0.2 | 1 | 28 | 201 |
| 1321 | 1116.7 | 390.85 | 625.35 | 0 | 0 | 212.17 | 23.45 | 55.835 | 0 | 0 | 0 | 0 | 0.141 | 1 | 23 | 1 | 1 | 1 | 13 | 0.2 | 1 | 42 | 228.7 |
| 1322 | 1120 | 145 | 1120 | 0 | 0 | 228 | 21 | 5.6 | 0 | 105 | 0 | 0 | 0.180 | 1 | 23 | 1 | 1 | 1 | 12 | 0.3 | 1 | 7 | 114.2 |
| 1323 | 1116.7 | 390.85 | 625.35 | 0 | 0 | 212.17 | 23.45 | 0 | 0 | 0 | 0 | 0 | 0.141 | 1 | 23 | 1 | 1 | 0 | 0 | 0 | 1 | 28 | 143.5 |
| 1324 | 1116.7 | 390.85 | 625.35 | 0 | 0 | 212.17 | 23.45 | 0 | 0 | 0 | 0 | 0 | 0.141 | 1 | 23 | 1 | 1 | 0 | 0 | 0 | 1 | 28 | 140.4 |
| 1325 | 1116.7 | 390.85 | 625.35 | 0 | 0 | 212.17 | 23.45 | 0 | 0 | 0 | 0 | 0 | 0.141 | 1 | 23 | 1 | 1 | 0 | 0 | 0 | 1 | 28 | 146.5 |
| 1326 | 1116.7 | 390.85 | 625.35 | 0 | 0 | 212.17 | 23.45 | 22.334 | 0 | 0 | 0 | 0 | 0.141 | 1 | 23 | 1 | 1 | 1 | 13 | 0.2 | 1 | 28 | 140.5 |
| 1327 | 1116.7 | 390.85 | 625.35 | 0 | 0 | 212.17 | 23.45 | 44.668 | 0 | 0 | 0 | 0 | 0.141 | 1 | 23 | 1 | 1 | 1 | 13 | 0.2 | 1 | 28 | 167.5 |
| 1328 | 1116.7 | 390.85 | 625.35 | 0 | 0 | 212.17 | 23.45 | 22.334 | 0 | 0 | 0 | 0 | 0.141 | 1 | 23 | 1 | 1 | 1 | 13 | 0.2 | 1 | 28 | 141.1 |
| 1329 | 1116.7 | 390.85 | 625.35 | 0 | 0 | 212.17 | 23.45 | 44.668 | 0 | 0 | 0 | 0 | 0.141 | 1 | 23 | 1 | 1 | 1 | 13 | 0.2 | 1 | 28 | 129.6 |
| 1330 | 1116.7 | 390.85 | 625.35 | 0 | 0 | 212.17 | 23.45 | 22.334 | 0 | 0 | 0 | 0 | 0.141 | 1 | 23 | 1 | 1 | 1 | 13 | 0.2 | 1 | 28 | 148.1 |
| 1331 | 1116.7 | 390.85 | 625.35 | 0 | 0 | 212.17 | 23.45 | 44.668 | 0 | 0 | 0 | 0 | 0.141 | 1 | 23 | 1 | 1 | 1 | 13 | 0.2 | 1 | 28 | 110.7 |
| 1332 | 1116.7 | 390.85 | 625.35 | 0 | 0 | 212.17 | 23.45 | 0 | 0 | 0 | 0 | 0 | 0.141 | 1 | 23 | 1 | 1 | 0 | 0 | 0 | 1 | 7 | 120.2 |
| 1333 | 1120 | 145 | 1120 | 0 | 0 | 228 | 21 | 8.4 | 0 | 105 | 0 | 0 | 0.180 | 1 | 23 | 1 | 1 | 1 | 12 | 0.3 | 1 | 7 | 122.3 |
| 1334 | 1116.7 | 390.85 | 625.35 | 0 | 0 | 212.17 | 23.45 | 22.334 | 0 | 0 | 0 | 0 | 0.141 | 1 | 23 | 1 | 1 | 1 | 13 | 0.2 | 1 | 7 | 135.5 |
| 1335 | 1116.7 | 390.85 | 625.35 | 0 | 0 | 212.17 | 23.45 | 33.501 | 0 | 0 | 0 | 0 | 0.141 | 1 | 23 | 1 | 1 | 1 | 13 | 0.2 | 1 | 7 | 141.4 |
| 1336 | 1116.7 | 390.85 | 625.35 | 0 | 0 | 212.17 | 23.45 | 44.668 | 0 | 0 | 0 | 0 | 0.141 | 1 | 23 | 1 | 1 | 1 | 13 | 0.2 | 1 | 7 | 153.2 |
| 1337 | 1116.7 | 390.85 | 625.35 | 0 | 0 | 212.17 | 23.45 | 55.835 | 0 | 0 | 0 | 0 | 0.141 | 1 | 23 | 1 | 1 | 1 | 13 | 0.2 | 1 | 7 | 145 |
| 1338 | 1116.7 | 390.85 | 625.35 | 0 | 0 | 212.17 | 23.45 | 0 | 0 | 0 | 0 | 0 | 0.141 | 1 | 23 | 1 | 1 | 0 | 0 | 0 | 1 | 21 | 170.9 |
| 1339 | 1116.7 | 390.85 | 625.35 | 0 | 0 | 212.17 | 23.45 | 22.334 | 0 | 0 | 0 | 0 | 0.141 | 1 | 23 | 1 | 1 | 1 | 13 | 0.2 | 1 | 21 | 160.3 |
| 1340 | 1116.7 | 390.85 | 625.35 | 0 | 0 | 212.17 | 23.45 | 33.501 | 0 | 0 | 0 | 0 | 0.141 | 1 | 23 | 1 | 1 | 1 | 13 | 0.2 | 1 | 21 | 142.6 |
| 1341 | 1116.7 | 390.85 | 625.35 | 0 | 0 | 212.17 | 23.45 | 44.668 | 0 | 0 | 0 | 0 | 0.141 | 1 | 23 | 1 | 1 | 1 | 13 | 0.2 | 1 | 21 | 165 |
| 1342 | 1116.7 | 390.85 | 625.35 | 0 | 0 | 212.17 | 23.45 | 55.835 | 0 | 0 | 0 | 0 | 0.141 | 1 | 23 | 1 | 1 | 1 | 13 | 0.2 | 1 | 21 | 168.5 |
| 1343 | 1116.7 | 390.85 | 625.35 | 0 | 0 | 212.17 | 23.45 | 0 | 0 | 0 | 0 | 0 | 0.141 | 1 | 23 | 1 | 1 | 0 | 0 | 0 | 1 | 28 | 170.9 |
| 1344 | 1120 | 145 | 1120 | 0 | 0 | 228 | 21 | 11.2 | 0 | 105 | 0 | 0 | 0.180 | 1 | 23 | 1 | 1 | 1 | 12 | 0.3 | 1 | 7 | 130 |
| 1345 | 1116.7 | 390.85 | 625.35 | 0 | 0 | 212.17 | 23.45 | 22.334 | 0 | 0 | 0 | 0 | 0.141 | 1 | 23 | 1 | 1 | 1 | 13 | 0.2 | 1 | 28 | 163.8 |
| 1346 | 1116.7 | 390.85 | 625.35 | 0 | 0 | 212.17 | 23.45 | 33.501 | 0 | 0 | 0 | 0 | 0.141 | 1 | 23 | 1 | 1 | 1 | 13 | 0.2 | 1 | 28 | 175.6 |
| 1347 | 1116.7 | 390.85 | 625.35 | 0 | 0 | 212.17 | 23.45 | 44.668 | 0 | 0 | 0 | 0 | 0.141 | 1 | 23 | 1 | 1 | 1 | 13 | 0.2 | 1 | 28 | 173.3 |
| 1348 | 1116.7 | 390.85 | 625.35 | 0 | 0 | 212.17 | 23.45 | 55.835 | 0 | 0 | 0 | 0 | 0.141 | 1 | 23 | 1 | 1 | 1 | 13 | 0.2 | 1 | 28 | 196.8 |
| 1349 | 429 | 107.2 | 1072 | 0 | 0 | 192.96 | 19.3 | 7.5075 | 214.4 | 321.6 | 0 | 0 | 0.360 | 1 | 23 | 1 | 1 | 1 | 13 | 0.2 | 1 | 7 | 92.6 |
| 1350 | 429 | 107.2 | 1072 | 0 | 0 | 192.96 | 19.3 | 7.5075 | 214.4 | 321.6 | 0 | 0 | 0.360 | 1 | 23 | 1 | 1 | 1 | 13 | 0.2 | 1 | 7 | 96.1 |
| 1351 | 429 | 107.2 | 1072 | 0 | 0 | 192.96 | 19.3 | 7.5075 | 214.4 | 321.6 | 0 | 0 | 0.360 | 1 | 23 | 1 | 1 | 1 | 13 | 0.2 | 1 | 7 | 99.6 |
| 1352 | 429 | 107.2 | 1072 | 0 | 0 | 192.96 | 19.3 | 7.5075 | 214.4 | 321.6 | 0 | 0 | 0.360 | 1 | 23 | 1 | 1 | 1 | 13 | 0.2 | 1 | 28 | 95.6 |
| 1353 | 429 | 107.2 | 1072 | 0 | 0 | 192.96 | 19.3 | 0 | 214.4 | 321.6 | 0 | 0 | 0.360 | 1 | 23 | 1 | 1 | 0 | 0 | 0 | 1 | 28 | 96.9 |
| 1354 | 429 | 107.2 | 1072 | 0 | 0 | 192.96 | 19.3 | 7.5075 | 214.4 | 321.6 | 0 | 0 | 0.360 | 1 | 23 | 1 | 1 | 1 | 13 | 0.2 | 1 | 28 | 127.9 |
| 1355 | 1120 | 145 | 1120 | 0 | 0 | 228 | 21 | 16.8 | 0 | 105 | 0 | 0 | 0.180 | 1 | 23 | 1 | 1 | 1 | 12 | 0.3 | 1 | 7 | 133.2 |
| 1356 | 429 | 107.2 | 1072 | 0 | 0 | 192.96 | 19.3 | 7.5075 | 214.4 | 321.6 | 0 | 0 | 0.360 | 1 | 23 | 1 | 1 | 1 | 13 | 0.2 | 1 | 56 | 92 |
| 1357 | 429 | 107.2 | 1072 | 0 | 0 | 192.96 | 19.3 | 0 | 214.4 | 321.6 | 0 | 0 | 0.360 | 1 | 23 | 1 | 1 | 0 | 0 | 0 | 1 | 56 | 97.2 |
| 1358 | 429 | 107.2 | 1072 | 0 | 0 | 192.96 | 19.3 | 7.5075 | 214.4 | 321.6 | 0 | 0 | 0.360 | 1 | 23 | 1 | 1 | 1 | 13 | 0.2 | 1 | 56 | 133.8 |
| 1359 | 809 | 270 | 1079 | 0 | 0 | 177 | 21.6 | 0 | 0 | 0 | 0 | 0 | 0.164 | 1 | 23 | 1 | 1 | 0 | 0 | 0 | 1 | 3 | 81.6 |
| 1360 | 800 | 267 | 1067 | 0 | 0 | 175 | 21.3 | 8 | 0 | 0 | 0 | 0 | 0.164 | 1 | 23 | 1 | 1 | 1 | 13 | 0.2 | 1 | 3 | 101.2 |
| 1361 | 792 | 264 | 1056 | 0 | 0 | 173 | 21.1 | 15.84 | 0 | 0 | 0 | 0 | 0.164 | 1 | 23 | 1 | 1 | 1 | 13 | 0.2 | 1 | 3 | 119.1 |
| 1362 | 784 | 261 | 1045 | 0 | 0 | 171 | 20.9 | 23.52 | 0 | 0 | 0 | 0 | 0.164 | 1 | 23 | 1 | 1 | 1 | 13 | 0.2 | 1 | 3 | 122.4 |
| 1363 | 809 | 270 | 1079 | 0 | 0 | 177 | 21.6 | 0 | 0 | 0 | 0 | 0 | 0.164 | 1 | 23 | 1 | 1 | 0 | 0 | 0 | 1 | 7 | 106.9 |
| 1364 | 800 | 267 | 1067 | 0 | 0 | 175 | 21.3 | 8 | 0 | 0 | 0 | 0 | 0.164 | 1 | 23 | 1 | 1 | 1 | 13 | 0.2 | 1 | 7 | 128.1 |
| 1365 | 792 | 264 | 1056 | 0 | 0 | 173 | 21.1 | 15.84 | 0 | 0 | 0 | 0 | 0.164 | 1 | 23 | 1 | 1 | 1 | 13 | 0.2 | 1 | 7 | 142.8 |
| 1366 | 797.78 | 112.36 | 1123.63 | 0 | 0 | 224.73 | 28.09 | 3.9889 | 44.95 | 112.36 | 56.18 | 0 | 0.247 | 1 | 23 | 1 | 1 | 4 | 18 | 0.013 | 1 | 28 | 121.2 |
| 1367 | 1120 | 145 | 1120 | 0 | 0 | 228 | 21 | 22.4 | 0 | 105 | 0 | 0 | 0.180 | 1 | 23 | 1 | 1 | 1 | 12 | 0.3 | 1 | 7 | 138.1 |
| 1368 | 784 | 261 | 1045 | 0 | 0 | 171 | 20.9 | 23.52 | 0 | 0 | 0 | 0 | 0.164 | 1 | 23 | 1 | 1 | 1 | 13 | 0.2 | 1 | 7 | 147.8 |
| 1369 | 809 | 270 | 1079 | 0 | 0 | 177 | 21.6 | 0 | 0 | 0 | 0 | 0 | 0.164 | 1 | 23 | 1 | 1 | 0 | 0 | 0 | 1 | 28 | 123.2 |
| 1370 | 800 | 267 | 1067 | 0 | 0 | 175 | 21.3 | 8 | 0 | 0 | 0 | 0 | 0.164 | 1 | 23 | 1 | 1 | 1 | 13 | 0.2 | 1 | 28 | 150.2 |
| 1371 | 792 | 264 | 1056 | 0 | 0 | 173 | 21.1 | 15.84 | 0 | 0 | 0 | 0 | 0.164 | 1 | 23 | 1 | 1 | 1 | 13 | 0.2 | 1 | 28 | 173.1 |
| 1372 | 784 | 261 | 1045 | 0 | 0 | 171 | 20.9 | 23.52 | 0 | 0 | 0 | 0 | 0.164 | 1 | 23 | 1 | 1 | 1 | 13 | 0.2 | 1 | 28 | 182.9 |
| 1373 | 809 | 270 | 1079 | 0 | 0 | 177 | 21.6 | 0 | 0 | 0 | 0 | 0 | 0.164 | 1 | 23 | 1 | 1 | 0 | 0 | 0 | 1 | 90 | 126.5 |
| 1374 | 800 | 267 | 1067 | 0 | 0 | 175 | 21.3 | 8 | 0 | 0 | 0 | 0 | 0.164 | 1 | 23 | 1 | 1 | 1 | 13 | 0.2 | 1 | 90 | 172.3 |
| 1375 | 792 | 264 | 1056 | 0 | 0 | 173 | 21.1 | 15.84 | 0 | 0 | 0 | 0 | 0.164 | 1 | 23 | 1 | 1 | 1 | 13 | 0.2 | 1 | 90 | 187 |
| 1376 | 784 | 261 | 1045 | 0 | 0 | 171 | 20.9 | 23.52 | 0 | 0 | 0 | 0 | 0.164 | 1 | 23 | 1 | 1 | 1 | 13 | 0.2 | 1 | 90 | 192.7 |
| 1377 | 788 | 197 | 866.8 | 0 | 315 | 173 | 14.77 | 19.7 | 0 | 0 | 0 | 0 | 0.176 | 1 | 23 | 1 | 1 | 1 | 13 | 0.16 | 1 | 7 | 155.8 |
| 1378 | 1120 | 145 | 1120 | 0 | 0 | 228 | 21 | 0 | 0 | 105 | 0 | 0 | 0.180 | 1 | 23 | 1 | 1 | 0 | 0 | 0 | 1 | 28 | 100.5 |
| 1379 | 788 | 197 | 866.8 | 0 | 315 | 173 | 14.77 | 15.76 | 0 | 0 | 0 | 0 | 0.176 | 1 | 23 | 1 | 1 | 1 | 13 | 0.16 | 1 | 7 | 150.2 |
| 1380 | 788 | 197 | 866.8 | 0 | 315 | 173 | 14.77 | 19.7 | 0 | 0 | 0 | 0 | 0.176 | 1 | 23 | 1 | 1 | 1 | 6 | 0.16 | 1 | 7 | 152.6 |
| 1381 | 788 | 197 | 866.8 | 0 | 315 | 173 | 14.77 | 15.76 | 0 | 0 | 0 | 0 | 0.176 | 1 | 23 | 1 | 1 | 1 | 6 | 0.16 | 1 | 7 | 132.4 |
| 1382 | 788 | 197 | 866.8 | 0 | 315 | 173 | 14.77 | 0 | 0 | 0 | 0 | 0 | 0.176 | 1 | 23 | 1 | 1 | 0 | 0 | 0 | 1 | 7 | 106.1 |
| 1383 | 788 | 197 | 866.8 | 0 | 315 | 173 | 14.77 | 19.7 | 0 | 0 | 0 | 0 | 0.176 | 1 | 23 | 1 | 1 | 1 | 13 | 0.16 | 1 | 14 | 171.9 |
| 1384 | 788 | 197 | 866.8 | 0 | 315 | 173 | 14.77 | 15.76 | 0 | 0 | 0 | 0 | 0.176 | 1 | 23 | 1 | 1 | 1 | 13 | 0.16 | 1 | 14 | 165.6 |
| 1385 | 788 | 197 | 866.8 | 0 | 315 | 173 | 14.77 | 19.7 | 0 | 0 | 0 | 0 | 0.176 | 1 | 23 | 1 | 1 | 1 | 6 | 0.16 | 1 | 14 | 170.6 |
| 1386 | 788 | 197 | 866.8 | 0 | 315 | 173 | 14.77 | 15.76 | 0 | 0 | 0 | 0 | 0.176 | 1 | 23 | 1 | 1 | 1 | 6 | 0.16 | 1 | 14 | 152.8 |
| 1387 | 788 | 197 | 866.8 | 0 | 315 | 173 | 14.77 | 0 | 0 | 0 | 0 | 0 | 0.176 | 1 | 23 | 1 | 1 | 0 | 0 | 0 | 1 | 14 | 125.5 |
| 1388 | 788 | 197 | 866.8 | 0 | 315 | 173 | 14.77 | 19.7 | 0 | 0 | 0 | 0 | 0.176 | 1 | 23 | 1 | 1 | 1 | 13 | 0.16 | 1 | 21 | 180 |
| 1389 | 1120 | 145 | 1120 | 0 | 0 | 228 | 21 | 2.8 | 0 | 105 | 0 | 0 | 0.180 | 1 | 23 | 1 | 1 | 4 | 12 | 0.45 | 1 | 28 | 114.6 |
| 1390 | 788 | 197 | 866.8 | 0 | 315 | 173 | 14.77 | 15.76 | 0 | 0 | 0 | 0 | 0.176 | 1 | 23 | 1 | 1 | 1 | 13 | 0.16 | 1 | 21 | 170.7 |
| 1391 | 788 | 197 | 866.8 | 0 | 315 | 173 | 14.77 | 19.7 | 0 | 0 | 0 | 0 | 0.176 | 1 | 23 | 1 | 1 | 1 | 6 | 0.16 | 1 | 21 | 175.5 |
| 1392 | 788 | 197 | 866.8 | 0 | 315 | 173 | 14.77 | 15.76 | 0 | 0 | 0 | 0 | 0.176 | 1 | 23 | 1 | 1 | 1 | 6 | 0.16 | 1 | 21 | 159.2 |
| 1393 | 788 | 197 | 866.8 | 0 | 315 | 173 | 14.77 | 0 | 0 | 0 | 0 | 0 | 0.176 | 1 | 23 | 1 | 1 | 0 | 0 | 0 | 1 | 21 | 134.7 |
| 1394 | 788 | 197 | 866.8 | 0 | 315 | 173 | 14.77 | 19.7 | 0 | 0 | 0 | 0 | 0.176 | 1 | 23 | 1 | 1 | 1 | 13 | 0.16 | 1 | 28 | 184 |
| 1395 | 788 | 197 | 866.8 | 0 | 315 | 173 | 14.77 | 15.76 | 0 | 0 | 0 | 0 | 0.176 | 1 | 23 | 1 | 1 | 1 | 13 | 0.16 | 1 | 28 | 173.8 |
| 1396 | 788 | 197 | 866.8 | 0 | 315 | 173 | 14.77 | 19.7 | 0 | 0 | 0 | 0 | 0.176 | 1 | 23 | 1 | 1 | 1 | 6 | 0.16 | 1 | 28 | 182.3 |
| 1397 | 788 | 197 | 866.8 | 0 | 315 | 173 | 14.77 | 15.76 | 0 | 0 | 0 | 0 | 0.176 | 1 | 23 | 1 | 1 | 1 | 6 | 0.16 | 1 | 28 | 167.3 |
| 1398 | 788 | 197 | 866.8 | 0 | 315 | 173 | 14.77 | 0 | 0 | 0 | 0 | 0 | 0.176 | 1 | 23 | 1 | 1 | 0 | 0 | 0 | 1 | 28 | 134.7 |
| 1399 | 472 | 262 | 1049 | 0 | 0 | 178 | 21 | 0 | 0 | 315 | 0 | 0 | 0.243 | 1 | 23 | 1 | 1 | 0 | 0 | 0 | 1 | 7 | 108.9 |
| 1400 | 1120 | 145 | 1120 | 0 | 0 | 228 | 21 | 5.6 | 0 | 105 | 0 | 0 | 0.180 | 1 | 23 | 1 | 1 | 4 | 12 | 0.45 | 1 | 28 | 122.7 |
| 1401 | 472 | 262 | 1049 | 0 | 0 | 178 | 21 | 9.44 | 0 | 315 | 0 | 0 | 0.243 | 1 | 23 | 1 | 1 | 1 | 13 | 0.2 | 1 | 7 | 132.9 |
| 1402 | 472 | 262 | 1049 | 0 | 0 | 178 | 21 | 9.44 | 0 | 315 | 0 | 0 | 0.243 | 1 | 23 | 1 | 1 | 1 | 6 | 0.2 | 1 | 7 | 116 |
| 1403 | 472 | 262 | 1049 | 0 | 0 | 178 | 21 | 0 | 0 | 315 | 0 | 0 | 0.243 | 1 | 23 | 1 | 1 | 0 | 0 | 0 | 1 | 28 | 114.6 |
| 1404 | 472 | 262 | 1049 | 0 | 0 | 178 | 21 | 9.44 | 0 | 315 | 0 | 0 | 0.243 | 1 | 23 | 1 | 1 | 1 | 13 | 0.2 | 1 | 28 | 154.9 |
| 1405 | 472 | 262 | 1049 | 0 | 0 | 178 | 21 | 9.44 | 0 | 315 | 0 | 0 | 0.243 | 1 | 23 | 1 | 1 | 1 | 6 | 0.2 | 1 | 28 | 139.3 |
| 1406 | 960 | 240 | 791.3 | 0 | 0 | 234 | 45 | 2.4 | 0 | 0 | 0 | 0 | 0.195 | 1 | 23 | 1 | 1 | 5 | 13 | 0.018 | 1 | 7 | 134 |
| 1407 | 960 | 240 | 788.9 | 0 | 0 | 234 | 45 | 4.8 | 0 | 0 | 0 | 0 | 0.195 | 1 | 23 | 1 | 1 | 5 | 13 | 0.018 | 1 | 7 | 137.4 |
| 1408 | 960 | 240 | 793.9 | 0 | 0 | 234 | 42 | 7.2 | 0 | 0 | 0 | 0 | 0.195 | 1 | 23 | 1 | 1 | 5 | 13 | 0.018 | 1 | 7 | 140.6 |
| 1409 | 960 | 240 | 798.8 | 0 | 0 | 234 | 39 | 9.6 | 0 | 0 | 0 | 0 | 0.195 | 1 | 23 | 1 | 1 | 5 | 13 | 0.018 | 1 | 7 | 143.7 |
| 1410 | 960 | 240 | 698.3 | 0 | 0 | 234 | 78 | 14.4 | 0 | 0 | 0 | 0 | 0.195 | 1 | 23 | 1 | 1 | 5 | 13 | 0.018 | 1 | 7 | 149 |
| 1411 | 1120 | 145 | 1120 | 0 | 0 | 228 | 21 | 8.4 | 0 | 105 | 0 | 0 | 0.180 | 1 | 23 | 1 | 1 | 4 | 12 | 0.45 | 1 | 28 | 119.3 |
| 1412 | 960 | 240 | 649.3 | 0 | 0 | 234 | 96 | 19.2 | 0 | 0 | 0 | 0 | 0.195 | 1 | 23 | 1 | 1 | 5 | 13 | 0.018 | 1 | 7 | 146 |
| 1413 | 960 | 240 | 791.3 | 0 | 0 | 234 | 45 | 2.4 | 0 | 0 | 0 | 0 | 0.195 | 1 | 23 | 1 | 1 | 5 | 13 | 0.018 | 1 | 28 | 161.1 |
| 1414 | 960 | 240 | 788.9 | 0 | 0 | 234 | 45 | 4.8 | 0 | 0 | 0 | 0 | 0.195 | 1 | 23 | 1 | 1 | 5 | 13 | 0.018 | 1 | 28 | 165.8 |
| 1415 | 960 | 240 | 793.9 | 0 | 0 | 234 | 42 | 7.2 | 0 | 0 | 0 | 0 | 0.195 | 1 | 23 | 1 | 1 | 5 | 13 | 0.018 | 1 | 28 | 171.1 |
| 1416 | 960 | 240 | 798.8 | 0 | 0 | 234 | 39 | 9.6 | 0 | 0 | 0 | 0 | 0.195 | 1 | 23 | 1 | 1 | 5 | 13 | 0.018 | 1 | 28 | 172 |
| 1417 | 960 | 240 | 698.3 | 0 | 0 | 234 | 78 | 14.4 | 0 | 0 | 0 | 0 | 0.195 | 1 | 23 | 1 | 1 | 5 | 13 | 0.018 | 1 | 28 | 177.3 |
| 1418 | 960 | 240 | 649.3 | 0 | 0 | 234 | 96 | 19.2 | 0 | 0 | 0 | 0 | 0.195 | 1 | 23 | 1 | 1 | 5 | 13 | 0.018 | 1 | 28 | 174.3 |
| 1419 | 960 | 240 | 791.3 | 0 | 0 | 234 | 45 | 2.4 | 0 | 0 | 0 | 0 | 0.195 | 1 | 23 | 1 | 1 | 5 | 13 | 0.018 | 1 | 56 | 178.4 |
| 1420 | 960 | 240 | 788.9 | 0 | 0 | 234 | 45 | 4.8 | 0 | 0 | 0 | 0 | 0.195 | 1 | 23 | 1 | 1 | 5 | 13 | 0.018 | 1 | 56 | 180.6 |
| 1421 | 960 | 240 | 793.9 | 0 | 0 | 234 | 42 | 7.2 | 0 | 0 | 0 | 0 | 0.195 | 1 | 23 | 1 | 1 | 5 | 13 | 0.018 | 1 | 56 | 183.4 |
| 1422 | 1120 | 145 | 1120 | 0 | 0 | 228 | 21 | 11.2 | 0 | 105 | 0 | 0 | 0.180 | 1 | 23 | 1 | 1 | 4 | 12 | 0.45 | 1 | 28 | 130.9 |
| 1423 | 960 | 240 | 798.8 | 0 | 0 | 234 | 39 | 9.6 | 0 | 0 | 0 | 0 | 0.195 | 1 | 23 | 1 | 1 | 5 | 13 | 0.018 | 1 | 56 | 183.7 |
| 1424 | 960 | 240 | 698.3 | 0 | 0 | 234 | 78 | 14.4 | 0 | 0 | 0 | 0 | 0.195 | 1 | 23 | 1 | 1 | 5 | 13 | 0.018 | 1 | 56 | 188.1 |
| 1425 | 960 | 240 | 649.3 | 0 | 0 | 234 | 96 | 19.2 | 0 | 0 | 0 | 0 | 0.195 | 1 | 23 | 1 | 1 | 5 | 13 | 0.018 | 1 | 56 | 186.6 |
| 1426 | 960 | 240 | 791.3 | 0 | 0 | 234 | 45 | 2.4 | 0 | 0 | 0 | 0 | 0.195 | 1 | 23 | 1 | 1 | 5 | 13 | 0.018 | 1 | 90 | 185.8 |
| 1427 | 960 | 240 | 788.9 | 0 | 0 | 234 | 45 | 4.8 | 0 | 0 | 0 | 0 | 0.195 | 1 | 23 | 1 | 1 | 5 | 13 | 0.018 | 1 | 90 | 188.3 |
| 1428 | 960 | 240 | 793.9 | 0 | 0 | 234 | 42 | 7.2 | 0 | 0 | 0 | 0 | 0.195 | 1 | 23 | 1 | 1 | 5 | 13 | 0.018 | 1 | 90 | 188.9 |
| 1429 | 960 | 240 | 798.8 | 0 | 0 | 234 | 39 | 9.6 | 0 | 0 | 0 | 0 | 0.195 | 1 | 23 | 1 | 1 | 5 | 13 | 0.018 | 1 | 90 | 191.1 |
| 1430 | 960 | 240 | 698.3 | 0 | 0 | 234 | 78 | 14.4 | 0 | 0 | 0 | 0 | 0.195 | 1 | 23 | 1 | 1 | 5 | 13 | 0.018 | 1 | 90 | 194.3 |
| 1431 | 960 | 240 | 649.3 | 0 | 0 | 234 | 96 | 19.2 | 0 | 0 | 0 | 0 | 0.195 | 1 | 23 | 1 | 1 | 5 | 13 | 0.018 | 1 | 90 | 193.4 |
| 1432 | 809 | 270 | 1079 | 0 | 0 | 177 | 21.6 | 0 | 0 | 0 | 0 | 0 | 0.164 | 1 | 23 | 1 | 1 | 0 | 13 | 0.2 | 1 | 3 | 69.3 |
| 1433 | 1120 | 145 | 1120 | 0 | 0 | 228 | 21 | 16.8 | 0 | 105 | 0 | 0 | 0.180 | 1 | 23 | 1 | 1 | 4 | 12 | 0.45 | 1 | 28 | 125.7 |
| 1434 | 800 | 267 | 1067 | 0 | 0 | 175 | 21.3 | 8 | 0 | 0 | 0 | 0 | 0.164 | 1 | 23 | 1 | 1 | 1 | 13 | 0.2 | 1 | 3 | 86.6 |
| 1435 | 792 | 264 | 1056 | 0 | 0 | 173 | 21.1 | 15.84 | 0 | 0 | 0 | 0 | 0.164 | 1 | 23 | 1 | 1 | 1 | 13 | 0.2 | 1 | 3 | 100 |
| 1436 | 784 | 261 | 1045 | 0 | 0 | 171 | 20.9 | 23.52 | 0 | 0 | 0 | 0 | 0.164 | 1 | 23 | 1 | 1 | 1 | 13 | 0.2 | 1 | 3 | 102.5 |
| 1437 | 809 | 270 | 1079 | 0 | 0 | 177 | 21.6 | 0 | 0 | 0 | 0 | 0 | 0.164 | 1 | 23 | 1 | 1 | 0 | 13 | 0.2 | 1 | 7 | 91.8 |
| 1438 | 800 | 267 | 1067 | 0 | 0 | 175 | 21.3 | 8 | 0 | 0 | 0 | 0 | 0.164 | 1 | 23 | 1 | 1 | 1 | 13 | 0.2 | 1 | 7 | 108.2 |
| 1439 | 792 | 264 | 1056 | 0 | 0 | 173 | 21.1 | 15.84 | 0 | 0 | 0 | 0 | 0.164 | 1 | 23 | 1 | 1 | 1 | 13 | 0.2 | 1 | 7 | 119.9 |
| 1440 | 784 | 261 | 1045 | 0 | 0 | 171 | 20.9 | 23.52 | 0 | 0 | 0 | 0 | 0.164 | 1 | 23 | 1 | 1 | 1 | 13 | 0.2 | 1 | 7 | 123.2 |
| 1441 | 809 | 270 | 1079 | 0 | 0 | 177 | 21.6 | 0 | 0 | 0 | 0 | 0 | 0.164 | 1 | 23 | 1 | 1 | 0 | 13 | 0.2 | 1 | 28 | 104.6 |
| 1442 | 800 | 267 | 1067 | 0 | 0 | 175 | 21.3 | 8 | 0 | 0 | 0 | 0 | 0.164 | 1 | 23 | 1 | 1 | 1 | 13 | 0.2 | 1 | 28 | 126.6 |
| 1443 | 792 | 264 | 1056 | 0 | 0 | 173 | 21.1 | 15.84 | 0 | 0 | 0 | 0 | 0.164 | 1 | 23 | 1 | 1 | 1 | 13 | 0.2 | 1 | 28 | 145.7 |
| 1444 | 1120 | 145 | 1120 | 0 | 0 | 228 | 21 | 2.8 | 0 | 105 | 0 | 0 | 0.180 | 1 | 23 | 1 | 1 | 1 | 12 | 0.3 | 1 | 28 | 120.6 |
| 1445 | 784 | 261 | 1045 | 0 | 0 | 171 | 20.9 | 23.52 | 0 | 0 | 0 | 0 | 0.164 | 1 | 23 | 1 | 1 | 1 | 13 | 0.2 | 1 | 28 | 152 |
| 1446 | 809 | 270 | 1079 | 0 | 0 | 177 | 21.6 | 0 | 0 | 0 | 0 | 0 | 0.164 | 1 | 23 | 1 | 1 | 0 | 13 | 0.2 | 1 | 90 | 109.1 |
| 1447 | 800 | 267 | 1067 | 0 | 0 | 175 | 21.3 | 8 | 0 | 0 | 0 | 0 | 0.164 | 1 | 23 | 1 | 1 | 1 | 13 | 0.2 | 1 | 90 | 144.8 |
| 1448 | 792 | 264 | 1056 | 0 | 0 | 173 | 21.1 | 15.84 | 0 | 0 | 0 | 0 | 0.164 | 1 | 23 | 1 | 1 | 1 | 13 | 0.2 | 1 | 90 | 156.3 |
| 1449 | 784 | 261 | 1045 | 0 | 0 | 171 | 20.9 | 23.52 | 0 | 0 | 0 | 0 | 0.164 | 1 | 23 | 1 | 1 | 1 | 13 | 0.2 | 1 | 90 | 162.6 |
| 1450 | 472 | 262 | 1049 | 0 | 0 | 178 | 21 | 0 | 0 | 315 | 0 | 0 | 0.243 | 1 | 23 | 1 | 1 | 0 | 0 | 0 | 1 | 7 | 89.5 |
| 1451 | 472 | 262 | 1049 | 0 | 0 | 178 | 21 | 9.44 | 0 | 315 | 0 | 0 | 0.243 | 1 | 23 | 1 | 1 | 1 | 6 | 0.2 | 1 | 7 | 113.5 |
| 1452 | 472 | 262 | 1049 | 0 | 0 | 178 | 21 | 9.44 | 0 | 315 | 0 | 0 | 0.243 | 1 | 23 | 1 | 1 | 1 | 8 | 0.2 | 1 | 7 | 116.4 |
| 1453 | 472 | 262 | 1049 | 0 | 0 | 178 | 21 | 9.44 | 0 | 315 | 0 | 0 | 0.243 | 1 | 23 | 1 | 1 | 1 | 10 | 0.2 | 1 | 7 | 103.9 |
| 1454 | 472 | 262 | 1049 | 0 | 0 | 178 | 21 | 9.44 | 0 | 315 | 0 | 0 | 0.243 | 1 | 23 | 1 | 1 | 1 | 11 | 0.2 | 1 | 7 | 99.1 |
| 1455 | 1120 | 145 | 1120 | 0 | 0 | 228 | 21 | 5.6 | 0 | 105 | 0 | 0 | 0.180 | 1 | 23 | 1 | 1 | 1 | 12 | 0.3 | 1 | 28 | 127.9 |
| 1456 | 472 | 262 | 1049 | 0 | 0 | 178 | 21 | 9.44 | 0 | 315 | 0 | 0 | 0.243 | 1 | 23 | 1 | 1 | 1 | 13 | 0.2 | 1 | 7 | 96.2 |
| 1457 | 472 | 262 | 1049 | 0 | 0 | 178 | 21 | 0 | 0 | 315 | 0 | 0 | 0.243 | 1 | 23 | 1 | 1 | 0 | 0 | 0 | 1 | 28 | 92.4 |
| 1458 | 472 | 262 | 1049 | 0 | 0 | 178 | 21 | 9.44 | 0 | 315 | 0 | 0 | 0.243 | 1 | 23 | 1 | 1 | 1 | 6 | 0.2 | 1 | 28 | 130.8 |
| 1459 | 472 | 262 | 1049 | 0 | 0 | 178 | 21 | 9.44 | 0 | 315 | 0 | 0 | 0.243 | 1 | 23 | 1 | 1 | 1 | 8 | 0.2 | 1 | 28 | 138.5 |
| 1460 | 472 | 262 | 1049 | 0 | 0 | 178 | 21 | 9.44 | 0 | 315 | 0 | 0 | 0.243 | 1 | 23 | 1 | 1 | 1 | 10 | 0.2 | 1 | 28 | 125.1 |
| 1461 | 472 | 262 | 1049 | 0 | 0 | 178 | 21 | 9.44 | 0 | 315 | 0 | 0 | 0.243 | 1 | 23 | 1 | 1 | 1 | 11 | 0.2 | 1 | 28 | 121.2 |
| 1462 | 472 | 262 | 1049 | 0 | 0 | 178 | 21 | 9.44 | 0 | 315 | 0 | 0 | 0.243 | 1 | 23 | 1 | 1 | 1 | 13 | 0.2 | 1 | 28 | 117.4 |
| 1463 | 950 | 255 | 873 | 0 | 0 | 189 | 31 | 23.75 | 0 | 0 | 0 | 0 | 0.157 | 1 | 23 | 1 | 1 | 1 | 13 | 0.2 | 1 | 7 | 101 |
| 1464 | 950 | 255 | 873 | 0 | 0 | 189 | 31 | 23.75 | 0 | 0 | 0 | 0 | 0.157 | 1 | 23 | 1 | 1 | 1 | 13 | 0.2 | 1 | 28 | 130.8 |
| 1465 | 950 | 255 | 873 | 0 | 0 | 189 | 31 | 23.75 | 0 | 0 | 0 | 0 | 0.157 | 1 | 23 | 1 | 1 | 1 | 13 | 0.2 | 1 | 90 | 135.6 |
| 1466 | 1120 | 145 | 1120 | 0 | 0 | 228 | 21 | 8.4 | 0 | 105 | 0 | 0 | 0.180 | 1 | 23 | 1 | 1 | 1 | 12 | 0.3 | 1 | 28 | 133.4 |
| 1467 | 1277.4 | 95.8 | 664.6 | 0 | 0 | 198 | 42.15 | 38.322 | 0 | 0 | 0 | 0 | 0.144 | 1 | 23 | 1 | 1 | 1 | 13 | 0.16 | 1 | 56 | 208.8 |
| 1468 | 591.9 | 24.7 | 1277.2 | 0 | 0 | 159.3 | 44 | 0 | 264.3 | 0 | 0 | 0 | 0.258 | 1 | 23 | 1 | 1 | 0 | 0 | 0 | 1 | 28 | 105.6 |
| 1469 | 591.9 | 24.7 | 1277.2 | 0 | 0 | 159.3 | 44 | 0 | 264.3 | 0 | 0 | 0 | 0.258 | 1 | 23 | 1 | 1 | 0 | 0 | 0 | 1 | 91 | 105.6 |
| 1470 | 606.4 | 25.3 | 1308.5 | 0 | 0 | 163.2 | 45.1 | 0 | 0 | 270.7 | 0 | 0 | 0.258 | 1 | 23 | 1 | 1 | 0 | 0 | 0 | 1 | 28 | 113.9 |
| 1471 | 606.4 | 25.3 | 1308.5 | 0 | 0 | 163.2 | 45.1 | 0 | 0 | 270.7 | 0 | 0 | 0.258 | 1 | 23 | 1 | 1 | 0 | 0 | 0 | 1 | 91 | 113.9 |
| 1472 | 883.9 | 25.5 | 1318.5 | 0 | 0 | 164.4 | 45.5 | 0 | 0 | 0 | 0 | 0 | 0.181 | 1 | 23 | 1 | 1 | 0 | 0 | 0 | 1 | 28 | 117.3 |
| 1473 | 883.9 | 25.5 | 1318.5 | 0 | 0 | 164.4 | 45.5 | 0 | 0 | 0 | 0 | 0 | 0.181 | 1 | 23 | 1 | 1 | 0 | 0 | 0 | 1 | 91 | 117.3 |
| 1474 | 657 | 119.4 | 1050 | 0 | 0 | 180.93 | 12.66 | 13.14 | 0 | 429.8 | 0 | 0 | 0.233 | 1 | 23 | 1 | 1 | 1 | 13 | 0.2 | 1 | 7 | 96.2 |
| 1475 | 657 | 119.4 | 1050 | 0 | 0 | 180.93 | 12.66 | 13.14 | 0 | 429.8 | 0 | 0 | 0.233 | 1 | 23 | 1 | 1 | 1 | 13 | 0.2 | 1 | 28 | 118.3 |
| 1476 | 657 | 119.4 | 1050 | 0 | 0 | 180.93 | 12.66 | 13.14 | 0 | 429.8 | 0 | 0 | 0.233 | 1 | 23 | 1 | 1 | 1 | 13 | 0.2 | 1 | 56 | 129.9 |
| 1477 | 797.78 | 112.36 | 1123.63 | 0 | 0 | 224.73 | 28.09 | 7.9778 | 44.95 | 112.36 | 56.18 | 0 | 0.247 | 1 | 23 | 1 | 1 | 4 | 18 | 0.013 | 1 | 1 | 65.3 |
| 1478 | 1120 | 145 | 1120 | 0 | 0 | 228 | 21 | 11.2 | 0 | 105 | 0 | 0 | 0.180 | 1 | 23 | 1 | 1 | 1 | 12 | 0.3 | 1 | 28 | 140.7 |
| 1479 | 657 | 119.4 | 1050 | 0 | 0 | 180.93 | 12.66 | 13.14 | 0 | 429.8 | 0 | 0 | 0.233 | 1 | 23 | 1 | 1 | 1 | 13 | 0.2 | 1 | 91 | 133.7 |
| 1480 | 960 | 240 | 960 | 0 | 0 | 174 | 24 | 76.8 | 0 | 0 | 0 | 0 | 0.145 | 1 | 23 | 1 | 1 | 1 | 13 | 0.18 | 1 | 3 | 70 |
| 1481 | 960 | 240 | 960 | 0 | 0 | 174 | 24 | 76.8 | 0 | 0 | 0 | 0 | 0.145 | 1 | 23 | 1 | 1 | 1 | 13 | 0.18 | 1 | 7 | 78.9 |
| 1482 | 960 | 240 | 960 | 0 | 0 | 174 | 24 | 76.8 | 0 | 0 | 0 | 0 | 0.145 | 1 | 23 | 1 | 1 | 1 | 13 | 0.18 | 1 | 28 | 106.1 |
| 1483 | 960 | 240 | 960 | 0 | 0 | 207 | 24 | 76.8 | 0 | 0 | 0 | 0 | 0.173 | 1 | 23 | 1 | 1 | 1 | 13 | 0.18 | 1 | 3 | 82.5 |
| 1484 | 960 | 240 | 960 | 0 | 0 | 207 | 24 | 76.8 | 0 | 0 | 0 | 0 | 0.173 | 1 | 23 | 1 | 1 | 1 | 13 | 0.18 | 1 | 7 | 115.1 |
| 1485 | 960 | 240 | 960 | 0 | 0 | 207 | 24 | 76.8 | 0 | 0 | 0 | 0 | 0.173 | 1 | 23 | 1 | 1 | 1 | 13 | 0.18 | 1 | 28 | 150.2 |
| 1486 | 960 | 240 | 960 | 0 | 0 | 228 | 24 | 76.8 | 0 | 0 | 0 | 0 | 0.190 | 1 | 23 | 1 | 1 | 1 | 13 | 0.18 | 1 | 1 | 40.4 |
| 1487 | 960 | 240 | 960 | 0 | 0 | 228 | 24 | 76.8 | 0 | 0 | 0 | 0 | 0.190 | 1 | 23 | 1 | 1 | 1 | 13 | 0.18 | 1 | 3 | 75.7 |
| 1488 | 960 | 240 | 960 | 0 | 0 | 228 | 24 | 76.8 | 0 | 0 | 0 | 0 | 0.190 | 1 | 23 | 1 | 1 | 1 | 13 | 0.18 | 1 | 7 | 100.8 |
| 1489 | 1120 | 145 | 1120 | 0 | 0 | 228 | 21 | 16.8 | 0 | 105 | 0 | 0 | 0.180 | 1 | 23 | 1 | 1 | 1 | 12 | 0.3 | 1 | 28 | 146.2 |
| 1490 | 960 | 240 | 960 | 0 | 0 | 228 | 24 | 76.8 | 0 | 0 | 0 | 0 | 0.190 | 1 | 23 | 1 | 1 | 1 | 13 | 0.18 | 1 | 28 | 136.1 |
| 1491 | 960 | 240 | 960 | 0 | 0 | 260 | 24 | 76.8 | 0 | 0 | 0 | 0 | 0.217 | 1 | 23 | 1 | 1 | 1 | 13 | 0.18 | 1 | 3 | 76 |
| 1492 | 960 | 240 | 960 | 0 | 0 | 260 | 24 | 76.8 | 0 | 0 | 0 | 0 | 0.217 | 1 | 23 | 1 | 1 | 1 | 13 | 0.18 | 1 | 7 | 105.1 |
| 1493 | 960 | 240 | 960 | 0 | 0 | 260 | 24 | 76.8 | 0 | 0 | 0 | 0 | 0.217 | 1 | 23 | 1 | 1 | 1 | 13 | 0.18 | 1 | 28 | 136.4 |
| 1494 | 960 | 240 | 960 | 0 | 0 | 286 | 24 | 76.8 | 0 | 0 | 0 | 0 | 0.238 | 1 | 23 | 1 | 1 | 1 | 13 | 0.18 | 1 | 3 | 66.6 |
| 1495 | 960 | 240 | 960 | 0 | 0 | 286 | 24 | 76.8 | 0 | 0 | 0 | 0 | 0.238 | 1 | 23 | 1 | 1 | 1 | 13 | 0.18 | 1 | 7 | 98.1 |
| 1496 | 960 | 240 | 960 | 0 | 0 | 286 | 24 | 76.8 | 0 | 0 | 0 | 0 | 0.238 | 1 | 23 | 1 | 1 | 1 | 13 | 0.18 | 1 | 28 | 132.6 |
| 1497 | 960 | 0 | 960 | 0 | 0 | 164 | 28 | 76.8 | 250 | 0 | 0 | 0 | 0.171 | 1 | 23 | 1 | 1 | 1 | 13 | 0.18 | 1 | 1 | 66.2 |
| 1498 | 960 | 0 | 960 | 0 | 0 | 174 | 28 | 76.8 | 250 | 0 | 0 | 0 | 0.181 | 1 | 23 | 1 | 1 | 1 | 13 | 0.18 | 1 | 1 | 63.7 |
| 1499 | 960 | 0 | 960 | 0 | 0 | 183 | 28 | 76.8 | 250 | 0 | 0 | 0 | 0.191 | 1 | 23 | 1 | 1 | 1 | 13 | 0.18 | 1 | 1 | 45.9 |
| 1500 | 1120 | 145 | 1120 | 0 | 0 | 228 | 21 | 22.4 | 0 | 105 | 0 | 0 | 0.180 | 1 | 23 | 1 | 1 | 1 | 12 | 0.3 | 1 | 28 | 156.9 |
| 1501 | 960 | 0 | 960 | 0 | 0 | 193 | 28 | 76.8 | 250 | 0 | 0 | 0 | 0.201 | 1 | 23 | 1 | 1 | 1 | 13 | 0.18 | 1 | 1 | 58.2 |
| 1502 | 960 | 0 | 960 | 0 | 0 | 202 | 28 | 76.8 | 250 | 0 | 0 | 0 | 0.210 | 1 | 23 | 1 | 1 | 1 | 13 | 0.18 | 1 | 1 | 49.9 |
| 1503 | 960 | 0 | 960 | 0 | 0 | 212 | 28 | 76.8 | 250 | 0 | 0 | 0 | 0.221 | 1 | 23 | 1 | 1 | 1 | 13 | 0.18 | 1 | 1 | 52.1 |
| 1504 | 960 | 250 | 960 | 0 | 0 | 212 | 28 | 76.8 | 0 | 0 | 0 | 0 | 0.175 | 1 | 23 | 1 | 1 | 1 | 13 | 0.18 | 1 | 1 | 72.6 |
| 1505 | 960 | 0 | 960 | 0 | 0 | 164 | 28 | 76.8 | 250 | 0 | 0 | 0 | 0.171 | 1 | 23 | 1 | 1 | 1 | 13 | 0.18 | 1 | 3 | 85 |
| 1506 | 960 | 0 | 960 | 0 | 0 | 174 | 28 | 76.8 | 250 | 0 | 0 | 0 | 0.181 | 1 | 23 | 1 | 1 | 1 | 13 | 0.18 | 1 | 3 | 79.7 |
| 1507 | 960 | 0 | 960 | 0 | 0 | 183 | 28 | 76.8 | 250 | 0 | 0 | 0 | 0.191 | 1 | 23 | 1 | 1 | 1 | 13 | 0.18 | 1 | 3 | 77 |
| 1508 | 960 | 0 | 960 | 0 | 0 | 193 | 28 | 76.8 | 250 | 0 | 0 | 0 | 0.201 | 1 | 23 | 1 | 1 | 1 | 13 | 0.18 | 1 | 3 | 81 |
| 1509 | 960 | 0 | 960 | 0 | 0 | 202 | 28 | 76.8 | 250 | 0 | 0 | 0 | 0.210 | 1 | 23 | 1 | 1 | 1 | 13 | 0.18 | 1 | 3 | 74.7 |
| 1510 | 960 | 0 | 960 | 0 | 0 | 212 | 28 | 76.8 | 250 | 0 | 0 | 0 | 0.221 | 1 | 23 | 1 | 1 | 1 | 13 | 0.18 | 1 | 3 | 75.7 |
| 1511 | 850 | 212.5 | 935 | 0 | 255 | 212.5 | 21.2 | 0 | 0 | 0 | 0 | 0 | 0.200 | 1 | 23 | 1 | 1 | 0 | 0 | 0 | 1 | 28 | 114.3 |
| 1512 | 960 | 250 | 960 | 0 | 0 | 212 | 28 | 76.8 | 0 | 0 | 0 | 0 | 0.175 | 1 | 23 | 1 | 1 | 1 | 13 | 0.18 | 1 | 3 | 85.7 |
| 1513 | 960 | 0 | 960 | 0 | 0 | 164 | 28 | 76.8 | 250 | 0 | 0 | 0 | 0.171 | 1 | 23 | 1 | 1 | 1 | 13 | 0.18 | 1 | 28 | 116.8 |
| 1514 | 960 | 0 | 960 | 0 | 0 | 174 | 28 | 76.8 | 250 | 0 | 0 | 0 | 0.181 | 1 | 23 | 1 | 1 | 1 | 13 | 0.18 | 1 | 28 | 113.8 |
| 1515 | 960 | 0 | 960 | 0 | 0 | 183 | 28 | 76.8 | 250 | 0 | 0 | 0 | 0.191 | 1 | 23 | 1 | 1 | 1 | 13 | 0.18 | 1 | 28 | 109.4 |
| 1516 | 960 | 0 | 960 | 0 | 0 | 193 | 28 | 76.8 | 250 | 0 | 0 | 0 | 0.201 | 1 | 23 | 1 | 1 | 1 | 13 | 0.18 | 1 | 28 | 115.2 |
| 1517 | 960 | 0 | 960 | 0 | 0 | 202 | 28 | 76.8 | 250 | 0 | 0 | 0 | 0.210 | 1 | 23 | 1 | 1 | 1 | 13 | 0.18 | 1 | 28 | 111.8 |
| 1518 | 960 | 0 | 960 | 0 | 0 | 212 | 28 | 76.8 | 250 | 0 | 0 | 0 | 0.221 | 1 | 23 | 1 | 1 | 1 | 13 | 0.18 | 1 | 28 | 110.5 |
| 1519 | 960 | 250 | 960 | 0 | 0 | 212 | 28 | 76.8 | 0 | 0 | 0 | 0 | 0.175 | 1 | 23 | 1 | 1 | 1 | 13 | 0.18 | 1 | 28 | 118.7 |
| 1520 | 657 | 119 | 1013 | 0 | 0 | 185 | 40 | 13.14 | 0 | 418 | 0 | 0 | 0.238 | 1 | 23 | 1 | 1 | 1 | 13 | 0.2 | 1 | 2 | 60.7 |
| 1521 | 657 | 119 | 1013 | 0 | 0 | 185 | 40 | 13.14 | 0 | 418 | 0 | 0 | 0.238 | 1 | 23 | 1 | 1 | 1 | 13 | 0.2 | 1 | 3 | 76.3 |
| 1522 | 850 | 212.5 | 935 | 0 | 255 | 212.5 | 21.2 | 8.5 | 0 | 0 | 0 | 0 | 0.200 | 1 | 23 | 1 | 1 | 4 | 10 | 0.44 | 1 | 28 | 123 |
| 1523 | 657 | 119 | 1013 | 0 | 0 | 185 | 40 | 13.14 | 0 | 418 | 0 | 0 | 0.238 | 1 | 23 | 1 | 1 | 1 | 13 | 0.2 | 1 | 5 | 94.3 |
| 1524 | 657 | 119 | 1013 | 0 | 0 | 185 | 40 | 13.14 | 0 | 418 | 0 | 0 | 0.238 | 1 | 23 | 1 | 1 | 1 | 13 | 0.2 | 1 | 7 | 104.4 |
| 1525 | 657 | 119 | 1013 | 0 | 0 | 185 | 40 | 13.14 | 0 | 418 | 0 | 0 | 0.238 | 1 | 23 | 1 | 1 | 1 | 13 | 0.2 | 1 | 14 | 118.6 |
| 1526 | 657 | 119 | 1013 | 0 | 0 | 185 | 40 | 13.14 | 0 | 418 | 0 | 0 | 0.238 | 1 | 23 | 1 | 1 | 1 | 13 | 0.2 | 1 | 28 | 130.1 |
| 1527 | 657 | 119 | 1013 | 0 | 0 | 185 | 40 | 13.14 | 0 | 418 | 0 | 0 | 0.238 | 1 | 23 | 1 | 1 | 1 | 13 | 0.2 | 1 | 60 | 145.1 |
| 1528 | 657 | 119 | 1013 | 0 | 0 | 185 | 40 | 13.14 | 0 | 418 | 0 | 0 | 0.238 | 1 | 23 | 1 | 1 | 1 | 13 | 0.2 | 1 | 120 | 151.1 |
| 1529 | 657 | 119 | 1013 | 0 | 0 | 185 | 40 | 13.14 | 0 | 418 | 0 | 0 | 0.238 | 1 | 23 | 1 | 1 | 1 | 13 | 0.2 | 1 | 150 | 153.8 |
| 1530 | 657 | 119 | 1013 | 0 | 0 | 185 | 40 | 13.14 | 0 | 418 | 0 | 0 | 0.238 | 1 | 23 | 1 | 1 | 1 | 13 | 0.2 | 1 | 180 | 156 |
| 1531 | 657 | 119 | 1013 | 0 | 0 | 185 | 40 | 13.14 | 0 | 418 | 0 | 0 | 0.238 | 1 | 23 | 1 | 1 | 1 | 13 | 0.2 | 1 | 360 | 163.9 |
| 1532 | 1251.2 | 291.3 | 407.8 | 0 | 0 | 201.4 | 28.2 | 0 | 0 | 0 | 0 | 0 | 0.131 | 1 | 23 | 1 | 1 | 0 | 0 | 0 | 1 | 3 | 107.6 |
| 1533 | 850 | 212.5 | 935 | 0 | 255 | 212.5 | 21.2 | 17 | 0 | 0 | 0 | 0 | 0.200 | 1 | 23 | 1 | 1 | 4 | 10 | 0.44 | 1 | 28 | 121.6 |
| 1534 | 1251.2 | 291.3 | 407.8 | 0 | 0 | 201.4 | 28.2 | 0 | 0 | 0 | 0 | 0 | 0.131 | 1 | 23 | 1 | 1 | 0 | 0 | 0 | 1 | 7 | 119.5 |
| 1535 | 1251.2 | 291.3 | 407.8 | 0 | 0 | 201.4 | 28.2 | 0 | 0 | 0 | 0 | 0 | 0.131 | 1 | 23 | 1 | 1 | 0 | 0 | 0 | 1 | 14 | 135.1 |
| 1536 | 1251.2 | 291.3 | 407.8 | 0 | 0 | 201.4 | 28.2 | 0 | 0 | 0 | 0 | 0 | 0.131 | 1 | 23 | 1 | 1 | 0 | 0 | 0 | 1 | 28 | 141.3 |
| 1537 | 1251.2 | 291.3 | 407.8 | 0 | 0 | 201.4 | 28.2 | 0 | 0 | 0 | 0 | 0 | 0.131 | 1 | 23 | 1 | 1 | 0 | 0 | 0 | 1 | 56 | 149.2 |
| 1538 | 700 | 50 | 1104 | 0 | 0 | 180 | 30 | 0 | 150 | 0 | 0 | 0 | 0.240 | 1 | 23 | 1 | 1 | 0 | 13 | 0.2 | 1 | 28 | 102.4 |
| 1539 | 700 | 50 | 1104 | 0 | 0 | 180 | 30 | 3.5 | 150 | 0 | 0 | 0 | 0.240 | 1 | 23 | 1 | 1 | 1 | 13 | 0.2 | 1 | 28 | 111.7 |
| 1540 | 700 | 50 | 1104 | 0 | 0 | 180 | 30 | 7 | 150 | 0 | 0 | 0 | 0.240 | 1 | 23 | 1 | 1 | 1 | 13 | 0.2 | 1 | 28 | 138.3 |
| 1541 | 700 | 50 | 1104 | 0 | 0 | 180 | 30 | 10.5 | 150 | 0 | 0 | 0 | 0.240 | 1 | 23 | 1 | 1 | 1 | 13 | 0.2 | 1 | 28 | 152.9 |
| 1542 | 700 | 50 | 1104 | 0 | 0 | 180 | 30 | 14 | 150 | 0 | 0 | 0 | 0.240 | 1 | 23 | 1 | 1 | 1 | 13 | 0.2 | 1 | 28 | 156.1 |
| 1543 | 700 | 50 | 1104 | 0 | 0 | 180 | 30 | 17.5 | 150 | 0 | 0 | 0 | 0.240 | 1 | 23 | 1 | 1 | 1 | 13 | 0.2 | 1 | 28 | 158.7 |
| 1544 | 850 | 212.5 | 935 | 0 | 255 | 212.5 | 21.2 | 25.5 | 0 | 0 | 0 | 0 | 0.200 | 1 | 23 | 1 | 1 | 4 | 10 | 0.44 | 1 | 28 | 131.1 |
| 1545 | 700 | 50 | 1104 | 0 | 0 | 180 | 30 | 0 | 150 | 0 | 0 | 0 | 0.240 | 1 | 23 | 1 | 1 | 0 | 13 | 0.2 | 1 | 7 | 91.7 |
| 1546 | 700 | 50 | 1104 | 0 | 0 | 180 | 30 | 3.5 | 150 | 0 | 0 | 0 | 0.240 | 1 | 23 | 1 | 1 | 1 | 13 | 0.2 | 1 | 7 | 93.9 |
| 1547 | 700 | 50 | 1104 | 0 | 0 | 180 | 30 | 7 | 150 | 0 | 0 | 0 | 0.240 | 1 | 23 | 1 | 1 | 1 | 13 | 0.2 | 1 | 7 | 96.2 |
| 1548 | 700 | 50 | 1104 | 0 | 0 | 180 | 30 | 10.5 | 150 | 0 | 0 | 0 | 0.240 | 1 | 23 | 1 | 1 | 1 | 13 | 0.2 | 1 | 7 | 113 |
| 1549 | 700 | 50 | 1104 | 0 | 0 | 180 | 30 | 14 | 150 | 0 | 0 | 0 | 0.240 | 1 | 23 | 1 | 1 | 1 | 13 | 0.2 | 1 | 7 | 115 |
| 1550 | 700 | 50 | 1104 | 0 | 0 | 180 | 30 | 17.5 | 150 | 0 | 0 | 0 | 0.240 | 1 | 23 | 1 | 1 | 1 | 13 | 0.2 | 1 | 7 | 117.9 |
| 1551 | 700 | 50 | 1104 | 0 | 0 | 180 | 30 | 0 | 150 | 0 | 0 | 0 | 0.240 | 1 | 23 | 1 | 1 | 0 | 13 | 0.2 | 1 | 1 | 55.7 |
| 1552 | 700 | 50 | 1104 | 0 | 0 | 180 | 30 | 3.5 | 150 | 0 | 0 | 0 | 0.240 | 1 | 23 | 1 | 1 | 1 | 13 | 0.2 | 1 | 1 | 65.4 |
| 1553 | 700 | 50 | 1104 | 0 | 0 | 180 | 30 | 7 | 150 | 0 | 0 | 0 | 0.240 | 1 | 23 | 1 | 1 | 1 | 13 | 0.2 | 1 | 1 | 74.2 |
| 1554 | 700 | 50 | 1104 | 0 | 0 | 180 | 30 | 10.5 | 150 | 0 | 0 | 0 | 0.240 | 1 | 23 | 1 | 1 | 1 | 13 | 0.2 | 1 | 1 | 79 |
| 1555 | 850 | 212.5 | 935 | 0 | 255 | 212.5 | 21.2 | 8.5 | 0 | 0 | 0 | 0 | 0.200 | 1 | 23 | 1 | 1 | 4 | 10 | 0.86 | 1 | 28 | 115.1 |
| 1556 | 700 | 50 | 1104 | 0 | 0 | 180 | 30 | 14 | 150 | 0 | 0 | 0 | 0.240 | 1 | 23 | 1 | 1 | 1 | 13 | 0.2 | 1 | 1 | 84.2 |
| 1557 | 700 | 50 | 1104 | 0 | 0 | 180 | 30 | 17.5 | 150 | 0 | 0 | 0 | 0.240 | 1 | 23 | 1 | 1 | 1 | 13 | 0.2 | 1 | 1 | 88.1 |
| 1558 | 680.27 | 119.73 | 1462.59 | 0 | 0 | 160 | 9.59 | 0 | 0 | 0 | 0 | 0 | 0.200 | 1 | 23 | 1 | 1 | 0 | 0 | 0 | 1 | 28 | 60.8 |
| 1559 | 640 | 160 | 1446.4 | 0 | 0 | 160 | 9.6 | 0 | 0 | 0 | 0 | 0 | 0.200 | 1 | 23 | 1 | 1 | 0 | 0 | 0 | 1 | 28 | 56 |
| 1560 | 600.15 | 199.85 | 1434.36 | 0 | 0 | 160 | 9.6 | 0 | 0 | 0 | 0 | 0 | 0.200 | 1 | 23 | 1 | 1 | 0 | 0 | 0 | 1 | 28 | 58.5 |
| 1561 | 680.27 | 119.73 | 1503.4 | 0 | 0 | 144 | 9.59 | 0 | 0 | 0 | 0 | 0 | 0.180 | 1 | 23 | 1 | 1 | 0 | 0 | 0 | 1 | 28 | 67.3 |
| 1562 | 680.27 | 119.73 | 1421.77 | 0 | 0 | 176 | 9.59 | 0 | 0 | 0 | 0 | 0 | 0.220 | 1 | 23 | 1 | 1 | 0 | 0 | 0 | 1 | 28 | 54.8 |
| 1563 | 680.27 | 119.73 | 1462.59 | 0 | 0 | 160 | 11.22 | 0 | 0 | 0 | 0 | 0 | 0.200 | 1 | 23 | 1 | 1 | 0 | 0 | 0 | 1 | 28 | 62.2 |
| 1564 | 680.27 | 119.73 | 1469.39 | 0 | 0 | 160 | 8.03 | 0 | 0 | 0 | 0 | 0 | 0.200 | 1 | 23 | 1 | 1 | 0 | 0 | 0 | 1 | 28 | 58.1 |
| 1565 | 765.31 | 134.69 | 1316.33 | 0 | 0 | 180 | 10.79 | 0 | 0 | 0 | 0 | 0 | 0.200 | 1 | 23 | 1 | 1 | 0 | 0 | 0 | 1 | 28 | 64.3 |
| 1566 | 850 | 212.5 | 935 | 0 | 255 | 212.5 | 21.2 | 17 | 0 | 0 | 0 | 0 | 0.200 | 1 | 23 | 1 | 1 | 4 | 10 | 0.86 | 1 | 28 | 117.9 |
| 1567 | 720 | 180 | 1303.2 | 0 | 0 | 180 | 10.8 | 0 | 0 | 0 | 0 | 0 | 0.200 | 1 | 23 | 1 | 1 | 0 | 0 | 0 | 1 | 28 | 67.1 |
| 1568 | 675.17 | 224.83 | 1289.57 | 0 | 0 | 180 | 10.8 | 0 | 0 | 0 | 0 | 0 | 0.200 | 1 | 23 | 1 | 1 | 0 | 0 | 0 | 1 | 28 | 62.5 |
| 1569 | 720 | 180 | 1353.6 | 0 | 0 | 162 | 10.8 | 0 | 0 | 0 | 0 | 0 | 0.180 | 1 | 23 | 1 | 1 | 0 | 0 | 0 | 1 | 28 | 70.1 |
| 1570 | 720 | 180 | 1260 | 0 | 0 | 198 | 10.8 | 0 | 0 | 0 | 0 | 0 | 0.220 | 1 | 23 | 1 | 1 | 0 | 0 | 0 | 1 | 28 | 62.4 |
| 1571 | 720 | 180 | 1296 | 0 | 0 | 180 | 12.6 | 0 | 0 | 0 | 0 | 0 | 0.200 | 1 | 23 | 1 | 1 | 0 | 0 | 0 | 1 | 28 | 68.4 |
| 1572 | 720 | 180 | 1310.4 | 0 | 0 | 180 | 9 | 0 | 0 | 0 | 0 | 0 | 0.200 | 1 | 23 | 1 | 1 | 0 | 0 | 0 | 1 | 28 | 66.1 |
| 1573 | 850.34 | 149.66 | 1173.47 | 0 | 0 | 200 | 11.99 | 0 | 0 | 0 | 0 | 0 | 0.200 | 1 | 23 | 1 | 1 | 0 | 0 | 0 | 1 | 28 | 70.2 |
| 1574 | 800 | 200 | 1160 | 0 | 0 | 200 | 12 | 0 | 0 | 0 | 0 | 0 | 0.200 | 1 | 23 | 1 | 1 | 0 | 0 | 0 | 1 | 28 | 70.8 |
| 1575 | 750.19 | 249.81 | 1140.29 | 0 | 0 | 200 | 12 | 0 | 0 | 0 | 0 | 0 | 0.200 | 1 | 23 | 1 | 1 | 0 | 0 | 0 | 1 | 28 | 66 |
| 1576 | 850.34 | 149.66 | 1224.49 | 0 | 0 | 180 | 11.99 | 0 | 0 | 0 | 0 | 0 | 0.180 | 1 | 23 | 1 | 1 | 0 | 0 | 0 | 1 | 28 | 72.7 |
| 1577 | 850 | 212.5 | 935 | 0 | 255 | 212.5 | 21.2 | 25.5 | 0 | 0 | 0 | 0 | 0.200 | 1 | 23 | 1 | 1 | 4 | 10 | 0.86 | 1 | 28 | 121.7 |
| 1578 | 850.34 | 149.66 | 1122.45 | 0 | 0 | 220 | 11.99 | 0 | 0 | 0 | 0 | 0 | 0.220 | 1 | 23 | 1 | 1 | 0 | 0 | 0 | 1 | 28 | 67 |
| 1579 | 850.34 | 149.66 | 1173.47 | 0 | 0 | 200 | 14.03 | 0 | 0 | 0 | 0 | 0 | 0.200 | 1 | 23 | 1 | 1 | 0 | 0 | 0 | 1 | 28 | 71.3 |
| 1580 | 850.34 | 149.66 | 1181.97 | 0 | 0 | 200 | 10.03 | 0 | 0 | 0 | 0 | 0 | 0.200 | 1 | 23 | 1 | 1 | 0 | 0 | 0 | 1 | 28 | 67.9 |
| 1581 | 680.27 | 119.73 | 1462.59 | 0 | 0 | 160 | 9.59 | 0 | 0 | 0 | 0 | 0 | 0.200 | 1 | 23 | 1 | 1 | 0 | 0 | 0 | 1 | 7 | 115 |
| 1582 | 640 | 160 | 1446.4 | 0 | 0 | 160 | 9.6 | 0 | 0 | 0 | 0 | 0 | 0.200 | 1 | 23 | 1 | 1 | 0 | 0 | 0 | 1 | 7 | 109.6 |
| 1583 | 600.15 | 199.85 | 1434.36 | 0 | 0 | 160 | 9.6 | 0 | 0 | 0 | 0 | 0 | 0.200 | 1 | 23 | 1 | 1 | 0 | 0 | 0 | 1 | 7 | 102.6 |
| 1584 | 680.27 | 119.73 | 1503.4 | 0 | 0 | 144 | 9.59 | 0 | 0 | 0 | 0 | 0 | 0.180 | 1 | 23 | 1 | 1 | 0 | 0 | 0 | 1 | 7 | 119.8 |
| 1585 | 680.27 | 119.73 | 1421.77 | 0 | 0 | 176 | 9.59 | 0 | 0 | 0 | 0 | 0 | 0.220 | 1 | 23 | 1 | 1 | 0 | 0 | 0 | 1 | 7 | 105.7 |
| 1586 | 680.27 | 119.73 | 1462.59 | 0 | 0 | 160 | 11.22 | 0 | 0 | 0 | 0 | 0 | 0.200 | 1 | 23 | 1 | 1 | 0 | 0 | 0 | 1 | 7 | 117.7 |
| 1587 | 680.27 | 119.73 | 1469.39 | 0 | 0 | 160 | 8.03 | 0 | 0 | 0 | 0 | 0 | 0.200 | 1 | 23 | 1 | 1 | 0 | 0 | 0 | 1 | 7 | 108.7 |
| 1588 | 797.78 | 112.36 | 1123.63 | 0 | 0 | 224.73 | 28.09 | 7.9778 | 44.95 | 112.36 | 56.18 | 0 | 0.247 | 1 | 23 | 1 | 1 | 4 | 18 | 0.013 | 1 | 14 | 108.4 |
| 1589 | 850 | 212.5 | 935 | 0 | 255 | 212.5 | 21.2 | 25.5 | 0 | 0 | 0 | 0 | 0.200 | 1 | 23 | 1 | 1 | 3 | 10 | 0.4 | 1 | 28 | 105.3 |
| 1590 | 765.31 | 134.69 | 1316.33 | 0 | 0 | 180 | 10.79 | 0 | 0 | 0 | 0 | 0 | 0.200 | 1 | 23 | 1 | 1 | 0 | 0 | 0 | 1 | 7 | 118.8 |
| 1591 | 720 | 180 | 1303.2 | 0 | 0 | 180 | 10.8 | 0 | 0 | 0 | 0 | 0 | 0.200 | 1 | 23 | 1 | 1 | 0 | 0 | 0 | 1 | 7 | 120.6 |
| 1592 | 675.17 | 224.83 | 1289.57 | 0 | 0 | 180 | 10.8 | 0 | 0 | 0 | 0 | 0 | 0.200 | 1 | 23 | 1 | 1 | 0 | 0 | 0 | 1 | 7 | 119.7 |
| 1593 | 720 | 180 | 1353.6 | 0 | 0 | 162 | 10.8 | 0 | 0 | 0 | 0 | 0 | 0.180 | 1 | 23 | 1 | 1 | 0 | 0 | 0 | 1 | 7 | 124.3 |
| 1594 | 720 | 180 | 1260 | 0 | 0 | 198 | 10.8 | 0 | 0 | 0 | 0 | 0 | 0.220 | 1 | 23 | 1 | 1 | 0 | 0 | 0 | 1 | 7 | 119.3 |
| 1595 | 720 | 180 | 1296 | 0 | 0 | 180 | 12.6 | 0 | 0 | 0 | 0 | 0 | 0.200 | 1 | 23 | 1 | 1 | 0 | 0 | 0 | 1 | 7 | 124 |
| 1596 | 720 | 180 | 1310.4 | 0 | 0 | 180 | 9 | 0 | 0 | 0 | 0 | 0 | 0.200 | 1 | 23 | 1 | 1 | 0 | 0 | 0 | 1 | 7 | 122.3 |
| 1597 | 850.34 | 149.66 | 1173.47 | 0 | 0 | 200 | 11.99 | 0 | 0 | 0 | 0 | 0 | 0.200 | 1 | 23 | 1 | 1 | 0 | 0 | 0 | 1 | 7 | 124 |
| 1598 | 800 | 200 | 1160 | 0 | 0 | 200 | 12 | 0 | 0 | 0 | 0 | 0 | 0.200 | 1 | 23 | 1 | 1 | 0 | 0 | 0 | 1 | 7 | 123.5 |
| 1599 | 750.19 | 249.81 | 1140.29 | 0 | 0 | 200 | 12 | 0 | 0 | 0 | 0 | 0 | 0.200 | 1 | 23 | 1 | 1 | 0 | 0 | 0 | 1 | 7 | 120.8 |
| 1600 | 850 | 212.5 | 935 | 0 | 255 | 212.5 | 21.2 | 25.5 | 0 | 0 | 0 | 0 | 0.200 | 1 | 23 | 1 | 1 | 1 | 12 | 0.2 | 1 | 28 | 148.7 |
| 1601 | 850.34 | 149.66 | 1224.49 | 0 | 0 | 180 | 11.99 | 0 | 0 | 0 | 0 | 0 | 0.180 | 1 | 23 | 1 | 1 | 0 | 0 | 0 | 1 | 7 | 128.9 |
| 1602 | 850.34 | 149.66 | 1122.45 | 0 | 0 | 220 | 11.99 | 0 | 0 | 0 | 0 | 0 | 0.220 | 1 | 23 | 1 | 1 | 0 | 0 | 0 | 1 | 7 | 118.8 |
| 1603 | 850.34 | 149.66 | 1173.47 | 0 | 0 | 200 | 14.03 | 0 | 0 | 0 | 0 | 0 | 0.200 | 1 | 23 | 1 | 1 | 0 | 0 | 0 | 1 | 7 | 126.2 |
| 1604 | 850.34 | 149.66 | 1181.97 | 0 | 0 | 200 | 10.03 | 0 | 0 | 0 | 0 | 0 | 0.200 | 1 | 23 | 1 | 1 | 0 | 0 | 0 | 1 | 7 | 123.5 |
| 1605 | 800 | 0 | 1471.3 | 0 | 0 | 160 | 21.6 | 0 | 0 | 0 | 0 | 0 | 0.200 | 1 | 23 | 1 | 1 | 0 | 0 | 0 | 1 | 1 | 72.9 |
| 1606 | 800 | 0 | 1471.3 | 0 | 0 | 160 | 21.6 | 0 | 0 | 0 | 0 | 0 | 0.200 | 1 | 23 | 1 | 1 | 0 | 0 | 0 | 1 | 3 | 88.9 |
| 1607 | 800 | 0 | 1471.3 | 0 | 0 | 160 | 21.6 | 0 | 0 | 0 | 0 | 0 | 0.200 | 1 | 23 | 1 | 1 | 0 | 0 | 0 | 1 | 7 | 94.4 |
| 1608 | 800 | 0 | 1471.3 | 0 | 0 | 160 | 21.6 | 0 | 0 | 0 | 0 | 0 | 0.200 | 1 | 23 | 1 | 1 | 0 | 0 | 0 | 1 | 14 | 100.4 |
| 1609 | 800 | 0 | 1471.3 | 0 | 0 | 160 | 21.6 | 0 | 0 | 0 | 0 | 0 | 0.200 | 1 | 23 | 1 | 1 | 0 | 0 | 0 | 1 | 28 | 104.7 |
| 1610 | 800 | 0 | 1471.3 | 0 | 0 | 160 | 21.6 | 0 | 0 | 0 | 0 | 0 | 0.200 | 1 | 23 | 1 | 1 | 0 | 0 | 0 | 1 | 56 | 110.1 |
| 1611 | 850 | 212.5 | 935 | 0 | 255 | 212.5 | 21.2 | 0 | 0 | 0 | 0 | 0 | 0.200 | 1 | 23 | 1 | 1 | 0 | 0 | 0 | 1 | 28 | 118.9 |
| 1612 | 800 | 0 | 1471.3 | 0 | 0 | 160 | 21.6 | 0 | 0 | 0 | 0 | 0 | 0.200 | 1 | 23 | 1 | 1 | 0 | 0 | 0 | 1 | 90 | 116.7 |
| 1613 | 720 | 80 | 1422.9 | 0 | 0 | 160 | 29.6 | 0 | 0 | 0 | 0 | 0 | 0.200 | 1 | 23 | 1 | 1 | 0 | 0 | 0 | 1 | 1 | 78.7 |
| 1614 | 720 | 80 | 1422.9 | 0 | 0 | 160 | 29.6 | 0 | 0 | 0 | 0 | 0 | 0.200 | 1 | 23 | 1 | 1 | 0 | 0 | 0 | 1 | 3 | 87.7 |
| 1615 | 720 | 80 | 1422.9 | 0 | 0 | 160 | 29.6 | 0 | 0 | 0 | 0 | 0 | 0.200 | 1 | 23 | 1 | 1 | 0 | 0 | 0 | 1 | 7 | 100.4 |
| 1616 | 720 | 80 | 1422.9 | 0 | 0 | 160 | 29.6 | 0 | 0 | 0 | 0 | 0 | 0.200 | 1 | 23 | 1 | 1 | 0 | 0 | 0 | 1 | 14 | 111.6 |
| 1617 | 720 | 80 | 1422.9 | 0 | 0 | 160 | 29.6 | 0 | 0 | 0 | 0 | 0 | 0.200 | 1 | 23 | 1 | 1 | 0 | 0 | 0 | 1 | 28 | 116.4 |
| 1618 | 720 | 80 | 1422.9 | 0 | 0 | 160 | 29.6 | 0 | 0 | 0 | 0 | 0 | 0.200 | 1 | 23 | 1 | 1 | 0 | 0 | 0 | 1 | 56 | 119.8 |
| 1619 | 720 | 80 | 1422.9 | 0 | 0 | 160 | 29.6 | 0 | 0 | 0 | 0 | 0 | 0.200 | 1 | 23 | 1 | 1 | 0 | 0 | 0 | 1 | 90 | 125.3 |
| 1620 | 741 | 185 | 815 | 0 | 259 | 185 | 9 | 14.82 | 0 | 0 | 0 | 0 | 0.200 | 1 | 23 | 1 | 1 | 1 | 13 | 0.2 | 1 | 3 | 81.9 |
| 1621 | 741 | 185 | 815 | 0 | 259 | 185 | 9 | 14.82 | 0 | 0 | 0 | 0 | 0.200 | 1 | 23 | 1 | 1 | 1 | 13 | 0.2 | 1 | 7 | 121.3 |
| 1622 | 850 | 212.5 | 935 | 0 | 255 | 212.5 | 21.2 | 8.5 | 0 | 0 | 0 | 0 | 0.200 | 1 | 23 | 1 | 1 | 4 | 10 | 0.44 | 1 | 28 | 125.5 |
| 1623 | 741 | 185 | 815 | 0 | 259 | 185 | 9 | 14.82 | 0 | 0 | 0 | 0 | 0.200 | 1 | 23 | 1 | 1 | 1 | 13 | 0.2 | 1 | 28 | 154.9 |
| 1624 | 741 | 185 | 815 | 0 | 259 | 185 | 9 | 14.82 | 0 | 0 | 0 | 0 | 0.200 | 1 | 23 | 1 | 1 | 1 | 13 | 0.2 | 1 | 91 | 150.4 |
| 1625 | 792 | 264 | 1056 | 0 | 0 | 173 | 21.1 | 15.84 | 0 | 0 | 0 | 0 | 0.164 | 1 | 23 | 1 | 1 | 1 | 13 | 0.2 | 1 | 3 | 99 |
| 1626 | 634 | 264 | 1056 | 0 | 0 | 173 | 21.1 | 12.68 | 0 | 159 | 0 | 0 | 0.193 | 1 | 23 | 1 | 1 | 1 | 13 | 0.2 | 1 | 3 | 92.6 |
| 1627 | 475 | 264 | 1056 | 0 | 0 | 173 | 21.1 | 9.5 | 0 | 317 | 0 | 0 | 0.234 | 1 | 23 | 1 | 1 | 1 | 13 | 0.2 | 1 | 3 | 99.1 |
| 1628 | 317 | 264 | 1056 | 0 | 0 | 173 | 21.1 | 6.34 | 0 | 475 | 0 | 0 | 0.298 | 1 | 23 | 1 | 1 | 1 | 13 | 0.2 | 1 | 3 | 94.3 |
| 1629 | 792 | 264 | 1056 | 0 | 0 | 173 | 21.1 | 15.84 | 0 | 0 | 0 | 0 | 0.164 | 1 | 23 | 1 | 1 | 1 | 13 | 0.2 | 1 | 3 | 99.8 |
| 1630 | 634 | 264 | 1056 | 0 | 0 | 173 | 21.1 | 12.68 | 159 | 0 | 0 | 0 | 0.193 | 1 | 23 | 1 | 1 | 1 | 13 | 0.2 | 1 | 3 | 84.8 |
| 1631 | 475 | 264 | 1056 | 0 | 0 | 173 | 21.1 | 9.5 | 317 | 0 | 0 | 0 | 0.234 | 1 | 23 | 1 | 1 | 1 | 13 | 0.2 | 1 | 3 | 81.1 |
| 1632 | 317 | 264 | 1056 | 0 | 0 | 173 | 21.1 | 6.34 | 475 | 0 | 0 | 0 | 0.298 | 1 | 23 | 1 | 1 | 1 | 13 | 0.2 | 1 | 3 | 71.5 |
| 1633 | 850 | 212.5 | 935 | 0 | 255 | 212.5 | 21.2 | 17 | 0 | 0 | 0 | 0 | 0.200 | 1 | 23 | 1 | 1 | 4 | 10 | 0.44 | 1 | 28 | 132.1 |
| 1634 | 792 | 264 | 1056 | 0 | 0 | 173 | 21.1 | 15.84 | 0 | 0 | 0 | 0 | 0.164 | 1 | 23 | 1 | 1 | 1 | 13 | 0.2 | 1 | 7 | 119.4 |
| 1635 | 634 | 264 | 1056 | 0 | 0 | 173 | 21.1 | 12.68 | 0 | 159 | 0 | 0 | 0.193 | 1 | 23 | 1 | 1 | 1 | 13 | 0.2 | 1 | 7 | 117.3 |
| 1636 | 475 | 264 | 1056 | 0 | 0 | 173 | 21.1 | 9.5 | 0 | 317 | 0 | 0 | 0.234 | 1 | 23 | 1 | 1 | 1 | 13 | 0.2 | 1 | 7 | 119 |
| 1637 | 317 | 264 | 1056 | 0 | 0 | 173 | 21.1 | 6.34 | 0 | 475 | 0 | 0 | 0.298 | 1 | 23 | 1 | 1 | 1 | 13 | 0.2 | 1 | 7 | 115.8 |
| 1638 | 792 | 264 | 1056 | 0 | 0 | 173 | 21.1 | 15.84 | 0 | 0 | 0 | 0 | 0.164 | 1 | 23 | 1 | 1 | 1 | 13 | 0.2 | 1 | 7 | 119.7 |
| 1639 | 634 | 264 | 1056 | 0 | 0 | 173 | 21.1 | 12.68 | 159 | 0 | 0 | 0 | 0.193 | 1 | 23 | 1 | 1 | 1 | 13 | 0.2 | 1 | 7 | 108.5 |
| 1640 | 475 | 264 | 1056 | 0 | 0 | 173 | 21.1 | 9.5 | 317 | 0 | 0 | 0 | 0.234 | 1 | 23 | 1 | 1 | 1 | 13 | 0.2 | 1 | 7 | 102.6 |
| 1641 | 317 | 264 | 1056 | 0 | 0 | 173 | 21.1 | 6.34 | 475 | 0 | 0 | 0 | 0.298 | 1 | 23 | 1 | 1 | 1 | 13 | 0.2 | 1 | 7 | 93.5 |
| 1642 | 792 | 264 | 1056 | 0 | 0 | 173 | 21.1 | 15.84 | 0 | 0 | 0 | 0 | 0.164 | 1 | 23 | 1 | 1 | 1 | 13 | 0.2 | 1 | 28 | 143.6 |
| 1643 | 634 | 264 | 1056 | 0 | 0 | 173 | 21.1 | 12.68 | 0 | 159 | 0 | 0 | 0.193 | 1 | 23 | 1 | 1 | 1 | 13 | 0.2 | 1 | 28 | 136.7 |
| 1644 | 850 | 212.5 | 935 | 0 | 255 | 212.5 | 21.2 | 25.5 | 0 | 0 | 0 | 0 | 0.200 | 1 | 23 | 1 | 1 | 4 | 10 | 0.44 | 1 | 28 | 139.1 |
| 1645 | 475 | 264 | 1056 | 0 | 0 | 173 | 21.1 | 9.5 | 0 | 317 | 0 | 0 | 0.234 | 1 | 23 | 1 | 1 | 1 | 13 | 0.2 | 1 | 28 | 138.9 |
| 1646 | 317 | 264 | 1056 | 0 | 0 | 173 | 21.1 | 6.34 | 0 | 475 | 0 | 0 | 0.298 | 1 | 23 | 1 | 1 | 1 | 13 | 0.2 | 1 | 28 | 133.6 |
| 1647 | 792 | 264 | 1056 | 0 | 0 | 173 | 21.1 | 15.84 | 0 | 0 | 0 | 0 | 0.164 | 1 | 23 | 1 | 1 | 1 | 13 | 0.2 | 1 | 28 | 144.4 |
| 1648 | 634 | 264 | 1056 | 0 | 0 | 173 | 21.1 | 12.68 | 159 | 0 | 0 | 0 | 0.193 | 1 | 23 | 1 | 1 | 1 | 13 | 0.2 | 1 | 28 | 133.7 |
| 1649 | 475 | 264 | 1056 | 0 | 0 | 173 | 21.1 | 9.5 | 317 | 0 | 0 | 0 | 0.234 | 1 | 23 | 1 | 1 | 1 | 13 | 0.2 | 1 | 28 | 139.2 |
| 1650 | 317 | 264 | 1056 | 0 | 0 | 173 | 21.1 | 6.34 | 475 | 0 | 0 | 0 | 0.298 | 1 | 23 | 1 | 1 | 1 | 13 | 0.2 | 1 | 28 | 140.9 |
| 1651 | 792 | 264 | 1056 | 0 | 0 | 173 | 21.1 | 15.84 | 0 | 0 | 0 | 0 | 0.164 | 1 | 23 | 1 | 1 | 1 | 13 | 0.2 | 1 | 91 | 156.5 |
| 1652 | 634 | 264 | 1056 | 0 | 0 | 173 | 21.1 | 12.68 | 0 | 159 | 0 | 0 | 0.193 | 1 | 23 | 1 | 1 | 1 | 13 | 0.2 | 1 | 91 | 155.5 |
| 1653 | 475 | 264 | 1056 | 0 | 0 | 173 | 21.1 | 9.5 | 0 | 317 | 0 | 0 | 0.234 | 1 | 23 | 1 | 1 | 1 | 13 | 0.2 | 1 | 91 | 156.1 |
| 1654 | 317 | 264 | 1056 | 0 | 0 | 173 | 21.1 | 6.34 | 0 | 475 | 0 | 0 | 0.298 | 1 | 23 | 1 | 1 | 1 | 13 | 0.2 | 1 | 91 | 151.3 |
| 1655 | 850 | 212.5 | 935 | 0 | 255 | 212.5 | 21.2 | 8.5 | 0 | 0 | 0 | 0 | 0.200 | 1 | 23 | 1 | 1 | 4 | 10 | 0.86 | 1 | 28 | 121.1 |
| 1656 | 792 | 264 | 1056 | 0 | 0 | 173 | 21.1 | 15.84 | 0 | 0 | 0 | 0 | 0.164 | 1 | 23 | 1 | 1 | 1 | 13 | 0.2 | 1 | 91 | 156.8 |
| 1657 | 634 | 264 | 1056 | 0 | 0 | 173 | 21.1 | 12.68 | 159 | 0 | 0 | 0 | 0.193 | 1 | 23 | 1 | 1 | 1 | 13 | 0.2 | 1 | 91 | 149.9 |
| 1658 | 475 | 264 | 1056 | 0 | 0 | 173 | 21.1 | 9.5 | 317 | 0 | 0 | 0 | 0.234 | 1 | 23 | 1 | 1 | 1 | 13 | 0.2 | 1 | 91 | 154.3 |
| 1659 | 317 | 264 | 1056 | 0 | 0 | 173 | 21.1 | 6.34 | 475 | 0 | 0 | 0 | 0.298 | 1 | 23 | 1 | 1 | 1 | 13 | 0.2 | 1 | 91 | 150 |
| 1660 | 850 | 212.5 | 935 | 0 | 255 | 212.5 | 21.2 | 17 | 0 | 0 | 0 | 0 | 0.200 | 1 | 23 | 1 | 1 | 4 | 10 | 0.86 | 1 | 28 | 126.3 |
| 1661 | 850 | 212.5 | 935 | 0 | 255 | 212.5 | 21.2 | 25.5 | 0 | 0 | 0 | 0 | 0.200 | 1 | 23 | 1 | 1 | 4 | 10 | 0.86 | 1 | 28 | 131.2 |
| 1662 | 850 | 212.5 | 935 | 0 | 255 | 212.5 | 21.2 | 25.5 | 0 | 0 | 0 | 0 | 0.200 | 1 | 23 | 1 | 1 | 3 | 10 | 0.4 | 1 | 28 | 110.9 |
| 1663 | 797.78 | 112.36 | 1123.63 | 0 | 0 | 224.73 | 28.09 | 7.9778 | 44.95 | 112.36 | 56.18 | 0 | 0.247 | 1 | 23 | 1 | 1 | 4 | 18 | 0.013 | 1 | 28 | 120.9 |
| 1664 | 850 | 212.5 | 935 | 0 | 255 | 212.5 | 21.2 | 25.5 | 0 | 0 | 0 | 0 | 0.200 | 1 | 23 | 1 | 1 | 1 | 12 | 0.2 | 1 | 28 | 159.1 |
| 1665 | 850 | 212.5 | 935 | 0 | 255 | 212.5 | 21.2 | 0 | 0 | 0 | 0 | 0 | 0.200 | 1 | 23 | 1 | 1 | 0 | 0 | 0 | 1 | 28 | 123.2 |
| 1666 | 850 | 212.5 | 935 | 0 | 255 | 212.5 | 21.2 | 8.5 | 0 | 0 | 0 | 0 | 0.200 | 1 | 23 | 1 | 1 | 4 | 10 | 0.44 | 1 | 28 | 127.4 |
| 1667 | 850 | 212.5 | 935 | 0 | 255 | 212.5 | 21.2 | 17 | 0 | 0 | 0 | 0 | 0.200 | 1 | 23 | 1 | 1 | 4 | 10 | 0.44 | 1 | 28 | 143 |
| 1668 | 850 | 212.5 | 935 | 0 | 255 | 212.5 | 21.2 | 25.5 | 0 | 0 | 0 | 0 | 0.200 | 1 | 23 | 1 | 1 | 4 | 10 | 0.44 | 1 | 28 | 147.2 |
| 1669 | 850 | 212.5 | 935 | 0 | 255 | 212.5 | 21.2 | 8.5 | 0 | 0 | 0 | 0 | 0.200 | 1 | 23 | 1 | 1 | 4 | 10 | 0.86 | 1 | 28 | 128 |
| 1670 | 850 | 212.5 | 935 | 0 | 255 | 212.5 | 21.2 | 17 | 0 | 0 | 0 | 0 | 0.200 | 1 | 23 | 1 | 1 | 4 | 10 | 0.86 | 1 | 28 | 135.8 |
| 1671 | 850 | 212.5 | 935 | 0 | 255 | 212.5 | 21.2 | 25.5 | 0 | 0 | 0 | 0 | 0.200 | 1 | 23 | 1 | 1 | 4 | 10 | 0.86 | 1 | 28 | 140.8 |
| 1672 | 850 | 212.5 | 935 | 0 | 255 | 212.5 | 21.2 | 25.5 | 0 | 0 | 0 | 0 | 0.200 | 1 | 23 | 1 | 1 | 3 | 10 | 0.4 | 1 | 28 | 116.5 |
| 1673 | 850 | 212.5 | 935 | 0 | 255 | 212.5 | 21.2 | 25.5 | 0 | 0 | 0 | 0 | 0.200 | 1 | 23 | 1 | 1 | 1 | 12 | 0.2 | 1 | 28 | 169.1 |
| 1674 | 805 | 224 | 971 | 0 | 0 | 195 | 15 | 0 | 0 | 0 | 0 | 0 | 0.190 | 1 | 23 | 1 | 1 | 0 | 0 | 0 | 1 | 28 | 110 |
| 1675 | 805 | 224 | 971 | 0 | 0 | 195 | 15 | 0 | 0 | 0 | 0 | 0 | 0.190 | 1 | 23 | 1 | 1 | 0 | 0 | 0 | 1 | 7 | 89.5 |
| 1676 | 805 | 224 | 971 | 0 | 0 | 195 | 15 | 0 | 0 | 0 | 0 | 0 | 0.190 | 2 | 90 | 1 | 1 | 0 | 0 | 0 | 1 | 28 | 130 |
| 1677 | 805 | 224 | 971 | 0 | 0 | 195 | 15 | 0 | 0 | 0 | 0 | 0 | 0.190 | 2 | 90 | 1 | 1 | 0 | 0 | 0 | 1 | 7 | 100 |
| 1678 | 955 | 239 | 1051 | 0 | 0 | 143 | 15 | 0 | 0 | 0 | 0 | 0 | 0.120 | 1 | 20 | 5 | 2 | 0 | 0 | 0 | 1 | 28 | 170 |
| 1679 | 955 | 220 | 1051 | 0 | 372 | 162 | 18 | 0 | 0 | 0 | 0 | 0 | 0.138 | 2 | 90 | 5 | 2 | 0 | 0 | 0 | 1 | 28 | 270 |
| 1680 | 955 | 239 | 1051 | 0 | 0 | 162 | 15 | 167 | 0 | 0 | 0 | 0 | 0.136 | 1 | 20 | 5 | 2 | 1 | 12 | 0.18 | 1 | 28 | 270 |
| 1681 | 955 | 220 | 1051 | 0 | 372 | 181 | 18 | 167 | 0 | 0 | 0 | 0 | 0.154 | 2 | 90 | 5 | 2 | 1 | 12 | 0.18 | 1 | 28 | 260 |
| 1682 | 750 | 158 | 444 | 0 | 662 | 189 | 37.5 | 0 | 0 | 0 | 0 | 0 | 0.208 | 1 | 20 | 4 | 1 | 0 | 0 | 0 | 1 | 28 | 145.9 |
| 1683 | 750 | 158 | 444 | 0 | 662 | 189 | 37.5 | 0 | 0 | 0 | 0 | 0 | 0.208 | 1 | 20 | 4 | 1 | 0 | 0 | 0 | 1 | 28 | 145.2 |
| 1684 | 750 | 158 | 444 | 0 | 662 | 189 | 37.5 | 0 | 0 | 0 | 0 | 0 | 0.208 | 1 | 20 | 4 | 1 | 0 | 0 | 0 | 1 | 28 | 140.7 |
| 1685 | 750 | 158 | 444 | 0 | 662 | 189 | 37.5 | 0 | 0 | 0 | 0 | 0 | 0.208 | 2 | 105 | 4 | 1 | 0 | 0 | 0 | 1 | 28 | 184.2 |
| 1686 | 750 | 158 | 444 | 0 | 662 | 189 | 37.5 | 0 | 0 | 0 | 0 | 0 | 0.208 | 2 | 105 | 4 | 1 | 0 | 0 | 0 | 1 | 28 | 160.7 |
| 1687 | 750 | 158 | 444 | 0 | 662 | 189 | 37.5 | 0 | 0 | 0 | 0 | 0 | 0.208 | 1 | 20 | 4 | 1 | 0 | 0 | 0 | 1 | 28 | 125 |
| 1688 | 750 | 158 | 444 | 0 | 662 | 189 | 37.5 | 0 | 0 | 0 | 0 | 0 | 0.208 | 1 | 20 | 4 | 1 | 0 | 0 | 0 | 1 | 28 | 135.4 |
| 1689 | 750 | 158 | 444 | 0 | 662 | 189 | 37.5 | 0 | 0 | 0 | 0 | 0 | 0.208 | 1 | 20 | 4 | 1 | 0 | 0 | 0 | 1 | 28 | 131.9 |
| 1690 | 750 | 158 | 444 | 0 | 662 | 189 | 37.5 | 0 | 0 | 0 | 0 | 0 | 0.208 | 1 | 20 | 4 | 1 | 0 | 0 | 0 | 1 | 28 | 145.9 |
| 1691 | 750 | 158 | 444 | 0 | 662 | 189 | 37.5 | 0 | 0 | 0 | 0 | 0 | 0.208 | 1 | 20 | 4 | 1 | 0 | 0 | 0 | 1 | 28 | 138.8 |
| 1692 | 750 | 158 | 444 | 0 | 662 | 189 | 37.5 | 0 | 0 | 0 | 0 | 0 | 0.208 | 1 | 20 | 4 | 1 | 0 | 0 | 0 | 1 | 28 | 148.2 |
| 1693 | 750 | 158 | 444 | 0 | 662 | 189 | 37.5 | 0 | 0 | 0 | 0 | 0 | 0.208 | 1 | 20 | 4 | 1 | 0 | 0 | 0 | 1 | 28 | 145.2 |
| 1694 | 750 | 158 | 444 | 0 | 662 | 189 | 37.5 | 0 | 0 | 0 | 0 | 0 | 0.208 | 1 | 20 | 4 | 1 | 0 | 0 | 0 | 1 | 28 | 157.8 |
| 1695 | 884.3 | 221.1 | 1105.4 | 0 | 0 | 148.2 | 27.6 | 150 | 0 | 0 | 0 | 0 | 0.134 | 2 | 80 | 1 | 1 | 1 | 13 | 0.23 | 1 | 7 | 236.8 |
| 1696 | 920 | 258 | 1030 | 0 | 0 | 235.6 | 30.6 | 0 | 0 | 0 | 0 | 0 | 0.200 | 2 | 90 | 1 | 1 | 2 | 0 | 0 | 1 | 7 | 118 |
| 1697 | 920 | 258 | 1030 | 0 | 0 | 235.6 | 30.6 | 276 | 0 | 0 | 0 | 0 | 0.200 | 2 | 90 | 1 | 1 | 2 | 18 | 0.15 | 1 | 7 | 113 |
| 1698 | 920 | 258 | 1030 | 0 | 0 | 235.6 | 30.6 | 92 | 0 | 0 | 0 | 0 | 0.200 | 2 | 90 | 1 | 1 | 2 | 6 | 0 | 1 | 7 | 120 |
| 1699 | 920 | 258 | 1030 | 0 | 0 | 235.6 | 30.6 | 184 | 0 | 0 | 0 | 0 | 0.200 | 2 | 90 | 1 | 1 | 2 | 6 | 0.15 | 1 | 7 | 115 |
| 1700 | 920 | 258 | 1030 | 0 | 0 | 235.6 | 30.6 | 276 | 0 | 0 | 0 | 0 | 0.200 | 2 | 90 | 1 | 1 | 2 | 6 | 0.15 | 1 | 7 | 117 |
| 1701 | 920 | 258 | 1030 | 0 | 0 | 235.6 | 30.6 | 92 | 0 | 0 | 0 | 0 | 0.200 | 2 | 90 | 1 | 1 | 2 | 12 | 0.15 | 1 | 7 | 125 |
| 1702 | 920 | 258 | 1030 | 0 | 0 | 235.6 | 30.6 | 184 | 0 | 0 | 0 | 0 | 0.200 | 2 | 90 | 1 | 1 | 2 | 12 | 0.15 | 1 | 7 | 116 |
| 1703 | 920 | 258 | 1030 | 0 | 0 | 235.6 | 30.6 | 276 | 0 | 0 | 0 | 0 | 0.200 | 2 | 90 | 1 | 1 | 2 | 12 | 0.15 | 1 | 7 | 119 |
| 1704 | 920 | 258 | 1030 | 0 | 0 | 235.6 | 30.6 | 92 | 0 | 0 | 0 | 0 | 0.200 | 2 | 90 | 1 | 1 | 2 | 18 | 0.15 | 1 | 7 | 134 |
| 1705 | 920 | 258 | 1030 | 0 | 0 | 235.6 | 30.6 | 184 | 0 | 0 | 0 | 0 | 0.200 | 2 | 90 | 1 | 1 | 2 | 18 | 0.15 | 1 | 7 | 126 |
| 1706 | 696 | 104 | 1479 | 0 | 0 | 160 | 6.4 | 0 | 0 | 0 | 0 | 0 | 0.200 | 1 | 23 | 7 | 3 | 0 | 0 | 0 | 1 | 7 | 62 |
| 1707 | 750 | 150 | 1315 | 0 | 0 | 180 | 7.2 | 0 | 0 | 0 | 0 | 0 | 0.200 | 1 | 23 | 7 | 3 | 0 | 0 | 0 | 1 | 28 | 140 |
| 1708 | 720 | 180 | 1298 | 0 | 0 | 180 | 7.2 | 0 | 0 | 0 | 0 | 0 | 0.200 | 1 | 23 | 7 | 3 | 0 | 0 | 0 | 1 | 7 | 60 |
| 1709 | 720 | 180 | 1298 | 0 | 0 | 180 | 7.2 | 0 | 0 | 0 | 0 | 0 | 0.200 | 1 | 23 | 7 | 3 | 0 | 0 | 0 | 1 | 28 | 120 |
| 1710 | 870 | 131 | 1185 | 0 | 0 | 200 | 8 | 0 | 0 | 0 | 0 | 0 | 0.200 | 1 | 23 | 7 | 3 | 0 | 0 | 0 | 1 | 7 | 75 |
| 1711 | 870 | 131 | 1185 | 0 | 0 | 200 | 8 | 0 | 0 | 0 | 0 | 0 | 0.200 | 1 | 23 | 7 | 3 | 0 | 0 | 0 | 1 | 28 | 142 |
| 1712 | 833 | 167 | 1166 | 0 | 0 | 200 | 8 | 0 | 0 | 0 | 0 | 0 | 0.200 | 1 | 23 | 7 | 3 | 0 | 0 | 0 | 1 | 7 | 73 |
| 1713 | 833 | 167 | 1166 | 0 | 0 | 200 | 8 | 0 | 0 | 0 | 0 | 0 | 0.200 | 1 | 23 | 7 | 3 | 0 | 0 | 0 | 1 | 28 | 139 |
| 1714 | 800 | 200 | 1148 | 0 | 0 | 200 | 8 | 0 | 0 | 0 | 0 | 0 | 0.200 | 1 | 23 | 7 | 3 | 0 | 0 | 0 | 1 | 7 | 68 |
| 1715 | 800 | 200 | 1148 | 0 | 0 | 200 | 8 | 0 | 0 | 0 | 0 | 0 | 0.200 | 1 | 23 | 7 | 3 | 0 | 0 | 0 | 1 | 28 | 125 |
| 1716 | 696 | 104 | 1522 | 0 | 0 | 144 | 6.4 | 0 | 0 | 0 | 0 | 0 | 0.180 | 1 | 23 | 7 | 3 | 0 | 0 | 0 | 1 | 7 | 70 |
| 1717 | 696 | 104 | 1479 | 0 | 0 | 160 | 6.4 | 0 | 0 | 0 | 0 | 0 | 0.200 | 1 | 23 | 7 | 3 | 0 | 0 | 0 | 1 | 28 | 126 |
| 1718 | 696 | 104 | 1522 | 0 | 0 | 144 | 6.4 | 0 | 0 | 0 | 0 | 0 | 0.180 | 1 | 23 | 7 | 3 | 0 | 0 | 0 | 1 | 28 | 133 |
| 1719 | 696 | 104 | 1437 | 0 | 0 | 176 | 6.4 | 0 | 0 | 0 | 0 | 0 | 0.220 | 1 | 23 | 7 | 3 | 0 | 0 | 0 | 1 | 7 | 60 |
| 1720 | 696 | 104 | 1437 | 0 | 0 | 176 | 6.4 | 0 | 0 | 0 | 0 | 0 | 0.220 | 1 | 23 | 7 | 3 | 0 | 0 | 0 | 1 | 28 | 115 |
| 1721 | 696 | 104 | 1481 | 0 | 0 | 160 | 5.6 | 0 | 0 | 0 | 0 | 0 | 0.200 | 1 | 23 | 7 | 3 | 0 | 0 | 0 | 1 | 7 | 62 |
| 1722 | 696 | 104 | 1481 | 0 | 0 | 160 | 5.6 | 0 | 0 | 0 | 0 | 0 | 0.200 | 1 | 23 | 7 | 3 | 0 | 0 | 0 | 1 | 28 | 125 |
| 1723 | 696 | 104 | 1477 | 0 | 0 | 160 | 7.2 | 0 | 0 | 0 | 0 | 0 | 0.200 | 1 | 23 | 7 | 3 | 0 | 0 | 0 | 1 | 7 | 63 |
| 1724 | 696 | 104 | 1477 | 0 | 0 | 160 | 7.2 | 0 | 0 | 0 | 0 | 0 | 0.200 | 1 | 23 | 7 | 3 | 0 | 0 | 0 | 1 | 28 | 128 |
| 1725 | 750 | 150 | 1362 | 0 | 0 | 162 | 7.2 | 0 | 0 | 0 | 0 | 0 | 0.180 | 1 | 23 | 7 | 3 | 0 | 0 | 0 | 1 | 7 | 80 |
| 1726 | 750 | 150 | 1362 | 0 | 0 | 162 | 7.2 | 0 | 0 | 0 | 0 | 0 | 0.180 | 1 | 23 | 7 | 3 | 0 | 0 | 0 | 1 | 28 | 142 |
| 1727 | 750 | 150 | 1267 | 0 | 0 | 198 | 7.2 | 0 | 0 | 0 | 0 | 0 | 0.220 | 1 | 23 | 7 | 3 | 0 | 0 | 0 | 1 | 7 | 75 |
| 1728 | 667 | 133 | 1463 | 0 | 0 | 160 | 6.4 | 0 | 0 | 0 | 0 | 0 | 0.200 | 1 | 23 | 7 | 3 | 0 | 0 | 0 | 1 | 7 | 55 |
| 1729 | 750 | 150 | 1267 | 0 | 0 | 198 | 7.2 | 0 | 0 | 0 | 0 | 0 | 0.220 | 1 | 23 | 7 | 3 | 0 | 0 | 0 | 1 | 28 | 128 |
| 1730 | 750 | 150 | 1317 | 0 | 0 | 180 | 6.3 | 0 | 0 | 0 | 0 | 0 | 0.200 | 1 | 23 | 7 | 3 | 0 | 0 | 0 | 1 | 7 | 76 |
| 1731 | 750 | 150 | 1317 | 0 | 0 | 180 | 6.3 | 0 | 0 | 0 | 0 | 0 | 0.200 | 1 | 23 | 7 | 3 | 0 | 0 | 0 | 1 | 28 | 134 |
| 1732 | 750 | 150 | 1312 | 0 | 0 | 180 | 8.1 | 0 | 0 | 0 | 0 | 0 | 0.200 | 1 | 23 | 7 | 3 | 0 | 0 | 0 | 1 | 7 | 80 |
| 1733 | 750 | 150 | 1312 | 0 | 0 | 180 | 8.1 | 0 | 0 | 0 | 0 | 0 | 0.200 | 1 | 23 | 7 | 3 | 0 | 0 | 0 | 1 | 28 | 135 |
| 1734 | 870 | 131 | 1238 | 0 | 0 | 180 | 8 | 0 | 0 | 0 | 0 | 0 | 0.180 | 1 | 23 | 7 | 3 | 0 | 0 | 0 | 1 | 7 | 80 |
| 1735 | 870 | 131 | 1238 | 0 | 0 | 180 | 8 | 0 | 0 | 0 | 0 | 0 | 0.180 | 1 | 23 | 7 | 3 | 0 | 0 | 0 | 1 | 28 | 148 |
| 1736 | 870 | 131 | 1132 | 0 | 0 | 220 | 8 | 0 | 0 | 0 | 0 | 0 | 0.220 | 1 | 23 | 7 | 3 | 0 | 0 | 0 | 1 | 7 | 73 |
| 1737 | 870 | 131 | 1132 | 0 | 0 | 220 | 8 | 0 | 0 | 0 | 0 | 0 | 0.220 | 1 | 23 | 7 | 3 | 0 | 0 | 0 | 1 | 28 | 141 |
| 1738 | 870 | 131 | 1188 | 0 | 0 | 200 | 7 | 0 | 0 | 0 | 0 | 0 | 0.200 | 1 | 23 | 7 | 3 | 0 | 0 | 0 | 1 | 7 | 84 |
| 1739 | 667 | 133 | 1463 | 0 | 0 | 160 | 6.4 | 0 | 0 | 0 | 0 | 0 | 0.200 | 1 | 23 | 7 | 3 | 0 | 0 | 0 | 1 | 28 | 120 |
| 1740 | 870 | 131 | 1188 | 0 | 0 | 200 | 7 | 0 | 0 | 0 | 0 | 0 | 0.200 | 1 | 23 | 7 | 3 | 0 | 0 | 0 | 1 | 28 | 140 |
| 1741 | 870 | 131 | 1183 | 0 | 0 | 200 | 9 | 0 | 0 | 0 | 0 | 0 | 0.200 | 1 | 23 | 7 | 3 | 0 | 0 | 0 | 1 | 7 | 72 |
| 1742 | 870 | 131 | 1183 | 0 | 0 | 200 | 9 | 0 | 0 | 0 | 0 | 0 | 0.200 | 1 | 23 | 7 | 3 | 0 | 0 | 0 | 1 | 28 | 145 |
| 1743 | 640 | 160 | 1448 | 0 | 0 | 160 | 6.4 | 0 | 0 | 0 | 0 | 0 | 0.200 | 1 | 23 | 7 | 3 | 0 | 0 | 0 | 1 | 7 | 53 |
| 1744 | 640 | 160 | 1448 | 0 | 0 | 160 | 6.4 | 0 | 0 | 0 | 0 | 0 | 0.200 | 1 | 23 | 7 | 3 | 0 | 0 | 0 | 1 | 28 | 119 |
| 1745 | 783 | 117 | 1333 | 0 | 0 | 180 | 7.2 | 0 | 0 | 0 | 0 | 0 | 0.200 | 1 | 23 | 7 | 3 | 0 | 0 | 0 | 1 | 7 | 70 |
| 1746 | 783 | 117 | 1333 | 0 | 0 | 180 | 7.2 | 0 | 0 | 0 | 0 | 0 | 0.200 | 1 | 23 | 7 | 3 | 0 | 0 | 0 | 1 | 28 | 135 |
| 1747 | 750 | 150 | 1315 | 0 | 0 | 180 | 7.2 | 0 | 0 | 0 | 0 | 0 | 0.200 | 1 | 23 | 7 | 3 | 0 | 0 | 0 | 1 | 7 | 73 |
| 1748 | 940 | 0 | 924 | 0 | 0 | 263 | 23.5 | 190 | 0 | 0 | 0 | 0 | 0.280 | 1 | 23 | 2 | 1 | 1 | 13 | 0.25 | 2 | 7 | 47.9 |
| 1749 | 890 | 222 | 799 | 0 | 0 | 222 | 29.6 | 119 | 0 | 0 | 0 | 0 | 0.200 | 2 | 250 | 2 | 1 | 1 | 13 | 0.25 | 1 | 7 | 146.1 |
| 1750 | 890 | 222 | 799 | 0 | 0 | 222 | 29.6 | 119 | 0 | 0 | 0 | 0 | 0.200 | 2 | 250 | 2 | 1 | 1 | 13 | 0.25 | 1 | 28 | 165.6 |
| 1751 | 710 | 230 | 844 | 0 | 0 | 263 | 14.8 | 190 | 0 | 0 | 0 | 0 | 0.280 | 1 | 23 | 2 | 1 | 1 | 13 | 0.25 | 2 | 7 | 47.5 |
| 1752 | 852 | 276 | 535 | 0 | 0 | 315 | 16 | 189 | 0 | 0 | 0 | 0 | 0.279 | 1 | 23 | 2 | 1 | 1 | 13 | 0.25 | 2 | 7 | 48.8 |
| 1753 | 852 | 276 | 535 | 0 | 0 | 315 | 16 | 189 | 0 | 0 | 0 | 0 | 0.279 | 1 | 23 | 2 | 1 | 1 | 13 | 0.25 | 2 | 28 | 63.5 |
| 1754 | 852 | 276 | 535 | 0 | 0 | 315 | 16 | 189 | 0 | 0 | 0 | 0 | 0.279 | 2 | 50 | 2 | 1 | 1 | 13 | 0.25 | 2 | 7 | 58.1 |
| 1755 | 852 | 276 | 535 | 0 | 0 | 315 | 16 | 189 | 0 | 0 | 0 | 0 | 0.279 | 2 | 50 | 2 | 1 | 1 | 13 | 0.25 | 2 | 28 | 66.6 |
| 1756 | 890 | 222 | 793 | 0 | 0 | 245 | 24.7 | 0 | 0 | 0 | 0 | 0 | 0.220 | 2 | 50 | 2 | 1 | 0 | 0 | 0 | 1 | 7 | 97.1 |
| 1757 | 890 | 222 | 793 | 0 | 0 | 245 | 24.7 | 0 | 0 | 0 | 0 | 0 | 0.220 | 2 | 50 | 2 | 1 | 0 | 0 | 0 | 1 | 28 | 112.1 |
| 1758 | 890 | 222 | 793 | 0 | 0 | 245 | 24.7 | 0 | 0 | 0 | 0 | 0 | 0.220 | 2 | 50 | 2 | 1 | 0 | 0 | 0 | 1 | 7 | 93.5 |
| 1759 | 890 | 222 | 793 | 0 | 0 | 245 | 24.7 | 0 | 0 | 0 | 0 | 0 | 0.220 | 2 | 50 | 2 | 1 | 0 | 0 | 0 | 1 | 28 | 104.8 |
| 1760 | 1067 | 267 | 498 | 0 | 0 | 294 | 17.3 | 0 | 0 | 0 | 0 | 0 | 0.220 | 2 | 250 | 2 | 1 | 0 | 0 | 0 | 1 | 7 | 97.1 |
| 1761 | 1067 | 267 | 498 | 0 | 0 | 294 | 17.3 | 0 | 0 | 0 | 0 | 0 | 0.220 | 2 | 250 | 2 | 1 | 0 | 0 | 0 | 1 | 28 | 118 |
| 1762 | 890 | 222 | 837 | 0 | 0 | 222 | 29.6 | 0 | 0 | 0 | 0 | 0 | 0.200 | 1 | 23 | 2 | 1 | 0 | 0 | 0 | 1 | 7 | 89.9 |
| 1763 | 890 | 222 | 837 | 0 | 0 | 222 | 29.6 | 0 | 0 | 0 | 0 | 0 | 0.200 | 1 | 23 | 2 | 1 | 0 | 0 | 0 | 1 | 28 | 122.4 |
| 1764 | 890 | 222 | 837 | 0 | 0 | 222 | 29.6 | 0 | 0 | 0 | 0 | 0 | 0.200 | 2 | 250 | 2 | 1 | 0 | 0 | 0 | 1 | 7 | 138.6 |
| 1765 | 890 | 222 | 837 | 0 | 0 | 222 | 29.6 | 0 | 0 | 0 | 0 | 0 | 0.200 | 2 | 250 | 2 | 1 | 0 | 0 | 0 | 1 | 28 | 161.9 |

| Appendix A-2: Dataset_Clean.csv | | | | | | | | | | | | | |
| --- | --- | --- | --- | --- | --- | --- | --- | --- | --- | --- | --- | --- | --- |
| N | C | SF | S | QP | W | SP | F | WB | FT | FL | FD | A | CS |
| 1 | 960 | 240 | 754 | 0 | 234 | 45 | 14.4 | 0.2 | 1 | 6 | 0.16 | 56 | 199.4 |
| 2 | 828 | 207 | 911 | 248 | 186.4 | 21 | 24.84 | 0.18 | 1 | 12 | 0.2 | 28 | 198.9 |
| 3 | 1116.7 | 390.85 | 625.35 | 0 | 212.17 | 23.45 | 33.501 | 0.14 | 1 | 13 | 0.2 | 42 | 198.7 |
| 4 | 960 | 240 | 767.2 | 0 | 234 | 45 | 9.6 | 0.2 | 1 | 6 | 0.16 | 56 | 197.2 |
| 5 | 960 | 240 | 773.8 | 0 | 234 | 45 | 7.2 | 0.2 | 1 | 6 | 0.16 | 90 | 197.2 |
| 6 | 1076.77 | 286.23 | 1090.8 | 0 | 272.6 | 46.2 | 32.3031 | 0.2 | 1 | 12.7 | 0.2 | 28 | 197 |
| 7 | 1116.7 | 390.85 | 625.35 | 0 | 212.17 | 23.45 | 55.835 | 0.14 | 1 | 13 | 0.2 | 28 | 196.8 |
| 8 | 1116.7 | 390.85 | 625.35 | 0 | 212.17 | 23.45 | 22.334 | 0.14 | 1 | 13 | 0.2 | 42 | 196.4 |
| 9 | 960 | 240 | 780.5 | 0 | 234 | 45 | 4.8 | 0.2 | 1 | 6 | 0.16 | 90 | 195.4 |
| 10 | 853 | 170.6 | 1023.6 | 255.9 | 196.19 | 29.86 | 51.18 | 0.19 | 1 | 16 | 0.2 | 56 | 194.7 |
| 11 | 853 | 170.6 | 1023.6 | 255.9 | 196.19 | 29.86 | 51.18 | 0.19 | 1 | 12 | 0.2 | 56 | 193.7 |
| 12 | 960 | 240 | 773.8 | 0 | 234 | 45 | 7.2 | 0.2 | 1 | 6 | 0.16 | 56 | 193.6 |
| 13 | 960 | 240 | 787.1 | 0 | 234 | 45 | 2.4 | 0.2 | 1 | 6 | 0.16 | 90 | 193.6 |
| 14 | 784 | 261 | 1045 | 0 | 171 | 20.9 | 23.52 | 0.16 | 1 | 13 | 0.2 | 90 | 192.7 |
| 15 | 960 | 240 | 780.5 | 0 | 234 | 45 | 4.8 | 0.2 | 1 | 6 | 0.16 | 56 | 191.8 |
| 16 | 853 | 170.6 | 1023.6 | 255.9 | 196.19 | 29.86 | 51.18 | 0.19 | 1 | 8 | 0.2 | 56 | 190.5 |
| 17 | 1116.7 | 390.85 | 625.35 | 0 | 212.17 | 23.45 | 44.668 | 0.14 | 1 | 13 | 0.2 | 28 | 189.4 |
| 18 | 960 | 240 | 787.1 | 0 | 234 | 45 | 2.4 | 0.2 | 1 | 6 | 0.16 | 56 | 188.6 |
| 19 | 960 | 240 | 740.7 | 0 | 234 | 45 | 19.2 | 0.2 | 1 | 6 | 0.16 | 28 | 188.3 |
| 20 | 792 | 264 | 1056 | 0 | 173 | 21.1 | 15.84 | 0.16 | 1 | 13 | 0.2 | 90 | 187 |
| 21 | 960 | 240 | 754 | 0 | 234 | 45 | 14.4 | 0.2 | 1 | 6 | 0.16 | 28 | 185.4 |
| 22 | 853 | 170.6 | 1023.6 | 255.9 | 196.19 | 29.86 | 25.59 | 0.19 | 1 | 12 | 0.2 | 56 | 185.3 |
| 23 | 853 | 170.6 | 1023.6 | 255.9 | 196.19 | 29.86 | 25.59 | 0.19 | 1 | 16 | 0.2 | 56 | 184.2 |
| 24 | 788 | 197 | 866.8 | 315 | 173 | 14.77 | 19.7 | 0.18 | 1 | 13 | 0.16 | 28 | 184 |
| 25 | 853 | 170.6 | 1023.6 | 255.9 | 196.19 | 29.86 | 25.59 | 0.19 | 1 | 8 | 0.2 | 56 | 183.2 |
| 26 | 784 | 261 | 1045 | 0 | 171 | 20.9 | 23.52 | 0.16 | 1 | 13 | 0.2 | 28 | 182.9 |
| 27 | 788 | 197 | 866.8 | 315 | 173 | 14.77 | 19.7 | 0.18 | 1 | 6 | 0.16 | 28 | 182.3 |
| 28 | 853 | 170.6 | 1023.6 | 255.9 | 196.19 | 29.86 | 51.18 | 0.19 | 1 | 12 | 0.2 | 28 | 182.1 |
| 29 | 853 | 170.6 | 1023.6 | 255.9 | 196.19 | 29.86 | 51.18 | 0.19 | 1 | 8 | 0.2 | 28 | 180 |
| 30 | 788 | 197 | 866.8 | 315 | 173 | 14.77 | 19.7 | 0.18 | 1 | 13 | 0.16 | 21 | 180 |
| 31 | 853 | 170.6 | 1023.6 | 255.9 | 196.19 | 29.86 | 51.18 | 0.19 | 1 | 16 | 0.2 | 28 | 178.9 |
| 32 | 960 | 240 | 767.2 | 0 | 234 | 45 | 9.6 | 0.2 | 1 | 6 | 0.16 | 28 | 178.9 |
| 33 | 863 | 216 | 1079 | 0 | 194.22 | 43.16 | 25.89 | 0.18 | 1 | 13 | 0.2 | 28 | 176.4 |
| 34 | 960 | 240 | 773.8 | 0 | 234 | 45 | 7.2 | 0.2 | 1 | 6 | 0.16 | 28 | 176.1 |
| 35 | 853 | 170.6 | 1023.6 | 255.9 | 196.19 | 29.86 | 8.53 | 0.19 | 1 | 16 | 0.2 | 56 | 175.8 |
| 36 | 1116.7 | 390.85 | 625.35 | 0 | 212.17 | 23.45 | 33.501 | 0.14 | 1 | 13 | 0.2 | 28 | 175.6 |
| 37 | 788 | 197 | 866.8 | 315 | 173 | 14.77 | 19.7 | 0.18 | 1 | 6 | 0.16 | 21 | 175.5 |
| 38 | 853 | 170.6 | 1023.6 | 255.9 | 196.19 | 29.86 | 25.59 | 0.19 | 1 | 12 | 0.2 | 28 | 174.7 |
| 39 | 1076.77 | 286.23 | 1090.8 | 0 | 272.6 | 46.2 | 32.3031 | 0.2 | 1 | 12.7 | 0.2 | 28 | 174.5 |
| 40 | 1116.7 | 390.85 | 625.35 | 0 | 212.17 | 23.45 | 33.501 | 0.14 | 1 | 13 | 0.2 | 28 | 174.4 |
| 41 | 788 | 197 | 866.8 | 315 | 173 | 14.77 | 15.76 | 0.18 | 1 | 13 | 0.16 | 28 | 173.8 |
| 42 | 853 | 170.6 | 1023.6 | 255.9 | 196.19 | 29.86 | 25.59 | 0.19 | 1 | 16 | 0.2 | 28 | 173.7 |
| 43 | 853 | 170.6 | 1023.6 | 255.9 | 196.19 | 29.86 | 8.53 | 0.19 | 1 | 8 | 0.2 | 56 | 173.7 |
| 44 | 853 | 170.6 | 1023.6 | 255.9 | 196.19 | 29.86 | 8.53 | 0.19 | 1 | 12 | 0.2 | 56 | 173.7 |
| 45 | 960 | 240 | 780.5 | 0 | 234 | 45 | 4.8 | 0.2 | 1 | 6 | 0.16 | 28 | 173.6 |
| 46 | 1116.7 | 390.85 | 625.35 | 0 | 212.17 | 23.45 | 44.668 | 0.14 | 1 | 13 | 0.2 | 28 | 173.3 |
| 47 | 792 | 264 | 1056 | 0 | 173 | 21.1 | 15.84 | 0.16 | 1 | 13 | 0.2 | 28 | 173.1 |
| 48 | 853 | 170.6 | 1023.6 | 255.9 | 196.19 | 29.86 | 25.59 | 0.19 | 1 | 8 | 0.2 | 28 | 172.6 |
| 49 | 1076.77 | 286.23 | 1090.8 | 0 | 272.6 | 46.2 | 32.3031 | 0.2 | 1 | 12.7 | 0.2 | 28 | 172.5 |
| 50 | 800 | 267 | 1067 | 0 | 175 | 21.3 | 8 | 0.16 | 1 | 13 | 0.2 | 90 | 172.3 |
| 51 | 960 | 240 | 787.1 | 0 | 234 | 45 | 2.4 | 0.2 | 1 | 6 | 0.16 | 28 | 172.1 |
| 52 | 788 | 197 | 866.8 | 315 | 173 | 14.77 | 19.7 | 0.18 | 1 | 13 | 0.16 | 14 | 171.9 |
| 53 | 853 | 170.6 | 1023.6 | 255.9 | 196.19 | 29.86 | 51.18 | 0.19 | 1 | 16 | 0.2 | 14 | 171.6 |
| 54 | 788 | 197 | 866.8 | 315 | 173 | 14.77 | 15.76 | 0.18 | 1 | 13 | 0.16 | 21 | 170.7 |
| 55 | 788 | 197 | 866.8 | 315 | 173 | 14.77 | 19.7 | 0.18 | 1 | 6 | 0.16 | 14 | 170.6 |
| 56 | 1116.7 | 390.85 | 625.35 | 0 | 212.17 | 23.45 | 22.334 | 0.14 | 1 | 13 | 0.2 | 28 | 169.8 |
| 57 | 850 | 212.5 | 935 | 255 | 212.5 | 21.2 | 25.5 | 0.2 | 1 | 12 | 0.2 | 28 | 169.1 |
| 58 | 1116.7 | 390.85 | 625.35 | 0 | 212.17 | 23.45 | 55.835 | 0.14 | 1 | 13 | 0.2 | 21 | 168.5 |
| 59 | 853 | 170.6 | 1023.6 | 255.9 | 196.19 | 29.86 | 51.18 | 0.19 | 1 | 8 | 0.2 | 14 | 168.4 |
| 60 | 1076.77 | 286.23 | 1090.8 | 0 | 272.6 | 46.2 | 32.3031 | 0.2 | 1 | 12.7 | 0.2 | 28 | 168.1 |
| 61 | 1116.7 | 390.85 | 625.35 | 0 | 212.17 | 23.45 | 44.668 | 0.14 | 1 | 13 | 0.2 | 28 | 167.5 |
| 62 | 853 | 170.6 | 1023.6 | 255.9 | 196.19 | 29.86 | 51.18 | 0.19 | 1 | 12 | 0.2 | 14 | 167.4 |
| 63 | 853 | 170.6 | 1023.6 | 255.9 | 196.19 | 29.86 | 8.53 | 0.19 | 1 | 16 | 0.2 | 28 | 167.4 |
| 64 | 788 | 197 | 866.8 | 315 | 173 | 14.77 | 15.76 | 0.18 | 1 | 6 | 0.16 | 28 | 167.3 |
| 65 | 853 | 170.6 | 1023.6 | 255.9 | 196.19 | 29.86 | 8.53 | 0.19 | 1 | 12 | 0.2 | 28 | 166.3 |
| 66 | 788 | 197 | 866.8 | 315 | 173 | 14.77 | 15.76 | 0.18 | 1 | 13 | 0.16 | 14 | 165.6 |
| 67 | 1116.7 | 390.85 | 625.35 | 0 | 212.17 | 23.45 | 44.668 | 0.14 | 1 | 13 | 0.2 | 21 | 165 |
| 68 | 863 | 216 | 1079 | 0 | 194.22 | 43.16 | 17.26 | 0.18 | 1 | 13 | 0.2 | 28 | 164.6 |
| 69 | 853 | 170.6 | 1023.6 | 255.9 | 196.19 | 29.86 | 8.53 | 0.19 | 1 | 8 | 0.2 | 28 | 164.2 |
| 70 | 936 | 140.4 | 1170 | 0 | 224.64 | 28.08 | 18.72 | 0.21 | 1 | 13 | 0.2 | 28 | 163.8 |
| 71 | 1116.7 | 390.85 | 625.35 | 0 | 212.17 | 23.45 | 22.334 | 0.14 | 1 | 13 | 0.2 | 28 | 163.8 |
| 72 | 853 | 170.6 | 1023.6 | 255.9 | 196.19 | 29.86 | 25.59 | 0.19 | 1 | 12 | 0.2 | 14 | 163.2 |
| 73 | 784 | 261 | 1045 | 0 | 171 | 20.9 | 23.52 | 0.16 | 1 | 13 | 0.2 | 90 | 162.6 |
| 74 | 853 | 170.6 | 1023.6 | 255.9 | 196.19 | 29.86 | 25.59 | 0.19 | 1 | 8 | 0.2 | 14 | 162.1 |
| 75 | 1116.7 | 390.85 | 625.35 | 0 | 212.17 | 23.45 | 22.334 | 0.14 | 1 | 13 | 0.2 | 21 | 160.3 |
| 76 | 853 | 170.6 | 1023.6 | 255.9 | 196.19 | 29.86 | 25.59 | 0.19 | 1 | 16 | 0.2 | 14 | 160 |
| 77 | 788 | 197 | 866.8 | 315 | 173 | 14.77 | 15.76 | 0.18 | 1 | 6 | 0.16 | 21 | 159.2 |
| 78 | 850 | 212.5 | 935 | 255 | 212.5 | 21.2 | 25.5 | 0.2 | 1 | 12 | 0.2 | 28 | 159.1 |
| 79 | 1076.77 | 286.23 | 1090.8 | 0 | 272.6 | 46.2 | 32.3031 | 0.2 | 1 | 12.7 | 0.2 | 28 | 158.2 |
| 80 | 960 | 240 | 740.7 | 0 | 234 | 45 | 19.2 | 0.2 | 1 | 6 | 0.16 | 7 | 157.8 |
| 81 | 853 | 170.6 | 1023.6 | 255.9 | 196.19 | 29.86 | 51.18 | 0.19 | 1 | 16 | 0.2 | 7 | 156.8 |
| 82 | 828 | 207 | 911 | 248 | 186.4 | 21 | 24.84 | 0.18 | 1 | 12 | 0.2 | 7 | 156.8 |
| 83 | 792 | 264 | 1056 | 0 | 173 | 21.1 | 15.84 | 0.16 | 1 | 13 | 0.2 | 91 | 156.8 |
| 84 | 853 | 170.6 | 1023.6 | 255.9 | 196.19 | 29.86 | 8.53 | 0.19 | 1 | 12 | 0.2 | 14 | 156.8 |
| 85 | 792 | 264 | 1056 | 0 | 173 | 21.1 | 15.84 | 0.16 | 1 | 13 | 0.2 | 91 | 156.5 |
| 86 | 792 | 264 | 1056 | 0 | 173 | 21.1 | 15.84 | 0.16 | 1 | 13 | 0.2 | 90 | 156.3 |
| 87 | 853 | 170.6 | 1023.6 | 255.9 | 196.19 | 29.86 | 51.18 | 0.19 | 1 | 8 | 0.2 | 7 | 155.8 |
| 88 | 788 | 197 | 866.8 | 315 | 173 | 14.77 | 19.7 | 0.18 | 1 | 13 | 0.16 | 7 | 155.8 |
| 89 | 853 | 170.6 | 1023.6 | 255.9 | 196.19 | 29.86 | 8.53 | 0.19 | 1 | 16 | 0.2 | 14 | 155.8 |
| 90 | 741 | 185 | 815 | 259 | 185 | 9 | 14.82 | 0.2 | 1 | 13 | 0.2 | 28 | 154.9 |
| 91 | 853 | 170.6 | 1023.6 | 255.9 | 196.19 | 29.86 | 51.18 | 0.19 | 1 | 12 | 0.2 | 7 | 154.7 |
| 92 | 853 | 170.6 | 1023.6 | 255.9 | 196.19 | 29.86 | 8.53 | 0.19 | 1 | 8 | 0.2 | 14 | 154.7 |
| 93 | 900 | 108 | 1000 | 0 | 161.28 | 18 | 11.25 | 0.16 | 1 | 35 | 0.603 | 28 | 154.1 |
| 94 | 1116.7 | 390.85 | 625.35 | 0 | 212.17 | 23.45 | 44.668 | 0.14 | 1 | 13 | 0.2 | 7 | 153.2 |
| 95 | 788 | 197 | 866.8 | 315 | 173 | 14.77 | 15.76 | 0.18 | 1 | 6 | 0.16 | 14 | 152.8 |
| 96 | 788 | 197 | 866.8 | 315 | 173 | 14.77 | 19.7 | 0.18 | 1 | 6 | 0.16 | 7 | 152.6 |
| 97 | 784 | 261 | 1045 | 0 | 171 | 20.9 | 23.52 | 0.16 | 1 | 13 | 0.2 | 28 | 152 |
| 98 | 853 | 170.6 | 1023.6 | 255.9 | 196.19 | 29.86 | 25.59 | 0.19 | 1 | 16 | 0.2 | 7 | 150.5 |
| 99 | 741 | 185 | 815 | 259 | 185 | 9 | 14.82 | 0.2 | 1 | 13 | 0.2 | 91 | 150.4 |
| 100 | 960 | 240 | 960 | 0 | 207 | 24 | 76.8 | 0.17 | 1 | 13 | 0.18 | 28 | 150.2 |
| 101 | 788 | 197 | 866.8 | 315 | 173 | 14.77 | 15.76 | 0.18 | 1 | 13 | 0.16 | 7 | 150.2 |
| 102 | 800 | 267 | 1067 | 0 | 175 | 21.3 | 8 | 0.16 | 1 | 13 | 0.2 | 28 | 150.2 |
| 103 | 853 | 170.6 | 1023.6 | 255.9 | 196.19 | 29.86 | 25.59 | 0.19 | 1 | 8 | 0.2 | 7 | 149.5 |
| 104 | 960 | 240 | 754 | 0 | 234 | 45 | 14.4 | 0.2 | 1 | 6 | 0.16 | 7 | 149.2 |
| 105 | 850 | 212.5 | 935 | 255 | 212.5 | 21.2 | 25.5 | 0.2 | 1 | 12 | 0.2 | 28 | 148.7 |
| 106 | 853 | 170.6 | 1023.6 | 255.9 | 196.19 | 29.86 | 25.59 | 0.19 | 1 | 12 | 0.2 | 7 | 148.4 |
| 107 | 1116.7 | 390.85 | 625.35 | 0 | 212.17 | 23.45 | 22.334 | 0.14 | 1 | 13 | 0.2 | 28 | 148.1 |
| 108 | 784 | 261 | 1045 | 0 | 171 | 20.9 | 23.52 | 0.16 | 1 | 13 | 0.2 | 7 | 147.8 |
| 109 | 960 | 240 | 767.2 | 0 | 234 | 45 | 9.6 | 0.2 | 1 | 6 | 0.16 | 7 | 146.7 |
| 110 | 792 | 264 | 1056 | 0 | 173 | 21.1 | 15.84 | 0.16 | 1 | 13 | 0.2 | 28 | 145.7 |
| 111 | 1116.7 | 390.85 | 625.35 | 0 | 212.17 | 23.45 | 55.835 | 0.14 | 1 | 13 | 0.2 | 7 | 145 |
| 112 | 800 | 267 | 1067 | 0 | 175 | 21.3 | 8 | 0.16 | 1 | 13 | 0.2 | 90 | 144.8 |
| 113 | 863 | 216 | 1079 | 0 | 194.22 | 43.16 | 8.63 | 0.18 | 1 | 13 | 0.2 | 28 | 144.7 |
| 114 | 792 | 264 | 1056 | 0 | 173 | 21.1 | 15.84 | 0.16 | 1 | 13 | 0.2 | 28 | 144.4 |
| 115 | 853 | 170.6 | 1023.6 | 255.9 | 196.19 | 29.86 | 8.53 | 0.19 | 1 | 16 | 0.2 | 7 | 144.2 |
| 116 | 792 | 264 | 1056 | 0 | 173 | 21.1 | 15.84 | 0.16 | 1 | 13 | 0.2 | 28 | 143.6 |
| 117 | 960 | 240 | 773.8 | 0 | 234 | 45 | 7.2 | 0.2 | 1 | 6 | 0.16 | 7 | 143.5 |
| 118 | 792 | 264 | 1056 | 0 | 173 | 21.1 | 15.84 | 0.16 | 1 | 13 | 0.2 | 7 | 142.8 |
| 119 | 1116.7 | 390.85 | 625.35 | 0 | 212.17 | 23.45 | 33.501 | 0.14 | 1 | 13 | 0.2 | 21 | 142.6 |
| 120 | 853 | 170.6 | 1023.6 | 255.9 | 196.19 | 29.86 | 8.53 | 0.19 | 1 | 8 | 0.2 | 7 | 142.1 |
| 121 | 853 | 170.6 | 1023.6 | 255.9 | 196.19 | 29.86 | 8.53 | 0.19 | 1 | 12 | 0.2 | 7 | 142.1 |
| 122 | 1116.7 | 390.85 | 625.35 | 0 | 212.17 | 23.45 | 33.501 | 0.14 | 1 | 13 | 0.2 | 7 | 141.4 |
| 123 | 1116.7 | 390.85 | 625.35 | 0 | 212.17 | 23.45 | 22.334 | 0.14 | 1 | 13 | 0.2 | 28 | 141.1 |
| 124 | 960 | 240 | 780.5 | 0 | 234 | 45 | 4.8 | 0.2 | 1 | 6 | 0.16 | 7 | 140.6 |
| 125 | 1116.7 | 390.85 | 625.35 | 0 | 212.17 | 23.45 | 22.334 | 0.14 | 1 | 13 | 0.2 | 28 | 140.5 |
| 126 | 1000 | 250 | 1000 | 0 | 200 | 50 | 15 | 0.16 | 1 | 12 | 0.2 | 28 | 140 |
| 127 | 960 | 240 | 787.1 | 0 | 234 | 45 | 2.4 | 0.2 | 1 | 6 | 0.16 | 7 | 137.7 |
| 128 | 828 | 207 | 911 | 248 | 186.4 | 21 | 24.84 | 0.18 | 1 | 12 | 0.2 | 3 | 136.6 |
| 129 | 960 | 240 | 960 | 0 | 260 | 24 | 76.8 | 0.22 | 1 | 13 | 0.18 | 28 | 136.4 |
| 130 | 960 | 240 | 960 | 0 | 228 | 24 | 76.8 | 0.19 | 1 | 13 | 0.18 | 28 | 136.1 |
| 131 | 950 | 255 | 873 | 0 | 189 | 31 | 23.75 | 0.16 | 1 | 13 | 0.2 | 90 | 135.6 |
| 132 | 1116.7 | 390.85 | 625.35 | 0 | 212.17 | 23.45 | 22.334 | 0.14 | 1 | 13 | 0.2 | 7 | 135.5 |
| 133 | 1076.77 | 286.23 | 1090.8 | 0 | 272.6 | 46.2 | 32.3031 | 0.2 | 1 | 12.7 | 0.2 | 28 | 135.1 |
| 134 | 960 | 240 | 960 | 0 | 286 | 24 | 76.8 | 0.24 | 1 | 13 | 0.18 | 28 | 132.6 |
| 135 | 788 | 197 | 866.8 | 315 | 173 | 14.77 | 15.76 | 0.18 | 1 | 6 | 0.16 | 7 | 132.4 |
| 136 | 950 | 255 | 873 | 0 | 189 | 31 | 23.75 | 0.16 | 1 | 13 | 0.2 | 28 | 130.8 |
| 137 | 1116.7 | 390.85 | 625.35 | 0 | 212.17 | 23.45 | 44.668 | 0.14 | 1 | 13 | 0.2 | 28 | 129.6 |
| 138 | 1000 | 250 | 1000 | 0 | 200 | 50 | 10 | 0.16 | 1 | 12 | 0.2 | 28 | 129.5 |
| 139 | 800 | 267 | 1067 | 0 | 175 | 21.3 | 8 | 0.16 | 1 | 13 | 0.2 | 7 | 128.1 |
| 140 | 800 | 267 | 1067 | 0 | 175 | 21.3 | 8 | 0.16 | 1 | 13 | 0.2 | 28 | 126.6 |
| 141 | 784 | 261 | 1045 | 0 | 171 | 20.9 | 23.52 | 0.16 | 1 | 13 | 0.2 | 7 | 123.2 |
| 142 | 784 | 261 | 1045 | 0 | 171 | 20.9 | 23.52 | 0.16 | 1 | 13 | 0.2 | 3 | 122.4 |
| 143 | 741 | 185 | 815 | 259 | 185 | 9 | 14.82 | 0.2 | 1 | 13 | 0.2 | 7 | 121.3 |
| 144 | 960 | 240 | 698.3 | 0 | 234 | 78 | 14.4 | 0.2 | 5 | 13 | 0.018 | 90 | 194.3 |
| 145 | 960 | 240 | 649.3 | 0 | 234 | 96 | 19.2 | 0.2 | 5 | 13 | 0.018 | 90 | 193.4 |
| 146 | 960 | 240 | 798.8 | 0 | 234 | 39 | 9.6 | 0.2 | 5 | 13 | 0.018 | 90 | 191.1 |
| 147 | 960 | 240 | 793.9 | 0 | 234 | 42 | 7.2 | 0.2 | 5 | 13 | 0.018 | 90 | 188.9 |
| 148 | 960 | 240 | 788.9 | 0 | 234 | 45 | 4.8 | 0.2 | 5 | 13 | 0.018 | 90 | 188.3 |
| 149 | 960 | 240 | 698.3 | 0 | 234 | 78 | 14.4 | 0.2 | 5 | 13 | 0.018 | 56 | 188.1 |
| 150 | 960 | 240 | 649.3 | 0 | 234 | 96 | 19.2 | 0.2 | 5 | 13 | 0.018 | 56 | 186.6 |
| 151 | 960 | 240 | 791.3 | 0 | 234 | 45 | 2.4 | 0.2 | 5 | 13 | 0.018 | 90 | 185.8 |
| 152 | 998.8 | 176.25 | 954.5 | 0 | 141 | 81.7 | 14.982 | 0.12 | 5 | 13 | 0.018 | 28 | 184.4 |
| 153 | 960 | 240 | 798.8 | 0 | 234 | 39 | 9.6 | 0.2 | 5 | 13 | 0.018 | 56 | 183.7 |
| 154 | 998.8 | 176.25 | 920.8 | 0 | 141 | 84.6 | 24.97 | 0.12 | 5 | 13 | 0.018 | 28 | 183.5 |
| 155 | 998.8 | 176.25 | 942.7 | 0 | 141 | 82.8 | 19.976 | 0.12 | 5 | 13 | 0.018 | 28 | 183.5 |
| 156 | 998.8 | 176.25 | 898.7 | 0 | 141 | 88.2 | 29.964 | 0.12 | 5 | 13 | 0.018 | 28 | 183.4 |
| 157 | 960 | 240 | 793.9 | 0 | 234 | 42 | 7.2 | 0.2 | 5 | 13 | 0.018 | 56 | 183.4 |
| 158 | 998.8 | 176.25 | 975 | 0 | 141 | 78.7 | 9.988 | 0.12 | 5 | 13 | 0.018 | 28 | 181 |
| 159 | 960 | 240 | 788.9 | 0 | 234 | 45 | 4.8 | 0.2 | 5 | 13 | 0.018 | 56 | 180.6 |
| 160 | 998.8 | 176.25 | 930.6 | 0 | 164.5 | 55.2 | 24.97 | 0.14 | 5 | 13 | 0.018 | 28 | 180.1 |
| 161 | 998.8 | 176.25 | 946.8 | 0 | 164.5 | 54.1 | 19.976 | 0.14 | 5 | 13 | 0.018 | 28 | 180.1 |
| 162 | 998.8 | 176.25 | 962.9 | 0 | 164.5 | 52.9 | 14.982 | 0.14 | 5 | 13 | 0.018 | 28 | 180.1 |
| 163 | 998.8 | 176.25 | 900.1 | 0 | 164.5 | 62.3 | 29.964 | 0.14 | 5 | 13 | 0.018 | 28 | 179.9 |
| 164 | 998.8 | 176.25 | 981.9 | 0 | 164.5 | 50.5 | 9.988 | 0.14 | 5 | 13 | 0.018 | 28 | 178.6 |
| 165 | 960 | 240 | 791.3 | 0 | 234 | 45 | 2.4 | 0.2 | 5 | 13 | 0.018 | 56 | 178.4 |
| 166 | 960 | 240 | 698.3 | 0 | 234 | 78 | 14.4 | 0.2 | 5 | 13 | 0.018 | 28 | 177.3 |
| 167 | 960 | 240 | 649.3 | 0 | 234 | 96 | 19.2 | 0.2 | 5 | 13 | 0.018 | 28 | 174.3 |
| 168 | 960 | 240 | 798.8 | 0 | 234 | 39 | 9.6 | 0.2 | 5 | 13 | 0.018 | 28 | 172 |
| 169 | 960 | 240 | 793.9 | 0 | 234 | 42 | 7.2 | 0.2 | 5 | 13 | 0.018 | 28 | 171.1 |
| 170 | 998.8 | 176.25 | 991.1 | 0 | 141 | 77.6 | 4.994 | 0.12 | 5 | 13 | 0.018 | 28 | 169.1 |
| 171 | 960 | 240 | 788.9 | 0 | 234 | 45 | 4.8 | 0.2 | 5 | 13 | 0.018 | 28 | 165.8 |
| 172 | 998.8 | 176.25 | 998 | 0 | 164.5 | 49.4 | 4.994 | 0.14 | 5 | 13 | 0.018 | 28 | 164.5 |
| 173 | 998.8 | 176.25 | 954.5 | 0 | 141 | 81.7 | 14.982 | 0.12 | 5 | 13 | 0.018 | 14 | 164.3 |
| 174 | 998.8 | 176.25 | 920.8 | 0 | 141 | 84.6 | 24.97 | 0.12 | 5 | 13 | 0.018 | 14 | 164.2 |
| 175 | 998.8 | 176.25 | 942.7 | 0 | 141 | 82.8 | 19.976 | 0.12 | 5 | 13 | 0.018 | 14 | 164.2 |
| 176 | 998.8 | 176.25 | 898.7 | 0 | 141 | 88.2 | 29.964 | 0.12 | 5 | 13 | 0.018 | 14 | 163.8 |
| 177 | 998.8 | 176.25 | 975 | 0 | 141 | 78.7 | 9.988 | 0.12 | 5 | 13 | 0.018 | 14 | 162 |
| 178 | 960 | 240 | 791.3 | 0 | 234 | 45 | 2.4 | 0.2 | 5 | 13 | 0.018 | 28 | 161.1 |
| 179 | 998.8 | 176.25 | 962.9 | 0 | 164.5 | 52.9 | 14.982 | 0.14 | 5 | 13 | 0.018 | 14 | 158.7 |
| 180 | 998.8 | 176.25 | 946.8 | 0 | 164.5 | 54.1 | 19.976 | 0.14 | 5 | 13 | 0.018 | 14 | 158.4 |
| 181 | 998.8 | 176.25 | 900.1 | 0 | 164.5 | 62.3 | 29.964 | 0.14 | 5 | 13 | 0.018 | 14 | 158.2 |
| 182 | 998.8 | 176.25 | 930.6 | 0 | 164.5 | 55.2 | 24.97 | 0.14 | 5 | 13 | 0.018 | 14 | 158.2 |
| 183 | 998.8 | 176.25 | 991.1 | 0 | 141 | 77.6 | 4.994 | 0.12 | 5 | 13 | 0.018 | 14 | 157.5 |
| 184 | 998.8 | 176.25 | 981.9 | 0 | 164.5 | 50.5 | 9.988 | 0.14 | 5 | 13 | 0.018 | 14 | 152 |
| 185 | 998.8 | 176.25 | 954.5 | 0 | 141 | 81.7 | 14.982 | 0.12 | 5 | 13 | 0.018 | 7 | 151.7 |
| 186 | 998.8 | 176.25 | 898.7 | 0 | 141 | 88.2 | 29.964 | 0.12 | 5 | 13 | 0.018 | 7 | 151.3 |
| 187 | 998.8 | 176.25 | 920.8 | 0 | 141 | 84.6 | 24.97 | 0.12 | 5 | 13 | 0.018 | 7 | 151.3 |
| 188 | 998.8 | 176.25 | 942.7 | 0 | 141 | 82.8 | 19.976 | 0.12 | 5 | 13 | 0.018 | 7 | 151.1 |
| 189 | 960 | 240 | 698.3 | 0 | 234 | 78 | 14.4 | 0.2 | 5 | 13 | 0.018 | 7 | 149 |
| 190 | 998.8 | 176.25 | 998 | 0 | 164.5 | 49.4 | 4.994 | 0.14 | 5 | 13 | 0.018 | 14 | 147.5 |
| 191 | 998.8 | 176.25 | 975 | 0 | 141 | 78.7 | 9.988 | 0.12 | 5 | 13 | 0.018 | 7 | 146.1 |
| 192 | 960 | 240 | 649.3 | 0 | 234 | 96 | 19.2 | 0.2 | 5 | 13 | 0.018 | 7 | 146 |
| 193 | 960 | 240 | 798.8 | 0 | 234 | 39 | 9.6 | 0.2 | 5 | 13 | 0.018 | 7 | 143.7 |
| 194 | 998.8 | 176.25 | 962.9 | 0 | 164.5 | 52.9 | 14.982 | 0.14 | 5 | 13 | 0.018 | 7 | 143.5 |
| 195 | 998.8 | 176.25 | 930.6 | 0 | 164.5 | 55.2 | 24.97 | 0.14 | 5 | 13 | 0.018 | 7 | 143.2 |
| 196 | 998.8 | 176.25 | 946.8 | 0 | 164.5 | 54.1 | 19.976 | 0.14 | 5 | 13 | 0.018 | 7 | 143.2 |
| 197 | 998.8 | 176.25 | 900.1 | 0 | 164.5 | 62.3 | 29.964 | 0.14 | 5 | 13 | 0.018 | 7 | 143 |
| 198 | 998.8 | 176.25 | 991.1 | 0 | 141 | 77.6 | 4.994 | 0.12 | 5 | 13 | 0.018 | 7 | 142.4 |
| 199 | 960 | 240 | 793.9 | 0 | 234 | 42 | 7.2 | 0.2 | 5 | 13 | 0.018 | 7 | 140.6 |
| 200 | 998.8 | 176.25 | 981.9 | 0 | 164.5 | 50.5 | 9.988 | 0.14 | 5 | 13 | 0.018 | 7 | 137.6 |
| 201 | 960 | 240 | 788.9 | 0 | 234 | 45 | 4.8 | 0.2 | 5 | 13 | 0.018 | 7 | 137.4 |
| 202 | 960 | 240 | 791.3 | 0 | 234 | 45 | 2.4 | 0.2 | 5 | 13 | 0.018 | 7 | 134 |
| 203 | 998.8 | 176.25 | 998 | 0 | 164.5 | 49.4 | 4.994 | 0.14 | 5 | 13 | 0.018 | 7 | 131.6 |
| 204 | 828 | 207 | 911 | 248 | 186.4 | 21 | 24.84 | 0.18 | 4 | 10 | 0.44 | 28 | 175.3 |
| 205 | 922 | 230 | 1152 | 0 | 184 | 11.52 | 13.83 | 0.16 | 4 | 12 | 0.018 | 28 | 168.2 |
| 206 | 922 | 230 | 1152 | 0 | 184 | 11.52 | 9.22 | 0.16 | 4 | 12 | 0.018 | 28 | 167.5 |
| 207 | 828 | 207 | 911 | 248 | 186.4 | 21 | 16.56 | 0.18 | 4 | 10 | 0.44 | 28 | 165.6 |
| 208 | 922 | 230 | 1152 | 0 | 184 | 11.52 | 4.61 | 0.16 | 4 | 12 | 0.018 | 28 | 163.7 |
| 209 | 922 | 230 | 1152 | 0 | 184 | 11.52 | 16.135 | 0.16 | 4 | 12 | 0.018 | 28 | 163.4 |
| 210 | 1000 | 250 | 1000 | 0 | 200 | 50 | 10 | 0.16 | 4 | 12 | 0.018 | 28 | 159.6 |
| 211 | 922 | 230 | 1152 | 0 | 184 | 11.52 | 1.844 | 0.16 | 4 | 12 | 0.018 | 28 | 158.9 |
| 212 | 828 | 207 | 911 | 248 | 186.4 | 21 | 8.28 | 0.18 | 4 | 10 | 0.44 | 28 | 155.2 |
| 213 | 828 | 207 | 911 | 248 | 186.4 | 21 | 24.84 | 0.18 | 4 | 10 | 0.86 | 28 | 155.1 |
| 214 | 850 | 212.5 | 935 | 255 | 212.5 | 21.2 | 2.55 | 0.2 | 4 | 9 | 0.013 | 28 | 153.2 |
| 215 | 828 | 207 | 911 | 248 | 186.4 | 21 | 24.84 | 0.18 | 4 | 10 | 0.44 | 7 | 153 |
| 216 | 828 | 207 | 911 | 248 | 186.4 | 21 | 16.56 | 0.18 | 4 | 10 | 0.86 | 28 | 152.6 |
| 217 | 1000 | 250 | 1000 | 0 | 200 | 50 | 5 | 0.16 | 4 | 12 | 0.018 | 28 | 151.8 |
| 218 | 828 | 207 | 911 | 248 | 186.4 | 21 | 8.28 | 0.18 | 4 | 10 | 0.86 | 28 | 150.4 |
| 219 | 850 | 212.5 | 935 | 255 | 212.5 | 21.2 | 0.85 | 0.2 | 4 | 9 | 0.013 | 28 | 149.8 |
| 220 | 850 | 212.5 | 935 | 255 | 212.5 | 21.2 | 25.5 | 0.2 | 4 | 10 | 0.4 | 28 | 148.6 |
| 221 | 1000 | 250 | 1000 | 0 | 200 | 50 | 15 | 0.16 | 4 | 12 | 0.018 | 28 | 148.4 |
| 222 | 828 | 207 | 911 | 248 | 186.4 | 21 | 24.84 | 0.18 | 4 | 20 | 0.86 | 28 | 148.1 |
| 223 | 850 | 212.5 | 935 | 255 | 212.5 | 21.2 | 25.5 | 0.2 | 4 | 10 | 0.44 | 28 | 147.2 |
| 224 | 850 | 212.5 | 935 | 255 | 212.5 | 21.2 | 17 | 0.2 | 4 | 10 | 0.44 | 28 | 143 |
| 225 | 850 | 212.5 | 935 | 255 | 212.5 | 21.2 | 4.25 | 0.2 | 4 | 9 | 0.013 | 28 | 141.7 |
| 226 | 850 | 212.5 | 935 | 255 | 212.5 | 21.2 | 25.5 | 0.2 | 4 | 10 | 0.86 | 28 | 140.8 |
| 227 | 828 | 207 | 911 | 248 | 186.4 | 21 | 16.56 | 0.18 | 4 | 10 | 0.44 | 7 | 140.1 |
| 228 | 850 | 212.5 | 935 | 255 | 212.5 | 21.2 | 25.5 | 0.2 | 4 | 10 | 0.44 | 28 | 139.1 |
| 229 | 850 | 212.5 | 935 | 255 | 212.5 | 21.2 | 5.95 | 0.2 | 4 | 9 | 0.013 | 28 | 137.1 |
| 230 | 828 | 207 | 911 | 248 | 186.4 | 21 | 24.84 | 0.18 | 4 | 10 | 0.44 | 3 | 136 |
| 231 | 850 | 212.5 | 935 | 255 | 212.5 | 21.2 | 17 | 0.2 | 4 | 10 | 0.86 | 28 | 135.8 |
| 232 | 828 | 207 | 911 | 248 | 186.4 | 21 | 8.28 | 0.18 | 4 | 10 | 0.44 | 7 | 135.1 |
| 233 | 875 | 150 | 1100 | 0 | 180 | 35 | 8.75 | 0.18 | 4 | 24 | 0.023 | 28 | 135.1 |
| 234 | 875 | 150 | 1100 | 0 | 180 | 35 | 6.5625 | 0.18 | 4 | 24 | 0.023 | 28 | 134.9 |
| 235 | 850 | 212.5 | 935 | 255 | 212.5 | 21.2 | 17 | 0.2 | 4 | 10 | 0.44 | 28 | 132.1 |
| 236 | 850 | 212.5 | 935 | 255 | 212.5 | 21.2 | 25.5 | 0.2 | 4 | 10 | 0.86 | 28 | 131.2 |
| 237 | 850 | 212.5 | 935 | 255 | 212.5 | 21.2 | 25.5 | 0.2 | 4 | 10 | 0.44 | 28 | 131.1 |
| 238 | 875 | 150 | 1100 | 0 | 180 | 35 | 6.5625 | 0.18 | 4 | 24 | 0.023 | 28 | 130.8 |
| 239 | 875 | 150 | 1100 | 0 | 180 | 35 | 8.75 | 0.18 | 4 | 24 | 0.023 | 28 | 130.1 |
| 240 | 875 | 150 | 1100 | 0 | 180 | 35 | 6.5625 | 0.18 | 4 | 24 | 0.023 | 28 | 129.8 |
| 241 | 828 | 207 | 911 | 248 | 186.4 | 21 | 16.56 | 0.18 | 4 | 10 | 0.44 | 3 | 129 |
| 242 | 850 | 212.5 | 935 | 255 | 212.5 | 21.2 | 8.5 | 0.2 | 4 | 10 | 0.86 | 28 | 128 |
| 243 | 850 | 212.5 | 935 | 255 | 212.5 | 21.2 | 8.5 | 0.2 | 4 | 10 | 0.44 | 28 | 127.4 |
| 244 | 850 | 212.5 | 935 | 255 | 212.5 | 21.2 | 17 | 0.2 | 4 | 10 | 0.86 | 28 | 126.3 |
| 245 | 828 | 207 | 911 | 248 | 186.4 | 21 | 24.84 | 0.18 | 4 | 10 | 0.86 | 7 | 125.6 |
| 246 | 850 | 212.5 | 935 | 255 | 212.5 | 21.2 | 8.5 | 0.2 | 4 | 10 | 0.44 | 28 | 125.5 |
| 247 | 875 | 150 | 1100 | 0 | 180 | 35 | 8.75 | 0.18 | 4 | 24 | 0.023 | 28 | 125.4 |
| 248 | 922 | 230 | 1152 | 0 | 184 | 11.52 | 13.83 | 0.16 | 4 | 12 | 0.018 | 7 | 125.1 |
| 249 | 828 | 207 | 911 | 248 | 186.4 | 21 | 16.56 | 0.18 | 4 | 10 | 0.86 | 7 | 123.6 |
| 250 | 850 | 212.5 | 935 | 255 | 212.5 | 21.2 | 8.5 | 0.2 | 4 | 10 | 0.44 | 28 | 123 |
| 251 | 828 | 207 | 911 | 248 | 186.4 | 21 | 8.28 | 0.18 | 4 | 10 | 0.86 | 7 | 121.8 |
| 252 | 850 | 212.5 | 935 | 255 | 212.5 | 21.2 | 25.5 | 0.2 | 4 | 10 | 0.86 | 28 | 121.7 |
| 253 | 850 | 212.5 | 935 | 255 | 212.5 | 21.2 | 17 | 0.2 | 4 | 10 | 0.44 | 28 | 121.6 |
| 254 | 850 | 212.5 | 935 | 255 | 212.5 | 21.2 | 8.5 | 0.2 | 4 | 10 | 0.86 | 28 | 121.1 |
| 255 | 850 | 212.5 | 935 | 255 | 212.5 | 21.2 | 25.5 | 0.2 | 3 | 15 | 0.6 | 28 | 165 |
| 256 | 936 | 140.4 | 1170 | 0 | 224.64 | 28.08 | 11.232 | 0.21 | 3 | 12 | 0.038 | 28 | 160.5 |
| 257 | 936 | 140.4 | 1170 | 0 | 224.64 | 28.08 | 7.488 | 0.21 | 3 | 12 | 0.038 | 28 | 154.8 |
| 258 | 936 | 140.4 | 1170 | 0 | 224.64 | 28.08 | 3.744 | 0.21 | 3 | 12 | 0.038 | 28 | 150.3 |
| 259 | 900 | 108 | 1000 | 0 | 161.28 | 18 | 3.06 | 0.16 | 3 | 54 | 0.054 | 28 | 139.8 |
| 260 | 850 | 212.5 | 935 | 255 | 212.5 | 21.2 | 2.55 | 0.2 | 3 | 9 | 0.017 | 28 | 138.9 |
| 261 | 900 | 108 | 1000 | 0 | 161.28 | 18 | 4.68 | 0.16 | 3 | 48 | 0.75 | 28 | 138.8 |
| 262 | 900 | 108 | 1000 | 0 | 161.28 | 18 | 6.21 | 0.16 | 3 | 48 | 0.75 | 28 | 137.8 |
| 263 | 833 | 167 | 836 | 363 | 184 | 24 | 1.666 | 0.18 | 3 | 20 | 0.034 | 28 | 135.7 |
| 264 | 833 | 167 | 836 | 363 | 184 | 24 | 1.666 | 0.18 | 3 | 20 | 0.034 | 28 | 135.6 |
| 265 | 900 | 108 | 1000 | 0 | 161.28 | 18 | 4.68 | 0.16 | 3 | 54 | 0.054 | 28 | 134.7 |
| 266 | 900 | 108 | 1000 | 0 | 161.28 | 18 | 6.21 | 0.16 | 3 | 54 | 0.054 | 28 | 132.7 |
| 267 | 900 | 108 | 1000 | 0 | 161.28 | 18 | 3.06 | 0.16 | 3 | 48 | 0.75 | 28 | 125.5 |
| 268 | 920 | 258 | 1030 | 0 | 235.6 | 30.6 | 92 | 0.2 | 2 | 18 | 0.15 | 7 | 134 |
| 269 | 920 | 258 | 1030 | 0 | 235.6 | 30.6 | 184 | 0.2 | 2 | 18 | 0.15 | 7 | 126 |
| 270 | 920 | 258 | 1030 | 0 | 235.6 | 30.6 | 92 | 0.2 | 2 | 12 | 0.15 | 7 | 125 |
| 271 | 920 | 258 | 1030 | 0 | 235.6 | 30.6 | 92 | 0.2 | 2 | 6 | 0.15 | 7 | 120 |
| 272 | 850 | 260 | 850 | 212 | 170 | 45 | 156 | 0.15 | 1 | 13 | 0.2 | 28 | 200 |
| 273 | 850 | 260 | 850 | 212 | 170 | 45 | 156 | 0.15 | 1 | 13 | 0.2 | 28 | 199.9 |
| 274 | 788.5 | 433.7 | 867.4 | 0 | 160.3 | 52.6 | 78 | 0.13 | 1 | 13 | 0.2 | 28 | 199.6 |
| 275 | 788.5 | 433.7 | 867.4 | 0 | 160.3 | 52.6 | 39 | 0.13 | 1 | 13 | 0.2 | 28 | 198.6 |
| 276 | 850 | 260 | 850 | 212 | 170 | 45 | 156 | 0.15 | 1 | 13 | 0.2 | 28 | 198.5 |
| 277 | 850 | 260 | 850 | 212 | 170 | 45 | 156 | 0.15 | 1 | 13 | 0.2 | 28 | 197.9 |
| 278 | 850 | 260 | 850 | 212 | 170 | 45 | 156 | 0.15 | 1 | 13 | 0.2 | 28 | 197.6 |
| 279 | 850 | 260 | 850 | 212 | 170 | 45 | 156 | 0.15 | 1 | 13 | 0.2 | 28 | 196.9 |
| 280 | 850 | 260 | 850 | 212 | 170 | 45 | 156 | 0.15 | 1 | 13 | 0.2 | 28 | 196.9 |
| 281 | 788.5 | 433.7 | 867.4 | 0 | 160.3 | 52.6 | 0 | 0.13 | 1 | 13 | 0.2 | 28 | 195.2 |
| 282 | 850 | 260 | 850 | 212 | 170 | 45 | 156 | 0.15 | 1 | 13 | 0.2 | 28 | 195.2 |
| 283 | 850 | 260 | 850 | 212 | 170 | 45 | 156 | 0.15 | 1 | 13 | 0.2 | 28 | 194.9 |
| 284 | 850 | 260 | 850 | 212 | 170 | 45 | 156 | 0.15 | 1 | 13 | 0.2 | 28 | 193.5 |
| 285 | 788.5 | 197.1 | 1104 | 0 | 160.3 | 50 | 156 | 0.16 | 1 | 13 | 0.2 | 28 | 192.3 |
| 286 | 850 | 260 | 850 | 212 | 170 | 45 | 156 | 0.15 | 1 | 13 | 0.2 | 28 | 192.2 |
| 287 | 850 | 260 | 850 | 212 | 170 | 45 | 156 | 0.15 | 1 | 13 | 0.2 | 28 | 192.2 |
| 288 | 850 | 260 | 850 | 212 | 170 | 45 | 156 | 0.15 | 1 | 13 | 0.2 | 28 | 191.2 |
| 289 | 850 | 260 | 850 | 212 | 170 | 45 | 156 | 0.15 | 1 | 13 | 0.2 | 28 | 190.2 |
| 290 | 850 | 260 | 850 | 212 | 170 | 45 | 156 | 0.15 | 1 | 13 | 0.2 | 28 | 188.2 |
| 291 | 850 | 260 | 850 | 212 | 170 | 45 | 156 | 0.15 | 1 | 13 | 0.2 | 28 | 187.5 |
| 292 | 788.5 | 197.1 | 1104 | 0 | 160.3 | 50 | 156 | 0.16 | 1 | 13 | 0.2 | 28 | 187.1 |
| 293 | 850 | 260 | 850 | 212 | 170 | 45 | 156 | 0.15 | 1 | 13 | 0.2 | 28 | 186.8 |
| 294 | 788.5 | 433.7 | 867.4 | 0 | 160.3 | 52.6 | 78 | 0.13 | 1 | 13 | 0.2 | 28 | 186.7 |
| 295 | 850 | 260 | 850 | 212 | 170 | 45 | 156 | 0.15 | 1 | 13 | 0.2 | 28 | 186.1 |
| 296 | 850 | 260 | 850 | 212 | 170 | 45 | 156 | 0.15 | 1 | 13 | 0.2 | 28 | 185.2 |
| 297 | 850 | 260 | 850 | 212 | 170 | 45 | 156 | 0.15 | 1 | 13 | 0.2 | 28 | 184.4 |
| 298 | 850 | 260 | 850 | 212 | 170 | 45 | 156 | 0.15 | 1 | 13 | 0.2 | 28 | 184 |
| 299 | 960 | 240 | 740.7 | 0 | 234 | 45 | 156 | 0.2 | 1 | 13 | 0.2 | 180 | 182.4 |
| 300 | 850 | 260 | 850 | 212 | 170 | 45 | 156 | 0.15 | 1 | 13 | 0.2 | 28 | 182.4 |
| 301 | 850 | 260 | 850 | 212 | 170 | 45 | 156 | 0.15 | 1 | 13 | 0.2 | 28 | 182.4 |
| 302 | 850 | 260 | 850 | 212 | 170 | 45 | 156 | 0.15 | 1 | 13 | 0.2 | 28 | 181.7 |
| 303 | 850 | 260 | 850 | 212 | 170 | 45 | 156 | 0.15 | 1 | 13 | 0.2 | 28 | 181 |
| 304 | 850 | 260 | 850 | 212 | 170 | 45 | 156 | 0.15 | 1 | 13 | 0.2 | 28 | 181 |
| 305 | 850 | 260 | 850 | 212 | 170 | 45 | 156 | 0.15 | 1 | 13 | 0.2 | 28 | 180.8 |
| 306 | 960 | 240 | 740.7 | 0 | 234 | 45 | 156 | 0.2 | 1 | 13 | 0.2 | 90 | 180.3 |
| 307 | 850 | 260 | 850 | 212 | 170 | 45 | 156 | 0.15 | 1 | 13 | 0.2 | 28 | 180.3 |
| 308 | 1151.11 | 211.89 | 1231 | 0 | 272.6 | 46.2 | 234 | 0.2 | 1 | 13 | 0.2 | 28 | 179 |
| 309 | 960 | 240 | 754 | 0 | 234 | 45 | 117 | 0.2 | 1 | 13 | 0.2 | 180 | 178.1 |
| 310 | 960 | 240 | 706.2 | 0 | 234 | 57 | 156 | 0.2 | 1 | 13 | 0.2 | 180 | 177.2 |
| 311 | 1151.11 | 211.89 | 1231 | 0 | 272.6 | 46.2 | 234 | 0.2 | 1 | 13 | 0.2 | 28 | 177 |
| 312 | 960 | 240 | 754 | 0 | 234 | 45 | 117 | 0.2 | 1 | 13 | 0.2 | 90 | 176.8 |
| 313 | 850 | 260 | 850 | 212 | 170 | 45 | 156 | 0.15 | 1 | 13 | 0.2 | 28 | 176.6 |
| 314 | 960 | 240 | 767.2 | 0 | 234 | 45 | 78 | 0.2 | 1 | 13 | 0.2 | 180 | 175.2 |
| 315 | 850 | 260 | 850 | 212 | 170 | 45 | 156 | 0.15 | 1 | 13 | 0.2 | 28 | 174.6 |
| 316 | 960 | 240 | 720.7 | 0 | 234 | 57 | 117 | 0.2 | 1 | 13 | 0.2 | 180 | 174.2 |
| 317 | 960 | 240 | 767.2 | 0 | 234 | 45 | 78 | 0.2 | 1 | 13 | 0.2 | 90 | 173.6 |
| 318 | 960 | 240 | 740.7 | 0 | 234 | 45 | 156 | 0.2 | 1 | 13 | 0.2 | 56 | 173.2 |
| 319 | 850 | 260 | 850 | 212 | 170 | 45 | 156 | 0.15 | 1 | 13 | 0.2 | 28 | 173.2 |
| 320 | 850 | 260 | 850 | 212 | 170 | 45 | 156 | 0.15 | 1 | 13 | 0.2 | 28 | 172.9 |
| 321 | 960 | 240 | 754 | 0 | 234 | 45 | 117 | 0.2 | 1 | 13 | 0.2 | 56 | 172.7 |
| 322 | 960 | 240 | 706.2 | 0 | 234 | 57 | 156 | 0.2 | 1 | 13 | 0.2 | 90 | 172.5 |
| 323 | 960 | 240 | 764.7 | 0 | 234 | 45 | 78 | 0.2 | 1 | 13 | 0.2 | 180 | 172.5 |
| 324 | 960 | 240 | 773.8 | 0 | 234 | 45 | 58.5 | 0.2 | 1 | 13 | 0.2 | 180 | 172.3 |
| 325 | 850 | 260 | 850 | 212 | 170 | 45 | 156 | 0.15 | 1 | 13 | 0.2 | 28 | 171.2 |
| 326 | 960 | 240 | 720.7 | 0 | 234 | 57 | 117 | 0.2 | 1 | 13 | 0.2 | 90 | 170.9 |
| 327 | 960 | 240 | 780.5 | 0 | 234 | 45 | 39 | 0.2 | 1 | 13 | 0.2 | 180 | 170.9 |
| 328 | 960 | 240 | 767.2 | 0 | 234 | 45 | 78 | 0.2 | 1 | 13 | 0.2 | 56 | 170.7 |
| 329 | 1151.11 | 211.89 | 1231 | 0 | 272.6 | 46.2 | 234 | 0.2 | 1 | 13 | 0.2 | 28 | 170.6 |
| 330 | 960 | 240 | 773.8 | 0 | 234 | 45 | 58.5 | 0.2 | 1 | 13 | 0.2 | 90 | 170.5 |
| 331 | 850 | 260 | 850 | 212 | 170 | 45 | 156 | 0.15 | 1 | 13 | 0.2 | 28 | 170.4 |
| 332 | 960 | 240 | 771.9 | 0 | 234 | 45 | 58.5 | 0.2 | 1 | 13 | 0.2 | 180 | 170.3 |
| 333 | 960 | 240 | 787.1 | 0 | 234 | 45 | 19.5 | 0.2 | 1 | 13 | 0.2 | 180 | 169.8 |
| 334 | 960 | 240 | 780.5 | 0 | 234 | 45 | 39 | 0.2 | 1 | 13 | 0.2 | 90 | 169.4 |
| 335 | 960 | 240 | 764.7 | 0 | 234 | 45 | 78 | 0.2 | 1 | 13 | 0.2 | 90 | 169.1 |
| 336 | 960 | 240 | 779.2 | 0 | 234 | 45 | 39 | 0.2 | 1 | 13 | 0.2 | 180 | 169.1 |
| 337 | 950 | 234 | 1030 | 95 | 215 | 12.7 | 234 | 0.18 | 1 | 13 | 0.2 | 28 | 168.6 |
| 338 | 960 | 240 | 771.9 | 0 | 234 | 45 | 58.5 | 0.2 | 1 | 13 | 0.2 | 90 | 167.9 |
| 339 | 960 | 240 | 786.4 | 0 | 234 | 45 | 19.5 | 0.2 | 1 | 13 | 0.2 | 180 | 167.9 |
| 340 | 960 | 240 | 787.1 | 0 | 234 | 45 | 19.5 | 0.2 | 1 | 13 | 0.2 | 90 | 167.8 |
| 341 | 960 | 240 | 779.2 | 0 | 234 | 45 | 39 | 0.2 | 1 | 13 | 0.2 | 90 | 167.7 |
| 342 | 960 | 240 | 773.8 | 0 | 234 | 45 | 58.5 | 0.2 | 1 | 13 | 0.2 | 56 | 167.6 |
| 343 | 960 | 240 | 706.2 | 0 | 234 | 57 | 156 | 0.2 | 1 | 13 | 0.2 | 56 | 166.7 |
| 344 | 784 | 261 | 1045 | 0 | 171 | 20.9 | 234 | 0.16 | 1 | 13 | 0.2 | 90 | 166.51 |
| 345 | 960 | 240 | 780.5 | 0 | 234 | 45 | 39 | 0.2 | 1 | 13 | 0.2 | 56 | 166.2 |
| 346 | 729 | 124 | 833 | 397 | 170 | 30 | 156 | 0.2 | 1 | 13 | 0.2 | 28 | 166.1 |
| 347 | 808 | 202 | 1091 | 0 | 202 | 24 | 234 | 0.2 | 1 | 13 | 0.2 | 28 | 165.7 |
| 348 | 890 | 222 | 799 | 0 | 222 | 29.6 | 119 | 0.2 | 1 | 13 | 0.25 | 28 | 165.6 |
| 349 | 960 | 240 | 720.7 | 0 | 234 | 57 | 117 | 0.2 | 1 | 13 | 0.2 | 56 | 165.5 |
| 350 | 960 | 240 | 786.4 | 0 | 234 | 45 | 19.5 | 0.2 | 1 | 13 | 0.2 | 90 | 165.1 |
| 351 | 741 | 185 | 815 | 259 | 185 | 9 | 156 | 0.2 | 1 | 13 | 0.2 | 90 | 165 |
| 352 | 960 | 240 | 764.7 | 0 | 234 | 45 | 78 | 0.2 | 1 | 13 | 0.2 | 56 | 164.9 |
| 353 | 960 | 240 | 771.9 | 0 | 234 | 45 | 58.5 | 0.2 | 1 | 13 | 0.2 | 56 | 164.7 |
| 354 | 960 | 240 | 779.2 | 0 | 234 | 45 | 39 | 0.2 | 1 | 13 | 0.2 | 56 | 163.3 |
| 355 | 960 | 240 | 787.1 | 0 | 234 | 45 | 19.5 | 0.2 | 1 | 13 | 0.2 | 56 | 163.1 |
| 356 | 960 | 240 | 740.7 | 0 | 234 | 45 | 156 | 0.2 | 1 | 13 | 0.2 | 28 | 162.8 |
| 357 | 701.5 | 210.5 | 820.8 | 259.6 | 164.2 | 17.5 | 156 | 0.18 | 1 | 13 | 0.2 | 28 | 161.9 |
| 358 | 900 | 220 | 1005 | 0 | 163 | 40 | 156 | 0.15 | 1 | 13 | 0.2 | 56 | 161.6 |
| 359 | 792 | 264 | 1056 | 0 | 173 | 21.1 | 156 | 0.16 | 1 | 13 | 0.2 | 90 | 161.47 |
| 360 | 960 | 240 | 786.4 | 0 | 234 | 45 | 19.5 | 0.2 | 1 | 13 | 0.2 | 56 | 160.9 |
| 361 | 960 | 240 | 754 | 0 | 234 | 45 | 117 | 0.2 | 1 | 13 | 0.2 | 28 | 160.6 |
| 362 | 960 | 240 | 706.2 | 0 | 234 | 57 | 156 | 0.2 | 1 | 13 | 0.2 | 28 | 160.1 |
| 363 | 784 | 261 | 1045 | 0 | 171 | 20.9 | 234 | 0.16 | 1 | 13 | 0.2 | 28 | 158.32 |
| 364 | 950 | 234 | 1030 | 95 | 215 | 12.7 | 234 | 0.18 | 1 | 13 | 0.2 | 28 | 158.1 |
| 365 | 960 | 240 | 720.7 | 0 | 234 | 57 | 117 | 0.2 | 1 | 13 | 0.2 | 28 | 156.2 |
| 366 | 874.9 | 43.7 | 1273.4 | 0 | 202.1 | 45.9 | 195 | 0.22 | 1 | 13 | 0.2 | 28 | 155.71 |
| 367 | 960 | 240 | 767.2 | 0 | 234 | 45 | 78 | 0.2 | 1 | 13 | 0.2 | 28 | 154.8 |
| 368 | 960 | 240 | 764.7 | 0 | 234 | 45 | 78 | 0.2 | 1 | 13 | 0.2 | 28 | 154.8 |
| 369 | 960 | 240 | 773.8 | 0 | 234 | 45 | 58.5 | 0.2 | 1 | 13 | 0.2 | 28 | 152.5 |
| 370 | 960 | 240 | 771.9 | 0 | 234 | 45 | 58.5 | 0.2 | 1 | 13 | 0.2 | 28 | 151.8 |
| 371 | 792 | 264 | 1056 | 0 | 173 | 21.1 | 156 | 0.16 | 1 | 13 | 0.2 | 28 | 150.13 |
| 372 | 960 | 240 | 780.5 | 0 | 234 | 45 | 39 | 0.2 | 1 | 13 | 0.2 | 28 | 150.1 |
| 373 | 800 | 267 | 1067 | 0 | 175 | 21.3 | 78 | 0.16 | 1 | 13 | 0.2 | 90 | 148.87 |
| 374 | 960 | 240 | 787.1 | 0 | 234 | 45 | 19.5 | 0.2 | 1 | 13 | 0.2 | 28 | 148.7 |
| 375 | 960 | 240 | 779.2 | 0 | 234 | 45 | 39 | 0.2 | 1 | 13 | 0.2 | 28 | 148.6 |
| 376 | 699.9 | 43.7 | 1273.4 | 175 | 202.1 | 45.9 | 195 | 0.27 | 1 | 13 | 0.2 | 28 | 148.32 |
| 377 | 729 | 182 | 1150 | 0 | 182 | 27 | 156 | 0.2 | 1 | 13 | 0.2 | 28 | 148 |
| 378 | 817 | 204 | 1102 | 0 | 204 | 25 | 156 | 0.2 | 1 | 13 | 0.2 | 28 | 147.5 |
| 379 | 874.9 | 43.7 | 1273.4 | 0 | 202.1 | 45.9 | 156 | 0.22 | 1 | 13 | 0.2 | 28 | 146.31 |
| 380 | 890 | 222 | 799 | 0 | 222 | 29.6 | 119 | 0.2 | 1 | 13 | 0.25 | 7 | 146.1 |
| 381 | 825 | 206 | 1114 | 0 | 206 | 25 | 78 | 0.2 | 1 | 13 | 0.2 | 28 | 145.5 |
| 382 | 950 | 200 | 690 | 350 | 230 | 53 | 234 | 0.2 | 1 | 13 | 0.2 | 28 | 144.7 |
| 383 | 960 | 240 | 786.4 | 0 | 234 | 45 | 19.5 | 0.2 | 1 | 13 | 0.2 | 28 | 144.5 |
| 384 | 950 | 200 | 810 | 350 | 230 | 53 | 234 | 0.2 | 1 | 13 | 0.2 | 28 | 144.5 |
| 385 | 741 | 185 | 815 | 259 | 185 | 9 | 156 | 0.2 | 1 | 13 | 0.2 | 7 | 144 |
| 386 | 900 | 220 | 1005 | 0 | 163 | 40 | 156 | 0.15 | 1 | 13 | 0.2 | 28 | 143 |
| 387 | 863 | 216 | 923 | 0 | 177 | 32.37 | 156 | 0.16 | 1 | 13 | 0.2 | 28 | 142.1 |
| 388 | 950 | 200 | 570 | 350 | 230 | 53 | 234 | 0.2 | 1 | 13 | 0.2 | 28 | 140.7 |
| 389 | 699.9 | 43.7 | 1273.4 | 175 | 202.1 | 45.9 | 156 | 0.27 | 1 | 13 | 0.2 | 28 | 139.6 |
| 390 | 960 | 240 | 740.7 | 0 | 234 | 45 | 156 | 0.2 | 1 | 13 | 0.2 | 7 | 136.6 |
| 391 | 900 | 220 | 1005 | 0 | 163 | 40 | 156 | 0.15 | 1 | 13 | 0.2 | 14 | 132.9 |
| 392 | 960 | 240 | 706.2 | 0 | 234 | 57 | 156 | 0.2 | 1 | 13 | 0.2 | 7 | 132.4 |
| 393 | 874.9 | 43.7 | 1273.4 | 0 | 202.1 | 45.9 | 117 | 0.22 | 1 | 13 | 0.2 | 28 | 132.22 |
| 394 | 890 | 222 | 799 | 0 | 222 | 29.7 | 117 | 0.2 | 1 | 13 | 0.2 | 28 | 130.3 |
| 395 | 699.9 | 43.7 | 1273.4 | 175 | 202.1 | 45.9 | 117 | 0.27 | 1 | 13 | 0.2 | 28 | 130.2 |
| 396 | 960 | 240 | 720.7 | 0 | 234 | 57 | 117 | 0.2 | 1 | 13 | 0.2 | 7 | 130 |
| 397 | 800 | 267 | 1067 | 0 | 175 | 21.3 | 78 | 0.16 | 1 | 13 | 0.2 | 28 | 129.96 |
| 398 | 960 | 240 | 754 | 0 | 234 | 45 | 117 | 0.2 | 1 | 13 | 0.2 | 7 | 129.2 |
| 399 | 784 | 261 | 1045 | 0 | 171 | 20.9 | 234 | 0.16 | 1 | 13 | 0.2 | 7 | 128.07 |
| 400 | 960 | 240 | 764.7 | 0 | 234 | 45 | 78 | 0.2 | 1 | 13 | 0.2 | 7 | 127.4 |
| 401 | 960 | 240 | 767.2 | 0 | 234 | 45 | 78 | 0.2 | 1 | 13 | 0.2 | 7 | 126.9 |
| 402 | 874.9 | 43.7 | 1273.4 | 0 | 202.1 | 45.9 | 78 | 0.22 | 1 | 13 | 0.2 | 28 | 124.83 |
| 403 | 960 | 240 | 773.8 | 0 | 234 | 45 | 58.5 | 0.2 | 1 | 13 | 0.2 | 7 | 124.2 |
| 404 | 900 | 220 | 1005 | 0 | 163 | 40 | 156 | 0.15 | 1 | 13 | 0.2 | 7 | 124.1 |
| 405 | 792 | 264 | 1056 | 0 | 173 | 21.1 | 156 | 0.16 | 1 | 13 | 0.2 | 7 | 123.66 |
| 406 | 699.9 | 43.7 | 1273.4 | 175 | 202.1 | 45.9 | 195 | 0.27 | 1 | 13 | 0.2 | 7 | 122.82 |
| 407 | 960 | 240 | 771.9 | 0 | 234 | 45 | 58.5 | 0.2 | 1 | 13 | 0.2 | 7 | 122.6 |
| 408 | 960 | 240 | 780.5 | 0 | 234 | 45 | 39 | 0.2 | 1 | 13 | 0.2 | 7 | 121.8 |
| 409 | 874.9 | 43.7 | 1273.4 | 0 | 202.1 | 45.9 | 156 | 0.22 | 1 | 13 | 0.2 | 7 | 120.81 |
| 410 | 699.9 | 43.7 | 1273.4 | 175 | 202.1 | 45.9 | 78 | 0.27 | 1 | 13 | 0.2 | 28 | 120.81 |
| 411 | 960 | 240 | 779.2 | 0 | 234 | 45 | 39 | 0.2 | 1 | 13 | 0.2 | 7 | 120.2 |
| 412 | 1076.77 | 286.23 | 1090.8 | 0 | 272.6 | 46.2 | 0 | 0.2 | 0 | 0 | 0 | 28 | 191.5 |
| 413 | 1116.7 | 390.85 | 625.35 | 0 | 212.17 | 23.45 | 0 | 0.14 | 0 | 0 | 0 | 42 | 189.4 |
| 414 | 1076.77 | 286.23 | 1090.8 | 0 | 272.6 | 46.2 | 0 | 0.2 | 0 | 0 | 0 | 28 | 189.3 |
| 415 | 1076.77 | 286.23 | 1090.8 | 0 | 272.6 | 46.2 | 0 | 0.2 | 0 | 0 | 0 | 28 | 184.5 |
| 416 | 750 | 158 | 444 | 662 | 189 | 37.5 | 0 | 0.21 | 0 | 0 | 0 | 28 | 184.2 |
| 417 | 958.55 | 50.45 | 1456.8 | 0 | 201.8 | 34.2 | 0 | 0.2 | 0 | 0 | 0 | 90 | 180.8 |
| 418 | 960 | 240 | 793.7 | 0 | 234 | 45 | 0 | 0.2 | 0 | 0 | 0 | 90 | 180.4 |
| 419 | 958.55 | 50.45 | 1456.8 | 0 | 201.8 | 34.2 | 0 | 0.2 | 0 | 0 | 0 | 90 | 180.1 |
| 420 | 810 | 90 | 1539 | 0 | 162 | 18 | 0 | 0.18 | 0 | 0 | 0 | 365 | 177 |
| 421 | 810 | 90 | 1539 | 0 | 162 | 18 | 0 | 0.18 | 0 | 0 | 0 | 365 | 177 |
| 422 | 958.55 | 50.45 | 1456.8 | 0 | 201.8 | 34.2 | 0 | 0.2 | 0 | 0 | 0 | 90 | 176.9 |
| 423 | 800 | 200 | 980 | 100 | 200 | 9.6 | 0 | 0.2 | 0 | 0 | 0 | 90 | 175.9 |
| 424 | 1009 | 0 | 1456.8 | 0 | 201.8 | 34.2 | 0 | 0.2 | 0 | 0 | 0 | 90 | 175.3 |
| 425 | 810 | 90 | 1539 | 0 | 162 | 18 | 0 | 0.18 | 0 | 0 | 0 | 180 | 174 |
| 426 | 810 | 90 | 1539 | 0 | 162 | 18 | 0 | 0.18 | 0 | 0 | 0 | 180 | 174 |
| 427 | 960 | 240 | 793.7 | 0 | 234 | 45 | 0 | 0.2 | 0 | 0 | 0 | 56 | 173.6 |
| 428 | 1009 | 0 | 1456.8 | 0 | 201.8 | 34.2 | 0 | 0.2 | 0 | 0 | 0 | 90 | 172 |
| 429 | 1009 | 0 | 1456.8 | 0 | 201.8 | 34.2 | 0 | 0.2 | 0 | 0 | 0 | 90 | 171.4 |
| 430 | 1116.7 | 390.85 | 625.35 | 0 | 212.17 | 23.45 | 0 | 0.14 | 0 | 0 | 0 | 21 | 170.9 |
| 431 | 1116.7 | 390.85 | 625.35 | 0 | 212.17 | 23.45 | 0 | 0.14 | 0 | 0 | 0 | 28 | 170.9 |
| 432 | 955 | 239 | 1051 | 0 | 143 | 15 | 0 | 0.12 | 0 | 0 | 0 | 28 | 170 |
| 433 | 810 | 90 | 1539 | 0 | 162 | 18 | 0 | 0.18 | 0 | 0 | 0 | 90 | 170 |
| 434 | 810 | 90 | 1539 | 0 | 162 | 18 | 0 | 0.18 | 0 | 0 | 0 | 90 | 170 |
| 435 | 630 | 70 | 1788 | 0 | 126 | 18 | 0 | 0.18 | 0 | 0 | 0 | 365 | 169 |
| 436 | 853 | 170.6 | 1023.6 | 255.9 | 196.19 | 29.86 | 0 | 0.19 | 0 | 0 | 0 | 56 | 168.4 |
| 437 | 998.8 | 176.25 | 1010.1 | 0 | 141 | 75.2 | 0 | 0.12 | 0 | 0 | 0 | 28 | 167.6 |
| 438 | 630 | 70 | 1788 | 0 | 126 | 18 | 0 | 0.18 | 0 | 0 | 0 | 180 | 167 |
| 439 | 800 | 100 | 980 | 200 | 200 | 9.6 | 0 | 0.22 | 0 | 0 | 0 | 90 | 166.4 |
| 440 | 1151.11 | 211.89 | 1231 | 0 | 272.6 | 46.2 | 0 | 0.2 | 0 | 0 | 0 | 28 | 165.8 |
| 441 | 1076.77 | 286.23 | 1090.8 | 0 | 272.6 | 46.2 | 0 | 0.2 | 0 | 0 | 0 | 28 | 165.6 |
| 442 | 1076.77 | 286.23 | 1090.8 | 0 | 272.6 | 46.2 | 0 | 0.2 | 0 | 0 | 0 | 28 | 164.6 |
| 443 | 1151.11 | 211.89 | 1231 | 0 | 272.6 | 46.2 | 0 | 0.2 | 0 | 0 | 0 | 28 | 163.9 |
| 444 | 800 | 200 | 880 | 200 | 200 | 9.6 | 0 | 0.2 | 0 | 0 | 0 | 90 | 163.3 |
| 445 | 1009 | 0 | 1456.8 | 0 | 201.8 | 34.2 | 0 | 0.2 | 0 | 0 | 0 | 90 | 163.1 |
| 446 | 998.8 | 176.25 | 1013.9 | 0 | 164.5 | 49.4 | 0 | 0.14 | 0 | 0 | 0 | 28 | 162.2 |
| 447 | 890 | 222 | 837 | 0 | 222 | 29.6 | 0 | 0.2 | 0 | 0 | 0 | 28 | 161.9 |
| 448 | 960 | 240 | 793.7 | 0 | 234 | 45 | 0 | 0.2 | 0 | 0 | 0 | 180 | 161.3 |
| 449 | 450 | 50 | 1992 | 0 | 90 | 18 | 0 | 0.18 | 0 | 0 | 0 | 365 | 161 |
| 450 | 750 | 158 | 444 | 662 | 189 | 37.5 | 0 | 0.21 | 0 | 0 | 0 | 28 | 160.7 |
| 451 | 1076.77 | 286.23 | 1090.8 | 0 | 272.6 | 46.2 | 0 | 0.2 | 0 | 0 | 0 | 28 | 160.6 |
| 452 | 1116.7 | 390.85 | 625.35 | 0 | 212.17 | 23.45 | 0 | 0.14 | 0 | 0 | 0 | 28 | 160.5 |
| 453 | 630 | 70 | 1788 | 0 | 126 | 18 | 0 | 0.18 | 0 | 0 | 0 | 90 | 160 |
| 454 | 1009 | 0 | 1456.8 | 0 | 201.8 | 34.2 | 0 | 0.2 | 0 | 0 | 0 | 90 | 160 |
| 455 | 1151.11 | 211.89 | 1231 | 0 | 272.6 | 46.2 | 0 | 0.2 | 0 | 0 | 0 | 28 | 159.7 |
| 456 | 1009 | 0 | 1456.8 | 0 | 201.8 | 34.2 | 0 | 0.2 | 0 | 0 | 0 | 90 | 159.6 |
| 457 | 800 | 200 | 1080 | 0 | 200 | 14.4 | 0 | 0.2 | 0 | 0 | 0 | 28 | 159.2 |
| 458 | 450 | 50 | 1992 | 0 | 90 | 18 | 0 | 0.18 | 0 | 0 | 0 | 180 | 159 |
| 459 | 853 | 170.6 | 1023.6 | 255.9 | 196.19 | 29.86 | 0 | 0.19 | 0 | 0 | 0 | 28 | 158.9 |
| 460 | 750 | 158 | 444 | 662 | 189 | 37.5 | 0 | 0.21 | 0 | 0 | 0 | 28 | 157.8 |
| 461 | 960 | 240 | 793.7 | 0 | 234 | 45 | 0 | 0.2 | 0 | 0 | 0 | 28 | 157.8 |
| 462 | 922 | 230 | 1152 | 0 | 184 | 11.52 | 0 | 0.16 | 0 | 0 | 0 | 28 | 156.8 |
| 463 | 1251.2 | 291.3 | 407.8 | 0 | 201.4 | 28.2 | 0 | 0.13 | 0 | 0 | 0 | 56 | 156.53 |
| 464 | 960 | 240 | 793.7 | 0 | 234 | 45 | 0 | 0.2 | 0 | 0 | 0 | 90 | 156.1 |
| 465 | 958.55 | 50.45 | 1231 | 0 | 201.8 | 34.2 | 0 | 0.2 | 0 | 0 | 0 | 90 | 155.2 |
| 466 | 998.8 | 176.25 | 1010.1 | 0 | 141 | 75.2 | 0 | 0.12 | 0 | 0 | 0 | 14 | 153.3 |
| 467 | 450 | 50 | 1992 | 0 | 90 | 18 | 0 | 0.18 | 0 | 0 | 0 | 90 | 151 |
| 468 | 900 | 108 | 1000 | 0 | 161.28 | 18 | 0 | 0.16 | 0 | 0 | 0 | 28 | 151 |
| 469 | 1009 | 0 | 1456.8 | 0 | 201.8 | 34.2 | 0 | 0.2 | 0 | 0 | 0 | 56 | 150.2 |
| 470 | 960 | 240 | 793.7 | 0 | 234 | 45 | 0 | 0.2 | 0 | 0 | 0 | 56 | 150.1 |
| 471 | 810 | 90 | 1539 | 0 | 162 | 18 | 0 | 0.18 | 0 | 0 | 0 | 56 | 150 |
| 472 | 810 | 90 | 1539 | 0 | 162 | 18 | 0 | 0.18 | 0 | 0 | 0 | 56 | 150 |
| 473 | 1009 | 0 | 1231 | 0 | 201.8 | 34.2 | 0 | 0.2 | 0 | 0 | 0 | 90 | 149.7 |
| 474 | 958.55 | 50.45 | 1456.8 | 0 | 201.8 | 34.2 | 0 | 0.2 | 0 | 0 | 0 | 56 | 149.6 |
| 475 | 853 | 170.6 | 1023.6 | 255.9 | 196.19 | 29.86 | 0 | 0.19 | 0 | 0 | 0 | 14 | 149.5 |
| 476 | 1251.2 | 291.3 | 407.8 | 0 | 201.4 | 28.2 | 0 | 0.13 | 0 | 0 | 0 | 56 | 149.2 |
| 477 | 958.55 | 50.45 | 1456.8 | 0 | 201.8 | 34.2 | 0 | 0.2 | 0 | 0 | 0 | 56 | 149 |
| 478 | 1009 | 0 | 1456.8 | 0 | 201.8 | 34.2 | 0 | 0.2 | 0 | 0 | 0 | 56 | 149 |
| 479 | 1009 | 0 | 1456.8 | 0 | 201.8 | 34.2 | 0 | 0.2 | 0 | 0 | 0 | 56 | 148.9 |
| 480 | 958.55 | 50.45 | 1456.8 | 0 | 201.8 | 34.2 | 0 | 0.2 | 0 | 0 | 0 | 56 | 148.4 |
| 481 | 750 | 158 | 444 | 662 | 189 | 37.5 | 0 | 0.21 | 0 | 0 | 0 | 28 | 148.2 |
| 482 | 801 | 89 | 1401.2 | 0 | 178 | 30.2 | 0 | 0.2 | 0 | 0 | 0 | 90 | 148.2 |
| 483 | 870 | 131 | 1238 | 0 | 180 | 8 | 0 | 0.2 | 0 | 0 | 0 | 28 | 148 |
| 484 | 801 | 89 | 1401.2 | 0 | 178 | 30.2 | 0 | 0.2 | 0 | 0 | 0 | 90 | 148 |
| 485 | 801 | 89 | 1401.2 | 0 | 178 | 30.2 | 0 | 0.2 | 0 | 0 | 0 | 56 | 147.8 |
| 486 | 800 | 100 | 980 | 200 | 200 | 9.6 | 0 | 0.22 | 0 | 0 | 0 | 28 | 147.3 |
| 487 | 1251.2 | 291.3 | 407.8 | 0 | 201.4 | 28.2 | 0 | 0.13 | 0 | 0 | 0 | 28 | 147.27 |
| 488 | 833 | 208 | 1125 | 0 | 208 | 25 | 0 | 0.2 | 0 | 0 | 0 | 28 | 147 |
| 489 | 801 | 89 | 1401.2 | 0 | 178 | 30.2 | 0 | 0.2 | 0 | 0 | 0 | 56 | 147 |
| 490 | 845.5 | 44.5 | 1401.2 | 0 | 178 | 30.2 | 0 | 0.2 | 0 | 0 | 0 | 90 | 146.8 |
| 491 | 1009 | 0 | 1456.8 | 0 | 201.8 | 34.2 | 0 | 0.2 | 0 | 0 | 0 | 56 | 146.7 |
| 492 | 801 | 89 | 1401.2 | 0 | 178 | 30.2 | 0 | 0.2 | 0 | 0 | 0 | 56 | 146.6 |
| 493 | 800 | 200 | 980 | 100 | 200 | 9.6 | 0 | 0.2 | 0 | 0 | 0 | 28 | 146.5 |
| 494 | 1009 | 0 | 1456.8 | 0 | 201.8 | 34.2 | 0 | 0.2 | 0 | 0 | 0 | 56 | 146.5 |
| 495 | 1116.7 | 390.85 | 625.35 | 0 | 212.17 | 23.45 | 0 | 0.14 | 0 | 0 | 0 | 28 | 146.5 |
| 496 | 998.8 | 176.25 | 1013.9 | 0 | 164.5 | 49.4 | 0 | 0.14 | 0 | 0 | 0 | 14 | 146.1 |
| 497 | 630 | 70 | 1788 | 0 | 126 | 18 | 0 | 0.18 | 0 | 0 | 0 | 56 | 146 |
| 498 | 801 | 89 | 1401.2 | 0 | 178 | 30.2 | 0 | 0.2 | 0 | 0 | 0 | 90 | 146 |
| 499 | 750 | 158 | 444 | 662 | 189 | 37.5 | 0 | 0.21 | 0 | 0 | 0 | 28 | 145.9 |
| 500 | 750 | 158 | 444 | 662 | 189 | 37.5 | 0 | 0.21 | 0 | 0 | 0 | 28 | 145.9 |
| 501 | 845.5 | 44.5 | 1401.2 | 0 | 178 | 30.2 | 0 | 0.2 | 0 | 0 | 0 | 90 | 145.8 |
| 502 | 845.5 | 44.5 | 1401.2 | 0 | 178 | 30.2 | 0 | 0.2 | 0 | 0 | 0 | 90 | 145.6 |
| 503 | 1076.77 | 286.23 | 1090.8 | 0 | 272.6 | 46.2 | 0 | 0.2 | 0 | 0 | 0 | 28 | 145.4 |
| 504 | 750 | 158 | 444 | 662 | 189 | 37.5 | 0 | 0.21 | 0 | 0 | 0 | 28 | 145.2 |
| 505 | 750 | 158 | 444 | 662 | 189 | 37.5 | 0 | 0.21 | 0 | 0 | 0 | 28 | 145.2 |
| 506 | 845.5 | 44.5 | 1401.2 | 0 | 178 | 30.2 | 0 | 0.2 | 0 | 0 | 0 | 90 | 145.1 |
| 507 | 870 | 131 | 1183 | 0 | 200 | 9 | 0 | 0.2 | 0 | 0 | 0 | 28 | 145 |
| 508 | 845.5 | 44.5 | 1401.2 | 0 | 178 | 30.2 | 0 | 0.2 | 0 | 0 | 0 | 90 | 144.8 |
| 509 | 828 | 207 | 911 | 248 | 186.4 | 21 | 0 | 0.18 | 0 | 0 | 0 | 28 | 144.6 |
| 510 | 850 | 212.5 | 935 | 255 | 212.5 | 21.2 | 0 | 0.2 | 0 | 0 | 0 | 28 | 144.6 |
| 511 | 1009 | 0 | 1456.8 | 0 | 201.8 | 34.2 | 0 | 0.2 | 0 | 0 | 0 | 56 | 144.1 |
| 512 | 845.5 | 44.5 | 1401.2 | 0 | 178 | 30.2 | 0 | 0.2 | 0 | 0 | 0 | 90 | 143.7 |
| 513 | 845.5 | 44.5 | 1401.2 | 0 | 178 | 30.2 | 0 | 0.2 | 0 | 0 | 0 | 56 | 143.6 |
| 514 | 845.5 | 44.5 | 1401.2 | 0 | 178 | 30.2 | 0 | 0.2 | 0 | 0 | 0 | 56 | 143.5 |
| 515 | 1116.7 | 390.85 | 625.35 | 0 | 212.17 | 23.45 | 0 | 0.14 | 0 | 0 | 0 | 28 | 143.5 |
| 516 | 845.5 | 44.5 | 1401.2 | 0 | 178 | 30.2 | 0 | 0.2 | 0 | 0 | 0 | 56 | 143 |
| 517 | 870 | 131 | 1185 | 0 | 200 | 8 | 0 | 0.2 | 0 | 0 | 0 | 28 | 142 |
| 518 | 750 | 150 | 1362 | 0 | 162 | 7.2 | 0 | 0.2 | 0 | 0 | 0 | 28 | 142 |
| 519 | 756.5 | 133.5 | 1401.2 | 0 | 178 | 30.2 | 0 | 0.2 | 0 | 0 | 0 | 90 | 141.6 |
| 520 | 800 | 200 | 880 | 200 | 200 | 9.6 | 0 | 0.2 | 0 | 0 | 0 | 28 | 141.3 |
| 521 | 1251.2 | 291.3 | 407.8 | 0 | 201.4 | 28.2 | 0 | 0.13 | 0 | 0 | 0 | 28 | 141.3 |
| 522 | 800 | 100 | 980 | 200 | 200 | 9.6 | 0 | 0.22 | 0 | 0 | 0 | 90 | 141.1 |
| 523 | 870 | 131 | 1132 | 0 | 220 | 8 | 0 | 0.2 | 0 | 0 | 0 | 28 | 141 |
| 524 | 1251.2 | 291.3 | 407.8 | 0 | 201.4 | 28.2 | 0 | 0.13 | 0 | 0 | 0 | 14 | 140.83 |
| 525 | 750 | 158 | 444 | 662 | 189 | 37.5 | 0 | 0.21 | 0 | 0 | 0 | 28 | 140.7 |
| 526 | 1116.7 | 390.85 | 625.35 | 0 | 212.17 | 23.45 | 0 | 0.14 | 0 | 0 | 0 | 28 | 140.4 |
| 527 | 936 | 140.4 | 1170 | 0 | 224.64 | 28.08 | 0 | 0.21 | 0 | 0 | 0 | 28 | 140.1 |
| 528 | 750 | 150 | 1315 | 0 | 180 | 7.2 | 0 | 0.2 | 0 | 0 | 0 | 28 | 140 |
| 529 | 870 | 131 | 1188 | 0 | 200 | 7 | 0 | 0.2 | 0 | 0 | 0 | 28 | 140 |
| 530 | 756.5 | 133.5 | 1401.2 | 0 | 178 | 30.2 | 0 | 0.2 | 0 | 0 | 0 | 56 | 139.4 |
| 531 | 1009 | 0 | 1231 | 0 | 201.8 | 34.2 | 0 | 0.2 | 0 | 0 | 0 | 90 | 139.3 |
| 532 | 890 | 0 | 1401.2 | 0 | 178 | 30.2 | 0 | 0.2 | 0 | 0 | 0 | 90 | 139.1 |
| 533 | 833 | 167 | 1166 | 0 | 200 | 8 | 0 | 0.2 | 0 | 0 | 0 | 28 | 139 |
| 534 | 450 | 50 | 1992 | 0 | 90 | 18 | 0 | 0.18 | 0 | 0 | 0 | 56 | 139 |
| 535 | 801 | 89 | 1401.2 | 0 | 178 | 30.2 | 0 | 0.2 | 0 | 0 | 0 | 28 | 138.9 |
| 536 | 845.5 | 44.5 | 1401.2 | 0 | 178 | 30.2 | 0 | 0.2 | 0 | 0 | 0 | 56 | 138.9 |
| 537 | 756.5 | 133.5 | 1401.2 | 0 | 178 | 30.2 | 0 | 0.2 | 0 | 0 | 0 | 90 | 138.9 |
| 538 | 750 | 158 | 444 | 662 | 189 | 37.5 | 0 | 0.21 | 0 | 0 | 0 | 28 | 138.8 |
| 539 | 845.5 | 44.5 | 1401.2 | 0 | 178 | 30.2 | 0 | 0.2 | 0 | 0 | 0 | 56 | 138.8 |
| 540 | 950 | 255 | 873 | 0 | 189 | 31 | 0 | 0.16 | 0 | 0 | 0 | 90 | 138.7 |
| 541 | 756.5 | 133.5 | 1401.2 | 0 | 178 | 30.2 | 0 | 0.2 | 0 | 0 | 0 | 56 | 138.7 |
| 542 | 890 | 222 | 837 | 0 | 222 | 29.6 | 0 | 0.2 | 0 | 0 | 0 | 7 | 138.6 |
| 543 | 845.5 | 44.5 | 1401.2 | 0 | 178 | 30.2 | 0 | 0.2 | 0 | 0 | 0 | 56 | 138.4 |
| 544 | 756.5 | 133.5 | 1401.2 | 0 | 178 | 30.2 | 0 | 0.2 | 0 | 0 | 0 | 90 | 138.4 |
| 545 | 756.5 | 133.5 | 1401.2 | 0 | 178 | 30.2 | 0 | 0.2 | 0 | 0 | 0 | 56 | 138 |
| 546 | 998.8 | 176.25 | 1010.1 | 0 | 141 | 75.2 | 0 | 0.12 | 0 | 0 | 0 | 7 | 137.9 |
| 547 | 853 | 170.6 | 1023.6 | 255.9 | 196.19 | 29.86 | 0 | 0.19 | 0 | 0 | 0 | 7 | 137.9 |
| 548 | 712 | 178 | 1401.2 | 0 | 178 | 30.2 | 0 | 0.2 | 0 | 0 | 0 | 90 | 137.9 |
| 549 | 801 | 89 | 1401.2 | 0 | 178 | 30.2 | 0 | 0.2 | 0 | 0 | 0 | 28 | 137.4 |
| 550 | 810 | 90 | 1539 | 0 | 162 | 18 | 0 | 0.18 | 0 | 0 | 0 | 28 | 137 |
| 551 | 810 | 90 | 1539 | 0 | 162 | 18 | 0 | 0.18 | 0 | 0 | 0 | 28 | 137 |
| 552 | 756.5 | 133.5 | 1401.2 | 0 | 178 | 30.2 | 0 | 0.2 | 0 | 0 | 0 | 28 | 136.9 |
| 553 | 712 | 178 | 1401.2 | 0 | 178 | 30.2 | 0 | 0.2 | 0 | 0 | 0 | 90 | 136.8 |
| 554 | 960 | 240 | 793.7 | 0 | 234 | 45 | 0 | 0.2 | 0 | 0 | 0 | 28 | 136.6 |
| 555 | 845.5 | 44.5 | 1401.2 | 0 | 178 | 30.2 | 0 | 0.2 | 0 | 0 | 0 | 28 | 136.4 |
| 556 | 712 | 178 | 1401.2 | 0 | 178 | 30.2 | 0 | 0.2 | 0 | 0 | 0 | 56 | 136.4 |
| 557 | 756.5 | 133.5 | 1401.2 | 0 | 178 | 30.2 | 0 | 0.2 | 0 | 0 | 0 | 28 | 136.3 |
| 558 | 845.5 | 44.5 | 1401.2 | 0 | 178 | 30.2 | 0 | 0.2 | 0 | 0 | 0 | 28 | 136.3 |
| 559 | 712 | 178 | 1401.2 | 0 | 178 | 30.2 | 0 | 0.2 | 0 | 0 | 0 | 90 | 136.3 |
| 560 | 890 | 0 | 1401.2 | 0 | 178 | 30.2 | 0 | 0.2 | 0 | 0 | 0 | 90 | 136.1 |
| 561 | 805 | 224 | 971 | 0 | 195 | 15 | 0 | 0.19 | 0 | 0 | 0 | 28 | 136 |
| 562 | 845.5 | 44.5 | 1401.2 | 0 | 178 | 30.2 | 0 | 0.2 | 0 | 0 | 0 | 28 | 135.8 |
| 563 | 756.5 | 133.5 | 1401.2 | 0 | 178 | 30.2 | 0 | 0.2 | 0 | 0 | 0 | 28 | 135.7 |
| 564 | 712 | 178 | 1401.2 | 0 | 178 | 30.2 | 0 | 0.2 | 0 | 0 | 0 | 56 | 135.5 |
| 565 | 750 | 158 | 444 | 662 | 189 | 37.5 | 0 | 0.21 | 0 | 0 | 0 | 28 | 135.4 |
| 566 | 1251.2 | 291.3 | 407.8 | 0 | 201.4 | 28.2 | 0 | 0.13 | 0 | 0 | 0 | 14 | 135.1 |
| 567 | 750 | 150 | 1312 | 0 | 180 | 8.1 | 0 | 0.2 | 0 | 0 | 0 | 28 | 135 |
| 568 | 783 | 117 | 1333 | 0 | 180 | 7.2 | 0 | 0.2 | 0 | 0 | 0 | 28 | 135 |
| 569 | 630 | 70 | 1788 | 0 | 126 | 18 | 0 | 0.18 | 0 | 0 | 0 | 28 | 135 |
| 570 | 1009 | 0 | 1456.8 | 0 | 201.8 | 34.2 | 0 | 0.2 | 0 | 0 | 0 | 28 | 135 |
| 571 | 845.5 | 44.5 | 1401.2 | 0 | 178 | 30.2 | 0 | 0.2 | 0 | 0 | 0 | 28 | 134.8 |
| 572 | 788 | 197 | 866.8 | 315 | 173 | 14.77 | 0 | 0.18 | 0 | 0 | 0 | 21 | 134.7 |
| 573 | 788 | 197 | 866.8 | 315 | 173 | 14.77 | 0 | 0.18 | 0 | 0 | 0 | 28 | 134.7 |
| 574 | 801 | 89 | 1401.2 | 0 | 178 | 30.2 | 0 | 0.2 | 0 | 0 | 0 | 28 | 134.6 |
| 575 | 958.55 | 50.45 | 1456.8 | 0 | 201.8 | 34.2 | 0 | 0.2 | 0 | 0 | 0 | 28 | 134.2 |
| 576 | 712 | 178 | 1401.2 | 0 | 178 | 30.2 | 0 | 0.2 | 0 | 0 | 0 | 56 | 134.2 |
| 577 | 890 | 0 | 1401.2 | 0 | 178 | 30.2 | 0 | 0.2 | 0 | 0 | 0 | 90 | 134.1 |
| 578 | 750 | 150 | 1317 | 0 | 180 | 6.3 | 0 | 0.2 | 0 | 0 | 0 | 28 | 134 |
| 579 | 845.5 | 44.5 | 1401.2 | 0 | 178 | 30.2 | 0 | 0.2 | 0 | 0 | 0 | 28 | 134 |
| 580 | 863 | 216 | 1079 | 0 | 194.22 | 43.16 | 0 | 0.18 | 0 | 0 | 0 | 28 | 133.6 |
| 581 | 890 | 0 | 1401.2 | 0 | 178 | 30.2 | 0 | 0.2 | 0 | 0 | 0 | 90 | 133.1 |
| 582 | 696 | 104 | 1522 | 0 | 144 | 6.4 | 0 | 0.2 | 0 | 0 | 0 | 28 | 133 |
| 583 | 1009 | 0 | 1456.8 | 0 | 201.8 | 34.2 | 0 | 0.2 | 0 | 0 | 0 | 28 | 132.9 |
| 584 | 958.55 | 50.45 | 1456.8 | 0 | 201.8 | 34.2 | 0 | 0.2 | 0 | 0 | 0 | 28 | 132.8 |
| 585 | 958.55 | 50.45 | 1456.8 | 0 | 201.8 | 34.2 | 0 | 0.2 | 0 | 0 | 0 | 28 | 132.8 |
| 586 | 845.5 | 44.5 | 1401.2 | 0 | 178 | 30.2 | 0 | 0.2 | 0 | 0 | 0 | 28 | 132.8 |
| 587 | 850 | 150 | 1224 | 0 | 180 | 11.99 | 0 | 0.18 | 0 | 0 | 0 | 28 | 132.59 |
| 588 | 950 | 255 | 873 | 0 | 189 | 31 | 0 | 0.16 | 0 | 0 | 0 | 28 | 132.5 |
| 589 | 1009 | 0 | 1456.8 | 0 | 201.8 | 34.2 | 0 | 0.2 | 0 | 0 | 0 | 28 | 132.5 |
| 590 | 890 | 0 | 1401.2 | 0 | 178 | 30.2 | 0 | 0.2 | 0 | 0 | 0 | 56 | 132.1 |
| 591 | 750 | 158 | 444 | 662 | 189 | 37.5 | 0 | 0.21 | 0 | 0 | 0 | 28 | 131.9 |
| 592 | 890 | 0 | 1401.2 | 0 | 178 | 30.2 | 0 | 0.2 | 0 | 0 | 0 | 28 | 131.8 |
| 593 | 1009 | 0 | 1456.8 | 0 | 201.8 | 34.2 | 0 | 0.2 | 0 | 0 | 0 | 28 | 131.7 |
| 594 | 1009 | 0 | 1456.8 | 0 | 201.8 | 34.2 | 0 | 0.2 | 0 | 0 | 0 | 28 | 131.7 |
| 595 | 890 | 0 | 1401.2 | 0 | 178 | 30.2 | 0 | 0.2 | 0 | 0 | 0 | 56 | 131.4 |
| 596 | 890 | 0 | 1401.2 | 0 | 178 | 30.2 | 0 | 0.2 | 0 | 0 | 0 | 90 | 131.2 |
| 597 | 890 | 0 | 1401.2 | 0 | 178 | 30.2 | 0 | 0.2 | 0 | 0 | 0 | 90 | 131.1 |
| 598 | 450 | 50 | 1992 | 0 | 90 | 18 | 0 | 0.18 | 0 | 0 | 0 | 28 | 131 |
| 599 | 890 | 0 | 1401.2 | 0 | 178 | 30.2 | 0 | 0.2 | 0 | 0 | 0 | 56 | 130.7 |
| 600 | 720 | 80 | 1422.9 | 0 | 160 | 29.6 | 0 | 0.2 | 0 | 0 | 0 | 90 | 130.3 |
| 601 | 890 | 0 | 1401.2 | 0 | 178 | 30.2 | 0 | 0.2 | 0 | 0 | 0 | 28 | 130.3 |
| 602 | 890 | 0 | 1401.2 | 0 | 178 | 30.2 | 0 | 0.2 | 0 | 0 | 0 | 28 | 130.2 |
| 603 | 850 | 150 | 1173 | 0 | 200 | 14.03 | 0 | 0.2 | 0 | 0 | 0 | 28 | 129.95 |
| 604 | 1009 | 0 | 1456.8 | 0 | 201.8 | 34.2 | 0 | 0.2 | 0 | 0 | 0 | 28 | 129.7 |
| 605 | 712 | 178 | 1401.2 | 0 | 178 | 30.2 | 0 | 0.2 | 0 | 0 | 0 | 28 | 129.5 |
| 606 | 1009 | 0 | 1231 | 0 | 201.8 | 34.2 | 0 | 0.2 | 0 | 0 | 0 | 56 | 129.3 |
| 607 | 958.55 | 50.45 | 1231 | 0 | 201.8 | 34.2 | 0 | 0.2 | 0 | 0 | 0 | 56 | 129 |
| 608 | 998.8 | 176.25 | 1013.9 | 0 | 164.5 | 49.4 | 0 | 0.14 | 0 | 0 | 0 | 7 | 129 |
| 609 | 850.34 | 149.66 | 1224.49 | 0 | 180 | 11.99 | 0 | 0.18 | 0 | 0 | 0 | 7 | 128.9 |
| 610 | 1076.77 | 286.23 | 1090.8 | 0 | 272.6 | 46.2 | 0 | 0.2 | 0 | 0 | 0 | 28 | 128.7 |
| 611 | 720 | 180 | 1353.6 | 0 | 162 | 10.8 | 0 | 0.18 | 0 | 0 | 0 | 28 | 128.2 |
| 612 | 850 | 150 | 1173 | 0 | 200 | 11.99 | 0 | 0.2 | 0 | 0 | 0 | 28 | 128.1 |
| 613 | 696 | 104 | 1477 | 0 | 160 | 7.2 | 0 | 0.2 | 0 | 0 | 0 | 28 | 128 |
| 614 | 750 | 150 | 1267 | 0 | 198 | 7.2 | 0 | 0.2 | 0 | 0 | 0 | 28 | 128 |
| 615 | 801 | 89 | 1231 | 0 | 178 | 30.2 | 0 | 0.2 | 0 | 0 | 0 | 90 | 127.6 |
| 616 | 712 | 178 | 1401.2 | 0 | 178 | 30.2 | 0 | 0.2 | 0 | 0 | 0 | 28 | 127.6 |
| 617 | 845.5 | 44.5 | 1401.2 | 0 | 178 | 30.2 | 0 | 0.2 | 0 | 0 | 0 | 7 | 127.5 |
| 618 | 712 | 178 | 1401.2 | 0 | 178 | 30.2 | 0 | 0.2 | 0 | 0 | 0 | 28 | 127.5 |
| 619 | 801 | 89 | 1231 | 0 | 178 | 30.2 | 0 | 0.2 | 0 | 0 | 0 | 56 | 127.4 |
| 620 | 720 | 180 | 1296 | 0 | 180 | 12.6 | 0 | 0.2 | 0 | 0 | 0 | 28 | 127.32 |
| 621 | 850 | 150 | 1181.5 | 0 | 200 | 10.03 | 0 | 0.2 | 0 | 0 | 0 | 28 | 127.32 |
| 622 | 890 | 0 | 1401.2 | 0 | 178 | 30.2 | 0 | 0.2 | 0 | 0 | 0 | 56 | 126.8 |
| 623 | 800 | 200 | 1160 | 0 | 200 | 12 | 0 | 0.2 | 0 | 0 | 0 | 28 | 126.6 |
| 624 | 809 | 270 | 1079 | 0 | 177 | 21.6 | 0 | 0.16 | 0 | 0 | 0 | 90 | 126.5 |
| 625 | 1009 | 0 | 1231 | 0 | 201.8 | 34.2 | 0 | 0.2 | 0 | 0 | 0 | 56 | 126.2 |
| 626 | 845.5 | 44.5 | 1231 | 0 | 178 | 30.2 | 0 | 0.2 | 0 | 0 | 0 | 90 | 126.2 |
| 627 | 850.34 | 149.66 | 1173.47 | 0 | 200 | 14.03 | 0 | 0.2 | 0 | 0 | 0 | 7 | 126.2 |
| 628 | 696 | 104 | 1479 | 0 | 160 | 6.4 | 0 | 0.2 | 0 | 0 | 0 | 28 | 126 |
| 629 | 890 | 0 | 1401.2 | 0 | 178 | 30.2 | 0 | 0.2 | 0 | 0 | 0 | 56 | 125.7 |
| 630 | 720 | 180 | 1310.4 | 0 | 180 | 9 | 0 | 0.2 | 0 | 0 | 0 | 28 | 125.56 |
| 631 | 788 | 197 | 866.8 | 315 | 173 | 14.77 | 0 | 0.18 | 0 | 0 | 0 | 14 | 125.5 |
| 632 | 845.5 | 44.5 | 1231 | 0 | 178 | 30.2 | 0 | 0.2 | 0 | 0 | 0 | 90 | 125.4 |
| 633 | 720 | 80 | 1422.9 | 0 | 160 | 29.6 | 0 | 0.2 | 0 | 0 | 0 | 90 | 125.3 |
| 634 | 750 | 158 | 444 | 662 | 189 | 37.5 | 0 | 0.21 | 0 | 0 | 0 | 28 | 125 |
| 635 | 800 | 200 | 1148 | 0 | 200 | 8 | 0 | 0.2 | 0 | 0 | 0 | 28 | 125 |
| 636 | 696 | 104 | 1481 | 0 | 160 | 5.6 | 0 | 0.2 | 0 | 0 | 0 | 28 | 125 |
| 637 | 750 | 250 | 1140 | 0 | 200 | 12 | 0 | 0.2 | 0 | 0 | 0 | 28 | 125 |
| 638 | 1251.2 | 291.3 | 407.8 | 0 | 201.4 | 28.2 | 0 | 0.13 | 0 | 0 | 0 | 7 | 124.94 |
| 639 | 890 | 0 | 1401.2 | 0 | 178 | 30.2 | 0 | 0.2 | 0 | 0 | 0 | 28 | 124.9 |
| 640 | 890 | 0 | 1401.2 | 0 | 178 | 30.2 | 0 | 0.2 | 0 | 0 | 0 | 56 | 124.5 |
| 641 | 720 | 180 | 1353.6 | 0 | 162 | 10.8 | 0 | 0.18 | 0 | 0 | 0 | 7 | 124.3 |
| 642 | 720 | 180 | 1303.2 | 0 | 180 | 10.8 | 0 | 0.2 | 0 | 0 | 0 | 28 | 124.2 |
| 643 | 720 | 80 | 1422.9 | 0 | 160 | 29.6 | 0 | 0.2 | 0 | 0 | 0 | 56 | 124.2 |
| 644 | 845.5 | 44.5 | 1231 | 0 | 178 | 30.2 | 0 | 0.2 | 0 | 0 | 0 | 56 | 124.1 |
| 645 | 800 | 0 | 1471.3 | 0 | 160 | 21.6 | 0 | 0.2 | 0 | 0 | 0 | 90 | 124.1 |
| 646 | 828 | 207 | 911 | 248 | 186.4 | 21 | 0 | 0.18 | 0 | 0 | 0 | 7 | 124.1 |
| 647 | 845.5 | 44.5 | 1401.2 | 0 | 178 | 30.2 | 0 | 0.2 | 0 | 0 | 0 | 7 | 124 |
| 648 | 960 | 240 | 793.7 | 0 | 234 | 45 | 0 | 0.2 | 0 | 0 | 0 | 7 | 124 |
| 649 | 720 | 180 | 1296 | 0 | 180 | 12.6 | 0 | 0.2 | 0 | 0 | 0 | 7 | 124 |
| 650 | 850.34 | 149.66 | 1173.47 | 0 | 200 | 11.99 | 0 | 0.2 | 0 | 0 | 0 | 7 | 124 |
| 651 | 800 | 200 | 1160 | 0 | 200 | 12 | 0 | 0.2 | 0 | 0 | 0 | 7 | 123.5 |
| 652 | 850.34 | 149.66 | 1181.97 | 0 | 200 | 10.03 | 0 | 0.2 | 0 | 0 | 0 | 7 | 123.5 |
| 653 | 675 | 225 | 1289.25 | 0 | 180 | 10.8 | 0 | 0.2 | 0 | 0 | 0 | 28 | 123.4 |
| 654 | 850 | 212.5 | 935 | 255 | 212.5 | 21.2 | 0 | 0.2 | 0 | 0 | 0 | 28 | 123.2 |
| 655 | 809 | 270 | 1079 | 0 | 177 | 21.6 | 0 | 0.16 | 0 | 0 | 0 | 28 | 123.2 |
| 656 | 680 | 120 | 1502.8 | 0 | 144 | 9.59 | 0 | 0.18 | 0 | 0 | 0 | 28 | 122.93 |
| 657 | 850 | 150 | 1122 | 0 | 220 | 11.99 | 0 | 0.22 | 0 | 0 | 0 | 28 | 122.93 |
| 658 | 765 | 135 | 1315.8 | 0 | 180 | 10.79 | 0 | 0.2 | 0 | 0 | 0 | 28 | 122.6 |
| 659 | 890 | 222 | 837 | 0 | 222 | 29.6 | 0 | 0.2 | 0 | 0 | 0 | 28 | 122.4 |
| 660 | 720 | 180 | 1310.4 | 0 | 180 | 9 | 0 | 0.2 | 0 | 0 | 0 | 7 | 122.3 |
| 661 | 890 | 0 | 1401.2 | 0 | 178 | 30.2 | 0 | 0.2 | 0 | 0 | 0 | 28 | 122.1 |
| 662 | 680 | 120 | 1462 | 0 | 160 | 11.22 | 0 | 0.2 | 0 | 0 | 0 | 28 | 122.05 |
| 663 | 720 | 180 | 1260 | 0 | 198 | 10.8 | 0 | 0.22 | 0 | 0 | 0 | 28 | 122.05 |
| 664 | 845.5 | 44.5 | 1401.2 | 0 | 178 | 30.2 | 0 | 0.2 | 0 | 0 | 0 | 7 | 121.9 |
| 665 | 890 | 0 | 1401.2 | 0 | 178 | 30.2 | 0 | 0.2 | 0 | 0 | 0 | 28 | 121.4 |
| 666 | 845.5 | 44.5 | 1401.2 | 0 | 178 | 30.2 | 0 | 0.2 | 0 | 0 | 0 | 7 | 121.3 |
| 667 | 958.55 | 50.45 | 1456.8 | 0 | 201.8 | 34.2 | 0 | 0.2 | 0 | 0 | 0 | 7 | 121.3 |
| 668 | 756.5 | 133.5 | 1231 | 0 | 178 | 30.2 | 0 | 0.2 | 0 | 0 | 0 | 90 | 120.9 |
| 669 | 845.5 | 44.5 | 1401.2 | 0 | 178 | 30.2 | 0 | 0.2 | 0 | 0 | 0 | 7 | 120.9 |
| 670 | 750.19 | 249.81 | 1140.29 | 0 | 200 | 12 | 0 | 0.2 | 0 | 0 | 0 | 7 | 120.8 |
| 671 | 720 | 80 | 1422.9 | 0 | 160 | 29.6 | 0 | 0.2 | 0 | 0 | 0 | 28 | 120.7 |
| 672 | 720 | 180 | 1303.2 | 0 | 180 | 10.8 | 0 | 0.2 | 0 | 0 | 0 | 7 | 120.6 |
| 673 | 1009 | 0 | 1456.8 | 0 | 201.8 | 34.2 | 0 | 0.2 | 0 | 0 | 0 | 7 | 120.5 |
| 674 | 1116.7 | 390.85 | 625.35 | 0 | 212.17 | 23.45 | 0 | 0.14 | 0 | 0 | 0 | 7 | 120.2 |
| 675 | 845.5 | 44.5 | 1231 | 0 | 178 | 30.2 | 0 | 0.2 | 0 | 0 | 0 | 56 | 120.1 |
| 676 | 756.5 | 133.5 | 1231 | 0 | 178 | 30.2 | 0 | 0.2 | 0 | 0 | 0 | 56 | 120.1 |
| 677 | 720 | 180 | 1298 | 0 | 180 | 7.2 | 0 | 0.2 | 0 | 0 | 0 | 28 | 120 |
| 678 | 667 | 133 | 1463 | 0 | 160 | 6.4 | 0 | 0.2 | 0 | 0 | 0 | 28 | 120 |

| Appendix A-3: Filtered_Dataset.csv | | | | | | | | | | | | | | | | | |
| --- | --- | --- | --- | --- | --- | --- | --- | --- | --- | --- | --- | --- | --- | --- | --- | --- | --- |
| N | C | SF | S | QP | W | SP | F | WB | T | CT | SPB | FT | FL | FD | ST | A | CS |
| 1 | 850 | 260 | 850 | 212 | 170 | 45 | 156 | 0.15 | 23 | 1 | 1 | 1 | 13 | 0.2 | 1 | 28 | 200 |
| 2 | 850 | 260 | 850 | 212 | 170 | 45 | 156 | 0.15 | 23 | 1 | 1 | 1 | 13 | 0.2 | 1 | 28 | 199.9 |
| 3 | 788.5 | 433.7 | 867.4 | 0 | 160.3 | 52.6 | 78 | 0.13 | 23 | 1 | 1 | 1 | 13 | 0.2 | 1 | 28 | 199.6 |
| 4 | 960 | 240 | 754 | 0 | 234 | 45 | 14.4 | 0.2 | 23 | 1 | 1 | 6 | 6 | 0.16 | 1 | 56 | 199.4 |
| 5 | 828 | 207 | 911 | 248 | 186.4 | 21 | 24.84 | 0.18 | 23 | 1 | 1 | 6 | 12 | 0.2 | 1 | 28 | 198.9 |
| 6 | 1116.7 | 390.85 | 625.35 | 0 | 212.17 | 23.45 | 33.501 | 0.14 | 23 | 1 | 1 | 6 | 13 | 0.2 | 1 | 42 | 198.7 |
| 7 | 788.5 | 433.7 | 867.4 | 0 | 160.3 | 52.6 | 39 | 0.13 | 23 | 1 | 1 | 1 | 13 | 0.2 | 1 | 28 | 198.6 |
| 8 | 850 | 260 | 850 | 212 | 170 | 45 | 156 | 0.15 | 23 | 1 | 1 | 1 | 13 | 0.2 | 1 | 28 | 198.5 |
| 9 | 850 | 260 | 850 | 212 | 170 | 45 | 156 | 0.15 | 23 | 1 | 1 | 1 | 13 | 0.2 | 1 | 28 | 197.9 |
| 10 | 850 | 260 | 850 | 212 | 170 | 45 | 156 | 0.15 | 23 | 1 | 1 | 1 | 13 | 0.2 | 1 | 28 | 197.6 |
| 11 | 960 | 240 | 767.2 | 0 | 234 | 45 | 9.6 | 0.2 | 23 | 1 | 1 | 6 | 6 | 0.16 | 1 | 56 | 197.2 |
| 12 | 960 | 240 | 773.8 | 0 | 234 | 45 | 7.2 | 0.2 | 23 | 1 | 1 | 6 | 6 | 0.16 | 1 | 90 | 197.2 |
| 13 | 1076.77 | 286.23 | 1090.8 | 0 | 272.6 | 46.2 | 32.3031 | 0.2 | 23 | 1 | 1 | 6 | 12.7 | 0.2 | 1 | 28 | 197 |
| 14 | 850 | 260 | 850 | 212 | 170 | 45 | 156 | 0.15 | 23 | 1 | 1 | 1 | 13 | 0.2 | 1 | 28 | 196.9 |
| 15 | 850 | 260 | 850 | 212 | 170 | 45 | 156 | 0.15 | 23 | 1 | 1 | 1 | 13 | 0.2 | 1 | 28 | 196.9 |
| 16 | 1116.7 | 390.85 | 625.35 | 0 | 212.17 | 23.45 | 55.835 | 0.14 | 23 | 1 | 1 | 6 | 13 | 0.2 | 1 | 28 | 196.8 |
| 17 | 1116.7 | 390.85 | 625.35 | 0 | 212.17 | 23.45 | 22.334 | 0.14 | 23 | 1 | 1 | 6 | 13 | 0.2 | 1 | 42 | 196.4 |
| 18 | 960 | 240 | 780.5 | 0 | 234 | 45 | 4.8 | 0.2 | 23 | 1 | 1 | 6 | 6 | 0.16 | 1 | 90 | 195.4 |
| 19 | 788.5 | 433.7 | 867.4 | 0 | 160.3 | 52.6 | 0 | 0.13 | 23 | 1 | 1 | 1 | 13 | 0.2 | 1 | 28 | 195.2 |
| 20 | 850 | 260 | 850 | 212 | 170 | 45 | 156 | 0.15 | 23 | 1 | 1 | 1 | 13 | 0.2 | 1 | 28 | 195.2 |
| 21 | 850 | 260 | 850 | 212 | 170 | 45 | 156 | 0.15 | 23 | 1 | 1 | 1 | 13 | 0.2 | 1 | 28 | 194.9 |
| 22 | 853 | 170.6 | 1023.6 | 255.9 | 196.19 | 29.86 | 51.18 | 0.19 | 23 | 1 | 1 | 6 | 16 | 0.2 | 1 | 56 | 194.7 |
| 23 | 960 | 240 | 698.3 | 0 | 234 | 78 | 14.4 | 0.2 | 23 | 1 | 1 | 5 | 13 | 0.018 | 1 | 90 | 194.3 |
| 24 | 853 | 170.6 | 1023.6 | 255.9 | 196.19 | 29.86 | 51.18 | 0.19 | 23 | 1 | 1 | 6 | 12 | 0.2 | 1 | 56 | 193.7 |
| 25 | 960 | 240 | 773.8 | 0 | 234 | 45 | 7.2 | 0.2 | 23 | 1 | 1 | 6 | 6 | 0.16 | 1 | 56 | 193.6 |
| 26 | 960 | 240 | 787.1 | 0 | 234 | 45 | 2.4 | 0.2 | 23 | 1 | 1 | 6 | 6 | 0.16 | 1 | 90 | 193.6 |
| 27 | 850 | 260 | 850 | 212 | 170 | 45 | 156 | 0.15 | 23 | 1 | 1 | 1 | 13 | 0.2 | 1 | 28 | 193.5 |
| 28 | 784 | 261 | 1045 | 0 | 171 | 20.9 | 23.52 | 0.16 | 23 | 1 | 1 | 6 | 13 | 0.2 | 1 | 90 | 192.7 |
| 29 | 788.5 | 197.1 | 1104 | 0 | 160.3 | 50 | 156 | 0.16 | 23 | 1 | 1 | 1 | 13 | 0.2 | 1 | 28 | 192.3 |
| 30 | 850 | 260 | 850 | 212 | 170 | 45 | 156 | 0.15 | 23 | 1 | 1 | 1 | 13 | 0.2 | 1 | 28 | 192.2 |
| 31 | 850 | 260 | 850 | 212 | 170 | 45 | 156 | 0.15 | 23 | 1 | 1 | 1 | 13 | 0.2 | 1 | 28 | 192.2 |
| 32 | 960 | 240 | 780.5 | 0 | 234 | 45 | 4.8 | 0.2 | 23 | 1 | 1 | 6 | 6 | 0.16 | 1 | 56 | 191.8 |
| 33 | 1076.77 | 286.23 | 1090.8 | 0 | 272.6 | 46.2 | 0 | 0.2 | 23 | 1 | 1 | 0 | 0 | 0 | 1 | 28 | 191.5 |
| 34 | 850 | 260 | 850 | 212 | 170 | 45 | 156 | 0.15 | 23 | 1 | 1 | 1 | 13 | 0.2 | 1 | 28 | 191.2 |
| 35 | 960 | 240 | 798.8 | 0 | 234 | 39 | 9.6 | 0.2 | 23 | 1 | 1 | 5 | 13 | 0.018 | 1 | 90 | 191.1 |
| 36 | 853 | 170.6 | 1023.6 | 255.9 | 196.19 | 29.86 | 51.18 | 0.19 | 23 | 1 | 1 | 6 | 8 | 0.2 | 1 | 56 | 190.5 |
| 37 | 850 | 260 | 850 | 212 | 170 | 45 | 156 | 0.15 | 23 | 1 | 1 | 1 | 13 | 0.2 | 1 | 28 | 190.2 |
| 38 | 1116.7 | 390.85 | 625.35 | 0 | 212.17 | 23.45 | 44.668 | 0.14 | 23 | 1 | 1 | 6 | 13 | 0.2 | 1 | 28 | 189.4 |
| 39 | 1116.7 | 390.85 | 625.35 | 0 | 212.17 | 23.45 | 0 | 0.14 | 23 | 1 | 1 | 0 | 0 | 0 | 1 | 42 | 189.4 |
| 40 | 1076.77 | 286.23 | 1090.8 | 0 | 272.6 | 46.2 | 0 | 0.2 | 23 | 1 | 1 | 0 | 0 | 0 | 1 | 28 | 189.3 |
| 41 | 960 | 240 | 793.9 | 0 | 234 | 42 | 7.2 | 0.2 | 23 | 1 | 1 | 5 | 13 | 0.018 | 1 | 90 | 188.9 |
| 42 | 960 | 240 | 787.1 | 0 | 234 | 45 | 2.4 | 0.2 | 23 | 1 | 1 | 6 | 6 | 0.16 | 1 | 56 | 188.6 |
| 43 | 960 | 240 | 740.7 | 0 | 234 | 45 | 19.2 | 0.2 | 23 | 1 | 1 | 6 | 6 | 0.16 | 1 | 28 | 188.3 |
| 44 | 960 | 240 | 788.9 | 0 | 234 | 45 | 4.8 | 0.2 | 23 | 1 | 1 | 5 | 13 | 0.018 | 1 | 90 | 188.3 |
| 45 | 850 | 260 | 850 | 212 | 170 | 45 | 156 | 0.15 | 23 | 1 | 1 | 1 | 13 | 0.2 | 1 | 28 | 188.2 |
| 46 | 960 | 240 | 698.3 | 0 | 234 | 78 | 14.4 | 0.2 | 23 | 1 | 1 | 5 | 13 | 0.018 | 1 | 56 | 188.1 |
| 47 | 850 | 260 | 850 | 212 | 170 | 45 | 156 | 0.15 | 23 | 1 | 1 | 1 | 13 | 0.2 | 1 | 28 | 187.5 |
| 48 | 788.5 | 197.1 | 1104 | 0 | 160.3 | 50 | 156 | 0.16 | 23 | 1 | 1 | 1 | 13 | 0.2 | 1 | 28 | 187.1 |
| 49 | 792 | 264 | 1056 | 0 | 173 | 21.1 | 15.84 | 0.16 | 23 | 1 | 1 | 6 | 13 | 0.2 | 1 | 90 | 187 |
| 50 | 850 | 260 | 850 | 212 | 170 | 45 | 156 | 0.15 | 23 | 1 | 1 | 1 | 13 | 0.2 | 1 | 28 | 186.8 |
| 51 | 788.5 | 433.7 | 867.4 | 0 | 160.3 | 52.6 | 78 | 0.13 | 23 | 1 | 1 | 1 | 13 | 0.2 | 1 | 28 | 186.7 |
| 52 | 850 | 260 | 850 | 212 | 170 | 45 | 156 | 0.15 | 23 | 1 | 1 | 1 | 13 | 0.2 | 1 | 28 | 186.1 |
| 53 | 960 | 240 | 791.3 | 0 | 234 | 45 | 2.4 | 0.2 | 23 | 1 | 1 | 5 | 13 | 0.018 | 1 | 90 | 185.8 |
| 54 | 960 | 240 | 754 | 0 | 234 | 45 | 14.4 | 0.2 | 23 | 1 | 1 | 6 | 6 | 0.16 | 1 | 28 | 185.4 |
| 55 | 853 | 170.6 | 1023.6 | 255.9 | 196.19 | 29.86 | 25.59 | 0.19 | 23 | 1 | 1 | 6 | 12 | 0.2 | 1 | 56 | 185.3 |
| 56 | 850 | 260 | 850 | 212 | 170 | 45 | 156 | 0.15 | 23 | 1 | 1 | 1 | 13 | 0.2 | 1 | 28 | 185.2 |
| 57 | 1076.77 | 286.23 | 1090.8 | 0 | 272.6 | 46.2 | 0 | 0.2 | 23 | 1 | 1 | 0 | 0 | 0 | 1 | 28 | 184.5 |
| 58 | 850 | 260 | 850 | 212 | 170 | 45 | 156 | 0.15 | 23 | 1 | 1 | 1 | 13 | 0.2 | 1 | 28 | 184.4 |
| 59 | 998.8 | 176.25 | 954.5 | 0 | 141 | 81.7 | 14.982 | 0.12 | 23 | 1 | 1 | 5 | 13 | 0.018 | 1 | 28 | 184.4 |
| 60 | 853 | 170.6 | 1023.6 | 255.9 | 196.19 | 29.86 | 25.59 | 0.19 | 23 | 1 | 1 | 6 | 16 | 0.2 | 1 | 56 | 184.2 |
| 61 | 850 | 260 | 850 | 212 | 170 | 45 | 156 | 0.15 | 23 | 1 | 1 | 1 | 13 | 0.2 | 1 | 28 | 184 |
| 62 | 788 | 197 | 866.8 | 315 | 173 | 14.77 | 19.7 | 0.18 | 23 | 1 | 1 | 6 | 13 | 0.16 | 1 | 28 | 184 |
| 63 | 960 | 240 | 798.8 | 0 | 234 | 39 | 9.6 | 0.2 | 23 | 1 | 1 | 5 | 13 | 0.018 | 1 | 56 | 183.7 |
| 64 | 960 | 240 | 793.9 | 0 | 234 | 42 | 7.2 | 0.2 | 23 | 1 | 1 | 5 | 13 | 0.018 | 1 | 56 | 183.4 |
| 65 | 853 | 170.6 | 1023.6 | 255.9 | 196.19 | 29.86 | 25.59 | 0.19 | 23 | 1 | 1 | 6 | 8 | 0.2 | 1 | 56 | 183.2 |
| 66 | 784 | 261 | 1045 | 0 | 171 | 20.9 | 23.52 | 0.16 | 23 | 1 | 1 | 6 | 13 | 0.2 | 1 | 28 | 182.9 |
| 67 | 850 | 260 | 850 | 212 | 170 | 45 | 156 | 0.15 | 23 | 1 | 1 | 1 | 13 | 0.2 | 1 | 28 | 182.4 |
| 68 | 850 | 260 | 850 | 212 | 170 | 45 | 156 | 0.15 | 23 | 1 | 1 | 1 | 13 | 0.2 | 1 | 28 | 182.4 |
| 69 | 788 | 197 | 866.8 | 315 | 173 | 14.77 | 19.7 | 0.18 | 23 | 1 | 1 | 6 | 6 | 0.16 | 1 | 28 | 182.3 |
| 70 | 853 | 170.6 | 1023.6 | 255.9 | 196.19 | 29.86 | 51.18 | 0.19 | 23 | 1 | 1 | 6 | 12 | 0.2 | 1 | 28 | 182.1 |
| 71 | 850 | 260 | 850 | 212 | 170 | 45 | 156 | 0.15 | 23 | 1 | 1 | 1 | 13 | 0.2 | 1 | 28 | 181.7 |
| 72 | 850 | 260 | 850 | 212 | 170 | 45 | 156 | 0.15 | 23 | 1 | 1 | 1 | 13 | 0.2 | 1 | 28 | 181 |
| 73 | 850 | 260 | 850 | 212 | 170 | 45 | 156 | 0.15 | 23 | 1 | 1 | 1 | 13 | 0.2 | 1 | 28 | 181 |
| 74 | 998.8 | 176.25 | 975 | 0 | 141 | 78.7 | 9.988 | 0.12 | 23 | 1 | 1 | 5 | 13 | 0.018 | 1 | 28 | 181 |
| 75 | 850 | 260 | 850 | 212 | 170 | 45 | 156 | 0.15 | 23 | 1 | 1 | 1 | 13 | 0.2 | 1 | 28 | 180.8 |
| 76 | 958.55 | 50.45 | 1456.8 | 0 | 201.8 | 34.2 | 0 | 0.2 | 23 | 1 | 1 | 0 | 0 | 0 | 1 | 90 | 180.8 |
| 77 | 960 | 240 | 788.9 | 0 | 234 | 45 | 4.8 | 0.2 | 23 | 1 | 1 | 5 | 13 | 0.018 | 1 | 56 | 180.6 |
| 78 | 960 | 240 | 793.7 | 0 | 234 | 45 | 0 | 0.2 | 23 | 1 | 1 | 0 | 0 | 0 | 1 | 90 | 180.4 |
| 79 | 960 | 240 | 740.7 | 0 | 234 | 45 | 156 | 0.2 | 23 | 1 | 1 | 1 | 13 | 0.2 | 1 | 90 | 180.3 |
| 80 | 850 | 260 | 850 | 212 | 170 | 45 | 156 | 0.15 | 23 | 1 | 1 | 1 | 13 | 0.2 | 1 | 28 | 180.3 |
| 81 | 998.8 | 176.25 | 930.6 | 0 | 164.5 | 55.2 | 24.97 | 0.14 | 23 | 1 | 1 | 5 | 13 | 0.018 | 1 | 28 | 180.1 |
| 82 | 998.8 | 176.25 | 946.8 | 0 | 164.5 | 54.1 | 19.976 | 0.14 | 23 | 1 | 1 | 5 | 13 | 0.018 | 1 | 28 | 180.1 |
| 83 | 998.8 | 176.25 | 962.9 | 0 | 164.5 | 52.9 | 14.982 | 0.14 | 23 | 1 | 1 | 5 | 13 | 0.018 | 1 | 28 | 180.1 |
| 84 | 958.55 | 50.45 | 1456.8 | 0 | 201.8 | 34.2 | 0 | 0.2 | 23 | 1 | 1 | 0 | 0 | 0 | 1 | 90 | 180.1 |
| 85 | 853 | 170.6 | 1023.6 | 255.9 | 196.19 | 29.86 | 51.18 | 0.19 | 23 | 1 | 1 | 6 | 8 | 0.2 | 1 | 28 | 180 |
| 86 | 788 | 197 | 866.8 | 315 | 173 | 14.77 | 19.7 | 0.18 | 23 | 1 | 1 | 6 | 13 | 0.16 | 1 | 21 | 180 |
| 87 | 998.8 | 176.25 | 900.1 | 0 | 164.5 | 62.3 | 29.964 | 0.14 | 23 | 1 | 1 | 5 | 13 | 0.018 | 1 | 28 | 179.9 |
| 88 | 853 | 170.6 | 1023.6 | 255.9 | 196.19 | 29.86 | 51.18 | 0.19 | 23 | 1 | 1 | 6 | 16 | 0.2 | 1 | 28 | 178.9 |
| 89 | 960 | 240 | 767.2 | 0 | 234 | 45 | 9.6 | 0.2 | 23 | 1 | 1 | 6 | 6 | 0.16 | 1 | 28 | 178.9 |
| 90 | 998.8 | 176.25 | 981.9 | 0 | 164.5 | 50.5 | 9.988 | 0.14 | 23 | 1 | 1 | 5 | 13 | 0.018 | 1 | 28 | 178.6 |
| 91 | 960 | 240 | 791.3 | 0 | 234 | 45 | 2.4 | 0.2 | 23 | 1 | 1 | 5 | 13 | 0.018 | 1 | 56 | 178.4 |
| 92 | 960 | 240 | 698.3 | 0 | 234 | 78 | 14.4 | 0.2 | 23 | 1 | 1 | 5 | 13 | 0.018 | 1 | 28 | 177.3 |
| 93 | 958.55 | 50.45 | 1456.8 | 0 | 201.8 | 34.2 | 0 | 0.2 | 23 | 1 | 1 | 0 | 0 | 0 | 1 | 90 | 176.9 |
| 94 | 960 | 240 | 754 | 0 | 234 | 45 | 117 | 0.2 | 23 | 1 | 1 | 1 | 13 | 0.2 | 1 | 90 | 176.8 |
| 95 | 850 | 260 | 850 | 212 | 170 | 45 | 156 | 0.15 | 23 | 1 | 1 | 1 | 13 | 0.2 | 1 | 28 | 176.6 |
| 96 | 863 | 216 | 1079 | 0 | 194.22 | 43.16 | 25.89 | 0.18 | 23 | 1 | 1 | 6 | 13 | 0.2 | 1 | 28 | 176.4 |
| 97 | 960 | 240 | 773.8 | 0 | 234 | 45 | 7.2 | 0.2 | 23 | 1 | 1 | 6 | 6 | 0.16 | 1 | 28 | 176.1 |
| 98 | 800 | 200 | 980 | 100 | 200 | 9.6 | 0 | 0.2 | 20 | 1 | 1 | 0 | 0 | 0 | 1 | 90 | 175.9 |
| 99 | 853 | 170.6 | 1023.6 | 255.9 | 196.19 | 29.86 | 8.53 | 0.19 | 23 | 1 | 1 | 6 | 16 | 0.2 | 1 | 56 | 175.8 |
| 100 | 1116.7 | 390.85 | 625.35 | 0 | 212.17 | 23.45 | 33.501 | 0.14 | 23 | 1 | 1 | 6 | 13 | 0.2 | 1 | 28 | 175.6 |
| 101 | 788 | 197 | 866.8 | 315 | 173 | 14.77 | 19.7 | 0.18 | 23 | 1 | 1 | 6 | 6 | 0.16 | 1 | 21 | 175.5 |
| 102 | 828 | 207 | 911 | 248 | 186.4 | 21 | 24.84 | 0.18 | 23 | 1 | 1 | 4 | 10 | 0.44 | 1 | 28 | 175.3 |
| 103 | 1009 | 0 | 1456.8 | 0 | 201.8 | 34.2 | 0 | 0.2 | 23 | 1 | 1 | 0 | 0 | 0 | 1 | 90 | 175.3 |
| 104 | 853 | 170.6 | 1023.6 | 255.9 | 196.19 | 29.86 | 25.59 | 0.19 | 23 | 1 | 1 | 6 | 12 | 0.2 | 1 | 28 | 174.7 |
| 105 | 850 | 260 | 850 | 212 | 170 | 45 | 156 | 0.15 | 23 | 1 | 1 | 1 | 13 | 0.2 | 1 | 28 | 174.6 |
| 106 | 1076.77 | 286.23 | 1090.8 | 0 | 272.6 | 46.2 | 32.3031 | 0.2 | 23 | 1 | 1 | 6 | 12.7 | 0.2 | 1 | 28 | 174.5 |
| 107 | 1116.7 | 390.85 | 625.35 | 0 | 212.17 | 23.45 | 33.501 | 0.14 | 23 | 1 | 1 | 6 | 13 | 0.2 | 1 | 28 | 174.4 |
| 108 | 788 | 197 | 866.8 | 315 | 173 | 14.77 | 15.76 | 0.18 | 23 | 1 | 1 | 6 | 13 | 0.16 | 1 | 28 | 173.8 |
| 109 | 853 | 170.6 | 1023.6 | 255.9 | 196.19 | 29.86 | 25.59 | 0.19 | 23 | 1 | 1 | 6 | 16 | 0.2 | 1 | 28 | 173.7 |
| 110 | 853 | 170.6 | 1023.6 | 255.9 | 196.19 | 29.86 | 8.53 | 0.19 | 23 | 1 | 1 | 6 | 8 | 0.2 | 1 | 56 | 173.7 |
| 111 | 853 | 170.6 | 1023.6 | 255.9 | 196.19 | 29.86 | 8.53 | 0.19 | 23 | 1 | 1 | 6 | 12 | 0.2 | 1 | 56 | 173.7 |
| 112 | 960 | 240 | 767.2 | 0 | 234 | 45 | 78 | 0.2 | 23 | 1 | 1 | 1 | 13 | 0.2 | 1 | 90 | 173.6 |
| 113 | 960 | 240 | 780.5 | 0 | 234 | 45 | 4.8 | 0.2 | 23 | 1 | 1 | 6 | 6 | 0.16 | 1 | 28 | 173.6 |
| 114 | 960 | 240 | 793.7 | 0 | 234 | 45 | 0 | 0.2 | 23 | 1 | 1 | 0 | 0 | 0 | 1 | 56 | 173.6 |
| 115 | 1116.7 | 390.85 | 625.35 | 0 | 212.17 | 23.45 | 44.668 | 0.14 | 23 | 1 | 1 | 6 | 13 | 0.2 | 1 | 28 | 173.3 |
| 116 | 960 | 240 | 740.7 | 0 | 234 | 45 | 156 | 0.2 | 23 | 1 | 1 | 1 | 13 | 0.2 | 1 | 56 | 173.2 |
| 117 | 850 | 260 | 850 | 212 | 170 | 45 | 156 | 0.15 | 23 | 1 | 1 | 1 | 13 | 0.2 | 1 | 28 | 173.2 |
| 118 | 792 | 264 | 1056 | 0 | 173 | 21.1 | 15.84 | 0.16 | 23 | 1 | 1 | 6 | 13 | 0.2 | 1 | 28 | 173.1 |
| 119 | 850 | 260 | 850 | 212 | 170 | 45 | 156 | 0.15 | 23 | 1 | 1 | 1 | 13 | 0.2 | 1 | 28 | 172.9 |
| 120 | 960 | 240 | 754 | 0 | 234 | 45 | 117 | 0.2 | 23 | 1 | 1 | 1 | 13 | 0.2 | 1 | 56 | 172.7 |
| 121 | 853 | 170.6 | 1023.6 | 255.9 | 196.19 | 29.86 | 25.59 | 0.19 | 23 | 1 | 1 | 6 | 8 | 0.2 | 1 | 28 | 172.6 |
| 122 | 960 | 240 | 706.2 | 0 | 234 | 57 | 156 | 0.2 | 23 | 1 | 1 | 1 | 13 | 0.2 | 1 | 90 | 172.5 |
| 123 | 1076.77 | 286.23 | 1090.8 | 0 | 272.6 | 46.2 | 32.3031 | 0.2 | 23 | 1 | 1 | 6 | 12.7 | 0.2 | 1 | 28 | 172.5 |
| 124 | 800 | 267 | 1067 | 0 | 175 | 21.3 | 8 | 0.16 | 23 | 1 | 1 | 6 | 13 | 0.2 | 1 | 90 | 172.3 |
| 125 | 960 | 240 | 787.1 | 0 | 234 | 45 | 2.4 | 0.2 | 23 | 1 | 1 | 6 | 6 | 0.16 | 1 | 28 | 172.1 |
| 126 | 960 | 240 | 798.8 | 0 | 234 | 39 | 9.6 | 0.2 | 23 | 1 | 1 | 5 | 13 | 0.018 | 1 | 28 | 172 |
| 127 | 1009 | 0 | 1456.8 | 0 | 201.8 | 34.2 | 0 | 0.2 | 23 | 1 | 1 | 0 | 0 | 0 | 1 | 90 | 172 |
| 128 | 788 | 197 | 866.8 | 315 | 173 | 14.77 | 19.7 | 0.18 | 23 | 1 | 1 | 6 | 13 | 0.16 | 1 | 14 | 171.9 |
| 129 | 853 | 170.6 | 1023.6 | 255.9 | 196.19 | 29.86 | 51.18 | 0.19 | 23 | 1 | 1 | 6 | 16 | 0.2 | 1 | 14 | 171.6 |
| 130 | 1009 | 0 | 1456.8 | 0 | 201.8 | 34.2 | 0 | 0.2 | 23 | 1 | 1 | 0 | 0 | 0 | 1 | 90 | 171.4 |
| 131 | 850 | 260 | 850 | 212 | 170 | 45 | 156 | 0.15 | 23 | 1 | 1 | 1 | 13 | 0.2 | 1 | 28 | 171.2 |
| 132 | 960 | 240 | 793.9 | 0 | 234 | 42 | 7.2 | 0.2 | 23 | 1 | 1 | 5 | 13 | 0.018 | 1 | 28 | 171.1 |
| 133 | 960 | 240 | 720.7 | 0 | 234 | 57 | 117 | 0.2 | 23 | 1 | 1 | 1 | 13 | 0.2 | 1 | 90 | 170.9 |
| 134 | 1116.7 | 390.85 | 625.35 | 0 | 212.17 | 23.45 | 0 | 0.14 | 23 | 1 | 1 | 0 | 0 | 0 | 1 | 21 | 170.9 |
| 135 | 1116.7 | 390.85 | 625.35 | 0 | 212.17 | 23.45 | 0 | 0.14 | 23 | 1 | 1 | 0 | 0 | 0 | 1 | 28 | 170.9 |
| 136 | 960 | 240 | 767.2 | 0 | 234 | 45 | 78 | 0.2 | 23 | 1 | 1 | 1 | 13 | 0.2 | 1 | 56 | 170.7 |
| 137 | 788 | 197 | 866.8 | 315 | 173 | 14.77 | 15.76 | 0.18 | 23 | 1 | 1 | 6 | 13 | 0.16 | 1 | 21 | 170.7 |
| 138 | 788 | 197 | 866.8 | 315 | 173 | 14.77 | 19.7 | 0.18 | 23 | 1 | 1 | 6 | 6 | 0.16 | 1 | 14 | 170.6 |
| 139 | 960 | 240 | 773.8 | 0 | 234 | 45 | 58.5 | 0.2 | 23 | 1 | 1 | 1 | 13 | 0.2 | 1 | 90 | 170.5 |
| 140 | 850 | 260 | 850 | 212 | 170 | 45 | 156 | 0.15 | 23 | 1 | 1 | 1 | 13 | 0.2 | 1 | 28 | 170.4 |
| 141 | 810 | 90 | 1539 | 0 | 162 | 18 | 0 | 0.18 | 20 | 1 | 1 | 0 | 0 | 0 | 1 | 90 | 170 |
| 142 | 810 | 90 | 1539 | 0 | 162 | 18 | 0 | 0.18 | 20 | 1 | 1 | 0 | 0 | 0 | 1 | 90 | 170 |
| 143 | 1116.7 | 390.85 | 625.35 | 0 | 212.17 | 23.45 | 22.334 | 0.14 | 23 | 1 | 1 | 6 | 13 | 0.2 | 1 | 28 | 169.8 |
| 144 | 960 | 240 | 780.5 | 0 | 234 | 45 | 39 | 0.2 | 23 | 1 | 1 | 1 | 13 | 0.2 | 1 | 90 | 169.4 |
| 145 | 960 | 240 | 764.7 | 0 | 234 | 45 | 78 | 0.2 | 23 | 1 | 1 | 1 | 13 | 0.2 | 1 | 90 | 169.1 |
| 146 | 850 | 212.5 | 935 | 255 | 212.5 | 21.2 | 25.5 | 0.2 | 23 | 1 | 1 | 6 | 12 | 0.2 | 1 | 28 | 169.1 |
| 147 | 998.8 | 176.25 | 991.1 | 0 | 141 | 77.6 | 4.994 | 0.12 | 23 | 1 | 1 | 5 | 13 | 0.018 | 1 | 28 | 169.1 |
| 148 | 1116.7 | 390.85 | 625.35 | 0 | 212.17 | 23.45 | 55.835 | 0.14 | 23 | 1 | 1 | 6 | 13 | 0.2 | 1 | 21 | 168.5 |
| 149 | 853 | 170.6 | 1023.6 | 255.9 | 196.19 | 29.86 | 51.18 | 0.19 | 23 | 1 | 1 | 6 | 8 | 0.2 | 1 | 14 | 168.4 |
| 150 | 853 | 170.6 | 1023.6 | 255.9 | 196.19 | 29.86 | 0 | 0.19 | 23 | 1 | 1 | 0 | 0 | 0 | 1 | 56 | 168.4 |
| 151 | 922 | 230 | 1152 | 0 | 184 | 11.52 | 13.83 | 0.16 | 23 | 1 | 1 | 4 | 12 | 0.018 | 1 | 28 | 168.2 |
| 152 | 1076.77 | 286.23 | 1090.8 | 0 | 272.6 | 46.2 | 32.3031 | 0.2 | 23 | 1 | 1 | 6 | 12.7 | 0.2 | 1 | 28 | 168.1 |
| 153 | 960 | 240 | 771.9 | 0 | 234 | 45 | 58.5 | 0.2 | 23 | 1 | 1 | 1 | 13 | 0.2 | 1 | 90 | 167.9 |
| 154 | 960 | 240 | 787.1 | 0 | 234 | 45 | 19.5 | 0.2 | 23 | 1 | 1 | 1 | 13 | 0.2 | 1 | 90 | 167.8 |
| 155 | 960 | 240 | 779.2 | 0 | 234 | 45 | 39 | 0.2 | 23 | 1 | 1 | 1 | 13 | 0.2 | 1 | 90 | 167.7 |
| 156 | 960 | 240 | 773.8 | 0 | 234 | 45 | 58.5 | 0.2 | 23 | 1 | 1 | 1 | 13 | 0.2 | 1 | 56 | 167.6 |
| 157 | 998.8 | 176.25 | 1010.1 | 0 | 141 | 75.2 | 0 | 0.12 | 23 | 1 | 1 | 0 | 0 | 0 | 1 | 28 | 167.6 |
| 158 | 1116.7 | 390.85 | 625.35 | 0 | 212.17 | 23.45 | 44.668 | 0.14 | 23 | 1 | 1 | 6 | 13 | 0.2 | 1 | 28 | 167.5 |
| 159 | 922 | 230 | 1152 | 0 | 184 | 11.52 | 9.22 | 0.16 | 23 | 1 | 1 | 4 | 12 | 0.018 | 1 | 28 | 167.5 |
| 160 | 853 | 170.6 | 1023.6 | 255.9 | 196.19 | 29.86 | 51.18 | 0.19 | 23 | 1 | 1 | 6 | 12 | 0.2 | 1 | 14 | 167.4 |
| 161 | 853 | 170.6 | 1023.6 | 255.9 | 196.19 | 29.86 | 8.53 | 0.19 | 23 | 1 | 1 | 6 | 16 | 0.2 | 1 | 28 | 167.4 |
| 162 | 788 | 197 | 866.8 | 315 | 173 | 14.77 | 15.76 | 0.18 | 23 | 1 | 1 | 6 | 6 | 0.16 | 1 | 28 | 167.3 |
| 163 | 960 | 240 | 706.2 | 0 | 234 | 57 | 156 | 0.2 | 23 | 1 | 1 | 1 | 13 | 0.2 | 1 | 56 | 166.7 |
| 164 | 800 | 100 | 980 | 200 | 200 | 9.6 | 0 | 0.22 | 20 | 1 | 1 | 0 | 0 | 0 | 1 | 90 | 166.4 |
| 165 | 853 | 170.6 | 1023.6 | 255.9 | 196.19 | 29.86 | 8.53 | 0.19 | 23 | 1 | 1 | 6 | 12 | 0.2 | 1 | 28 | 166.3 |
| 166 | 960 | 240 | 780.5 | 0 | 234 | 45 | 39 | 0.2 | 23 | 1 | 1 | 1 | 13 | 0.2 | 1 | 56 | 166.2 |
| 167 | 729 | 124 | 833 | 397 | 170 | 30 | 156 | 0.2 | 23 | 1 | 1 | 1 | 13 | 0.2 | 1 | 28 | 166.1 |
| 168 | 960 | 240 | 788.9 | 0 | 234 | 45 | 4.8 | 0.2 | 23 | 1 | 1 | 5 | 13 | 0.018 | 1 | 28 | 165.8 |
| 169 | 788 | 197 | 866.8 | 315 | 173 | 14.77 | 15.76 | 0.18 | 23 | 1 | 1 | 6 | 13 | 0.16 | 1 | 14 | 165.6 |
| 170 | 828 | 207 | 911 | 248 | 186.4 | 21 | 16.56 | 0.18 | 23 | 1 | 1 | 4 | 10 | 0.44 | 1 | 28 | 165.6 |
| 171 | 1076.77 | 286.23 | 1090.8 | 0 | 272.6 | 46.2 | 0 | 0.2 | 23 | 1 | 1 | 0 | 0 | 0 | 1 | 28 | 165.6 |
| 172 | 960 | 240 | 720.7 | 0 | 234 | 57 | 117 | 0.2 | 23 | 1 | 1 | 1 | 13 | 0.2 | 1 | 56 | 165.5 |
| 173 | 960 | 240 | 786.4 | 0 | 234 | 45 | 19.5 | 0.2 | 23 | 1 | 1 | 1 | 13 | 0.2 | 1 | 90 | 165.1 |
| 174 | 741 | 185 | 815 | 259 | 185 | 9 | 156 | 0.2 | 30 | 1 | 1 | 1 | 13 | 0.2 | 1 | 90 | 165 |
| 175 | 850 | 212.5 | 935 | 255 | 212.5 | 21.2 | 25.5 | 0.2 | 23 | 1 | 1 | 3 | 15 | 0.6 | 1 | 28 | 165 |
| 176 | 1116.7 | 390.85 | 625.35 | 0 | 212.17 | 23.45 | 44.668 | 0.14 | 23 | 1 | 1 | 6 | 13 | 0.2 | 1 | 21 | 165 |
| 177 | 960 | 240 | 764.7 | 0 | 234 | 45 | 78 | 0.2 | 23 | 1 | 1 | 1 | 13 | 0.2 | 1 | 56 | 164.9 |
| 178 | 960 | 240 | 771.9 | 0 | 234 | 45 | 58.5 | 0.2 | 23 | 1 | 1 | 1 | 13 | 0.2 | 1 | 56 | 164.7 |
| 179 | 863 | 216 | 1079 | 0 | 194.22 | 43.16 | 17.26 | 0.18 | 23 | 1 | 1 | 6 | 13 | 0.2 | 1 | 28 | 164.6 |
| 180 | 1076.77 | 286.23 | 1090.8 | 0 | 272.6 | 46.2 | 0 | 0.2 | 23 | 1 | 1 | 0 | 0 | 0 | 1 | 28 | 164.6 |
| 181 | 998.8 | 176.25 | 998 | 0 | 164.5 | 49.4 | 4.994 | 0.14 | 23 | 1 | 1 | 5 | 13 | 0.018 | 1 | 28 | 164.5 |
| 182 | 998.8 | 176.25 | 954.5 | 0 | 141 | 81.7 | 14.982 | 0.12 | 23 | 1 | 1 | 5 | 13 | 0.018 | 1 | 14 | 164.3 |
| 183 | 853 | 170.6 | 1023.6 | 255.9 | 196.19 | 29.86 | 8.53 | 0.19 | 23 | 1 | 1 | 6 | 8 | 0.2 | 1 | 28 | 164.2 |
| 184 | 936 | 140.4 | 1170 | 0 | 224.64 | 28.08 | 18.72 | 0.21 | 23 | 1 | 1 | 6 | 13 | 0.2 | 1 | 28 | 163.8 |
| 185 | 1116.7 | 390.85 | 625.35 | 0 | 212.17 | 23.45 | 22.334 | 0.14 | 23 | 1 | 1 | 6 | 13 | 0.2 | 1 | 28 | 163.8 |
| 186 | 922 | 230 | 1152 | 0 | 184 | 11.52 | 4.61 | 0.16 | 23 | 1 | 1 | 4 | 12 | 0.018 | 1 | 28 | 163.7 |
| 187 | 922 | 230 | 1152 | 0 | 184 | 11.52 | 16.135 | 0.16 | 23 | 1 | 1 | 4 | 12 | 0.018 | 1 | 28 | 163.4 |
| 188 | 960 | 240 | 779.2 | 0 | 234 | 45 | 39 | 0.2 | 23 | 1 | 1 | 1 | 13 | 0.2 | 1 | 56 | 163.3 |
| 189 | 800 | 200 | 880 | 200 | 200 | 9.6 | 0 | 0.2 | 20 | 1 | 1 | 0 | 0 | 0 | 1 | 90 | 163.3 |
| 190 | 853 | 170.6 | 1023.6 | 255.9 | 196.19 | 29.86 | 25.59 | 0.19 | 23 | 1 | 1 | 6 | 12 | 0.2 | 1 | 14 | 163.2 |
| 191 | 960 | 240 | 787.1 | 0 | 234 | 45 | 19.5 | 0.2 | 23 | 1 | 1 | 1 | 13 | 0.2 | 1 | 56 | 163.1 |
| 192 | 1009 | 0 | 1456.8 | 0 | 201.8 | 34.2 | 0 | 0.2 | 23 | 1 | 1 | 0 | 0 | 0 | 1 | 90 | 163.1 |
| 193 | 960 | 240 | 740.7 | 0 | 234 | 45 | 156 | 0.2 | 23 | 1 | 1 | 1 | 13 | 0.2 | 1 | 28 | 162.8 |
| 194 | 784 | 261 | 1045 | 0 | 171 | 20.9 | 23.52 | 0.16 | 23 | 1 | 1 | 6 | 13 | 0.2 | 1 | 90 | 162.6 |
| 195 | 998.8 | 176.25 | 1013.9 | 0 | 164.5 | 49.4 | 0 | 0.14 | 23 | 1 | 1 | 0 | 0 | 0 | 1 | 28 | 162.2 |
| 196 | 853 | 170.6 | 1023.6 | 255.9 | 196.19 | 29.86 | 25.59 | 0.19 | 23 | 1 | 1 | 6 | 8 | 0.2 | 1 | 14 | 162.1 |
| 197 | 998.8 | 176.25 | 975 | 0 | 141 | 78.7 | 9.988 | 0.12 | 23 | 1 | 1 | 5 | 13 | 0.018 | 1 | 14 | 162 |
| 198 | 900 | 220 | 1005 | 0 | 163 | 40 | 156 | 0.15 | 23 | 1 | 1 | 1 | 13 | 0.2 | 1 | 56 | 161.6 |
| 199 | 792 | 264 | 1056 | 0 | 173 | 21.1 | 156 | 0.16 | 20 | 1 | 1 | 1 | 13 | 0.2 | 1 | 90 | 161.47 |
| 200 | 960 | 240 | 791.3 | 0 | 234 | 45 | 2.4 | 0.2 | 23 | 1 | 1 | 5 | 13 | 0.018 | 1 | 28 | 161.1 |
| 201 | 960 | 240 | 786.4 | 0 | 234 | 45 | 19.5 | 0.2 | 23 | 1 | 1 | 1 | 13 | 0.2 | 1 | 56 | 160.9 |
| 202 | 960 | 240 | 754 | 0 | 234 | 45 | 117 | 0.2 | 23 | 1 | 1 | 1 | 13 | 0.2 | 1 | 28 | 160.6 |
| 203 | 1076.77 | 286.23 | 1090.8 | 0 | 272.6 | 46.2 | 0 | 0.2 | 23 | 1 | 1 | 0 | 0 | 0 | 1 | 28 | 160.6 |
| 204 | 936 | 140.4 | 1170 | 0 | 224.64 | 28.08 | 11.232 | 0.21 | 23 | 1 | 1 | 3 | 12 | 0.038 | 1 | 28 | 160.5 |
| 205 | 1116.7 | 390.85 | 625.35 | 0 | 212.17 | 23.45 | 0 | 0.14 | 23 | 1 | 1 | 0 | 0 | 0 | 1 | 28 | 160.5 |
| 206 | 1116.7 | 390.85 | 625.35 | 0 | 212.17 | 23.45 | 22.334 | 0.14 | 23 | 1 | 1 | 6 | 13 | 0.2 | 1 | 21 | 160.3 |
| 207 | 960 | 240 | 706.2 | 0 | 234 | 57 | 156 | 0.2 | 23 | 1 | 1 | 1 | 13 | 0.2 | 1 | 28 | 160.1 |
| 208 | 630 | 70 | 1788 | 0 | 126 | 18 | 0 | 0.18 | 20 | 1 | 1 | 0 | 0 | 0 | 1 | 90 | 160 |
| 209 | 853 | 170.6 | 1023.6 | 255.9 | 196.19 | 29.86 | 25.59 | 0.19 | 23 | 1 | 1 | 6 | 16 | 0.2 | 1 | 14 | 160 |
| 210 | 1009 | 0 | 1456.8 | 0 | 201.8 | 34.2 | 0 | 0.2 | 23 | 1 | 1 | 0 | 0 | 0 | 1 | 90 | 160 |
| 211 | 1000 | 250 | 1000 | 0 | 200 | 50 | 10 | 0.16 | 23 | 1 | 1 | 4 | 12 | 0.018 | 1 | 28 | 159.6 |
| 212 | 1009 | 0 | 1456.8 | 0 | 201.8 | 34.2 | 0 | 0.2 | 23 | 1 | 1 | 0 | 0 | 0 | 1 | 90 | 159.6 |
| 213 | 800 | 200 | 1080 | 0 | 200 | 14.4 | 0 | 0.2 | 20 | 1 | 1 | 0 | 0 | 0 | 1 | 28 | 159.2 |
| 214 | 788 | 197 | 866.8 | 315 | 173 | 14.77 | 15.76 | 0.18 | 23 | 1 | 1 | 6 | 6 | 0.16 | 1 | 21 | 159.2 |
| 215 | 850 | 212.5 | 935 | 255 | 212.5 | 21.2 | 25.5 | 0.2 | 23 | 1 | 1 | 6 | 12 | 0.2 | 1 | 28 | 159.1 |
| 216 | 922 | 230 | 1152 | 0 | 184 | 11.52 | 1.844 | 0.16 | 23 | 1 | 1 | 4 | 12 | 0.018 | 1 | 28 | 158.9 |
| 217 | 853 | 170.6 | 1023.6 | 255.9 | 196.19 | 29.86 | 0 | 0.19 | 23 | 1 | 1 | 0 | 0 | 0 | 1 | 28 | 158.9 |
| 218 | 998.8 | 176.25 | 962.9 | 0 | 164.5 | 52.9 | 14.982 | 0.14 | 23 | 1 | 1 | 5 | 13 | 0.018 | 1 | 14 | 158.7 |
| 219 | 998.8 | 176.25 | 946.8 | 0 | 164.5 | 54.1 | 19.976 | 0.14 | 23 | 1 | 1 | 5 | 13 | 0.018 | 1 | 14 | 158.4 |
| 220 | 1076.77 | 286.23 | 1090.8 | 0 | 272.6 | 46.2 | 32.3031 | 0.2 | 23 | 1 | 1 | 6 | 12.7 | 0.2 | 1 | 28 | 158.2 |
| 221 | 998.8 | 176.25 | 900.1 | 0 | 164.5 | 62.3 | 29.964 | 0.14 | 23 | 1 | 1 | 5 | 13 | 0.018 | 1 | 14 | 158.2 |
| 222 | 998.8 | 176.25 | 930.6 | 0 | 164.5 | 55.2 | 24.97 | 0.14 | 23 | 1 | 1 | 5 | 13 | 0.018 | 1 | 14 | 158.2 |
| 223 | 960 | 240 | 740.7 | 0 | 234 | 45 | 19.2 | 0.2 | 23 | 1 | 1 | 6 | 6 | 0.16 | 1 | 7 | 157.8 |
| 224 | 960 | 240 | 793.7 | 0 | 234 | 45 | 0 | 0.2 | 23 | 1 | 1 | 0 | 0 | 0 | 1 | 28 | 157.8 |
| 225 | 998.8 | 176.25 | 991.1 | 0 | 141 | 77.6 | 4.994 | 0.12 | 23 | 1 | 1 | 5 | 13 | 0.018 | 1 | 14 | 157.5 |
| 226 | 853 | 170.6 | 1023.6 | 255.9 | 196.19 | 29.86 | 51.18 | 0.19 | 23 | 1 | 1 | 6 | 16 | 0.2 | 1 | 7 | 156.8 |
| 227 | 828 | 207 | 911 | 248 | 186.4 | 21 | 24.84 | 0.18 | 23 | 1 | 1 | 6 | 12 | 0.2 | 1 | 7 | 156.8 |
| 228 | 792 | 264 | 1056 | 0 | 173 | 21.1 | 15.84 | 0.16 | 23 | 1 | 1 | 6 | 13 | 0.2 | 1 | 91 | 156.8 |
| 229 | 853 | 170.6 | 1023.6 | 255.9 | 196.19 | 29.86 | 8.53 | 0.19 | 23 | 1 | 1 | 6 | 12 | 0.2 | 1 | 14 | 156.8 |
| 230 | 922 | 230 | 1152 | 0 | 184 | 11.52 | 0 | 0.16 | 23 | 1 | 1 | 0 | 0 | 0 | 1 | 28 | 156.8 |
| 231 | 792 | 264 | 1056 | 0 | 173 | 21.1 | 15.84 | 0.16 | 23 | 1 | 1 | 6 | 13 | 0.2 | 1 | 91 | 156.5 |
| 232 | 792 | 264 | 1056 | 0 | 173 | 21.1 | 15.84 | 0.16 | 23 | 1 | 1 | 6 | 13 | 0.2 | 1 | 90 | 156.3 |
| 233 | 960 | 240 | 720.7 | 0 | 234 | 57 | 117 | 0.2 | 23 | 1 | 1 | 1 | 13 | 0.2 | 1 | 28 | 156.2 |
| 234 | 960 | 240 | 793.7 | 0 | 234 | 45 | 0 | 0.2 | 23 | 1 | 1 | 0 | 0 | 0 | 1 | 90 | 156.1 |
| 235 | 853 | 170.6 | 1023.6 | 255.9 | 196.19 | 29.86 | 51.18 | 0.19 | 23 | 1 | 1 | 6 | 8 | 0.2 | 1 | 7 | 155.8 |
| 236 | 788 | 197 | 866.8 | 315 | 173 | 14.77 | 19.7 | 0.18 | 23 | 1 | 1 | 6 | 13 | 0.16 | 1 | 7 | 155.8 |
| 237 | 853 | 170.6 | 1023.6 | 255.9 | 196.19 | 29.86 | 8.53 | 0.19 | 23 | 1 | 1 | 6 | 16 | 0.2 | 1 | 14 | 155.8 |
| 238 | 874.9 | 43.7 | 1273.4 | 0 | 202.1 | 45.9 | 195 | 0.22 | 21 | 1 | 1 | 1 | 13 | 0.2 | 1 | 28 | 155.71 |
| 239 | 958.55 | 50.45 | 1231 | 0 | 201.8 | 34.2 | 0 | 0.2 | 21 | 1 | 1 | 0 | 0 | 0 | 1 | 90 | 155.2 |
| 240 | 828 | 207 | 911 | 248 | 186.4 | 21 | 8.28 | 0.18 | 23 | 1 | 1 | 4 | 10 | 0.44 | 1 | 28 | 155.2 |
| 241 | 741 | 185 | 815 | 259 | 185 | 9 | 14.82 | 0.2 | 23 | 1 | 1 | 6 | 13 | 0.2 | 1 | 28 | 154.9 |
| 242 | 960 | 240 | 767.2 | 0 | 234 | 45 | 78 | 0.2 | 23 | 1 | 1 | 1 | 13 | 0.2 | 1 | 28 | 154.8 |
| 243 | 960 | 240 | 764.7 | 0 | 234 | 45 | 78 | 0.2 | 23 | 1 | 1 | 1 | 13 | 0.2 | 1 | 28 | 154.8 |
| 244 | 936 | 140.4 | 1170 | 0 | 224.64 | 28.08 | 7.488 | 0.21 | 23 | 1 | 1 | 3 | 12 | 0.038 | 1 | 28 | 154.8 |
| 245 | 853 | 170.6 | 1023.6 | 255.9 | 196.19 | 29.86 | 51.18 | 0.19 | 23 | 1 | 1 | 6 | 12 | 0.2 | 1 | 7 | 154.7 |
| 246 | 853 | 170.6 | 1023.6 | 255.9 | 196.19 | 29.86 | 8.53 | 0.19 | 23 | 1 | 1 | 6 | 8 | 0.2 | 1 | 14 | 154.7 |
| 247 | 998.8 | 176.25 | 1010.1 | 0 | 141 | 75.2 | 0 | 0.12 | 23 | 1 | 1 | 0 | 0 | 0 | 1 | 14 | 153.3 |
| 248 | 1116.7 | 390.85 | 625.35 | 0 | 212.17 | 23.45 | 44.668 | 0.14 | 23 | 1 | 1 | 6 | 13 | 0.2 | 1 | 7 | 153.2 |
| 249 | 850 | 212.5 | 935 | 255 | 212.5 | 21.2 | 2.55 | 0.2 | 23 | 1 | 1 | 4 | 9 | 0.013 | 1 | 28 | 153.2 |
| 250 | 828 | 207 | 911 | 248 | 186.4 | 21 | 24.84 | 0.18 | 23 | 1 | 1 | 4 | 10 | 0.44 | 1 | 7 | 153 |
| 251 | 788 | 197 | 866.8 | 315 | 173 | 14.77 | 15.76 | 0.18 | 23 | 1 | 1 | 6 | 6 | 0.16 | 1 | 14 | 152.8 |
| 252 | 788 | 197 | 866.8 | 315 | 173 | 14.77 | 19.7 | 0.18 | 23 | 1 | 1 | 6 | 6 | 0.16 | 1 | 7 | 152.6 |
| 253 | 960 | 240 | 773.8 | 0 | 234 | 45 | 58.5 | 0.2 | 23 | 1 | 1 | 1 | 13 | 0.2 | 1 | 28 | 152.5 |
| 254 | 784 | 261 | 1045 | 0 | 171 | 20.9 | 23.52 | 0.16 | 23 | 1 | 1 | 6 | 13 | 0.2 | 1 | 28 | 152 |
| 255 | 998.8 | 176.25 | 981.9 | 0 | 164.5 | 50.5 | 9.988 | 0.14 | 23 | 1 | 1 | 5 | 13 | 0.018 | 1 | 14 | 152 |
| 256 | 960 | 240 | 771.9 | 0 | 234 | 45 | 58.5 | 0.2 | 23 | 1 | 1 | 1 | 13 | 0.2 | 1 | 28 | 151.8 |
| 257 | 1000 | 250 | 1000 | 0 | 200 | 50 | 5 | 0.16 | 23 | 1 | 1 | 4 | 12 | 0.018 | 1 | 28 | 151.8 |
| 258 | 998.8 | 176.25 | 954.5 | 0 | 141 | 81.7 | 14.982 | 0.12 | 23 | 1 | 1 | 5 | 13 | 0.018 | 1 | 7 | 151.7 |
| 259 | 900 | 108 | 1000 | 0 | 161.28 | 18 | 0 | 0.16 | 23 | 1 | 1 | 0 | 0 | 0 | 1 | 28 | 151 |
| 260 | 853 | 170.6 | 1023.6 | 255.9 | 196.19 | 29.86 | 25.59 | 0.19 | 23 | 1 | 1 | 6 | 16 | 0.2 | 1 | 7 | 150.5 |
| 261 | 741 | 185 | 815 | 259 | 185 | 9 | 14.82 | 0.2 | 23 | 1 | 1 | 6 | 13 | 0.2 | 1 | 91 | 150.4 |
| 262 | 936 | 140.4 | 1170 | 0 | 224.64 | 28.08 | 3.744 | 0.21 | 23 | 1 | 1 | 3 | 12 | 0.038 | 1 | 28 | 150.3 |
| 263 | 960 | 240 | 960 | 0 | 207 | 24 | 76.8 | 0.17 | 23 | 1 | 1 | 6 | 13 | 0.18 | 1 | 28 | 150.2 |
| 264 | 788 | 197 | 866.8 | 315 | 173 | 14.77 | 15.76 | 0.18 | 23 | 1 | 1 | 6 | 13 | 0.16 | 1 | 7 | 150.2 |
| 265 | 800 | 267 | 1067 | 0 | 175 | 21.3 | 8 | 0.16 | 23 | 1 | 1 | 6 | 13 | 0.2 | 1 | 28 | 150.2 |
| 266 | 1009 | 0 | 1456.8 | 0 | 201.8 | 34.2 | 0 | 0.2 | 23 | 1 | 1 | 0 | 0 | 0 | 1 | 56 | 150.2 |
| 267 | 792 | 264 | 1056 | 0 | 173 | 21.1 | 156 | 0.16 | 20 | 1 | 1 | 1 | 13 | 0.2 | 1 | 28 | 150.13 |
| 268 | 960 | 240 | 780.5 | 0 | 234 | 45 | 39 | 0.2 | 23 | 1 | 1 | 1 | 13 | 0.2 | 1 | 28 | 150.1 |
| 269 | 960 | 240 | 793.7 | 0 | 234 | 45 | 0 | 0.2 | 23 | 1 | 1 | 0 | 0 | 0 | 1 | 56 | 150.1 |
| 270 | 810 | 90 | 1539 | 0 | 162 | 18 | 0 | 0.18 | 20 | 1 | 1 | 0 | 0 | 0 | 1 | 56 | 150 |
| 271 | 810 | 90 | 1539 | 0 | 162 | 18 | 0 | 0.18 | 20 | 1 | 1 | 0 | 0 | 0 | 1 | 56 | 150 |
| 272 | 850 | 212.5 | 935 | 255 | 212.5 | 21.2 | 0.85 | 0.2 | 23 | 1 | 1 | 4 | 9 | 0.013 | 1 | 28 | 149.8 |
| 273 | 1009 | 0 | 1231 | 0 | 201.8 | 34.2 | 0 | 0.2 | 21 | 1 | 1 | 0 | 0 | 0 | 1 | 90 | 149.7 |
| 274 | 958.55 | 50.45 | 1456.8 | 0 | 201.8 | 34.2 | 0 | 0.2 | 23 | 1 | 1 | 0 | 0 | 0 | 1 | 56 | 149.6 |
| 275 | 853 | 170.6 | 1023.6 | 255.9 | 196.19 | 29.86 | 25.59 | 0.19 | 23 | 1 | 1 | 6 | 8 | 0.2 | 1 | 7 | 149.5 |
| 276 | 853 | 170.6 | 1023.6 | 255.9 | 196.19 | 29.86 | 0 | 0.19 | 23 | 1 | 1 | 0 | 0 | 0 | 1 | 14 | 149.5 |
| 277 | 960 | 240 | 754 | 0 | 234 | 45 | 14.4 | 0.2 | 23 | 1 | 1 | 6 | 6 | 0.16 | 1 | 7 | 149.2 |
| 278 | 960 | 240 | 698.3 | 0 | 234 | 78 | 14.4 | 0.2 | 23 | 1 | 1 | 5 | 13 | 0.018 | 1 | 7 | 149 |
| 279 | 958.55 | 50.45 | 1456.8 | 0 | 201.8 | 34.2 | 0 | 0.2 | 23 | 1 | 1 | 0 | 0 | 0 | 1 | 56 | 149 |
| 280 | 1009 | 0 | 1456.8 | 0 | 201.8 | 34.2 | 0 | 0.2 | 23 | 1 | 1 | 0 | 0 | 0 | 1 | 56 | 149 |
| 281 | 1009 | 0 | 1456.8 | 0 | 201.8 | 34.2 | 0 | 0.2 | 23 | 1 | 1 | 0 | 0 | 0 | 1 | 56 | 148.9 |
| 282 | 800 | 267 | 1067 | 0 | 175 | 21.3 | 78 | 0.16 | 20 | 1 | 1 | 1 | 13 | 0.2 | 1 | 90 | 148.87 |
| 283 | 960 | 240 | 787.1 | 0 | 234 | 45 | 19.5 | 0.2 | 23 | 1 | 1 | 1 | 13 | 0.2 | 1 | 28 | 148.7 |
| 284 | 850 | 212.5 | 935 | 255 | 212.5 | 21.2 | 25.5 | 0.2 | 23 | 1 | 1 | 6 | 12 | 0.2 | 1 | 28 | 148.7 |
| 285 | 960 | 240 | 779.2 | 0 | 234 | 45 | 39 | 0.2 | 23 | 1 | 1 | 1 | 13 | 0.2 | 1 | 28 | 148.6 |
| 286 | 850 | 212.5 | 935 | 255 | 212.5 | 21.2 | 25.5 | 0.2 | 23 | 1 | 1 | 4 | 10 | 0.4 | 1 | 28 | 148.6 |
| 287 | 853 | 170.6 | 1023.6 | 255.9 | 196.19 | 29.86 | 25.59 | 0.19 | 23 | 1 | 1 | 6 | 12 | 0.2 | 1 | 7 | 148.4 |
| 288 | 1000 | 250 | 1000 | 0 | 200 | 50 | 15 | 0.16 | 23 | 1 | 1 | 4 | 12 | 0.018 | 1 | 28 | 148.4 |
| 289 | 958.55 | 50.45 | 1456.8 | 0 | 201.8 | 34.2 | 0 | 0.2 | 23 | 1 | 1 | 0 | 0 | 0 | 1 | 56 | 148.4 |
| 290 | 801 | 89 | 1401.2 | 0 | 178 | 30.2 | 0 | 0.2 | 23 | 1 | 1 | 0 | 0 | 0 | 1 | 90 | 148.2 |
| 291 | 1116.7 | 390.85 | 625.35 | 0 | 212.17 | 23.45 | 22.334 | 0.14 | 23 | 1 | 1 | 6 | 13 | 0.2 | 1 | 28 | 148.1 |
| 292 | 729 | 182 | 1150 | 0 | 182 | 27 | 156 | 0.2 | 20 | 1 | 1 | 1 | 13 | 0.2 | 1 | 28 | 148 |
| 293 | 801 | 89 | 1401.2 | 0 | 178 | 30.2 | 0 | 0.2 | 23 | 1 | 1 | 0 | 0 | 0 | 1 | 90 | 148 |
| 294 | 784 | 261 | 1045 | 0 | 171 | 20.9 | 23.52 | 0.16 | 23 | 1 | 1 | 6 | 13 | 0.2 | 1 | 7 | 147.8 |
| 295 | 801 | 89 | 1401.2 | 0 | 178 | 30.2 | 0 | 0.2 | 23 | 1 | 1 | 0 | 0 | 0 | 1 | 56 | 147.8 |
| 296 | 817 | 204 | 1102 | 0 | 204 | 25 | 156 | 0.2 | 23 | 1 | 1 | 1 | 13 | 0.2 | 1 | 28 | 147.5 |
| 297 | 998.8 | 176.25 | 998 | 0 | 164.5 | 49.4 | 4.994 | 0.14 | 23 | 1 | 1 | 5 | 13 | 0.018 | 1 | 14 | 147.5 |
| 298 | 800 | 100 | 980 | 200 | 200 | 9.6 | 0 | 0.22 | 20 | 1 | 1 | 0 | 0 | 0 | 1 | 28 | 147.3 |
| 299 | 850 | 212.5 | 935 | 255 | 212.5 | 21.2 | 25.5 | 0.2 | 23 | 1 | 1 | 4 | 10 | 0.44 | 1 | 28 | 147.2 |
| 300 | 833 | 208 | 1125 | 0 | 208 | 25 | 0 | 0.2 | 23 | 1 | 1 | 0 | 0 | 0 | 1 | 28 | 147 |
| 301 | 801 | 89 | 1401.2 | 0 | 178 | 30.2 | 0 | 0.2 | 23 | 1 | 1 | 0 | 0 | 0 | 1 | 56 | 147 |
| 302 | 845.5 | 44.5 | 1401.2 | 0 | 178 | 30.2 | 0 | 0.2 | 23 | 1 | 1 | 0 | 0 | 0 | 1 | 90 | 146.8 |
| 303 | 960 | 240 | 767.2 | 0 | 234 | 45 | 9.6 | 0.2 | 23 | 1 | 1 | 6 | 6 | 0.16 | 1 | 7 | 146.7 |
| 304 | 1009 | 0 | 1456.8 | 0 | 201.8 | 34.2 | 0 | 0.2 | 23 | 1 | 1 | 0 | 0 | 0 | 1 | 56 | 146.7 |
| 305 | 801 | 89 | 1401.2 | 0 | 178 | 30.2 | 0 | 0.2 | 23 | 1 | 1 | 0 | 0 | 0 | 1 | 56 | 146.6 |
| 306 | 800 | 200 | 980 | 100 | 200 | 9.6 | 0 | 0.2 | 20 | 1 | 1 | 0 | 0 | 0 | 1 | 28 | 146.5 |
| 307 | 1009 | 0 | 1456.8 | 0 | 201.8 | 34.2 | 0 | 0.2 | 23 | 1 | 1 | 0 | 0 | 0 | 1 | 56 | 146.5 |
| 308 | 1116.7 | 390.85 | 625.35 | 0 | 212.17 | 23.45 | 0 | 0.14 | 23 | 1 | 1 | 0 | 0 | 0 | 1 | 28 | 146.5 |
| 309 | 874.9 | 43.7 | 1273.4 | 0 | 202.1 | 45.9 | 156 | 0.22 | 21 | 1 | 1 | 1 | 13 | 0.2 | 1 | 28 | 146.31 |
| 310 | 998.8 | 176.25 | 975 | 0 | 141 | 78.7 | 9.988 | 0.12 | 23 | 1 | 1 | 5 | 13 | 0.018 | 1 | 7 | 146.1 |
| 311 | 998.8 | 176.25 | 1013.9 | 0 | 164.5 | 49.4 | 0 | 0.14 | 23 | 1 | 1 | 0 | 0 | 0 | 1 | 14 | 146.1 |
| 312 | 630 | 70 | 1788 | 0 | 126 | 18 | 0 | 0.18 | 20 | 1 | 1 | 0 | 0 | 0 | 1 | 56 | 146 |
| 313 | 801 | 89 | 1401.2 | 0 | 178 | 30.2 | 0 | 0.2 | 23 | 1 | 1 | 0 | 0 | 0 | 1 | 90 | 146 |
| 314 | 845.5 | 44.5 | 1401.2 | 0 | 178 | 30.2 | 0 | 0.2 | 23 | 1 | 1 | 0 | 0 | 0 | 1 | 90 | 145.8 |
| 315 | 792 | 264 | 1056 | 0 | 173 | 21.1 | 15.84 | 0.16 | 23 | 1 | 1 | 6 | 13 | 0.2 | 1 | 28 | 145.7 |
| 316 | 845.5 | 44.5 | 1401.2 | 0 | 178 | 30.2 | 0 | 0.2 | 23 | 1 | 1 | 0 | 0 | 0 | 1 | 90 | 145.6 |
| 317 | 825 | 206 | 1114 | 0 | 206 | 25 | 78 | 0.2 | 23 | 1 | 1 | 1 | 13 | 0.2 | 1 | 28 | 145.5 |
| 318 | 1076.77 | 286.23 | 1090.8 | 0 | 272.6 | 46.2 | 0 | 0.2 | 23 | 1 | 1 | 0 | 0 | 0 | 1 | 28 | 145.4 |
| 319 | 845.5 | 44.5 | 1401.2 | 0 | 178 | 30.2 | 0 | 0.2 | 23 | 1 | 1 | 0 | 0 | 0 | 1 | 90 | 145.1 |
| 320 | 1116.7 | 390.85 | 625.35 | 0 | 212.17 | 23.45 | 55.835 | 0.14 | 23 | 1 | 1 | 6 | 13 | 0.2 | 1 | 7 | 145 |
| 321 | 800 | 267 | 1067 | 0 | 175 | 21.3 | 8 | 0.16 | 23 | 1 | 1 | 6 | 13 | 0.2 | 1 | 90 | 144.8 |
| 322 | 845.5 | 44.5 | 1401.2 | 0 | 178 | 30.2 | 0 | 0.2 | 23 | 1 | 1 | 0 | 0 | 0 | 1 | 90 | 144.8 |
| 323 | 863 | 216 | 1079 | 0 | 194.22 | 43.16 | 8.63 | 0.18 | 23 | 1 | 1 | 6 | 13 | 0.2 | 1 | 28 | 144.7 |
| 324 | 828 | 207 | 911 | 248 | 186.4 | 21 | 0 | 0.18 | 23 | 1 | 1 | 0 | 0 | 0 | 1 | 28 | 144.6 |
| 325 | 850 | 212.5 | 935 | 255 | 212.5 | 21.2 | 0 | 0.2 | 23 | 1 | 1 | 0 | 0 | 0 | 1 | 28 | 144.6 |
| 326 | 960 | 240 | 786.4 | 0 | 234 | 45 | 19.5 | 0.2 | 23 | 1 | 1 | 1 | 13 | 0.2 | 1 | 28 | 144.5 |
| 327 | 792 | 264 | 1056 | 0 | 173 | 21.1 | 15.84 | 0.16 | 23 | 1 | 1 | 6 | 13 | 0.2 | 1 | 28 | 144.4 |
| 328 | 853 | 170.6 | 1023.6 | 255.9 | 196.19 | 29.86 | 8.53 | 0.19 | 23 | 1 | 1 | 6 | 16 | 0.2 | 1 | 7 | 144.2 |
| 329 | 1009 | 0 | 1456.8 | 0 | 201.8 | 34.2 | 0 | 0.2 | 23 | 1 | 1 | 0 | 0 | 0 | 1 | 56 | 144.1 |
| 330 | 741 | 185 | 815 | 259 | 185 | 9 | 156 | 0.2 | 30 | 1 | 1 | 1 | 13 | 0.2 | 1 | 7 | 144 |
| 331 | 960 | 240 | 798.8 | 0 | 234 | 39 | 9.6 | 0.2 | 23 | 1 | 1 | 5 | 13 | 0.018 | 1 | 7 | 143.7 |
| 332 | 845.5 | 44.5 | 1401.2 | 0 | 178 | 30.2 | 0 | 0.2 | 23 | 1 | 1 | 0 | 0 | 0 | 1 | 90 | 143.7 |
| 333 | 792 | 264 | 1056 | 0 | 173 | 21.1 | 15.84 | 0.16 | 23 | 1 | 1 | 6 | 13 | 0.2 | 1 | 28 | 143.6 |
| 334 | 845.5 | 44.5 | 1401.2 | 0 | 178 | 30.2 | 0 | 0.2 | 23 | 1 | 1 | 0 | 0 | 0 | 1 | 56 | 143.6 |
| 335 | 960 | 240 | 773.8 | 0 | 234 | 45 | 7.2 | 0.2 | 23 | 1 | 1 | 6 | 6 | 0.16 | 1 | 7 | 143.5 |
| 336 | 998.8 | 176.25 | 962.9 | 0 | 164.5 | 52.9 | 14.982 | 0.14 | 23 | 1 | 1 | 5 | 13 | 0.018 | 1 | 7 | 143.5 |
| 337 | 845.5 | 44.5 | 1401.2 | 0 | 178 | 30.2 | 0 | 0.2 | 23 | 1 | 1 | 0 | 0 | 0 | 1 | 56 | 143.5 |
| 338 | 1116.7 | 390.85 | 625.35 | 0 | 212.17 | 23.45 | 0 | 0.14 | 23 | 1 | 1 | 0 | 0 | 0 | 1 | 28 | 143.5 |
| 339 | 998.8 | 176.25 | 930.6 | 0 | 164.5 | 55.2 | 24.97 | 0.14 | 23 | 1 | 1 | 5 | 13 | 0.018 | 1 | 7 | 143.2 |
| 340 | 998.8 | 176.25 | 946.8 | 0 | 164.5 | 54.1 | 19.976 | 0.14 | 23 | 1 | 1 | 5 | 13 | 0.018 | 1 | 7 | 143.2 |
| 341 | 900 | 220 | 1005 | 0 | 163 | 40 | 156 | 0.15 | 23 | 1 | 1 | 1 | 13 | 0.2 | 1 | 28 | 143 |
| 342 | 998.8 | 176.25 | 900.1 | 0 | 164.5 | 62.3 | 29.964 | 0.14 | 23 | 1 | 1 | 5 | 13 | 0.018 | 1 | 7 | 143 |
| 343 | 850 | 212.5 | 935 | 255 | 212.5 | 21.2 | 17 | 0.2 | 23 | 1 | 1 | 4 | 10 | 0.44 | 1 | 28 | 143 |
| 344 | 845.5 | 44.5 | 1401.2 | 0 | 178 | 30.2 | 0 | 0.2 | 23 | 1 | 1 | 0 | 0 | 0 | 1 | 56 | 143 |
| 345 | 792 | 264 | 1056 | 0 | 173 | 21.1 | 15.84 | 0.16 | 23 | 1 | 1 | 6 | 13 | 0.2 | 1 | 7 | 142.8 |
| 346 | 1116.7 | 390.85 | 625.35 | 0 | 212.17 | 23.45 | 33.501 | 0.14 | 23 | 1 | 1 | 6 | 13 | 0.2 | 1 | 21 | 142.6 |
| 347 | 998.8 | 176.25 | 991.1 | 0 | 141 | 77.6 | 4.994 | 0.12 | 23 | 1 | 1 | 5 | 13 | 0.018 | 1 | 7 | 142.4 |
| 348 | 863 | 216 | 923 | 0 | 177 | 32.37 | 156 | 0.16 | 20 | 1 | 1 | 1 | 13 | 0.2 | 1 | 28 | 142.1 |
| 349 | 853 | 170.6 | 1023.6 | 255.9 | 196.19 | 29.86 | 8.53 | 0.19 | 23 | 1 | 1 | 6 | 8 | 0.2 | 1 | 7 | 142.1 |
| 350 | 853 | 170.6 | 1023.6 | 255.9 | 196.19 | 29.86 | 8.53 | 0.19 | 23 | 1 | 1 | 6 | 12 | 0.2 | 1 | 7 | 142.1 |
| 351 | 850 | 212.5 | 935 | 255 | 212.5 | 21.2 | 4.25 | 0.2 | 23 | 1 | 1 | 4 | 9 | 0.013 | 1 | 28 | 141.7 |
| 352 | 756.5 | 133.5 | 1401.2 | 0 | 178 | 30.2 | 0 | 0.2 | 23 | 1 | 1 | 0 | 0 | 0 | 1 | 90 | 141.6 |
| 353 | 1116.7 | 390.85 | 625.35 | 0 | 212.17 | 23.45 | 33.501 | 0.14 | 23 | 1 | 1 | 6 | 13 | 0.2 | 1 | 7 | 141.4 |
| 354 | 800 | 200 | 880 | 200 | 200 | 9.6 | 0 | 0.2 | 20 | 1 | 1 | 0 | 0 | 0 | 1 | 28 | 141.3 |
| 355 | 800 | 100 | 980 | 200 | 200 | 9.6 | 0 | 0.22 | 20 | 1 | 1 | 0 | 0 | 0 | 1 | 90 | 141.1 |
| 356 | 1116.7 | 390.85 | 625.35 | 0 | 212.17 | 23.45 | 22.334 | 0.14 | 23 | 1 | 1 | 6 | 13 | 0.2 | 1 | 28 | 141.1 |
| 357 | 960 | 240 | 780.5 | 0 | 234 | 45 | 4.8 | 0.2 | 23 | 1 | 1 | 6 | 6 | 0.16 | 1 | 7 | 140.6 |
| 358 | 960 | 240 | 793.9 | 0 | 234 | 42 | 7.2 | 0.2 | 23 | 1 | 1 | 5 | 13 | 0.018 | 1 | 7 | 140.6 |
| 359 | 1116.7 | 390.85 | 625.35 | 0 | 212.17 | 23.45 | 22.334 | 0.14 | 23 | 1 | 1 | 6 | 13 | 0.2 | 1 | 28 | 140.5 |
| 360 | 1116.7 | 390.85 | 625.35 | 0 | 212.17 | 23.45 | 0 | 0.14 | 23 | 1 | 1 | 0 | 0 | 0 | 1 | 28 | 140.4 |
| 361 | 828 | 207 | 911 | 248 | 186.4 | 21 | 16.56 | 0.18 | 23 | 1 | 1 | 4 | 10 | 0.44 | 1 | 7 | 140.1 |
| 362 | 936 | 140.4 | 1170 | 0 | 224.64 | 28.08 | 0 | 0.21 | 23 | 1 | 1 | 0 | 0 | 0 | 1 | 28 | 140.1 |
| 363 | 1000 | 250 | 1000 | 0 | 200 | 50 | 15 | 0.16 | 23 | 1 | 1 | 6 | 12 | 0.2 | 1 | 28 | 140 |
| 364 | 756.5 | 133.5 | 1401.2 | 0 | 178 | 30.2 | 0 | 0.2 | 23 | 1 | 1 | 0 | 0 | 0 | 1 | 56 | 139.4 |
| 365 | 1009 | 0 | 1231 | 0 | 201.8 | 34.2 | 0 | 0.2 | 21 | 1 | 1 | 0 | 0 | 0 | 1 | 90 | 139.3 |
| 366 | 850 | 212.5 | 935 | 255 | 212.5 | 21.2 | 25.5 | 0.2 | 23 | 1 | 1 | 4 | 10 | 0.44 | 1 | 28 | 139.1 |
| 367 | 890 | 0 | 1401.2 | 0 | 178 | 30.2 | 0 | 0.2 | 23 | 1 | 1 | 0 | 0 | 0 | 1 | 90 | 139.1 |
| 368 | 850 | 212.5 | 935 | 255 | 212.5 | 21.2 | 2.55 | 0.2 | 23 | 1 | 1 | 3 | 9 | 0.017 | 1 | 28 | 138.9 |
| 369 | 801 | 89 | 1401.2 | 0 | 178 | 30.2 | 0 | 0.2 | 23 | 1 | 1 | 0 | 0 | 0 | 1 | 28 | 138.9 |
| 370 | 845.5 | 44.5 | 1401.2 | 0 | 178 | 30.2 | 0 | 0.2 | 23 | 1 | 1 | 0 | 0 | 0 | 1 | 56 | 138.9 |
| 371 | 756.5 | 133.5 | 1401.2 | 0 | 178 | 30.2 | 0 | 0.2 | 23 | 1 | 1 | 0 | 0 | 0 | 1 | 90 | 138.9 |
| 372 | 845.5 | 44.5 | 1401.2 | 0 | 178 | 30.2 | 0 | 0.2 | 23 | 1 | 1 | 0 | 0 | 0 | 1 | 56 | 138.8 |
| 373 | 950 | 255 | 873 | 0 | 189 | 31 | 0 | 0.16 | 23 | 1 | 1 | 0 | 0 | 0 | 1 | 90 | 138.7 |
| 374 | 756.5 | 133.5 | 1401.2 | 0 | 178 | 30.2 | 0 | 0.2 | 23 | 1 | 1 | 0 | 0 | 0 | 1 | 56 | 138.7 |
| 375 | 845.5 | 44.5 | 1401.2 | 0 | 178 | 30.2 | 0 | 0.2 | 23 | 1 | 1 | 0 | 0 | 0 | 1 | 56 | 138.4 |
| 376 | 756.5 | 133.5 | 1401.2 | 0 | 178 | 30.2 | 0 | 0.2 | 23 | 1 | 1 | 0 | 0 | 0 | 1 | 90 | 138.4 |
| 377 | 756.5 | 133.5 | 1401.2 | 0 | 178 | 30.2 | 0 | 0.2 | 23 | 1 | 1 | 0 | 0 | 0 | 1 | 56 | 138 |
| 378 | 998.8 | 176.25 | 1010.1 | 0 | 141 | 75.2 | 0 | 0.12 | 23 | 1 | 1 | 0 | 0 | 0 | 1 | 7 | 137.9 |
| 379 | 853 | 170.6 | 1023.6 | 255.9 | 196.19 | 29.86 | 0 | 0.19 | 23 | 1 | 1 | 0 | 0 | 0 | 1 | 7 | 137.9 |
| 380 | 712 | 178 | 1401.2 | 0 | 178 | 30.2 | 0 | 0.2 | 23 | 1 | 1 | 0 | 0 | 0 | 1 | 90 | 137.9 |
| 381 | 960 | 240 | 787.1 | 0 | 234 | 45 | 2.4 | 0.2 | 23 | 1 | 1 | 6 | 6 | 0.16 | 1 | 7 | 137.7 |
| 382 | 998.8 | 176.25 | 981.9 | 0 | 164.5 | 50.5 | 9.988 | 0.14 | 23 | 1 | 1 | 5 | 13 | 0.018 | 1 | 7 | 137.6 |
| 383 | 960 | 240 | 788.9 | 0 | 234 | 45 | 4.8 | 0.2 | 23 | 1 | 1 | 5 | 13 | 0.018 | 1 | 7 | 137.4 |
| 384 | 801 | 89 | 1401.2 | 0 | 178 | 30.2 | 0 | 0.2 | 23 | 1 | 1 | 0 | 0 | 0 | 1 | 28 | 137.4 |
| 385 | 850 | 212.5 | 935 | 255 | 212.5 | 21.2 | 5.95 | 0.2 | 23 | 1 | 1 | 4 | 9 | 0.013 | 1 | 28 | 137.1 |
| 386 | 810 | 90 | 1539 | 0 | 162 | 18 | 0 | 0.18 | 20 | 1 | 1 | 0 | 0 | 0 | 1 | 28 | 137 |
| 387 | 810 | 90 | 1539 | 0 | 162 | 18 | 0 | 0.18 | 20 | 1 | 1 | 0 | 0 | 0 | 1 | 28 | 137 |
| 388 | 756.5 | 133.5 | 1401.2 | 0 | 178 | 30.2 | 0 | 0.2 | 23 | 1 | 1 | 0 | 0 | 0 | 1 | 28 | 136.9 |
| 389 | 712 | 178 | 1401.2 | 0 | 178 | 30.2 | 0 | 0.2 | 23 | 1 | 1 | 0 | 0 | 0 | 1 | 90 | 136.8 |
| 390 | 960 | 240 | 793.7 | 0 | 234 | 45 | 0 | 0.2 | 23 | 1 | 1 | 0 | 0 | 0 | 1 | 28 | 136.6 |
| 391 | 960 | 240 | 740.7 | 0 | 234 | 45 | 156 | 0.2 | 23 | 1 | 1 | 1 | 13 | 0.2 | 1 | 7 | 136.6 |
| 392 | 828 | 207 | 911 | 248 | 186.4 | 21 | 24.84 | 0.18 | 23 | 1 | 1 | 6 | 12 | 0.2 | 1 | 3 | 136.6 |
| 393 | 960 | 240 | 960 | 0 | 260 | 24 | 76.8 | 0.22 | 23 | 1 | 1 | 6 | 13 | 0.18 | 1 | 28 | 136.4 |
| 394 | 845.5 | 44.5 | 1401.2 | 0 | 178 | 30.2 | 0 | 0.2 | 23 | 1 | 1 | 0 | 0 | 0 | 1 | 28 | 136.4 |
| 395 | 712 | 178 | 1401.2 | 0 | 178 | 30.2 | 0 | 0.2 | 23 | 1 | 1 | 0 | 0 | 0 | 1 | 56 | 136.4 |
| 396 | 756.5 | 133.5 | 1401.2 | 0 | 178 | 30.2 | 0 | 0.2 | 23 | 1 | 1 | 0 | 0 | 0 | 1 | 28 | 136.3 |
| 397 | 845.5 | 44.5 | 1401.2 | 0 | 178 | 30.2 | 0 | 0.2 | 23 | 1 | 1 | 0 | 0 | 0 | 1 | 28 | 136.3 |
| 398 | 712 | 178 | 1401.2 | 0 | 178 | 30.2 | 0 | 0.2 | 23 | 1 | 1 | 0 | 0 | 0 | 1 | 90 | 136.3 |
| 399 | 960 | 240 | 960 | 0 | 228 | 24 | 76.8 | 0.19 | 23 | 1 | 1 | 6 | 13 | 0.18 | 1 | 28 | 136.1 |
| 400 | 890 | 0 | 1401.2 | 0 | 178 | 30.2 | 0 | 0.2 | 23 | 1 | 1 | 0 | 0 | 0 | 1 | 90 | 136.1 |
| 401 | 828 | 207 | 911 | 248 | 186.4 | 21 | 24.84 | 0.18 | 23 | 1 | 1 | 4 | 10 | 0.44 | 1 | 3 | 136 |
| 402 | 845.5 | 44.5 | 1401.2 | 0 | 178 | 30.2 | 0 | 0.2 | 23 | 1 | 1 | 0 | 0 | 0 | 1 | 28 | 135.8 |
| 403 | 833 | 167 | 836 | 363 | 184 | 24 | 1.666 | 0.18 | 23 | 1 | 1 | 3 | 20 | 0.034 | 1 | 28 | 135.7 |
| 404 | 756.5 | 133.5 | 1401.2 | 0 | 178 | 30.2 | 0 | 0.2 | 23 | 1 | 1 | 0 | 0 | 0 | 1 | 28 | 135.7 |
| 405 | 833 | 167 | 836 | 363 | 184 | 24 | 1.666 | 0.18 | 23 | 1 | 1 | 3 | 20 | 0.034 | 1 | 28 | 135.6 |
| 406 | 950 | 255 | 873 | 0 | 189 | 31 | 23.75 | 0.16 | 23 | 1 | 1 | 6 | 13 | 0.2 | 1 | 90 | 135.6 |
| 407 | 1116.7 | 390.85 | 625.35 | 0 | 212.17 | 23.45 | 22.334 | 0.14 | 23 | 1 | 1 | 6 | 13 | 0.2 | 1 | 7 | 135.5 |
| 408 | 712 | 178 | 1401.2 | 0 | 178 | 30.2 | 0 | 0.2 | 23 | 1 | 1 | 0 | 0 | 0 | 1 | 56 | 135.5 |
| 409 | 1076.77 | 286.23 | 1090.8 | 0 | 272.6 | 46.2 | 32.3031 | 0.2 | 23 | 1 | 1 | 6 | 12.7 | 0.2 | 1 | 28 | 135.1 |
| 410 | 828 | 207 | 911 | 248 | 186.4 | 21 | 8.28 | 0.18 | 23 | 1 | 1 | 4 | 10 | 0.44 | 1 | 7 | 135.1 |
| 411 | 875 | 150 | 1100 | 0 | 180 | 35 | 8.75 | 0.18 | 23 | 1 | 1 | 4 | 24 | 0.023 | 1 | 28 | 135.1 |
| 412 | 630 | 70 | 1788 | 0 | 126 | 18 | 0 | 0.18 | 20 | 1 | 1 | 0 | 0 | 0 | 1 | 28 | 135 |
| 413 | 1009 | 0 | 1456.8 | 0 | 201.8 | 34.2 | 0 | 0.2 | 23 | 1 | 1 | 0 | 0 | 0 | 1 | 28 | 135 |
| 414 | 875 | 150 | 1100 | 0 | 180 | 35 | 6.5625 | 0.18 | 23 | 1 | 1 | 4 | 24 | 0.023 | 1 | 28 | 134.9 |
| 415 | 845.5 | 44.5 | 1401.2 | 0 | 178 | 30.2 | 0 | 0.2 | 23 | 1 | 1 | 0 | 0 | 0 | 1 | 28 | 134.8 |
| 416 | 788 | 197 | 866.8 | 315 | 173 | 14.77 | 0 | 0.18 | 23 | 1 | 1 | 0 | 0 | 0 | 1 | 21 | 134.7 |
| 417 | 788 | 197 | 866.8 | 315 | 173 | 14.77 | 0 | 0.18 | 23 | 1 | 1 | 0 | 0 | 0 | 1 | 28 | 134.7 |
| 418 | 801 | 89 | 1401.2 | 0 | 178 | 30.2 | 0 | 0.2 | 23 | 1 | 1 | 0 | 0 | 0 | 1 | 28 | 134.6 |
| 419 | 958.55 | 50.45 | 1456.8 | 0 | 201.8 | 34.2 | 0 | 0.2 | 23 | 1 | 1 | 0 | 0 | 0 | 1 | 28 | 134.2 |
| 420 | 712 | 178 | 1401.2 | 0 | 178 | 30.2 | 0 | 0.2 | 23 | 1 | 1 | 0 | 0 | 0 | 1 | 56 | 134.2 |
| 421 | 890 | 0 | 1401.2 | 0 | 178 | 30.2 | 0 | 0.2 | 23 | 1 | 1 | 0 | 0 | 0 | 1 | 90 | 134.1 |
| 422 | 960 | 240 | 791.3 | 0 | 234 | 45 | 2.4 | 0.2 | 23 | 1 | 1 | 5 | 13 | 0.018 | 1 | 7 | 134 |
| 423 | 845.5 | 44.5 | 1401.2 | 0 | 178 | 30.2 | 0 | 0.2 | 23 | 1 | 1 | 0 | 0 | 0 | 1 | 28 | 134 |
| 424 | 863 | 216 | 1079 | 0 | 194.22 | 43.16 | 0 | 0.18 | 23 | 1 | 1 | 0 | 0 | 0 | 1 | 28 | 133.6 |
| 425 | 890 | 0 | 1401.2 | 0 | 178 | 30.2 | 0 | 0.2 | 23 | 1 | 1 | 0 | 0 | 0 | 1 | 90 | 133.1 |
| 426 | 900 | 220 | 1005 | 0 | 163 | 40 | 156 | 0.15 | 23 | 1 | 1 | 1 | 13 | 0.2 | 1 | 14 | 132.9 |
| 427 | 1009 | 0 | 1456.8 | 0 | 201.8 | 34.2 | 0 | 0.2 | 23 | 1 | 1 | 0 | 0 | 0 | 1 | 28 | 132.9 |
| 428 | 958.55 | 50.45 | 1456.8 | 0 | 201.8 | 34.2 | 0 | 0.2 | 23 | 1 | 1 | 0 | 0 | 0 | 1 | 28 | 132.8 |
| 429 | 958.55 | 50.45 | 1456.8 | 0 | 201.8 | 34.2 | 0 | 0.2 | 23 | 1 | 1 | 0 | 0 | 0 | 1 | 28 | 132.8 |
| 430 | 845.5 | 44.5 | 1401.2 | 0 | 178 | 30.2 | 0 | 0.2 | 23 | 1 | 1 | 0 | 0 | 0 | 1 | 28 | 132.8 |
| 431 | 960 | 240 | 960 | 0 | 286 | 24 | 76.8 | 0.24 | 23 | 1 | 1 | 6 | 13 | 0.18 | 1 | 28 | 132.6 |
| 432 | 850 | 150 | 1224 | 0 | 180 | 11.99 | 0 | 0.18 | 23 | 1 | 1 | 0 | 0 | 0 | 1 | 28 | 132.59 |
| 433 | 950 | 255 | 873 | 0 | 189 | 31 | 0 | 0.16 | 23 | 1 | 1 | 0 | 0 | 0 | 1 | 28 | 132.5 |
| 434 | 1009 | 0 | 1456.8 | 0 | 201.8 | 34.2 | 0 | 0.2 | 23 | 1 | 1 | 0 | 0 | 0 | 1 | 28 | 132.5 |
| 435 | 960 | 240 | 706.2 | 0 | 234 | 57 | 156 | 0.2 | 23 | 1 | 1 | 1 | 13 | 0.2 | 1 | 7 | 132.4 |
| 436 | 788 | 197 | 866.8 | 315 | 173 | 14.77 | 15.76 | 0.18 | 23 | 1 | 1 | 6 | 6 | 0.16 | 1 | 7 | 132.4 |
| 437 | 874.9 | 43.7 | 1273.4 | 0 | 202.1 | 45.9 | 117 | 0.22 | 21 | 1 | 1 | 1 | 13 | 0.2 | 1 | 28 | 132.22 |
| 438 | 850 | 212.5 | 935 | 255 | 212.5 | 21.2 | 17 | 0.2 | 23 | 1 | 1 | 4 | 10 | 0.44 | 1 | 28 | 132.1 |
| 439 | 890 | 0 | 1401.2 | 0 | 178 | 30.2 | 0 | 0.2 | 23 | 1 | 1 | 0 | 0 | 0 | 1 | 56 | 132.1 |
| 440 | 890 | 0 | 1401.2 | 0 | 178 | 30.2 | 0 | 0.2 | 23 | 1 | 1 | 0 | 0 | 0 | 1 | 28 | 131.8 |
| 441 | 1009 | 0 | 1456.8 | 0 | 201.8 | 34.2 | 0 | 0.2 | 23 | 1 | 1 | 0 | 0 | 0 | 1 | 28 | 131.7 |
| 442 | 1009 | 0 | 1456.8 | 0 | 201.8 | 34.2 | 0 | 0.2 | 23 | 1 | 1 | 0 | 0 | 0 | 1 | 28 | 131.7 |
| 443 | 998.8 | 176.25 | 998 | 0 | 164.5 | 49.4 | 4.994 | 0.14 | 23 | 1 | 1 | 5 | 13 | 0.018 | 1 | 7 | 131.6 |
| 444 | 890 | 0 | 1401.2 | 0 | 178 | 30.2 | 0 | 0.2 | 23 | 1 | 1 | 0 | 0 | 0 | 1 | 56 | 131.4 |
| 445 | 890 | 0 | 1401.2 | 0 | 178 | 30.2 | 0 | 0.2 | 23 | 1 | 1 | 0 | 0 | 0 | 1 | 90 | 131.2 |
| 446 | 850 | 212.5 | 935 | 255 | 212.5 | 21.2 | 25.5 | 0.2 | 23 | 1 | 1 | 4 | 10 | 0.44 | 1 | 28 | 131.1 |
| 447 | 890 | 0 | 1401.2 | 0 | 178 | 30.2 | 0 | 0.2 | 23 | 1 | 1 | 0 | 0 | 0 | 1 | 90 | 131.1 |
| 448 | 950 | 255 | 873 | 0 | 189 | 31 | 23.75 | 0.16 | 23 | 1 | 1 | 6 | 13 | 0.2 | 1 | 28 | 130.8 |
| 449 | 875 | 150 | 1100 | 0 | 180 | 35 | 6.5625 | 0.18 | 23 | 1 | 1 | 4 | 24 | 0.023 | 1 | 28 | 130.8 |
| 450 | 890 | 0 | 1401.2 | 0 | 178 | 30.2 | 0 | 0.2 | 23 | 1 | 1 | 0 | 0 | 0 | 1 | 56 | 130.7 |
| 451 | 890 | 222 | 799 | 0 | 222 | 29.7 | 117 | 0.2 | 23 | 1 | 1 | 1 | 13 | 0.2 | 1 | 28 | 130.3 |
| 452 | 720 | 80 | 1422.9 | 0 | 160 | 29.6 | 0 | 0.2 | 23 | 1 | 1 | 0 | 0 | 0 | 1 | 90 | 130.3 |
| 453 | 890 | 0 | 1401.2 | 0 | 178 | 30.2 | 0 | 0.2 | 23 | 1 | 1 | 0 | 0 | 0 | 1 | 28 | 130.3 |
| 454 | 890 | 0 | 1401.2 | 0 | 178 | 30.2 | 0 | 0.2 | 23 | 1 | 1 | 0 | 0 | 0 | 1 | 28 | 130.2 |
| 455 | 875 | 150 | 1100 | 0 | 180 | 35 | 8.75 | 0.18 | 23 | 1 | 1 | 4 | 24 | 0.023 | 1 | 28 | 130.1 |
| 456 | 960 | 240 | 720.7 | 0 | 234 | 57 | 117 | 0.2 | 23 | 1 | 1 | 1 | 13 | 0.2 | 1 | 7 | 130 |
| 457 | 800 | 267 | 1067 | 0 | 175 | 21.3 | 78 | 0.16 | 20 | 1 | 1 | 1 | 13 | 0.2 | 1 | 28 | 129.96 |
| 458 | 850 | 150 | 1173 | 0 | 200 | 14.03 | 0 | 0.2 | 23 | 1 | 1 | 0 | 0 | 0 | 1 | 28 | 129.95 |
| 459 | 875 | 150 | 1100 | 0 | 180 | 35 | 6.5625 | 0.18 | 23 | 1 | 1 | 4 | 24 | 0.023 | 1 | 28 | 129.8 |
| 460 | 1009 | 0 | 1456.8 | 0 | 201.8 | 34.2 | 0 | 0.2 | 23 | 1 | 1 | 0 | 0 | 0 | 1 | 28 | 129.7 |
| 461 | 1116.7 | 390.85 | 625.35 | 0 | 212.17 | 23.45 | 44.668 | 0.14 | 23 | 1 | 1 | 6 | 13 | 0.2 | 1 | 28 | 129.6 |
| 462 | 1000 | 250 | 1000 | 0 | 200 | 50 | 10 | 0.16 | 23 | 1 | 1 | 6 | 12 | 0.2 | 1 | 28 | 129.5 |
| 463 | 712 | 178 | 1401.2 | 0 | 178 | 30.2 | 0 | 0.2 | 23 | 1 | 1 | 0 | 0 | 0 | 1 | 28 | 129.5 |
| 464 | 1009 | 0 | 1231 | 0 | 201.8 | 34.2 | 0 | 0.2 | 21 | 1 | 1 | 0 | 0 | 0 | 1 | 56 | 129.3 |
| 465 | 960 | 240 | 754 | 0 | 234 | 45 | 117 | 0.2 | 23 | 1 | 1 | 1 | 13 | 0.2 | 1 | 7 | 129.2 |
| 466 | 958.55 | 50.45 | 1231 | 0 | 201.8 | 34.2 | 0 | 0.2 | 21 | 1 | 1 | 0 | 0 | 0 | 1 | 56 | 129 |
| 467 | 828 | 207 | 911 | 248 | 186.4 | 21 | 16.56 | 0.18 | 23 | 1 | 1 | 4 | 10 | 0.44 | 1 | 3 | 129 |
| 468 | 998.8 | 176.25 | 1013.9 | 0 | 164.5 | 49.4 | 0 | 0.14 | 23 | 1 | 1 | 0 | 0 | 0 | 1 | 7 | 129 |
| 469 | 850.34 | 149.66 | 1224.49 | 0 | 180 | 11.99 | 0 | 0.18 | 23 | 1 | 1 | 0 | 0 | 0 | 1 | 7 | 128.9 |
| 470 | 1076.77 | 286.23 | 1090.8 | 0 | 272.6 | 46.2 | 0 | 0.2 | 23 | 1 | 1 | 0 | 0 | 0 | 1 | 28 | 128.7 |
| 471 | 720 | 180 | 1353.6 | 0 | 162 | 10.8 | 0 | 0.18 | 23 | 1 | 1 | 0 | 0 | 0 | 1 | 28 | 128.2 |
| 472 | 850 | 150 | 1173 | 0 | 200 | 11.99 | 0 | 0.2 | 23 | 1 | 1 | 0 | 0 | 0 | 1 | 28 | 128.1 |
| 473 | 800 | 267 | 1067 | 0 | 175 | 21.3 | 8 | 0.16 | 23 | 1 | 1 | 6 | 13 | 0.2 | 1 | 7 | 128.1 |
| 474 | 801 | 89 | 1231 | 0 | 178 | 30.2 | 0 | 0.2 | 21 | 1 | 1 | 0 | 0 | 0 | 1 | 90 | 127.6 |
| 475 | 712 | 178 | 1401.2 | 0 | 178 | 30.2 | 0 | 0.2 | 23 | 1 | 1 | 0 | 0 | 0 | 1 | 28 | 127.6 |
| 476 | 845.5 | 44.5 | 1401.2 | 0 | 178 | 30.2 | 0 | 0.2 | 23 | 1 | 1 | 0 | 0 | 0 | 1 | 7 | 127.5 |
| 477 | 712 | 178 | 1401.2 | 0 | 178 | 30.2 | 0 | 0.2 | 23 | 1 | 1 | 0 | 0 | 0 | 1 | 28 | 127.5 |
| 478 | 801 | 89 | 1231 | 0 | 178 | 30.2 | 0 | 0.2 | 21 | 1 | 1 | 0 | 0 | 0 | 1 | 56 | 127.4 |
| 479 | 960 | 240 | 764.7 | 0 | 234 | 45 | 78 | 0.2 | 23 | 1 | 1 | 1 | 13 | 0.2 | 1 | 7 | 127.4 |
| 480 | 850 | 212.5 | 935 | 255 | 212.5 | 21.2 | 8.5 | 0.2 | 23 | 1 | 1 | 4 | 10 | 0.44 | 1 | 28 | 127.4 |
| 481 | 720 | 180 | 1296 | 0 | 180 | 12.6 | 0 | 0.2 | 23 | 1 | 1 | 0 | 0 | 0 | 1 | 28 | 127.32 |
| 482 | 850 | 150 | 1181.5 | 0 | 200 | 10.03 | 0 | 0.2 | 23 | 1 | 1 | 0 | 0 | 0 | 1 | 28 | 127.32 |
| 483 | 960 | 240 | 767.2 | 0 | 234 | 45 | 78 | 0.2 | 23 | 1 | 1 | 1 | 13 | 0.2 | 1 | 7 | 126.9 |
| 484 | 890 | 0 | 1401.2 | 0 | 178 | 30.2 | 0 | 0.2 | 23 | 1 | 1 | 0 | 0 | 0 | 1 | 56 | 126.8 |
| 485 | 800 | 200 | 1160 | 0 | 200 | 12 | 0 | 0.2 | 23 | 1 | 1 | 0 | 0 | 0 | 1 | 28 | 126.6 |
| 486 | 800 | 267 | 1067 | 0 | 175 | 21.3 | 8 | 0.16 | 23 | 1 | 1 | 6 | 13 | 0.2 | 1 | 28 | 126.6 |
| 487 | 809 | 270 | 1079 | 0 | 177 | 21.6 | 0 | 0.16 | 23 | 1 | 1 | 0 | 0 | 0 | 1 | 90 | 126.5 |
| 488 | 1009 | 0 | 1231 | 0 | 201.8 | 34.2 | 0 | 0.2 | 21 | 1 | 1 | 0 | 0 | 0 | 1 | 56 | 126.2 |
| 489 | 845.5 | 44.5 | 1231 | 0 | 178 | 30.2 | 0 | 0.2 | 21 | 1 | 1 | 0 | 0 | 0 | 1 | 90 | 126.2 |
| 490 | 850.34 | 149.66 | 1173.47 | 0 | 200 | 14.03 | 0 | 0.2 | 23 | 1 | 1 | 0 | 0 | 0 | 1 | 7 | 126.2 |
| 491 | 890 | 0 | 1401.2 | 0 | 178 | 30.2 | 0 | 0.2 | 23 | 1 | 1 | 0 | 0 | 0 | 1 | 56 | 125.7 |
| 492 | 720 | 180 | 1310.4 | 0 | 180 | 9 | 0 | 0.2 | 23 | 1 | 1 | 0 | 0 | 0 | 1 | 28 | 125.56 |
| 493 | 850 | 212.5 | 935 | 255 | 212.5 | 21.2 | 8.5 | 0.2 | 23 | 1 | 1 | 4 | 10 | 0.44 | 1 | 28 | 125.5 |
| 494 | 788 | 197 | 866.8 | 315 | 173 | 14.77 | 0 | 0.18 | 23 | 1 | 1 | 0 | 0 | 0 | 1 | 14 | 125.5 |
| 495 | 845.5 | 44.5 | 1231 | 0 | 178 | 30.2 | 0 | 0.2 | 21 | 1 | 1 | 0 | 0 | 0 | 1 | 90 | 125.4 |
| 496 | 875 | 150 | 1100 | 0 | 180 | 35 | 8.75 | 0.18 | 23 | 1 | 1 | 4 | 24 | 0.023 | 1 | 28 | 125.4 |
| 497 | 720 | 80 | 1422.9 | 0 | 160 | 29.6 | 0 | 0.2 | 23 | 1 | 1 | 0 | 0 | 0 | 1 | 90 | 125.3 |
| 498 | 922 | 230 | 1152 | 0 | 184 | 11.52 | 13.83 | 0.16 | 23 | 1 | 1 | 4 | 12 | 0.018 | 1 | 7 | 125.1 |
| 499 | 750 | 250 | 1140 | 0 | 200 | 12 | 0 | 0.2 | 23 | 1 | 1 | 0 | 0 | 0 | 1 | 28 | 125 |
| 500 | 890 | 0 | 1401.2 | 0 | 178 | 30.2 | 0 | 0.2 | 23 | 1 | 1 | 0 | 0 | 0 | 1 | 28 | 124.9 |
| 501 | 874.9 | 43.7 | 1273.4 | 0 | 202.1 | 45.9 | 78 | 0.22 | 21 | 1 | 1 | 1 | 13 | 0.2 | 1 | 28 | 124.83 |
| 502 | 890 | 0 | 1401.2 | 0 | 178 | 30.2 | 0 | 0.2 | 23 | 1 | 1 | 0 | 0 | 0 | 1 | 56 | 124.5 |
| 503 | 720 | 180 | 1353.6 | 0 | 162 | 10.8 | 0 | 0.18 | 23 | 1 | 1 | 0 | 0 | 0 | 1 | 7 | 124.3 |
| 504 | 720 | 180 | 1303.2 | 0 | 180 | 10.8 | 0 | 0.2 | 23 | 1 | 1 | 0 | 0 | 0 | 1 | 28 | 124.2 |
| 505 | 720 | 80 | 1422.9 | 0 | 160 | 29.6 | 0 | 0.2 | 23 | 1 | 1 | 0 | 0 | 0 | 1 | 56 | 124.2 |
| 506 | 960 | 240 | 773.8 | 0 | 234 | 45 | 58.5 | 0.2 | 23 | 1 | 1 | 1 | 13 | 0.2 | 1 | 7 | 124.2 |
| 507 | 845.5 | 44.5 | 1231 | 0 | 178 | 30.2 | 0 | 0.2 | 21 | 1 | 1 | 0 | 0 | 0 | 1 | 56 | 124.1 |
| 508 | 800 | 0 | 1471.3 | 0 | 160 | 21.6 | 0 | 0.2 | 23 | 1 | 1 | 0 | 0 | 0 | 1 | 90 | 124.1 |
| 509 | 900 | 220 | 1005 | 0 | 163 | 40 | 156 | 0.15 | 23 | 1 | 1 | 1 | 13 | 0.2 | 1 | 7 | 124.1 |
| 510 | 828 | 207 | 911 | 248 | 186.4 | 21 | 0 | 0.18 | 23 | 1 | 1 | 0 | 0 | 0 | 1 | 7 | 124.1 |
| 511 | 845.5 | 44.5 | 1401.2 | 0 | 178 | 30.2 | 0 | 0.2 | 23 | 1 | 1 | 0 | 0 | 0 | 1 | 7 | 124 |
| 512 | 960 | 240 | 793.7 | 0 | 234 | 45 | 0 | 0.2 | 23 | 1 | 1 | 0 | 0 | 0 | 1 | 7 | 124 |
| 513 | 720 | 180 | 1296 | 0 | 180 | 12.6 | 0 | 0.2 | 23 | 1 | 1 | 0 | 0 | 0 | 1 | 7 | 124 |
| 514 | 850.34 | 149.66 | 1173.47 | 0 | 200 | 11.99 | 0 | 0.2 | 23 | 1 | 1 | 0 | 0 | 0 | 1 | 7 | 124 |
| 515 | 792 | 264 | 1056 | 0 | 173 | 21.1 | 156 | 0.16 | 20 | 1 | 1 | 1 | 13 | 0.2 | 1 | 7 | 123.66 |
| 516 | 800 | 200 | 1160 | 0 | 200 | 12 | 0 | 0.2 | 23 | 1 | 1 | 0 | 0 | 0 | 1 | 7 | 123.5 |
| 517 | 850.34 | 149.66 | 1181.97 | 0 | 200 | 10.03 | 0 | 0.2 | 23 | 1 | 1 | 0 | 0 | 0 | 1 | 7 | 123.5 |
| 518 | 675 | 225 | 1289.25 | 0 | 180 | 10.8 | 0 | 0.2 | 23 | 1 | 1 | 0 | 0 | 0 | 1 | 28 | 123.4 |
| 519 | 784 | 261 | 1045 | 0 | 171 | 20.9 | 23.52 | 0.16 | 23 | 1 | 1 | 6 | 13 | 0.2 | 1 | 7 | 123.2 |
| 520 | 850 | 212.5 | 935 | 255 | 212.5 | 21.2 | 0 | 0.2 | 23 | 1 | 1 | 0 | 0 | 0 | 1 | 28 | 123.2 |
| 521 | 809 | 270 | 1079 | 0 | 177 | 21.6 | 0 | 0.16 | 23 | 1 | 1 | 0 | 0 | 0 | 1 | 28 | 123.2 |
| 522 | 850 | 212.5 | 935 | 255 | 212.5 | 21.2 | 8.5 | 0.2 | 23 | 1 | 1 | 4 | 10 | 0.44 | 1 | 28 | 123 |
| 523 | 680 | 120 | 1502.8 | 0 | 144 | 9.59 | 0 | 0.18 | 23 | 1 | 1 | 0 | 0 | 0 | 1 | 28 | 122.93 |
| 524 | 850 | 150 | 1122 | 0 | 220 | 11.99 | 0 | 0.22 | 23 | 1 | 1 | 0 | 0 | 0 | 1 | 28 | 122.93 |
| 525 | 765 | 135 | 1315.8 | 0 | 180 | 10.79 | 0 | 0.2 | 23 | 1 | 1 | 0 | 0 | 0 | 1 | 28 | 122.6 |
| 526 | 960 | 240 | 771.9 | 0 | 234 | 45 | 58.5 | 0.2 | 23 | 1 | 1 | 1 | 13 | 0.2 | 1 | 7 | 122.6 |
| 527 | 890 | 222 | 837 | 0 | 222 | 29.6 | 0 | 0.2 | 23 | 2 | 1 | 0 | 0 | 0 | 1 | 28 | 122.4 |
| 528 | 784 | 261 | 1045 | 0 | 171 | 20.9 | 23.52 | 0.16 | 23 | 1 | 1 | 6 | 13 | 0.2 | 1 | 3 | 122.4 |
| 529 | 720 | 180 | 1310.4 | 0 | 180 | 9 | 0 | 0.2 | 23 | 1 | 1 | 0 | 0 | 0 | 1 | 7 | 122.3 |
| 530 | 890 | 0 | 1401.2 | 0 | 178 | 30.2 | 0 | 0.2 | 23 | 1 | 1 | 0 | 0 | 0 | 1 | 28 | 122.1 |
| 531 | 680 | 120 | 1462 | 0 | 160 | 11.22 | 0 | 0.2 | 23 | 1 | 1 | 0 | 0 | 0 | 1 | 28 | 122.05 |
| 532 | 720 | 180 | 1260 | 0 | 198 | 10.8 | 0 | 0.22 | 23 | 1 | 1 | 0 | 0 | 0 | 1 | 28 | 122.05 |
| 533 | 845.5 | 44.5 | 1401.2 | 0 | 178 | 30.2 | 0 | 0.2 | 23 | 1 | 1 | 0 | 0 | 0 | 1 | 7 | 121.9 |
| 534 | 960 | 240 | 780.5 | 0 | 234 | 45 | 39 | 0.2 | 23 | 1 | 1 | 1 | 13 | 0.2 | 1 | 7 | 121.8 |
| 535 | 850 | 212.5 | 935 | 255 | 212.5 | 21.2 | 17 | 0.2 | 23 | 1 | 1 | 4 | 10 | 0.44 | 1 | 28 | 121.6 |
| 536 | 890 | 0 | 1401.2 | 0 | 178 | 30.2 | 0 | 0.2 | 23 | 1 | 1 | 0 | 0 | 0 | 1 | 28 | 121.4 |
| 537 | 741 | 185 | 815 | 259 | 185 | 9 | 14.82 | 0.2 | 23 | 1 | 1 | 6 | 13 | 0.2 | 1 | 7 | 121.3 |
| 538 | 845.5 | 44.5 | 1401.2 | 0 | 178 | 30.2 | 0 | 0.2 | 23 | 1 | 1 | 0 | 0 | 0 | 1 | 7 | 121.3 |
| 539 | 958.55 | 50.45 | 1456.8 | 0 | 201.8 | 34.2 | 0 | 0.2 | 23 | 1 | 1 | 0 | 0 | 0 | 1 | 7 | 121.3 |
| 540 | 756.5 | 133.5 | 1231 | 0 | 178 | 30.2 | 0 | 0.2 | 21 | 1 | 1 | 0 | 0 | 0 | 1 | 90 | 120.9 |
| 541 | 845.5 | 44.5 | 1401.2 | 0 | 178 | 30.2 | 0 | 0.2 | 23 | 1 | 1 | 0 | 0 | 0 | 1 | 7 | 120.9 |
| 542 | 874.9 | 43.7 | 1273.4 | 0 | 202.1 | 45.9 | 156 | 0.22 | 21 | 1 | 1 | 1 | 13 | 0.2 | 1 | 7 | 120.81 |
| 543 | 750.19 | 249.81 | 1140.29 | 0 | 200 | 12 | 0 | 0.2 | 23 | 1 | 1 | 0 | 0 | 0 | 1 | 7 | 120.8 |
| 544 | 720 | 80 | 1422.9 | 0 | 160 | 29.6 | 0 | 0.2 | 23 | 1 | 1 | 0 | 0 | 0 | 1 | 28 | 120.7 |
| 545 | 720 | 180 | 1303.2 | 0 | 180 | 10.8 | 0 | 0.2 | 23 | 1 | 1 | 0 | 0 | 0 | 1 | 7 | 120.6 |
| 546 | 1009 | 0 | 1456.8 | 0 | 201.8 | 34.2 | 0 | 0.2 | 23 | 1 | 1 | 0 | 0 | 0 | 1 | 7 | 120.5 |
| 547 | 960 | 240 | 779.2 | 0 | 234 | 45 | 39 | 0.2 | 23 | 1 | 1 | 1 | 13 | 0.2 | 1 | 7 | 120.2 |
| 548 | 1116.7 | 390.85 | 625.35 | 0 | 212.17 | 23.45 | 0 | 0.14 | 23 | 1 | 1 | 0 | 0 | 0 | 1 | 7 | 120.2 |
| 549 | 845.5 | 44.5 | 1231 | 0 | 178 | 30.2 | 0 | 0.2 | 21 | 1 | 1 | 0 | 0 | 0 | 1 | 56 | 120.1 |
| 550 | 756.5 | 133.5 | 1231 | 0 | 178 | 30.2 | 0 | 0.2 | 21 | 1 | 1 | 0 | 0 | 0 | 1 | 56 | 120.1 |

Appendix B**:** Existing ML methods for predicting UHPC compressive strength and SHAP analysis most influential features.

*Trees Models*

Across all base models [**Fig. 4-1**], the silica fume content (SF) consistently emerges as the most influential variable, exhibiting high SHAP values in both positive and negative directions. This indicates that variations in SF content have a substantial and bidirectional effect on the model output, with higher or lower values leading to significant shifts in predicted strength.


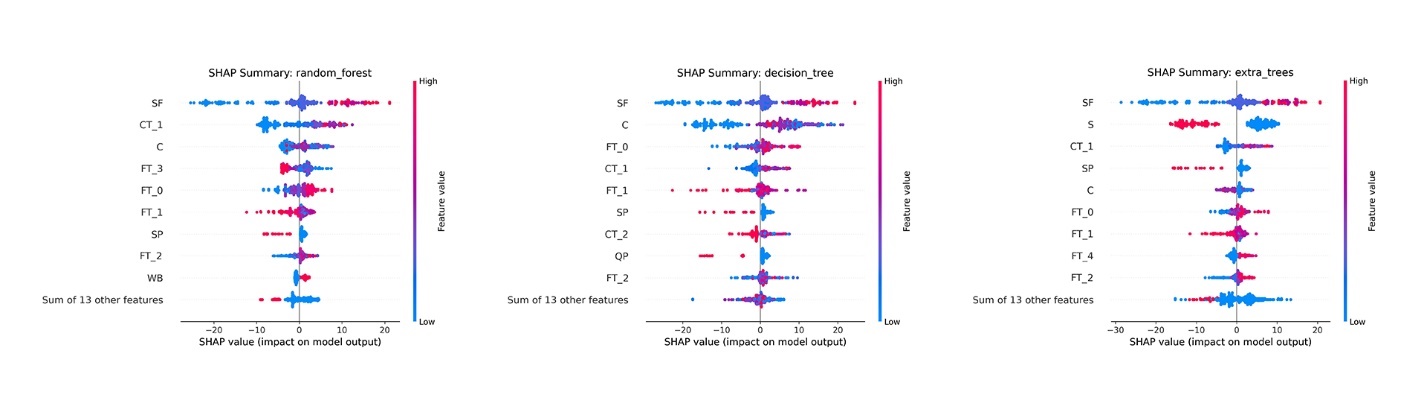


**Fig. ‎4‑1:** SHAP Analysis for Non-Optimized Tree Models

The SHAP summary plots for the Decision Tree models optimized with various metaheuristic algorithms (ABC, DE, Firefly, GA, HS, PSO, and SA) [**Fig. ‎4‑2**] provide valuable insights into how each optimization strategy affects feature prioritization in predicting the compressive strength of UHPC. Across all optimization methods, two features; Curing Age (A) and sand content (S) consistently appear within the top three most influential variables, indicating their dominant role in determining strength outcomes regardless of optimization approach.
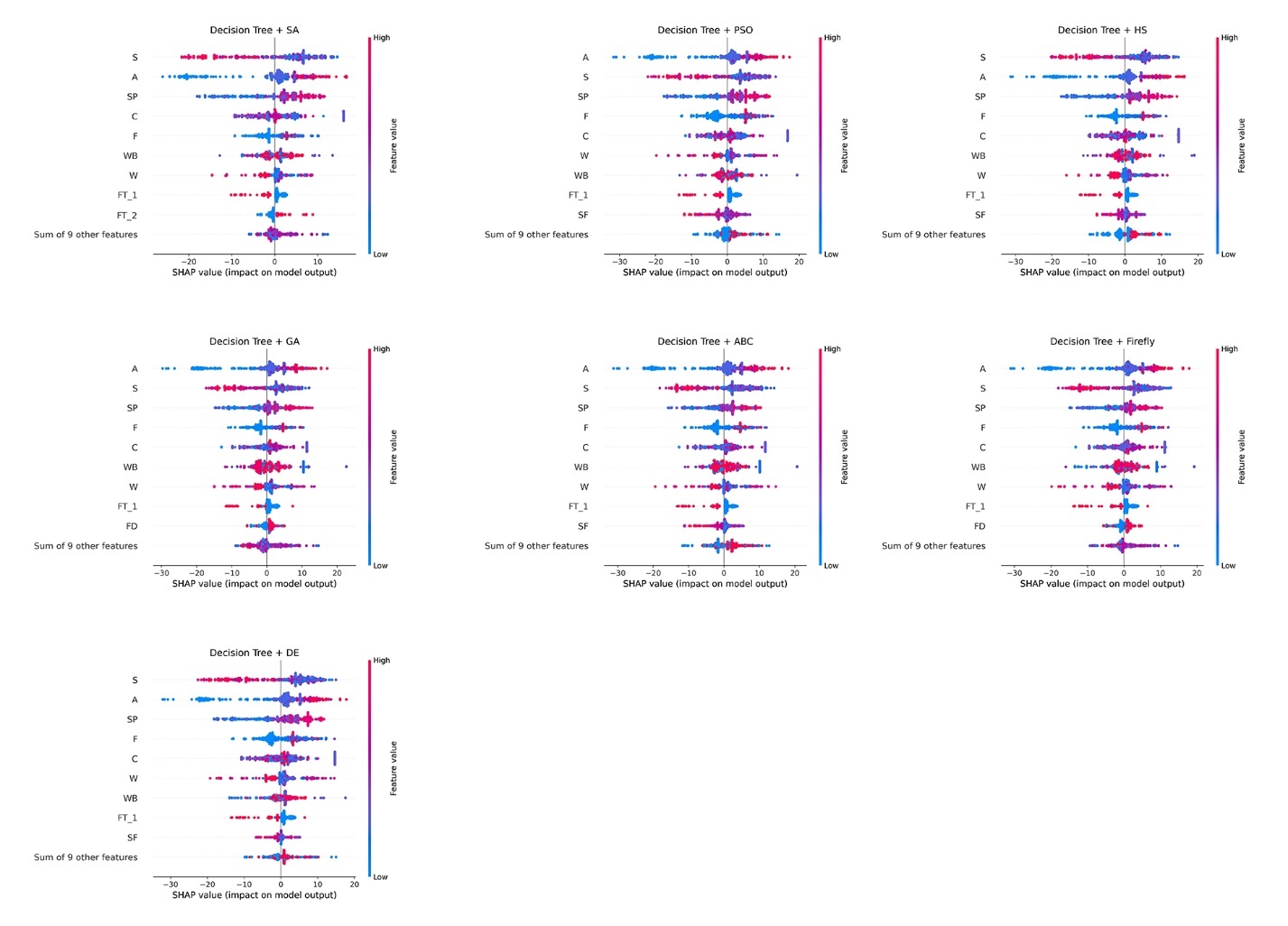


**Fig. ‎4‑2:** SHAP Analysis for Optimized Decision Tree Models

The SHAP summary plots for the Extra Trees models optimized with different metaheuristic algorithms (ABC, DE, Firefly, GA, HS, PSO, and SA) [**Fig. ‎4‑3**] reveal both strong consistencies and notable variations in the feature rankings used to predict UHPC compressive strength. Across all optimization methods, curing age (A) dominates as the most influential predictor, consistently occupying the first rank. This consistency underscores the fundamental role of curing age in determining UHPC mechanical performance, regardless of the optimization strategy.


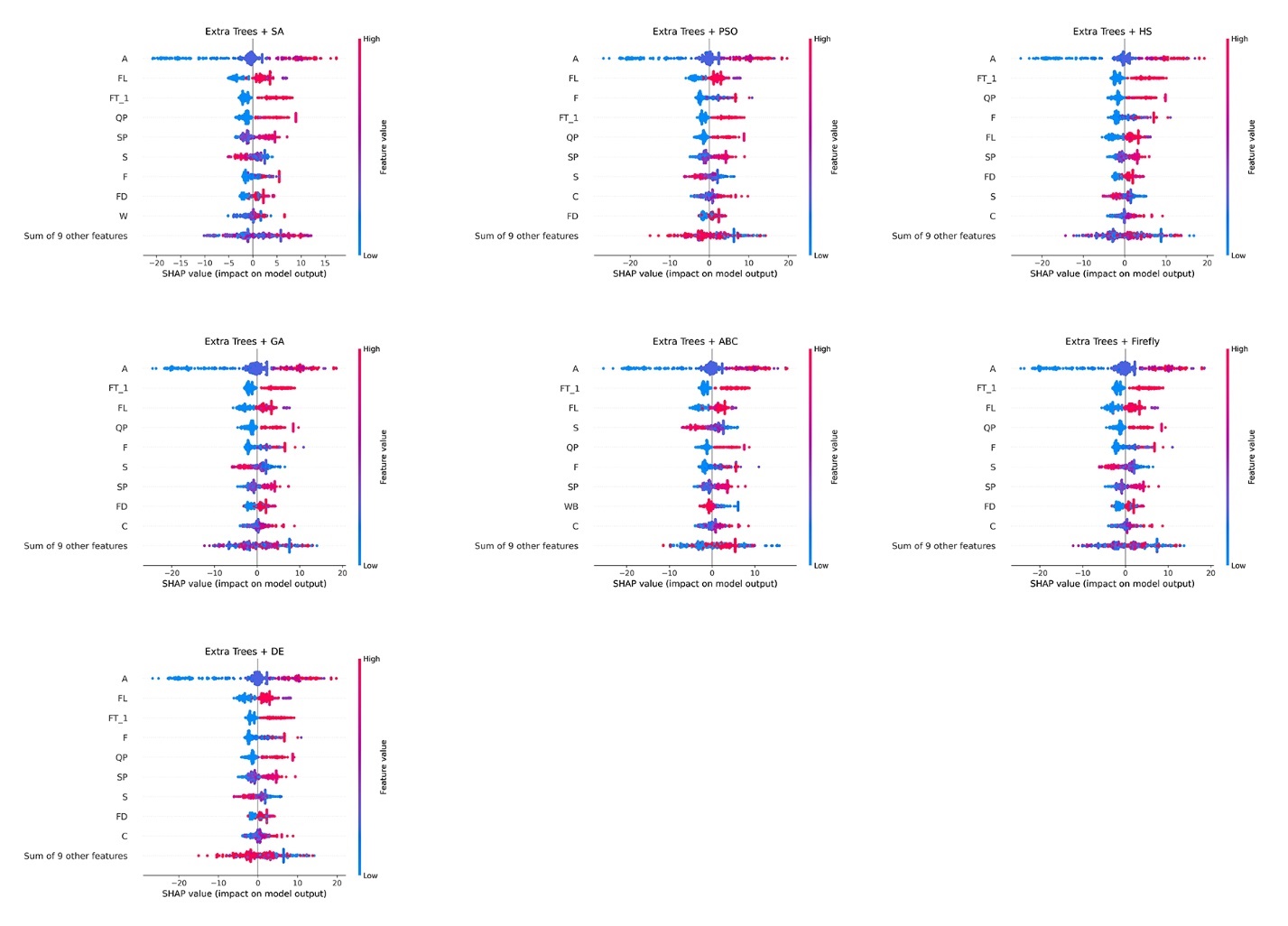


**Fig. ‎4‑3:** SHAP Analysis for Optimized Extra Tree Models

Across all Random Forest models optimized with the seven metaheuristic algorithms [**Fig. 4-4**] (PSO, SA, ABC, DE, Firefly, GA, and HS), a consistent pattern of feature influence emerges. Curing Age (A) is the most dominant predictor, with higher values strongly associated with increased compressive strength, followed by Sand (S), which also shows a robust positive impact. In contrast, Fiber content (F) consistently acts as the strongest negative driver, where higher values reduce predictions across all models. Fiber Diameter (FD) demonstrates a minor but generally positive effect, while features such as Cement (C), Water (W), and Superplasticizer (SP) remain marginal, with SHAP values tightly clustered around zero, indicating negligible influence. Although some models (e.g., Firefly, DE) reveal slightly broader or more scattered SHAP distributions for secondary features, the overarching trend confirms that the models’ predictive capacity is largely governed by the interplay of curing age, sand content, and fiber dosage, regardless of the optimization technique employed.


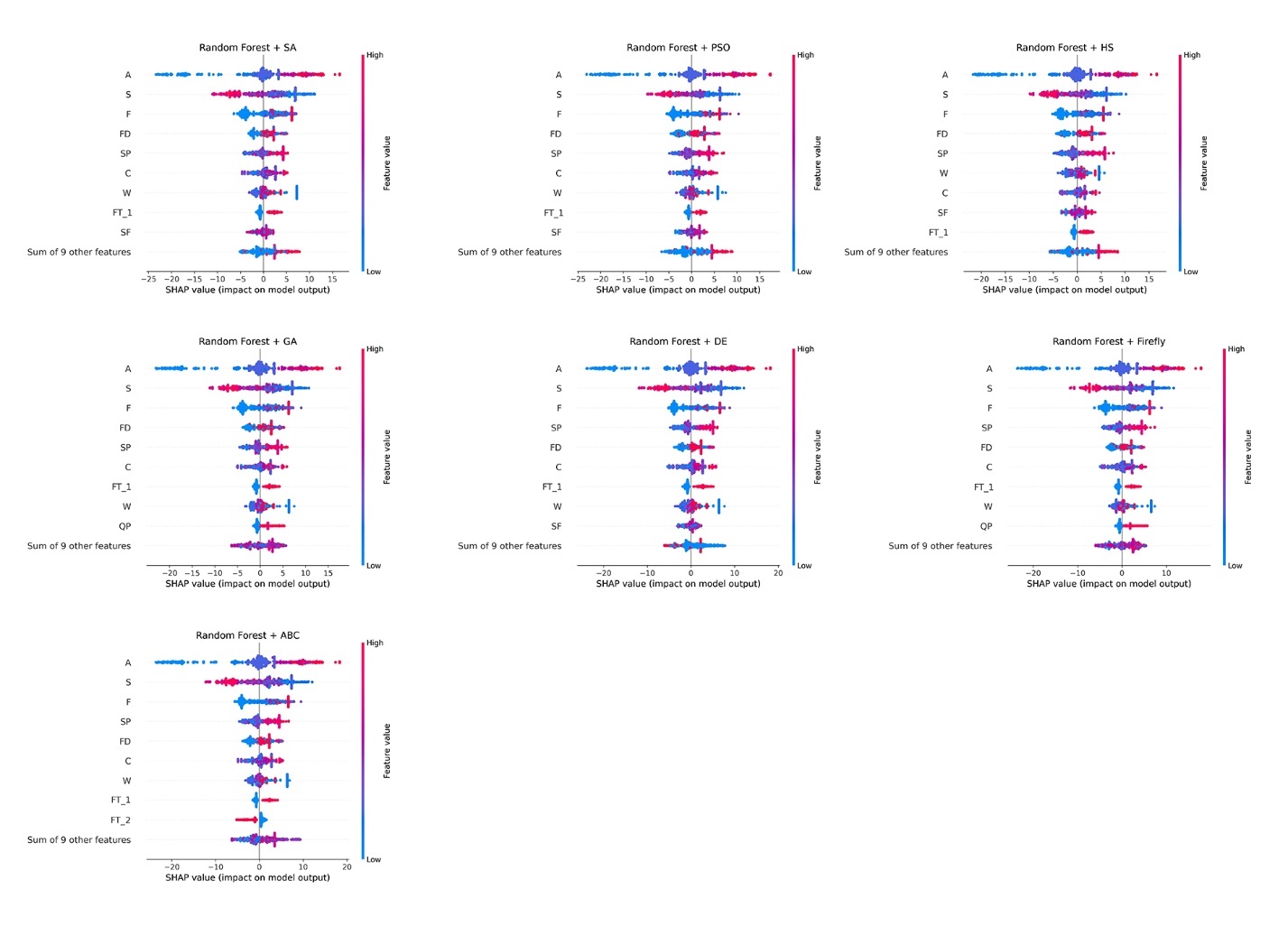


**Fig. ‎4‑4:** SHAP Analysis for Optimized Random Forest Models

*Boosting Models*

The SHAP analysis for boosting models is crucial to understand the model’s behavior. However, the SHAP analysis for the Adaboost model was excluded as Adaboost was significantly the worst performing model among the other three models. For the non-optimized boosting models (XGBoost, CatBoost, and LightGBM) [**Fig. ‎4-5**], Silica Fume (SF) consistently emerges as the most influential feature, where low dosages strongly reduce predictions and higher dosages enhance them. CT_ also plays a major role across all three models, with higher values generally exerting a negative influence on compressive strength, while lower values tend to increase predictions. FT_0 is another key driver, consistently showing a positive impact, whereas FT_1 and FT_3 have smaller but noticeable effects, mostly positive. The Water-Binder Ratio (WB), particularly emphasized in the CatBoost and LightGBM models, shows a clear negative relationship, with higher values reducing predicted strength. Cement (C) contributes positively across models, with its influence being especially distinct in LightGBM. Meanwhile, Superplasticizer (SP) shows only a minor but consistent positive effect. Collectively, these findings highlight SF, CT_1, and FT_0 as the dominant features, with WB and Cement providing additional influence depending on the model structure.

Across all optimized boosting models (CatBoost, LightGBM, and XGBoost) [**Figs. ‎4-6 to ‎4-8**], the SHAP analyses reveal a highly consistent hierarchy of influential features. Curing Age (A) is the dominant positive driver across all models, where higher values strongly increase predicted compressive strength. In contrast, Fiber content (F) consistently emerges as the most influential negative contributor, with higher values reducing predictions. Superplasticizer (SP) and Sand (S) also play important roles, showing a clear and repeated positive influence across models, while Fiber Diameter (FD) provides an additional positive contribution, albeit to a lesser extent. Cement (C) is occasionally highlighted, particularly in the GA-optimized XGBoost model, where its positive effect becomes more distinct. FT_1 and Fiber Length (FL) show only marginal impacts, with their SHAP values generally clustered near zero. The consistency of these findings across all optimization algorithms emphasizes that the predictive performance of boosting models is primarily governed by the interplay between curing age, fiber dosage, superplasticizer content, and sand proportion, while the contributions of secondary features remain comparatively minor
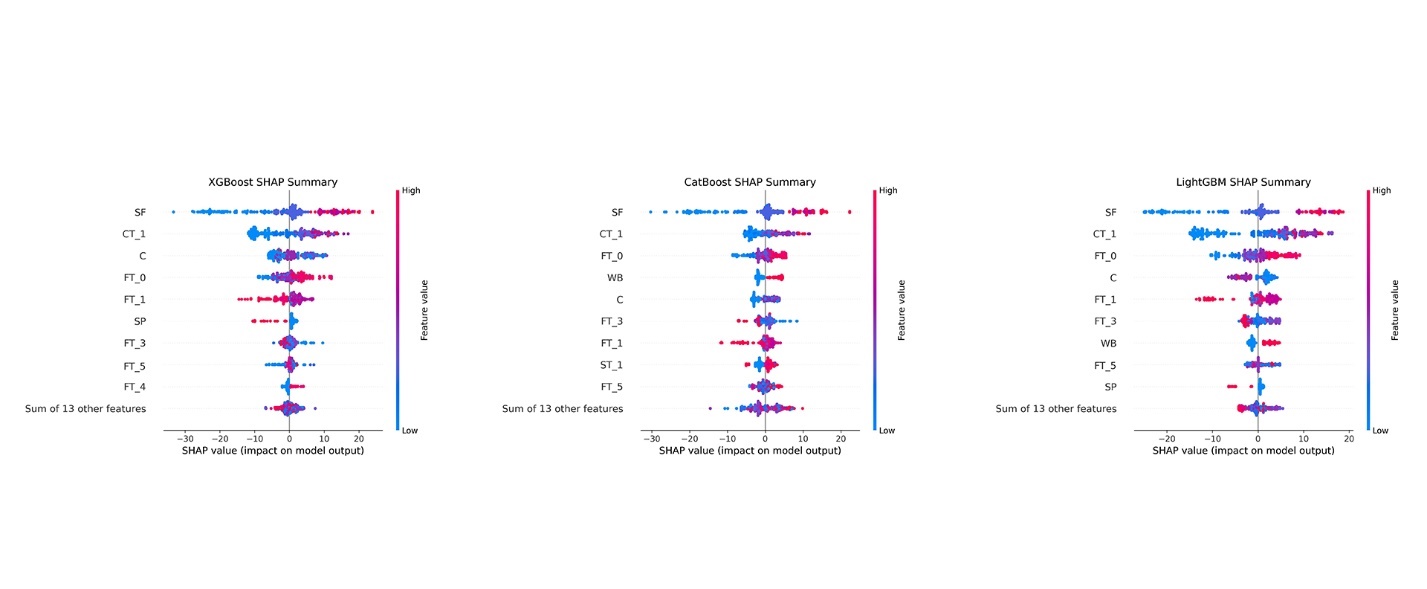


**Fig. ‎4‑5:** SHAP Analysis results for Non-Optimized Boosting Models


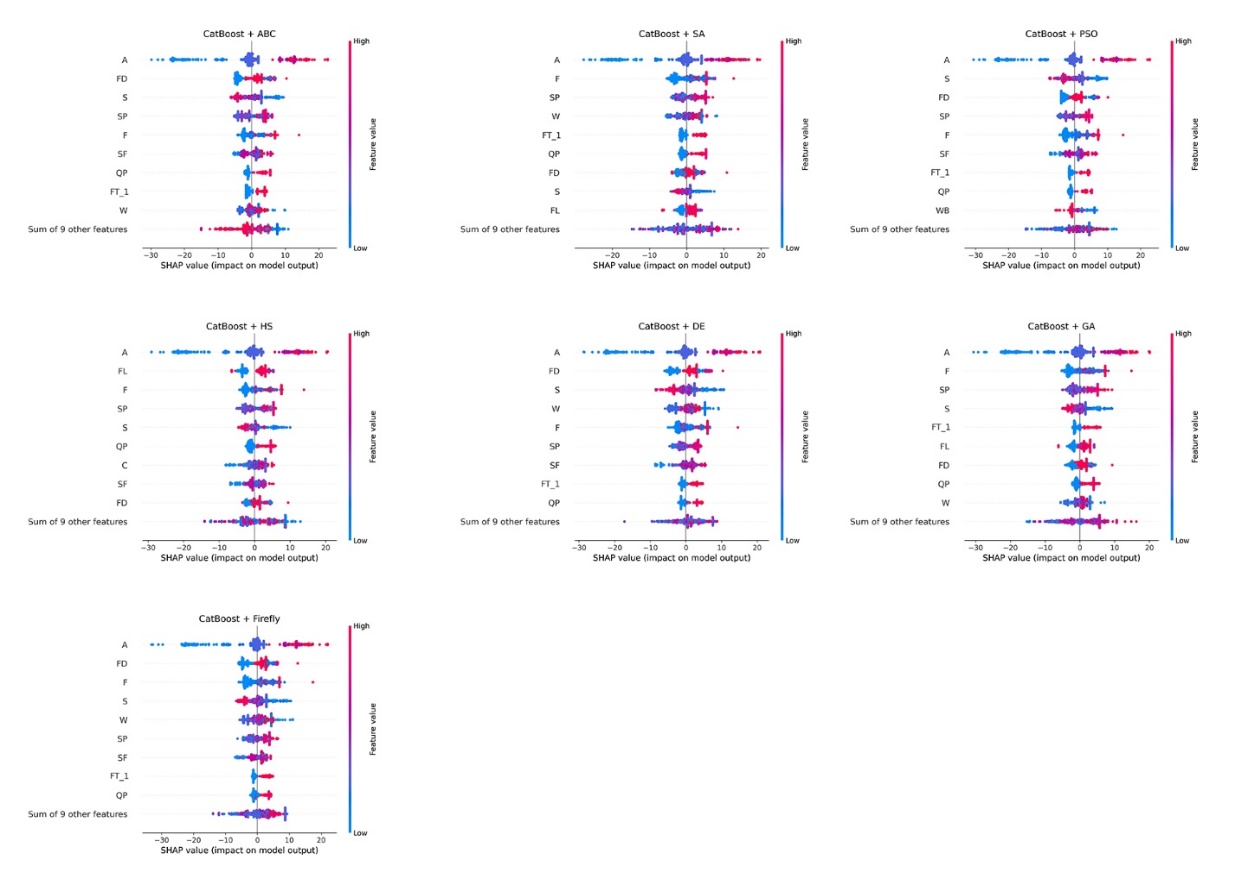


**Fig. ‎4‑6:** SHAP Analysis results for Optimized Catboost Models


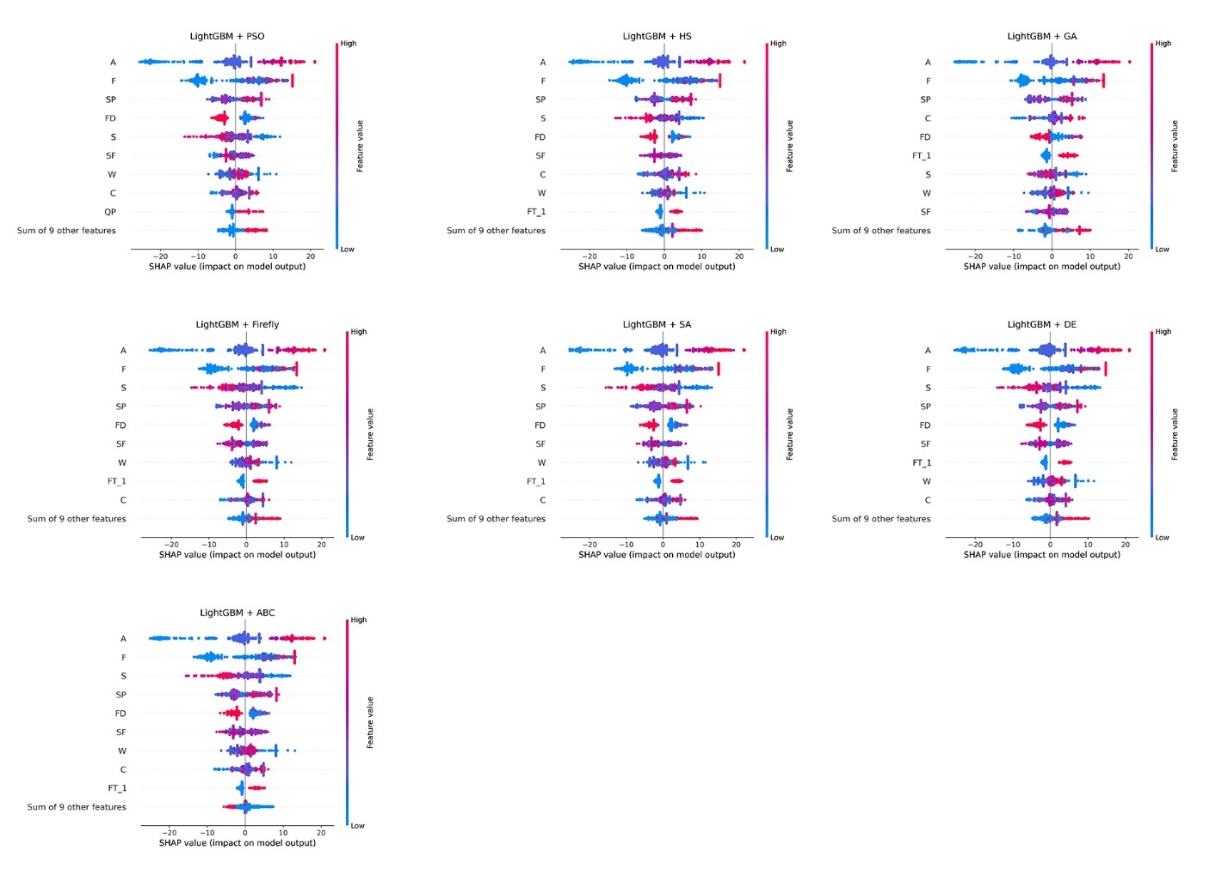


**Fig. ‎4‑7:** SHAP Analysis Results for Optimized LightGBM Models


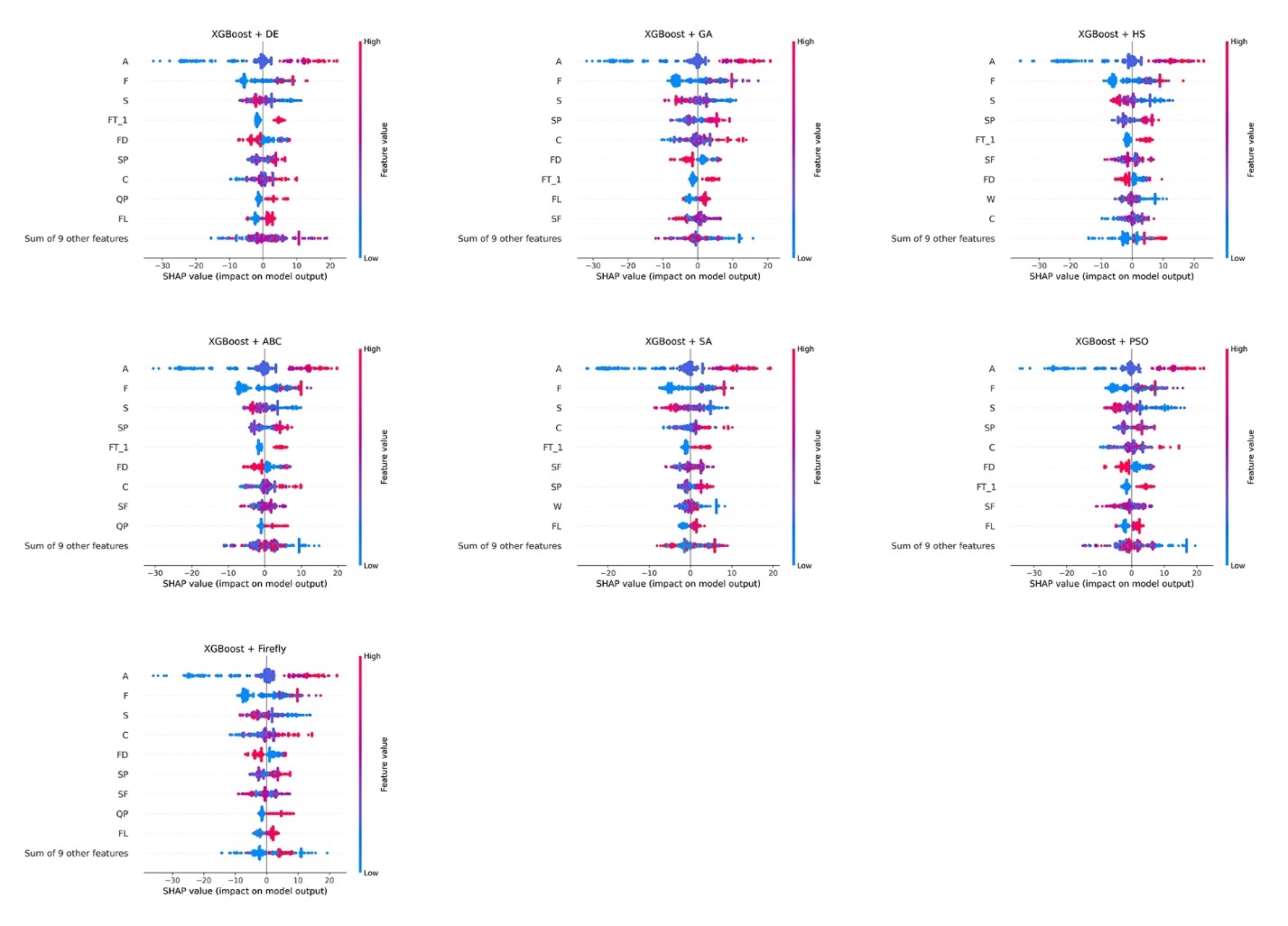


**Fig. ‎4‑8:** SHAP Analysis Results for Optimized XGBoost Models

*Support Vector Machines Models*

For the non-optimized support vector models [**Fig. 4-9**] (NuSVR and SVR), the SHAP analyses consistently identify Silica Fume (SF) and Water-Binder Ratio (WB) as the most influential features. SF exhibits a strong positive correlation across both models, where higher values significantly increase predictions, while WB shows an equally strong but negative influence, with higher values consistently lowering predictions. FT_0 and Sand (S) also emerge as notable positive contributors, while FT_4 and FT_2 provide additional but smaller positive impacts in the SVR model. CT_1 and Cement (C) display a more mixed yet generally positive influence, whereas features such as Fiber (F), Superplasticizer (SP), Water (W), and Curing Temperature (T) have minimal relevance, with their SHAP values clustered near zero. Collectively, these findings demonstrate that the predictive performance of both NuSVR and SVR models is primarily governed by the balance between silica fume dosage and water-binder ratio, with secondary contributions from specific fiber types and sand content.


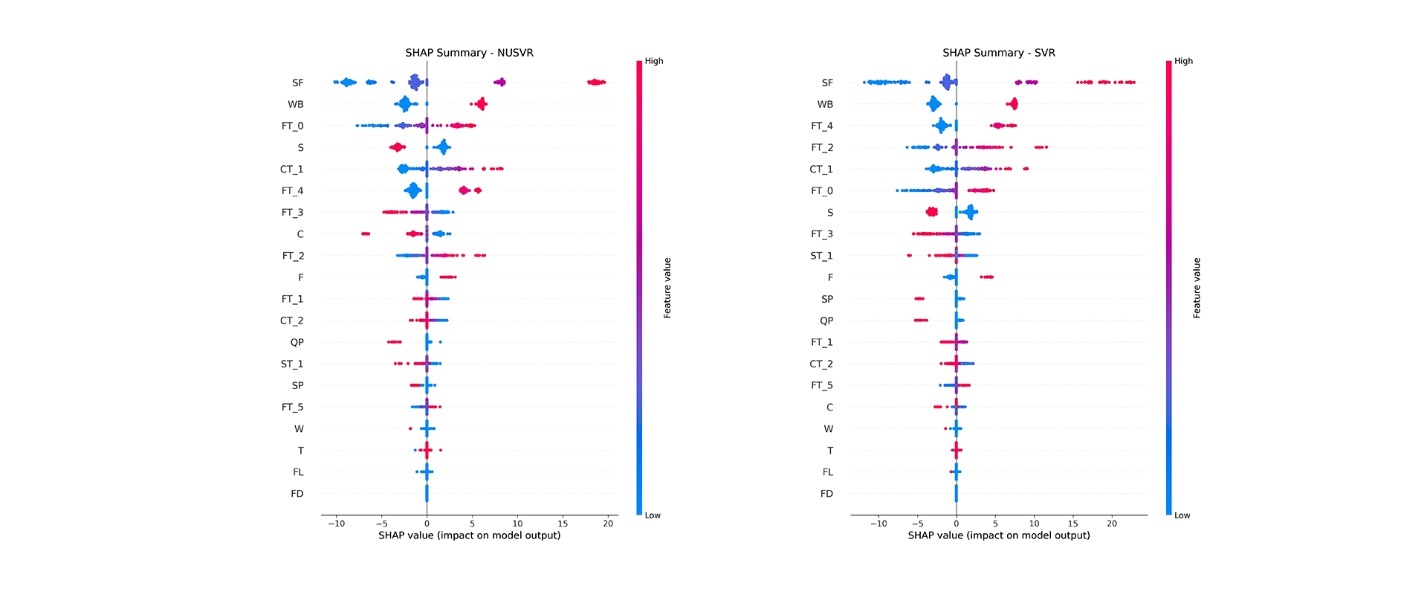


**Fig. ‎4‑9:** SHAP Analysis Results for Non-Optimized SVM Models

Across all optimized SVR and NuSVR models [**Figs. ‎4-10 and ‎4-11**], the SHAP analyses reveal a consistent pattern in feature importance. Curing Age (A) is the dominant positive driver across all optimizations, where higher values strongly enhance predictions of compressive strength. Fiber content (F) consistently acts as the strongest negative contributor, with higher values reducing predictions. Quartz Powder (QP) emerges as a major positive feature alongside A, while Superplasticizer (SP) also shows a clear positive impact, particularly in the DE- and Firefly-optimized models. Secondary contributions include Fiber Diameter (FD), Cement (C), and FT_1, which show subtle but generally positive influences, and Fiber Length (FL), which often exhibits a minor negative effect. Features such as Water (W) and Silica Fume (SF) remain negligible across models, with their SHAP values clustered near zero. Collectively, these results confirm that the optimized SVR and NuSVR models primarily rely on the interplay of curing age, quartz powder, fiber dosage, and superplasticizer, while the contributions of other features are comparatively limited.


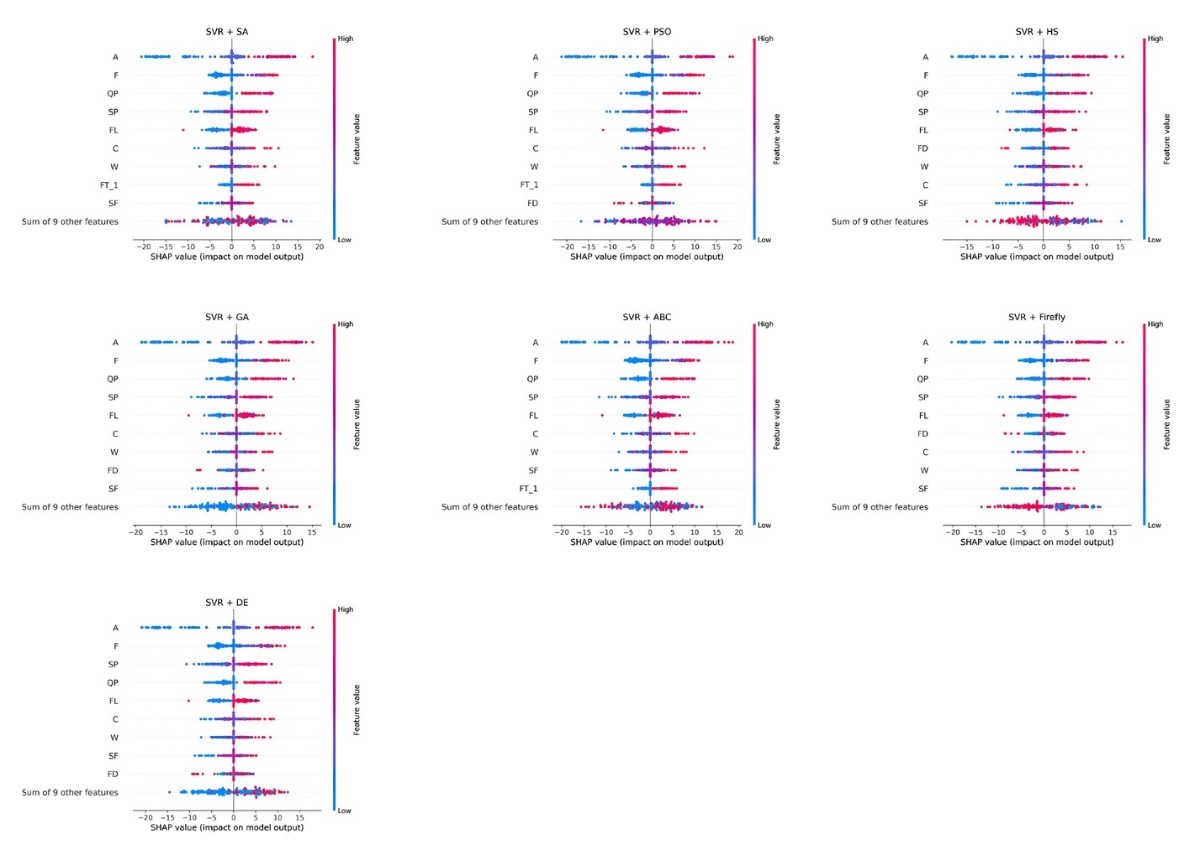


**Fig. ‎4‑10:** SHAP Analysis Results for Optimized SVR Models


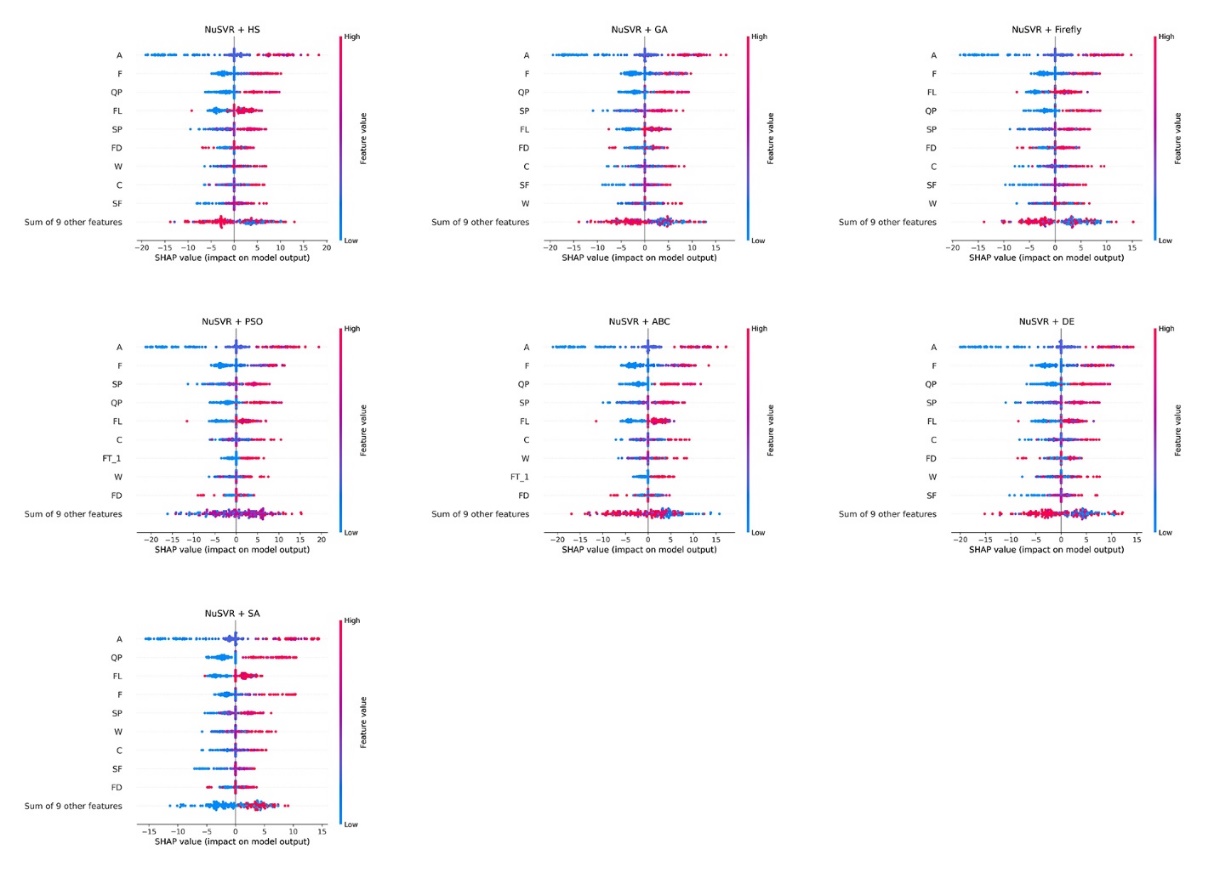


**Fig. ‎4‑11:** SHAP Analysis Results for Optimized NuSVR Model

Appendix C**:** Models' Hyperparameters

| **Model** | **Hyperparameter** | **Search Range** |
| --- | --- | --- |
| Decision Tree | max_depth | 5 – 20 |
|  | min_samples_split | 2 – 15 |
|  | min_samples_leaf | 1 – 5 |
| Random Forest | n_estimators | 50 – 120 |
|  | max_depth | 5 – 20 |
|  | min_samples_split | 2 – 10 |
|  | min_samples_leaf | 1 – 5 |
|  | max_features | 0.3 – 0.8 |
| Extra Trees | n_estimators | 50 – 120 |
|  | max_depth | 5 – 20 |
|  | min_samples_split | 2 – 10 |
|  | min_samples_leaf | 1 – 5 |
|  | max_features | 0.3 – 0.8 |
| XGBoost | n_estimators | 50 – 150 |
|  | max_depth | 3 – 8 |
|  | learning_rate | 0.05 – 0.2 |
|  | subsample | 0.7 – 1.0 |
|  | colsample_bytree | 0.6 – 1.0 |
| AdaBoost | n_estimators | 30 – 100 |
|  | learning_rate | 0.05 – 0.5 |
| CatBoost | iterations | 50 – 150 |
|  | depth | 3 – 6 |
|  | learning_rate | 0.05 – 0.2 |
| LightGBM | n_estimators | 50 – 150 |
|  | max_depth | 3 – 8 |
|  | learning_rate | 0.05 – 0.2 |
|  | num_leaves | 20 – 80 |
|  | subsample | 0.7 – 1.0 |
| SVR | C | 1.0 – 100.0 |
|  | epsilon | 0.01 – 0.1 |
|  | gamma | 0.001 – 0.5 |
| NuSVR | nu | 0.1 – 0.9 |
|  | C | 1.0 – 100.0 |
|  | gamma | 0.001 – 0.5 |

Appendix D**:** Metaheuristic Optimizers Hyperparameters

| **Model** | **Hyperparameter** | **Value** |
| --- | --- | --- |
| Particle Swarm Optimization (PSO) | n_particles | 25 |
|  | max_iter | 35 |
|  | w | 0.6 |
|  | c1 | 1.2 |
|  | c2 | 2.2 |
| Genetic Algorithm (GA) | pop_size | 24 |
|  | generations | 35 |
|  | mutation_rate | 0.25 |
|  | crossover_rate | 0.75 |
| Firefly Algorithm (FFA) | n | 22 |
|  | max_iter | 33 |
|  | alpha | 0.6 |
|  | beta | 1.0 |
|  | gamma | 1.5 |
| Simulated Annealing (SA) | max_iter | 40 |
|  | T0 | 120 |
|  | alpha | 0.85 |
| Differential Evolution (DE) | pop_size | 20 |
|  | max_iter | 30 |
|  | F | 0.5 |
|  | CR | 0.7 |
| Artificial Bee Colony (ABC) | pop_size | 20 |
|  | max_iter | 30 |
|  | limit | 5 |
| Harmony Search (HS) | hm_size | 20 |
|  | max_iter | 30 |
|  | HMCR | 0.9 |
|  | PAR | 0.3 |
|  | bw | 0.01 |

Appendix E**:** Comparing Existing ML methods for predicting UHPC compressive strength and SHAP analysis most influential features

| **N°** | **References** | **Dataset size** | **ML models** | **Target of prediction** | **Hyperparameters tuning** | **Input features** | **Best Performing Model** | **SHAP Most Influential Features** |
| --- | --- | --- | --- | --- | --- | --- | --- | --- |
|  | Shen et al. [84] | 372 | XGBoost; AdaBoost; Bagging | Compressive Strength of UHPC | K-fold cross-validation | **10 input variables :**  *C;FA;SF;Silicate;S;W;SP;Steel Fiber;Fiber Aspect Ratio;Curing Time* | **XGBoost:**  R^2^=0.90  RMSE =7.6 MPa  MAE=6.4 MPa | 1^st^: Curing Time  2^nd^: Silica Fume Content  3^rd^: Sand Content |
|  | Qian et al. [74] | 626 | Decision Tree; Bootstrap Aggregating; Gradient Boosting | Compressive Strength of UHPC | K-fold cross-validation | **21 input variables :**  *C; FA; SF; Slag; NanoSilica; QP;LimePowder;S; Coarse Aggregate; SP; Steel Fiber; Polystyrene Fiber; W; Strength of Cement; Max. Agg. Size;Steel FD;Steel FL, Poly FD; Poly FL* | **Gradient Boosting:**  R^2^=0.93  RMSE =9.2 MPa  MAE=7.0 MPa | 1st: Curing Time  2^nd^: Silica Fume Content  3^rd^: Sand Content |
|  | Alabduljabbar [18] | 810 | Gene Expression Programming (GEP) | Compressive Strength of UHPC | Not Mentioned | **15 input variables :**  *C;W;NanoSilica;QP;LimePowder;Gravel;S;Slag;SP;F;T;A;FA;RelativeHumidity;SF* | **GEP :**  **Train :**  RMSE:6.5 MPa  MAE:4.9 MPa  RSE:0.512  **Testing:**  RMSE:4.7 MPa  MAE:3.6 MPa  RSE:0.0037 | 1^st^: Curing Age  2^nd^: Fiber Content  3^rd^: Silica Fume Content |

**Appendix E:** Comparing Existing ML methods for predicting UHPC compressive strength and SHAP analysis most influential features ***cont.***

| **N°** | **References** | **Dataset size** | **ML models** | **Target of prediction** | **Hyperparameters tuning** | **Input features** | **Best Performing Model** | **SHAP Most Influential Features** |
| --- | --- | --- | --- | --- | --- | --- | --- | --- |
|  | Nguyen et al. [17] | 810 | XGBoost; Ada-XGBoost; Bagging-XGBoost; Voting-XGBoost; Stacking-XGBoost. | Compressive Strength of UHPC | Monte Carlo simulation, and 10-fold cross-validation | **15 input variables :**  *C;W;NanoSilica;QP;LimePowder;Gravel;S;Slag;SP;F;T;A;FA;RelativeHumidity;SF* | **Stacking-XGBoost:**  **Train:**  R^2^=0.974  RMSE=6.5MPa  MAE=5.06MPa  **Testing:**  R^2^=0.973  RMSE=6.67MPa  MAE=5.109 | 1^st^: Curing Age  2^nd^: Fiber Content  3^rd^: Silica Fume Content |
|  | Aydin et al. [85] | 890 | MLP; Stacking Ensemble | Compressive Strength of UHPC | Grid search | **13 input variables :**  *C;Slag;LimePowder;QP;FA;NanoSilica;Aggregate;W;F;SP;T;A* | **Stacking Ensemble**:  R^2^=0.971 | 1^st^: Curing Age  2^nd^: Silica Fume Content  3^rd^: Fiber Content |

**Appendix E:** Comparing Existing ML methods for predicting UHPC compressive strength and SHAP analysis most influential features ***cont.***

| **N°** | **References** | **Dataset size** | **ML models** | **Target of prediction** | **Hyperparameters tuning** | **Input features** | **Best Performing Model** | **SHAP Most Influential Features** |
| --- | --- | --- | --- | --- | --- | --- | --- | --- |
| 6 | Kashem et al. [86] | 810 | Random Forest; AdaBoost; Gradient Boosting | Compressive Strength of UHPC | Particle Swarm Optimization | **15 input variables :**  *C;W;NanoSilica;QP;LimePowder;Gravel;S;Slag;SP;F;T;A;FA;RelativeHumidity;SF* | **Gradient Boosting:**  **Train:**  R^2^=0.991  RMSE=3.69MPa  MAE=2.16MPa  MAPE=1.7157  **Testing:**  R^2^=0.98  RMSE=5.94MPa  MAE=4.2MPa  MAPE=3.9207 | 1^st^: Curing Age  2^nd^: Fiber Content  3^rd^: Cement Content |
| 7 | Katlav & Ergen [87] | 785 | CatBoost. | Compressive Strength of UHPC | Phasor Particle Swarm Optimization (PPSO); Dwarf Mangoose Optimization (DMO); Atom Search Optimization (ASO) | **15 input variables :**  *C;W;NanoSilica;QP;LimePowder;Gravel;S;Slag;SP;F;T;A;FA;RelativeHumidity;SF* | **CatBoost-DMO :**  **Train:**  R^2^=0.993  RMSE=3.67MPa  MAE=2.35MPa  MAPE=0.019  **Test :**  R^2^=0.978  RMSE=6.15MPa  MAE=4.51MPa  MAPE=0.038 | 1^st^: Curing Age  2^nd^: Fiber Content  3^rd^: Cement Content |

**Appendix E:** Comparing Existing ML methods for predicting UHPC compressive strength and SHAP analysis most influential features ***cont.***

| **N°** | **References** | **Dataset size** | **ML models** | **Target of prediction** | **Hyperparameters tuning** | **Input features** | **Best Performing Model** | **SHAP Most Influential Features** |
| --- | --- | --- | --- | --- | --- | --- | --- | --- |
| 8 | Present study | 550 | Decision Tree, Random Forest, Extra Trees; XGBoost; AdaBoost; CatBoost; LightGBM; SVR; NuSVR. | Compressive Strength of UHPC | Particle Swarm Optimization (PSO); Genetic Algorithm (GA); Simulated Annealing (SA); Differential Evolution (DE); Artificial Bee Colony (ABC); Harmony Search (HS); and Firefly Algorithm (FFA) | **16 input variables :**  *C;SF;QP;F;W;SP;WB;T;FT;FL;FD;CT;SPB;ST;A* | **DE-Random Forest:**  **Train:**  R^2^=0.912  RMSE=6.312MPa  MAE=3.707MPa  MAPE=2.418  **Test:**  R^2^=0.867  RMSE=8.704MPa  MAE=6MPa  MAPE=3.854  **PSO-XGBoost:**  **Train:**  R^2^=0.876  RMSE=7.5MPa  MAE=5.192MPa  MAPE=0.93  **Test:**  R^2^=0.897  RMSE=7.632MPa  MAE=5.917MPa  MAPE=3.819 | **DE-Random Forest:**  1^st^: Curing Age  2^nd^: Silica Fume Content  3^rd^: Fiber Content  **PSO-XGBoost:**  1^st^: Curing Age  2^nd^: Fiber Content  3^rd^: Silica Fume Content |
